# Play, Learn, and Teach Outdoors—Network (PLaTO-Net): terminology, taxonomy, and ontology

**DOI:** 10.1186/s12966-022-01294-0

**Published:** 2022-06-15

**Authors:** Eun-Young Lee, Louise de Lannoy, Lucy Li, Maria Isabel Amando de Barros, Peter Bentsen, Mariana Brussoni, Tove Anita Fiskum, Michelle Guerrero, Bjørg Oddrun Hallås, Susanna Ho, Catherine Jordan, Mark Leather, Greg Mannion, Sarah A. Moore, Ellen Beate Hansen Sandseter, Nancy L. I. Spencer, Susan Waite, Po-Yu Wang, Mark S. Tremblay, participating PLaTO-Net members, Mary Louise Adams, Christine Alden, Salomé Aubert, Marie-Claude Beaudry, Félix Berrigan, Alan Champkins, Rita Cordovil, Émilie McKinnon-Côté, Patrick Daigle, Iryna Demchenko, Jan Ellinger, Guy Faulkner, Tanya Halsall, David Harvey, Stephen Hunter, Richard Irvine, Rachel Jones, Avril Johnstone, Anders Wånge Kjellsson, Yannick Lacoste, Rachel A. Larimore, Richard Larouche, Frederico Lopes, Helen Lynch, Christoph Mall, Taru Manyanga, Anne Martin, Gail Molenaar, Shawnda A. Morrison, Jorge Mota, Zoi Nikiforidou, Alexandra Parrington, Katie Parsons, Mathieu Point, Shelagh Pyper, Stephen D. Ritchie, Martin van Rooijen, Vanessa Scoon, Martyn Standage, Michelle Stone, Son Truong, Riaz Uddin, Diego Augusto Santos Silva, Leigh M. Vanderloo, Rosemary Welensky, Erin Wentzell, Øystein Winje, Megan Zeni, Milos Zorica

**Affiliations:** 1 School of Kinesiology & Health Studies, Queen’s University, Kingston, ON Canada; 2 Outdoor Play Canada, Children’s Hospital of Eastern Ontario Research Institute, Ottawa, ON Canada; 3 Instituto Alana, São Paulo, Brazil; 4 Copenhagen University Hospital – Bispebjerg and Frederiksberg and University of Copenhagen, Copenhagen, Denmark; 5 School of Population & Public Health, University of British Columbia, Vancouver, BC Canada; 6 Nord University, Bodø, Norway; 7 Children’s Hospital of Eastern Ontario Research Institute, Ottawa, ON Canada; 8 Western Norway University of Applied Sciences, Bergen, Norway; 9 Singapore University of Social Sciences, Singapore & Ministry of Education, Singapore, Singapore; 10 University of Minnesota & Children & Nature Network, Minneapolis, Minnesota USA; 11 Plymouth Marjon University, Plymouth, UK; 12 Faculty of Social Sciences, University of Stirling, Stirling, Scotland; 13 School of Health and Human Performance, Dalhousie University, Halifax, NS Canada; 14 Queen Maud University College, Trondheim, Norway; 15 Faculty of Kinesiology, Sport, and Recreation, University of Alberta, Edmonton, AB Canada; 16 University of Plymouth, United Kingdom & Jonkoping University, Jönköping, Sweden; 17 Department of Recreational Sport, National Taiwan University of Sport, Taiwan Taichung, Republic of China; 18 Healthy Active Living and Obesity Research Group, CHEO Research Institute, 401 Smyth Rd, Ottawa, Ontario K1H 8L1 Canada

## Abstract

**Background:**

A recent dialogue in the field of play, learn, and teach outdoors (referred to as “PLaTO” hereafter) demonstrated the need for developing harmonized and consensus-based terminology, taxonomy, and ontology for PLaTO. This is important as the field evolves and diversifies in its approaches, contents, and contexts over time and in different countries, cultures, and settings. Within this paper, we report the systematic and iterative processes undertaken to achieve this objective, which has built on the creation of the global PLaTO-Network (*PLaTO-Net*).

**Methods:**

This project comprised of four major methodological phases. First, a systematic scoping review was conducted to identify common terms and definitions used pertaining to PLaTO. Second, based on the results of the scoping review, a draft set of key terms, taxonomy, and ontology were developed, and shared with PLaTO members, who provided feedback via four rounds of consultation. Third, PLaTO terminology, taxonomy, and ontology were then finalized based on the feedback received from 50 international PLaTO member participants who responded to ≥ 3 rounds of the consultation survey and dialogue. Finally, efforts to share and disseminate project outcomes were made through different online platforms.

**Results:**

This paper presents the final definitions and taxonomy of 31 PLaTO terms along with the PLaTO-Net ontology model. The model incorporates other relevant concepts in recognition that all the aspects of the model are interrelated and interconnected. The final terminology, taxonomy, and ontology are intended to be applicable to, and relevant for, all people encompassing various identities (e.g., age, gender, culture, ethnicity, ability).

**Conclusions:**

This project contributes to advancing PLaTO-based research and facilitating intersectoral and interdisciplinary collaboration, with the long-term goal of fostering and strengthening PLaTO’s synergistic linkages with healthy living, environmental stewardship, climate action, and planetary health agendas. Notably, PLaTO terminology, taxonomy and ontology will continue to evolve, and *PLaTO-Net* is committed to advancing and periodically updating harmonized knowledge and understanding in the vast and interrelated areas of PLaTO.

**Supplementary Information:**

The online version contains supplementary material available at 10.1186/s12966-022-01294-0.

## Introduction

The literature on play, learn, and teach outdoors (referred to as “PLaTO” hereafter) is broad and extends across diverse disciplines. For the most part, PLaTO has been discussed from an early childhood pedagogical perspective [1–3] or a perspective of health and well-being across the lifespan [4–7]. The importance of outdoor play for health and well-being has received particular attention in recent years, as evidenced by the *2015 Position Statement on Active Outdoor Play* [8], which has been endorsed by more than 400 individuals and organizations globally [9]. Accumulating literature also suggests that spending time in natural outdoor environments is associated with reduced stress and behavioral patterns that are favorable to health (e.g., more physical activity and less sedentary behavior) [10–12]. Evidence is also building on the importance of natural environments in urban settings, including both green (e.g., parks, forests, fields, trees) and blue spaces (e.g., rivers, lakes, ponds, ocean), for human health and well-being, in addition to reducing air pollution, noise, and extreme temperatures in urban centers [10, 13–17].

The importance of PLaTO terms taking a natural path to grow, flourish, and adapt to different contexts, languages, and cultures is acknowledged. However, as diverse conversations on PLaTO emerge and expand, along with and their implications to sustainability and human health, so does the confusion. Specifically, there is substantial ambiguity in the definitions, purposes, positioning, processes, methods, and outcomes of PLaTO terms across different contexts and cultures [18–20]. This ambiguity has been discussed and partially addressed in the literature [21–26]; however, as a collective scholarship, it is largely unclear how PLaTO terms are defined (i.e., terminology) and categorized (i.e., taxonomy), and how the categories are related or conceptualized (i.e., ontology). This confusion, and occasionally contradiction, makes it challenging to create a harmonized dialogue across different academic fields and cultures, and thus limit the cohesiveness of, and potential and credibility for, the PLaTO sector. Lack of consistent and coherent communication from the PLaTO sector to other sectors and disciplines may be a barrier in building a strong evidence base for important policy-making and providing opportunity for cross-disciplinary support and partnership on global issues [27, 28]. For example, PLaTO has clear synergistic linkages with healthy living across the lifespan, environmental stewardship, climate action, and planetary health agendas, yet such synergies remain largely underexplored and underexploited, perhaps due to a lack of cohesiveness or clarity of message. PLaTO constantly evolves and diversifies in its approaches, contents, and contexts over time and across different countries, cultures, and settings. What is needed, therefore, is to develop and adopt consistent terminology, taxonomy, and ontology for PLaTO to enhance communication between and within the sector.

Accordingly, in March 2018, several experts in the field of outdoor education and play gathered at the *International Udeskole Conference* held in Fredensborg, Denmark and identified the following research priorities: 1) develop a conceptual framework that encompasses the broad area of PLaTO research and practice; and 2) reach consensus on terminology, taxonomy, and ontology on PLaTO terms. Shortly after the conference, *PLaTO-Net (Play, Learn, and Teach Outdoors Network)*, a global network that brings interested leaders, activists, researchers, educators, stakeholders, and policymakers together to promote and advance outdoor play and education, was established (https://www.outdoorplaycanada.ca/plato-net/). The overarching goal of this global network, *PLaTO-Net,* is to demonstrate leadership in creating a world where everyone has equal opportunity and access to play and learning in high quality natural environments on a regular basis.

The aim of this project was to develop harmonized, consensus-based terminology, taxonomy, and ontology for the PLaTO sector by leveraging the expanding *PLaTO-Net* membership (459 members from 53 countries as of August 31, 2021) and building upon previous efforts to identify and define common terms. These previous efforts included, for example, the outdoor play glossary of terms drafted by *Outdoor Play Canada* [29], as well as other work focusing on aspects of either outdoor play [8, 22, 30], outdoor education [18, 31–34], or the environment [35]. The specific objective of this paper was to report on the process to achieve this aim, and the outcomes.

## Methods

**Fig. 1:**
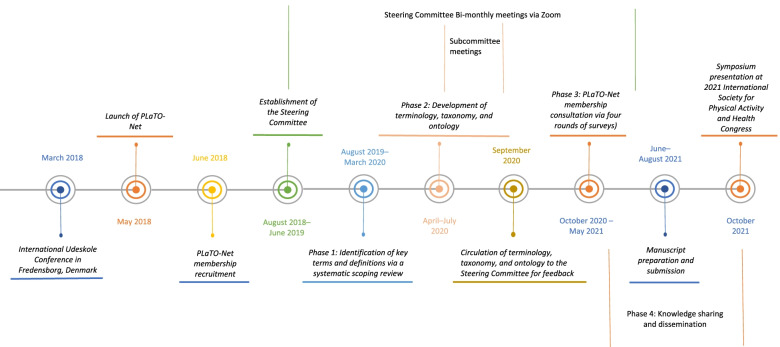
Play, Learn, and Teach Outdoors Network (*PLaTO-Net*) Terminology, Taxonomy, and Ontology Consensus Project steps and timeline

The project followed a series of processes, replicating the process of an earlier international terminology consensus project led by the corresponding author (MST) [36]. A summary timeline of these processes is provided in Fig. 1. Briefly, the overall effort included seven sequential steps: 1) launching *PLaTO-Net* via an online platform (www.outdoorplaycanada.ca/plato-net) and recruiting global *PLaTO-Net* members; 2) establishing an internationally representative Steering Committee for the project; 3) identifying key terms and definitions via a systematic scoping review; 4) analyzing data and developing draft terminology, taxonomy, and ontology; 5) finalizing the PLaTO terminology, taxonomy, and ontology based on *PLaTO-Net* members’ feedback via four rounds of consultation surveys; 6) preparing the manuscript with approval from all participants; and, 7) translating and disseminating our findings through publication, presentation, and online platforms.

The *PLaTO-Net* online platform was created to identify and gather thought-leaders interested in research and practice related to PLaTO and to facilitate international, intersectoral, and interdisciplinary exchanges of knowledge. The PLaTO-Net Steering Committee was established to guide the methodological phases and content development of the project. To establish the Steering Committee, the initial project leaders (EL, MG, MST) created a list of experts in the fields of outdoor play and outdoor education, with an emphasis on international and diverse community representation. Email invitations were then sent to recruit Steering Committee members. The four major methodological phases that the Steering Committee guided, included: 1) systematic scoping review; 2) terminology, taxonomy, and ontology development; 3) international membership consultation; and 4) knowledge sharing and dissemination.

### Phase 1: systematic scoping review

A systematic scoping review of relevant terms and/or definitions in the PLaTO literature was conducted between August 2019 and March 2020, following the methodology extended by Peters and colleagues (2015) [37] and the Preferred Reporting Items for Systematic reviews and Meta-Analysis extension for Scoping Review (PRISMA-ScR) guidelines [38] (Checklist is provided in Additional File 1). The goal of the scoping review was to develop a comprehensive list of key terms and definitions related to PLaTO.

#### Study inclusion criteria

For this scoping review, the authors reviewed studies consisting of terms and/or definitions related to PLaTO in English published up to September 2019. No exclusions based on study design (i.e., previous literature reviews were included) or specific characteristics of study participants were made (i.e., children living with disability, diagnosed with chronic conditions, and from diverse backgrounds were all included). Articles were included if the mean/median age of participants was between 2 and 18 years or age-stratified results in this age range were provided. Although the overall consensus project did not specify the age group of interest a priori, the literature search for this scoping review was limited to children and youth. This was based on a collective decision made by the members of the Steering Committee on the basis that: 1) there was a vast amount of literature available in the field of PLaTO due to its broad scope and; 2) the majority of the relevant evidence focused on children and youth. All included articles must have mentioned PLaTO and defined or described terms or models related to PLaTO. Articles were excluded if they involved organized sports played outside that were competitive in nature (e.g., league soccer or baseball), regardless of age.

#### Information sources and search

The search strategy was developed by research staff (LC) and reviewed by a research librarian. The electronic search was executed on September 6, 2019 on Ovid MEDLINE and ERIC. The two search concepts for the strategy included ‘outdoor playing/learning/teaching’ and ‘children/youth’. Key terms included outdoor education, teaching outside, education outside the classroom, outdoor play, forest school, green classroom, and nature play, amongst others. Full search strategies for each database are provided in Additional File 2, Supplementary Table 1. Additional citations were suggested by members of the *PLaTO-Net* Steering Committee (list available in Additional File 2, Supplementary Table 2).

#### Evidence screening and data extraction

All articles that included playing, learning, or teaching in the outdoors and defined or described outdoor play, learning, or teaching term(s) or provided a conceptual model(s) were imported into Covidence—a web-based software for screening references [39]. Screening was conducted independently by a team of nine screeners (AB, GB, LC, PE, LM, SH, EL, SAM, KR). A training session was held in advance of screening to familiarize the screeners with the project, explain the inclusion/exclusion criteria and answer any questions. After the training session, all eight screeners commenced Level 1 screening (titles and abstracts) by reviewing the same ten articles. The group then met to discuss any challenges and provide clarification, as necessary. Regular meetings took place to discuss screening progress and ensure the team members were consistently applying eligibility criteria. The same process was followed for Level 2 screening (full-text articles).

Data were extracted independently by the eight individuals who participated in the screening with support from one research staff (PP). The data extraction form was developed a priori in Excel and piloted and revised before use. Variables extracted from the relevant articles included author(s); publication year; title; journal name; country; age group of interest; participant sex; key terms related to playing, teaching and learning in the outdoors; definitions/descriptions corresponding to the key terms; source of definition/description (if available); and whether a conceptual model was provided (yes/no, describe if yes). Each article was screened (Level 1 and Level 2) and the data were extracted by a single screener/extractor. Since the list of key terms and definitions would later be reviewed and refined by the Steering Committee and interested *PLaTO-Net* members, it was not essential that the included articles be completely exhaustive. Thus, errors that may have been introduced by taking the single screening approach was considered nonthreatening to the validity of the data generated.

#### Evidence synthesis

A project leader (EL) organized the extracted data by terms with definitions and terms without definitions. Terms with definitions were from the studies that introduced relevant terms and definitions or descriptions for a given term. Taking a content analysis approach, terms were then organized into the following 10 categories based on their relevance that emerged during the data extraction process: play, outdoor play/time/playtime, education/learning/school, curriculum/pedagogy/program, therapy, activity/adventure/expedition/recreation, environment/location/space, approach/model/theory, outcome/component, and element/feature. Terms without definitions were from studies that included terms relevant to outdoor play, learning, or teaching but provided no definitions or descriptions for the terms. These were compiled into an alphabetical list and cross-checked against all defined terms; terms not defined in any of the identified articles were flagged and later defined by the project leaders (EL, LDL, MST) in consultation with online dictionaries and the Steering Committee. The summary of these data was done by an undergraduate research intern (LL).

### Phase 2: terminology, taxonomy, and ontology development

Based on synthesized evidence from the scoping review, developing terminology, taxonomy, and ontology was done via multiple iterative processes based on independent and joint work of two Sub-Committees (A and B). Sub-committees were recruited on a voluntary basis from the Steering Committee members and their trainees while ensuring that there was representation from each of the varying areas of research: Sub-Committee A consisted of seven experts and Sub-Committee B consisted of five experts, all representing unique areas of expertise (Additional File 2, Supplementary Table 3). The outcome of the Sub-Committees’ work was presented to the Steering Committee for feedback and consolidation.

Sub-Committee A was formed to determine PLaTO terms and definitions that were most relevant and representative of sub-terms/definitions and to develop a draft diagram (e.g., framings and categorizations of terms and definitions). The goal of Sub-Committee A was to develop a draft ontology model that best defined the relationships among elements of PLaTO. Sub-Committee B focused on refining and sorting through the identified terms and definitions. The purpose of Sub-Committee B was to select key terms (i.e., terminology) and classify key terms and definitions from the list (i.e., taxonomy) created based on the scoping review.

### Phase 3: international membership consultation

Based on the outcomes of Phases 1 and 2, Phase 3 involved four rounds of membership consultation surveys between October 2020 and May 2021. Surveys were constructed iteratively with Round 1 focusing on evaluating the likability of the draft ontology model and logo (i.e., simplified version of the model), and the suitability of relevant terms in five key areas (i.e., outdoors, outdoor play, outdoor learning, outdoor teaching/education, and outdoor schools). Survey Round 2 was focused on further evaluation of the likability of the revised ontology model and logo, as well as the suitability of the taxonomy of key terms. Survey Round 3 was focused on obtaining consensus on the ontology model, logo, and terminology to be included in the final work. Survey Round 4 primarily focused on obtaining consensus on taxonomy and terminology with definitions. The project leaders (EL, LDL, MST) and a graphic designer (LS) were responsible for drafting the content for each survey (all four rounds of surveys are available in Additional File 3).

Within each survey we asked participants to indicate whether the draft models and proposed terms and definitions were clear and to note the extent to which they liked or agreed with each item. For draft models (Rounds 1–2), a five-point Likert scale ranging between “strongly like” to “strongly dislike” was used. For terms and definitions, either binary options (“yes” or “no”) for the inclusion of an item or a five-point Likert scale (ranging between “strongly agree” to “strongly disagree”) were used. Consensus was achieved when ≥ 75% (or a mean score of at least 4 out of 5) of the survey participants reacted positively to each item [36]. Agreement was considered as high, moderate, or borderline strengths for ≥ 90%, 80–99%, and 75–79%, respectively. Feedback from all survey participants for each survey was summarized by an undergraduate research assistant (LL) and discussed amongst project leaders (EL, LDL, MST) for consolidation. The final revisions to the ontology model and terms and definitions were reviewed by the Steering Committee. The manuscript, which included the final ontology model, taxonomy, terms and definitions, and all relevant additional files, was sent to survey participants who participated in at least three rounds of the consultation surveys (*n* = 50, excluding the members of the Steering Committee) for their review, feedback, and final approval.

### Phase 4: knowledge sharing and dissemination

Phase 4 focused on facilitating exchanges of knowledge related to PLaTO and disseminating the outcomes of this project through different platforms. Throughout the process of the consensus project, knowledge sharing was primarily done online through the *PLaTO-Net* online platform and social media (Twitter). Under the supervision of two members of the Steering Committee (EL, SAM), two research assistants (LL, LR) collaborated on developing detailed social media timelines and content related to PLaTO and posted them on Twitter. Content was developed under four main themes: outdoor play, outdoor learning, outdoor teaching, and ecosystems/nature/environment. Types of content included blog posts/news articles, videos, notices about holidays/international days and weeks, features/recent publications, other forms of resources (e.g., non-peer reviewed articles, government reports), and *PLaTO-Net* announcements. All tweets were pre-recorded on Excel in advance by the research assistants and approved by the supervisors in a timely manner. Given that Twitter, by design, is made to accommodate short and frequent updates, approved tweets were posted three times a week. Tweet activities were evaluated based on two indicators: total impressions and engagement rate. The number of total impressions––the total number of times a tweet was loaded in a Twitter feed—was used to indicate the reach. The number of total engagements—the number of times people engaged with a tweet by commenting on it, liking it, retweeting it, or clicking on it for any reason—was used to indicate actual engagement with the posted material. The engagement rate, provided by Twitter, was also reported based on the following formula: total engagements/total impressions $\times$ 100. Dissemination of the project outcome was planned via two channels: a symposium at the 8^th^ International Society of Physical Activity and Health Congress and publication in an internationally renowned journal with high impact. Details of the knowledge dissemination plans are described in the Results section below.

## Results

### Creation of *PLaTO-Net* and Membership Recruitment

In May 2018, the first and corresponding authors (EL, MST) co-created *PLaTO-Net* and launched its webpage (https://www.outdoorplaycanada.ca/plato-net/) to respond to a collective call among participants at the *International Udeskole Conference* in March 2018 for a method to facilitate dialogue on achieving consensus on PLaTO terms and definitions. Currently, the webpage has two sections, the first section is for member recruitment and registration (free of charge) and the other section describes the *PLaTO-Net* consensus project and includes list of *PLaTO-Net* Steering Committee members. The purpose of recruiting members was to gather thought-leaders interested in research and practice related to PLaTO. The member recruitment section also outlines *PLaTO-Net*’s vision, mission, and membership responsibilities, which include working on various projects to share evidence and resources, harmonize terminology, and collaborate on research and advocacy efforts to advance PLaTO. As of August 31, 2021, *PLaTO-Net* had 459 members from 53 countries. In November 2020, following the launch of the webpage, the *PLaTO-Net* Twitter account (https://twitter.com/PLaTO_Net) was launched to broaden and diversify *PLaTO-Net’s* reach and communications. The *PLATO-Net* Twitter handle @PLaTO_Net started posting in November 2020 and has 293 followers (as of September 22, 2021). A detailed description of Twitter activities is available in the *Phase 4: Knowledge Sharing and Dissemination* section below.

### Establishment of the Steering Committee

A total of 18 leaders from nine countries were initially invited via email to join the Steering Committee. The invitation email included the goal and main activities of the project and the commitment required from members. Out of a total of 18 invitees, 13 from seven countries accepted the invitation. Nine additional international members from seven countries were invited based on suggestions from the existing members of the Steering Committee, making up a total of 22 members from diverse countries around the world. Two research staff (MG and LC) provided substantial support in identifying and recruiting the Steering Committee. A list of the final Steering Committee members can be found in Additional File 2, Supplementary Table 2.

### Phase 1: Systematic Scoping Review

**Fig. 2:**
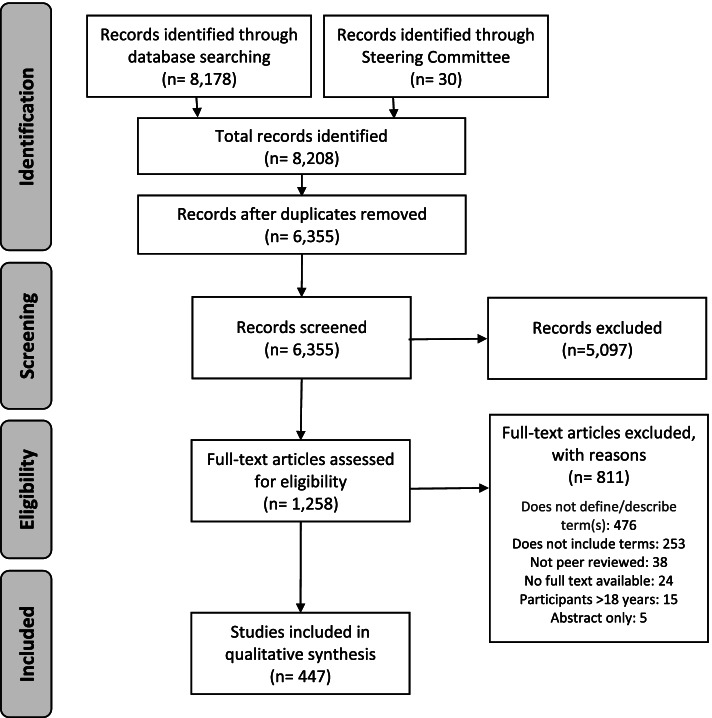
Summary of evidence identification and study selection. *Note:* The full list of 447 references can be found in Additional File 2

A summary of evidence identification and study selection is illustrated in Fig. 2. Briefly, the electronic search and suggestions from the Steering Committee yielded 8,208 records. After duplicates were removed, a total of 6,355 articles were eligible for Level 1 screening. Of those, 1,258 articles were included in Level 2 screening and a total of 447 articles met the eligibility criteria to be included in the review (full list of 447 articles is available in Additional File 4).

Relevant terms with definitions were originally organized based on 10 key categories (Additional File 5). A total of 428 terms and 984 definitions were identified from 441 studies, representing 35 countries (of these, 26 articles were international, and three articles did not specify the geographical location). The terms were not necessarily mutually exclusive because several terms were a combination of two or three distinct words that were already captured. For instance, “play” (*n* = 29 studies with definitions), “active play” (*n* = 13 studies with definitions), or “active free play” (*n* = 2 studies with definitions) were all treated as unique terms and recorded separately.

A total of 181 terms without definitions or descriptions were identified from 143 unique studies (Additional File 5), representing 18 countries (of these, 24 articles were international work and four articles did not specify the geographical location of their investigation). The terms that were used in most studies without definitions included “active play” (*n* = 8 studies), “free play” (*n* = 14 studies), “outdoor education” (*n* = 15 studies), “outdoor learning” (*n* = 17 studies), and “outdoor play” (*n* = 55 studies). Of the 181 terms without definitions provided in the searched studies, 98 non-mutually exclusive terms had no definitions or descriptions in any studies included in this review. For instance, “hands-on experiences” and “hands-on learning” were two of the 98 non-mutually exclusive terms that were never explicitly defined or described by study authors.

### Phase 2: Terminology, Taxonomy, and Ontology Development

Three independent meetings of Sub-committee A and one joint meeting with Sub-committee B were held via an online meeting platform between April and May of 2020, during which five possible ontology models were developed based on the synthesized evidence from the scoping review. From June to August 2020, the five ontology models were merged into one holistic, over-arching ontology model. The initial draft of the model was circulated to the Sub-committees A and B in early September 2020 for their feedback and to ensure the model captured the key aspects from the five ontology models. This model was further circulated to the Steering Committee in mid-September 2020 for feedback and to fill any expertise gaps.

The goal for the ontology model was to capture all aspects of activities that occur in outdoor spaces, for all ages, and represent their relationship with one another. In addition, while highlighting the key aspects of PLaTO, other relevant and related concepts (e.g., work, commute, recreate) were incorporated into the model as a way of acknowledging the broad range of activities that occur outdoors and the way they are interrelated with PLaTO. We also sought to create a model that was as inclusive as possible for all ages, genders, cultures, and abilities. The initial model reflected the general concept of ecological modelling, where multiple factors in different domains are positioned in layers, emphasizing the importance of interrelatedness, but also introducing patterns of hierarchy.

**Fig. 3:**
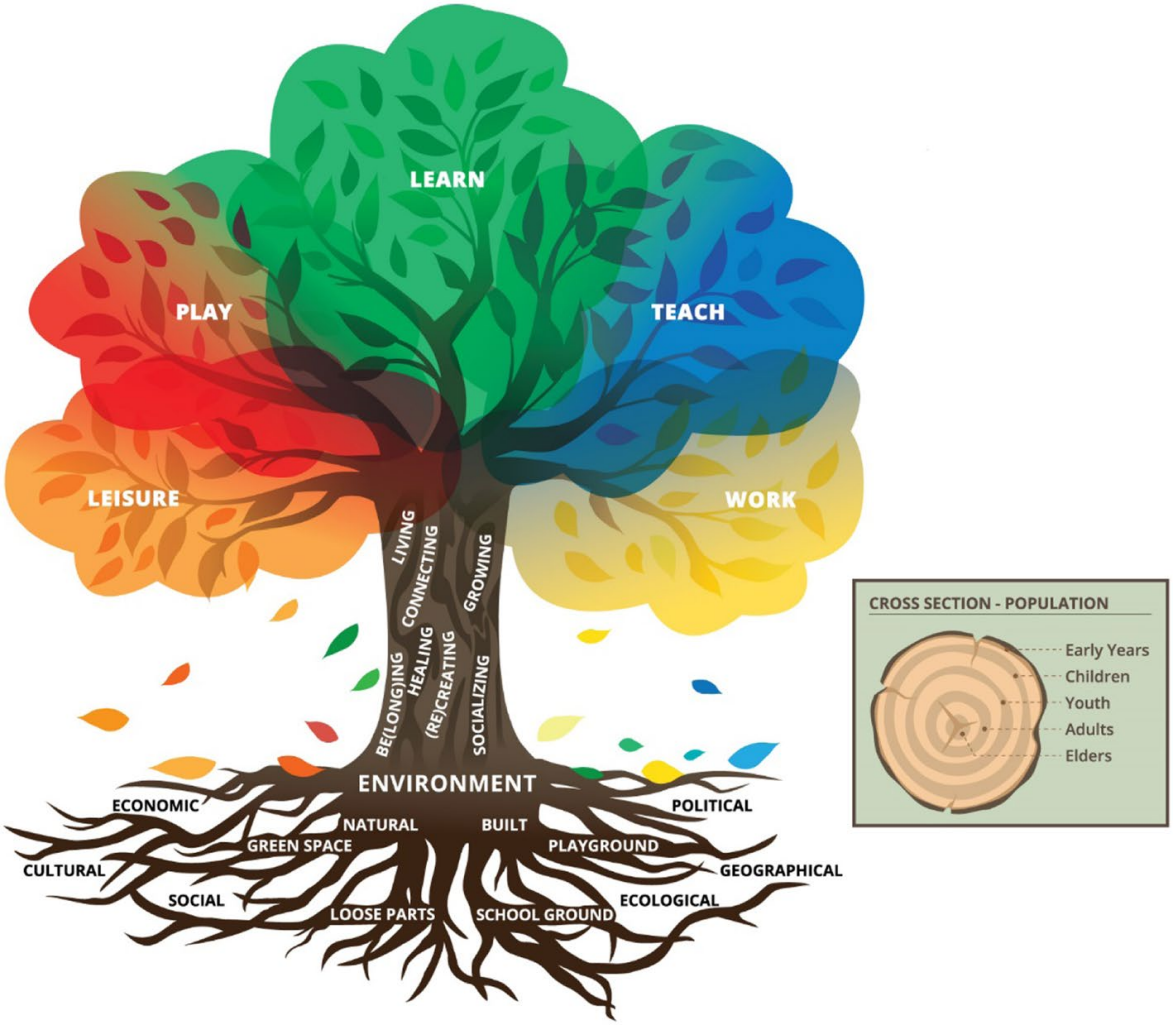
Play, Learn, and Teach Outdoors Network (*PLaTO-Net*) ontology model. *Note*: At the bottom of the model, surrounding the tree roots in the earth, are the terms *economic*, *cultural*, *social*, *ecological*, *geographical*, and *political* which denote some of the main influences on/consequences of outdoor activities. Along the roots are examples of outdoor settings, starting at the top of the roots with ‘environment,’ branching out to the terms *natural* and *built*, leading to *green space* and *loose parts,* and *school ground* and *playground*, respectively. The roots overlap with each other to indicate inter-relationships between all terms in the earth and roots. Along the bark of the tree trunk are examples of purposes/outcomes that can be achieved while engaging in different activities in the outdoors (e.g., *living*, *connecting, growing, be[long]ing, healing, [re]creating,* and *socializing*), where the bark again overlaps with the different terms to indicate the connectivity of and fluidity between these terms. The trunk supports the leafy canopy where we suggest there are five overlapping main types of activities that can be performed outdoors. These activities span across a range of colors that blend into and overlap with each other, with *leisure* in orange, leading into *play* in red, *learn* in green, *teach* in purple, and *work* in yellow (e.g., some leisure activities involve play, a lot of play can be informal learning, working outdoors can involve play, or teaching and so on). Some leaves have fallen back down to the soil to indicate the cyclical and interconnected relationship between all elements. There is also a box to the right of the tree with a cross-section indicating the rings of the tree, with the labels *early years, children, youth, adults,* and *elders* in concentric rings moving from the outermost ring to the center, just as the youngest tree rings are at the edge and the oldest at the center, to highlight the applicability of the model to all humans across the lifespan

**Fig. 4:**
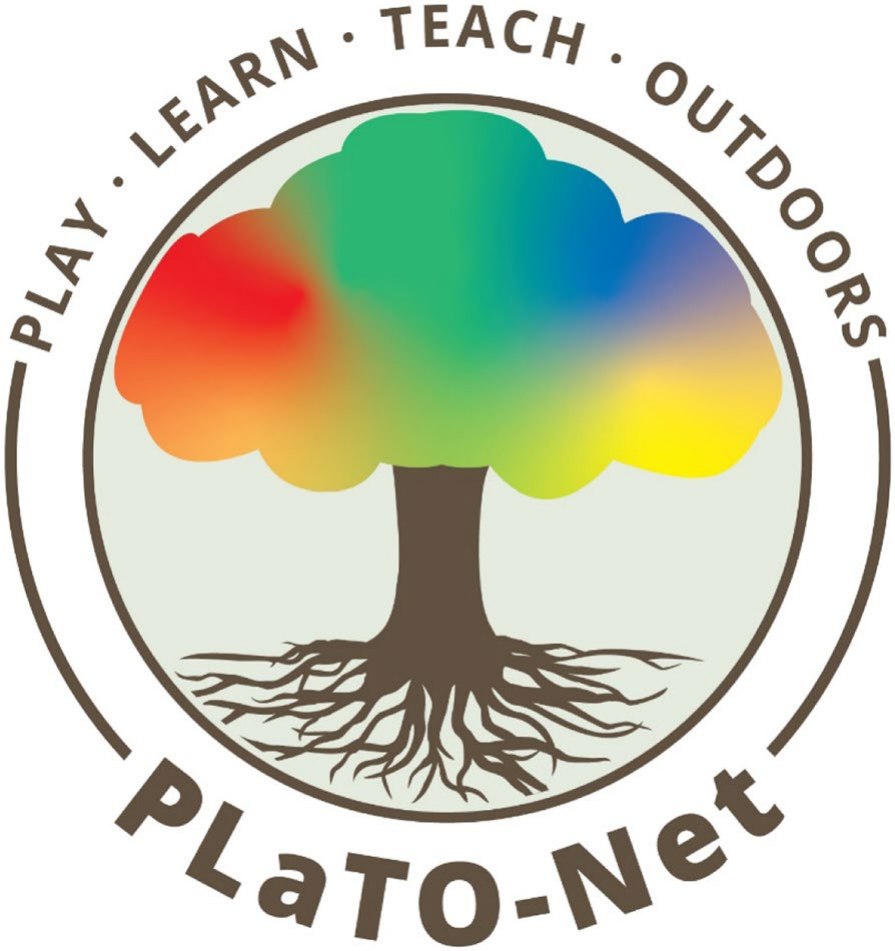
Play, Learn, and Teach Outdoors-Network (*PLaTO-Net*) logo. *Note*: The logo still has all elements from the PLaTO ontology model; however, the words on the logo only highlight the focus of *PLaTO-Net*

The initial model (see Additional File 4: *PLaTO-Net* Global Harmonization Project – Membership Consultation Questionnaire page 3/12) was circular in shape with five concentric rings. In this model, the center ring denoted the context, “who, where, when, why, and how.” The second ring indicated the dynamicism of these contexts using the following action verbs: playing, learning, teaching, working, recreating, being/connecting, and socializing. The third ring indicated general/thematic activities that are performed outdoors using the following terms: education, work, spiritual pursuits, and leisure. The fourth ring indicated the main setting of interest “outdoors.” Lastly, the outermost ring indicated influences on, and consequences of, outdoor activities using the following terms: social, cultural, political, geographical, and climatic. The five rings were interpreted as circulating around each other to indicate the dynamicity and fluidity within and between rings. Edges between each ring were also purposefully blurred to highlight the interconnectivity of terms and acknowledge that there may be other terms/concepts not included in the model. However, given a lack of consensus on this initial model within the Round 1 consultation survey (see Phase 3: Global Membership Consultation), the project leaders opted to hire a graphic designer to develop a new model and logo that better captured all aspects of activities that occur in outdoor spaces and their relation to one other. For a full description of the final ontology model and logo, see Figs. 3 and 4, respectively.

Four independent meetings of Sub-committee B and one joint meeting with Sub-committees A and B were held in Phase 2 to refine the 428 terms found in the scoping review in Phase 1. Members consolidated congruent terms and developed agreed upon definitions for these. Through discussions on relevance, accuracy, and redundancy, 36 key terms and definitions were selected for further consideration. Of those, nine terms were considered relevant to outdoor play (i.e., outdoor play as its own separate term, free play, land-based play, nature play, risky play, free-flow play, outdoor recess/break time, active play, social play), eight terms were relevant to outdoor learning (i.e., outdoor learning as its own separate term, nature-based learning, land-based learning, place-based learning, environmental learning, informal learning, learning–or education– outside the classroom, service learning), six terms were related to outdoor teaching/education (i.e., outdoor teaching, outdoor education, nature-based education, place-based education, adventure-based education, environmental education), and 10 terms were related to the outdoors as a setting (i.e., home range, nature, natural environment, built environment, green schoolyards, outdoor play space, nature play spaces, playground, nature playground, garden). Additionally, three school-related terms (i.e., outdoor school, forest school, nature school) were also included during Phase 2.

### Phase 3: International Membership Consultation

Phase 3 comprised of four rounds (Rounds 1–4) of *PLaTO-Net* membership consultation surveys using the Delphi approach [40]. All four surveys can be found in Additional File 4. The initial invitation to participate in the membership survey included 395 *PLaTO-Net* members. Based on the draft of the initial ontology model and key terms identified during Phases 1 and 2, the Round 1 consultation survey went out to the *PLaTO*-*Net* membership on October 16, 2020, and the follow-up consultation surveys (Rounds 2–4) went out on January 11, March 5, and May 26, 2021. A total of 132 members completed the first survey, and 85, 62, and 70 members participated in the follow-up surveys, respectively. Among those, 52 members participated in all four surveys (including the members of the Steering Committee), eight participated in three surveys, 23 participated in two surveys, and 48 members participated in one survey. Overall, survey participants represented 36 countries with varying cultural/racial/ethnic backgrounds, working in relevant fields as researchers, educators, practitioners, and government employees, with the majority (68.2%) having ≥ 10 years of experience in the relevant areas. Of the 132 members who completed the first survey, 66.7% indicated their gender as female or woman while 33.3% indicated their gender as male or man. None of the participants self-identified as non-binary or other gender. Of the 84 who indicated their disability status, 9.7% identified as living with disability. Out of 52 members who participated in ≥ 3 surveys, 48 members have agreed to be listed as co-author and provided critical feedback on the manuscript (see Additional File 6 for information about survey participants).

Table 1 presents the results of each round of the surveys. Likability of the model with the ontology model and logo increased from Round 1 (model: 67.0%; logo: 26.5%) to Round 2 (model: 81.2%; logo: 83.5%) and levels of agreement with the final model and logo was the highest in Round 3 (i.e., 93.6% and 95.2% respectively, see Table 1), indicating strong support with iterative modifications made during Rounds 1 and 2. This manuscript only presents the final versions of the ontology model (Fig. 3) and logo (Fig. 4). Level of agreement with the structure of the key terms was 80% in survey Round 2, achieving international consensus on the taxonomy.

Consensus on terminology was achieved through three rounds of surveys (Rounds 1, 3, and 4). A total of 36 key terms included in Round 1 was based on the results of the scoping review (*n* = 33), as well as suggestions from the Steering Committee to include school-related terms (*n* = 3). Of these, eight terms were removed from Survey Round 3 due to low (< 75%) likability (indicated in red in Round 1, Table 1) and six additional terms were removed despite achieving ≥ 75% of likability (indicated in green and “xx” in Round 1, Table 1) as they were later identified as co-hyponyms (i.e., subordinate terms of the same superordinate term) of other terms. Five terms achieved < 75% likability but were included in Survey Round 3 based on a decision made by the members of the Steering Committee to further explore the suitability of those terms given their relevance to the project (indicated in yellow and “vv” in Round 1, Table 1). In addition, 27 new terms were suggested for further consideration by survey participants (indicated as “v” in Round 1, Table 1).

Survey Round 3 evaluated the suitability of 49 terms forwarded from Round 1. Of these, ≥ 75% of survey participants agreed with the inclusion of 32 terms (≥ 90% of agreement for 12 terms; 80–89% of agreement for 13 terms; 75–79% of agreement for 7 items). Additionally, four terms with < 75% agreement (i.e., *loose parts*, *social play*, *outdoor time*, *nature-based recreation*; indicated in blue in Round 4, Table 1) were re-evaluated by the members of the Steering Committee based on qualitative feedback and the leadership group made the unanimous decision to include these terms in survey Round 4 for further consideration. Of the 13 terms removed during Round 3, 11 terms were originally not part of Round 1 but were suggested by survey participants while the other two terms—*home range* and *free-flow play*––received low likability during Round 1 but were forwarded to Round 3 based on expert suggestions. Qualitative feedback indicated the lack of agreement (< 75%) on 13 terms in Round 3 (indicated in red in Round 3, Table 1) were mainly due to a lack of direct relevance to PLaTO (e.g., *adventure therapy*, *wilderness therapy/treatment*), overlap with other included terms or being co-hyponyms (e.g., *nature-based preschool*, *outdoor school*), or being too culturally specific (e.g., *shirin yoku*, *friluftsliv*).

Survey Round 4 aimed to achieve consensus on working definitions of 36 terms forwarded from Round 3 and four additional terms deemed to be root terms (i.e., play, teaching, learning, education). Of those, proposed definitions for 17 terms achieved a high level of agreement (≥ 90%), 12 terms achieved a moderate level of agreement (80–89%), and two terms achieved a borderline level of agreement (75–79%), while agreement with the proposed definition for three terms (i.e., *nature*, *outdoor learning*, *outdoor education*) was < 75% (indicated in red in Round 4, Table 1). All terms and definitions were reconsidered based on both quantitative and qualitative feedback when finalizing the included terminology. For survey Round 4 alone, a total of 238 qualitative comments were provided. Among those, 82 comments were relevant to root terms, 53 to outdoor play sub-terms, 48 to outdoor education sub-terms, and seven additional terms were relevant to wording or the inclusion of other types/languages.

Several modifications were made to the list of 40 terms and definitions by the project leaders to incorporate both quantitative and qualitative feedback from Survey Round 4 participants. Most edits were to improve clarity and inclusivity. For instance, *outdoors* was originally defined as: “any open-air, wild, natural, or human-made space which may have a temporary or fixed cover (e.g., awning or roof).” However, a few survey participants felt that reference to ‘a temporary or fixed cover’ confused the term while others indicated that in countries with many extreme heat days, many outdoor spaces have a fixed cover used by humans for protection. In response, the project leaders included reference to ‘a temporary or fixed cover’ as a nuance but not as part of the main definition (Table 2). Also, based on several comments that there was lack of consistency among definitions of root terms and sub-terms for play, we revised the definition for root terms, so they were more encompassing of all play sub-terms. We also simplified the definitions of sub-terms to better reflect hyponymy. For instance, in the revised version, all play sub-terms started with the phrase “a form of play….” However, this change was not applied to *learning*, *teaching*, and *education* and their sub-terms, given the variability across the terms included in these categories. In addition, the project leaders decided to remove 13 sub-terms based on survey feedback that some terms were too broad (e.g., garden, nature) or too obvious (e.g., outdoor time, outdoor activity), and thus out of scope for this project.

**Table 1:**
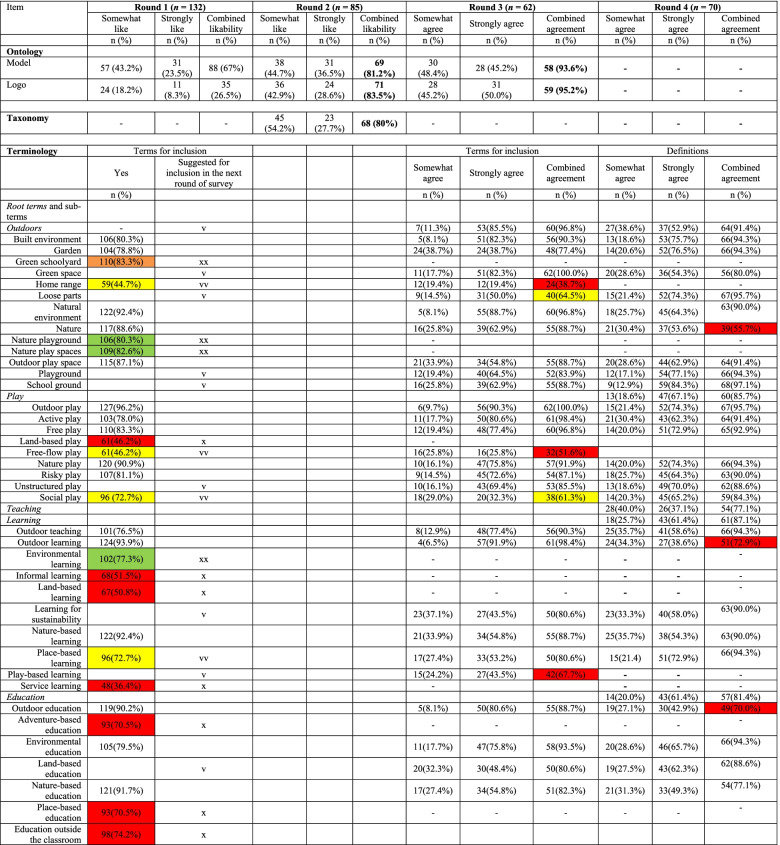

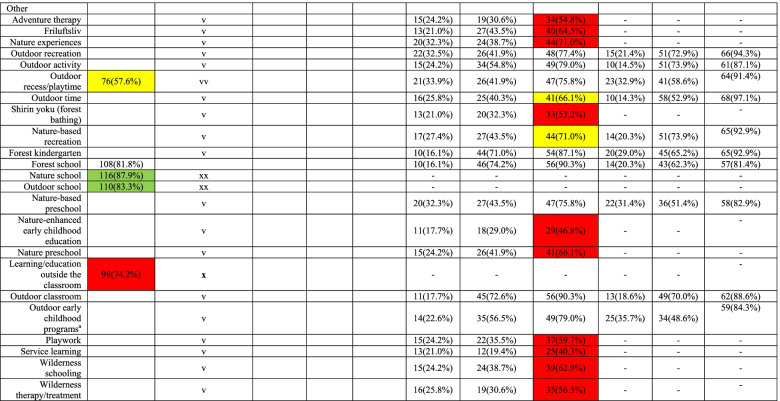
Survey results for agreement with the Play, Learn, and Teach Outdoors (PLaTO) terminology, taxonomy, and ontology

v: New terms suggested for further consideration by survey participants; vv (also in yellow): terms suggested for inclusion by the members of the Steering Committee despite achieving < 75% likability; x (also in red in Round 1 and Round 3): terms removed as they were suggested for exclusion by survey participants (< 75% likability/agreement); xx (also in green): terms removed despite achieving ≥ 75% of likability as they were later identified as co-hyponyms

^a^ Outdoor early childhood programs included outdoor kindergarten, outdoor preschool

**Table 2:** Terminology and taxonomy of the Play, Learn, and Teach Outdoors—Network (*PLaTO-Net*)

Root terms^a^	PlaTO-terms	Sub-terms	Proposed definition	Synonym/co-hyponym
Outdoors			Any open-air, wild, natural, or human-made space	
			*Nuances*: The space may include a temporary or fixed cover (e.g., awning or roof) but maintain exposure to ambient environmental conditions	
		Built environment	Human-constructed physical surroundings (e.g., structures, features, facilities) in which people live, learn, work, travel, and play	
		Green space	Any vegetated land, an area of grass or trees that may also contain bodies of water (e.g., pond, creek), in an urban environment	
			*Nuances*: The space may either set apart for recreational or aesthetic purposes or wasteland areas that have been colonized by nature in an otherwise urban environment	
		Loose parts	Natural or manufactured materials with no specific set of directions that can be used alone or combined with other materials, moved, carried, combined, redesigned, lined up, and taken apart and put back together in multiple ways and used for play	
		Playground	A piece of land usually equipped with facilities and/or equipment that is used for outdoor play and recreation	
		School ground	Proprietary outdoor area on the land of educational institution buildings	Synonym: School yard
		Natural environment	Non-built surroundings and conditions in nature in which living and non-living things co-exist	Synonym: Nature
		Garden	Planted, developed, or cultivated land used to grow vegetables, fruit, herbs, flowers, and other living plants and organisms	
		Outdoor play space	Any outdoor area where people can play	
Play			Voluntary engagement in activity that is fun and/or rewarding and usually driven by intrinsic motivation	
			*Nuances*: Not all play is self-directed and intrinsically motivated	
	Outdoor play		A form of play^a^ that takes place outdoors^a^	Co-hyponym: Outdoor recreation
		Active play	A form of play^a^ that involves physical activity of any intensity	
		Free play	A form of play^a^ that is unstructured and self-directed	Synonym: Unstructured play
		Nature play	A form of play^a^ that takes place in a natural environment and/or involves interaction with natural elements and features (e.g., water, mud, rocks, hills, forests, and natural loose parts, such as sticks, pinecones, leaves, and grass)	Co-hyponym: Nature-based recreation
		Risky play	A form of play^a^ that is thrilling and exciting, which involves uncertainty, unpredictability, and varying degrees of risk-taking	
		Social play	A form of play that involves interacting with others	
Learning			The development of knowledge, skills, values, morals, beliefs, and habits	
	Outdoor learning		Learning^a^ that takes place outdoors^a^	
Teaching			The process of facilitation of learning	
	Outdoor teaching		Teaching^a^ that takes place outdoors^a^	
Education			The process of learning^a^ and teaching^a^	
	Outdoor education		Education^a^ that takes place outdoors^a^	
		Environmental education	A form of education^a^ aimed at increasing knowledge, awareness, and appreciation of the environment	
		Forest schools	An educational^a^ approach that includes regular and repeated access to natural space and participant-directed, emergent, and place-based learning	Co-hyponym: Forest kindergartens, forest preschools, forest programs
		Outdoor classroom	A shared space of learning^a^ and teaching^a^ in the school context that is entirely outdoors	
			*Nuances*: Unlike outdoor education, an outdoor classroom takes regular pedagogy and curriculum outdoors in the school context	
		Place-based learning	Learning^a^ that considers the importance of connecting learners with their community by anchoring pedagogy within the context of the locally natural, cultural, and social ecosystems	Co-hyponym: Place-based education
			*Nuances*: The learning focuses on a specific physical space which may or may not involve the natural environment	
		Land-based education	An approach to education^a^ that recognizes a deep connection and relationship of reciprocity between people and the land	Co-hyponym: Land-based learning
			*Nuances*: This is specific to the North American context based on Indigenous epistemology of which the land is being understood beyond the physical sense and as with spiritual, emotional, and intellectual sense	
		Nature-based education	A form of teaching^a^ and learning^a^ situated in the context of outdoor natural settings	Co-hyponym: Nature-based learning, nature-based preschool
		Learning for sustainability	A cross-curricular approach to life and learning^a^ which enables learners, educators, schools, and their wider communities to build a socially just, ecologically sustainable, and equitable society	
**Other terms related to PLaTO**				
		Outdoor activity	Leisure, recreational, educational, occupational, and/or health-enhancing activity engaged in the outdoors	
		Outdoor time	Time spent outdoors	

^a^Refer to the definition of corresponding root term

The revised list consisting of 27 terms and their definitions was then circulated to the Steering Committee for final feedback. Included in the document sent to the Steering Committee was a list of 13 terms originally included in Survey Round 4, but that the project leaders felt should be removed, with justification for their removal from the consensus project included. Three of these terms were removed and four terms were re-introduced as synonyms. The Steering Committee suggested the following remaining six terms be removed: *garden*, *nature*, *outdoor recreation*, *nature-based recreation*, *outdoor activity*, and *learning for sustainability*. Yet, among these, *nature* was removed but re-introduced as a synonym for *natural environment. Outdoor recreation* and *nature-based recreation* were re-introduced as synonyms for *outdoor play* and *nature play*, respectively. The other three terms remained in the consensus project. The project leaders decided to include *garden* as it is an intentional green space frequently used for play or educational purposes (e.g., school garden), and thus would be helpful to include. *Outdoor activity* was kept because it is often conflated with other terms (e.g., *active outdoor play*) included in the consensus project, and therefore should be included to provide further clarification. *Learning for sustainability* also remained in the consensus project because of its increasing importance for climate action.

As the last step in achieving consensus on PLaTO terminology, taxonomy, and ontology as well as the logo, the research manuscript with all relevant attachments were circulated to those who completed at least three rounds of the consultation surveys for final review and sign-off via email. Participants were also asked to respond to the questions:

- Question 1. “Do you feel your feedback has been acknowledged and considered during the consensus process?” with the following response options: “Yes”, “No”, “Unsure”, and “Other”.
- Question 2. “Has your level of agreement changed since the completion of the last survey round?” with the following response options: “Increased”, “Decreased”, “No change”, and “Other”.
- Question 3. “Will you use the outcome of this project in your future work?” with the following response options: “Yes”, “No”, “Unsure”, and “Other”.

Of the 67 final co-authors, 97% answered “Yes” to Question 1, while 1.5% (*n* = 1) responded that they are “Unsure” and another 1.5% (*n* = 1) responded to “Other” with a note “I am not an expert”. For Question 2, 59.7% responded “Increased” and 40.3% responded “No change”, while no one responded “Decreased”. For Question 3, 95.5% responded that they will use the outcome of this project in their future work, while 4.5% responded “Unsure”. Non one responded to “No”. These survey results, combined with the level of agreement in consultation surveys, demonstrated that, overall, survey participants provided strong support for the final ontology, taxonomy, and terminology of PLaTO developed in this project.

#### Final terminology, taxonomy, and ontology

The final terminology and taxonomy of 31 terms are presented in Table 2. Among them, five terms (i.e., *outdoors*, *play*, *learning*, *teaching*, *education*) were root terms that serve as hypernyms (i.e., superordinate terms that have several subordinate terms that fall under them) for their respective sub-terms. Three PLaTO terms, namely, *outdoor play, outdoor learning,* and *outdoor teaching* as well as *outdoor education*, consisted of the main topics of this manuscript. The proposed definition of the hybrid term *outdoor play* was based on the definitions for *outdoors* and *play* (Table 2). Similarly, the proposed definitions for *outdoor learning, outdoor teaching*, and *outdoor education* were determined based on the definitions of their root terms (i.e., a combination of definitions for *outdoors* and *learning*). There are also 20 sub-terms included in Table 2. These terms are co-hyponyms with PLaTO terms, which can also be combined with PLaTO terms to create a variety of hybrid terms. For instance, *active play* is defined as a form of play that involves physical activity of any intensity. Combined with *outdoor play*, *active outdoor play* can be defined as a form of play that takes place outdoors which involves physical activity of any intensity (not included in Table 2). Though not part of the key aspects of this consensus project, *outdoor activity* and *outdoor time* and their definitions were also provided given these are used as overarching terms and key outcome variables in investigations relevant to PLaTO. In addition to definitions, we added nuances to better articulate subtle differences between PLaTO terms as well as synonyms and co-hyponyms identified in this project but not included as part of the defined PLaTO terms, to be as inclusive and useful as possible.

The PLaTO ontology model was developed to capture all aspects of activities, beyond PLaTO, that occur in the outdoors and their relation to one another. For the consensus project, our intention was to highlight the PLaTO terms, while incorporating other relevant concepts to recognize that all the aspects included in the model are interrelated and interconnected. It was also our intention that the model be applicable to all individuals with different identities (e.g., age, gender, culture, ability). The final ontology model is presented in Fig. 3.

### Phase 4: knowledge sharing and dissemination

During the development stage, the *PLaTO-Net* online platform (https://www.outdoorplaycanada.ca/plato-net/) was launched in May 2018 and was actively promoted on social media (e.g., the Outdoor Play Canada Twitter account) as well as through Outdoor Play Canada presentations. Since its launch, there have been 2,336 visitors to the PLaTO-Net webpage from 60 different countries. The knowledge sharing of PLaTO with the public was done mainly via Twitter given its utility in sharing ideas and real time information. When the Twitter handle @PLaTO_Net was first created, five tweets were posted and attracted a total of 1.3 K impressions over the first 50-day period alone (between November 12 and December 31, 2019). Since then, four tweets and 5.6 K impressions were made between January and March 2020 and one tweet and 2.0 K impressions were made between April and June 2020. No tweets were posted between July and October 2020, but 887 impressions were earned. In November 2020, detailed timelines and contents for Twitter posts were developed (see Additional File 7). Main strategies for Twitter communications were to: 1) monitor conversations and engage with already existing communities related to PLaTO; 2) offer interactive tweets that encourage responding and retweeting; 3) promote relevant individuals, researchers, groups, and organizations to show support; and, 4) be a resource to existing and potential audiences, to ultimately help build a strong PLaTO community. A total of eight tweets were updated in November 2020 which earned 7.0 K impressions, 75 likes, 31 re-tweets without comments and 4.4% engagement rate (total engagement [the number of times people engaged with a tweet by commenting on it, liking it, retweeting it, or clicking on it for any reason] / total impressions [the total number of times a tweet was loaded in a Twitter feed] $\times$ 100). Since then, in every three-month period, the following impressions and engagement rates were recorded: 11.3 K impressions and 1.7% engagement rate (between December 2020 and February 2021), 24.5 K impressions and 1.9% engagement rate (between March and May 2021), 9.9 K impressions and 1.7% engagement rate (between June and August 12, 2021).

To reach academic audiences, the members of the Steering Committee (EL, LDL, MST, PB) delivered a symposium session at the 8^th^ International Society for Physical Activity and Health Virtual Congress (ISPAH) in October 2021. The main goal of this symposium session was to present the overview, process, and outcome of this international, interdisciplinary collaboration project aimed at achieving international consensus on terminology, taxonomy, and ontology of PLaTO. The symposium was aired during the conference period (October 12 and 14, 2021). In addition to disseminating knowledge to a primarily academic audience, efforts to reach broader audiences are currently being planned, which include translating the PLaTO terminology, taxonomy, and ontology in different languages and making different versions available on the *PLaTO-Net* webpage, and delivering a series of free webinars for non-academic audiences (e.g., practitioners, educators, stakeholders, policymakers) as per requests following the publication of the results. It is important to acknowledge that even with our efforts to reach the global audience, the platforms we use may be more accessible to those who are in high-income countries. Further, knowledge sharing and dissemination plans specifically targeting audiences from low- and middle-income countries will be brainstormed during the ISPAH 2022 Congress where the launch of the Global Matrix 4.0 [41] and a general meeting for Active Healthy Kids Global Alliance (activehealthykids.org) is scheduled.

## Discussion

This paper summarized the processes and outcomes of an internationally collaborative, intersectoral, and interdisciplinary consensus project to develop a shared terminology, taxonomy, and ontology for PLaTO, a critical area of research and practice. Ignited by a discussion among experts, graduate students, and practitioners in the field of outdoor education and play at the *International Udeskole Conference* in 2018 and fueled by the establishment of *PLaTO-Net*, the consensus project was intended to provide harmonized, consensus-based terminology, taxonomy, and ontology for PLaTO to reduce confusion, create cohesion, amplify a sense of community, and support further advancement of research, practice, and policy related to PLaTO. Based on a 3.5-year-long exchange of views, consisting of the establishment of *PLaTO-Net* and the Steering Committee for the project; an extensive systematic scoping review of literature; initial development of terminology, taxonomy, and ontology through two Sub-committees; and four rounds of membership consultation surveys, this project provides terminology and taxonomy of 31 PLaTO terms and an ontology model to guide future work in PLaTO-related research and practice.

Via this project, we identified important PLaTO terms and reached agreement on definitions to create a common understanding of relevant terms. Several terms included in this project did not have universal definitions. For example, based on a recent discussion paper on urban green space [35], there is currently no internationally accepted criteria for *green space*, which makes it difficult to measure and examine its health benefits, which in turn creates research-practice gaps. Having clear, standardized definitions may help developing effective resource allocation for public health policy and land use planning. Furthermore, in a recent systematic review [42] summarizing the correlates of outdoor play and outdoor time, standardizing terminology and measurement of these terms is noted as necessity to advance the field. Outdoor play has also been conflated with other terms such as “outdoor activity” and “outdoor physical activity”, and has been used in combination with other terms such as “free play”, “active play”, and “risky play”. The standardized terminology provided in this project offers clear definitions for, as well as distinctive definitions that may be used when combining hybrid terms, such as *active outdoor play*. We anticipate that these efforts will advance measurement methods and procedures for a variety of PLaTO-related activities.

This project generated the ontology model of PLaTO. The emphasis of the PLaTO ontology model is on the interconnectedness of different activities that can take place outdoors (i.e., between play, learning, teaching, leisure, and work) and the cyclical characteristics of all elements included in the model. Here, we recognize the limitations of graphically presenting elements selectively in the shape of a tree trunk and roots, instead of being inclusive of all possible processes, effects, settings, and consequences that are far more complex than what is visually represented in Fig. 3. Nonetheless, the main contribution we sought to make through this project was to provide root ontological definitions of inputs, processes, and activity types. The dynamic and interconnected nature of elements related to PLaTO reflected in our ontology model shows that each element of the system is related to, and thus may influence or be influenced by, each of the others. The model also includes elements beyond the purview of PLaTO per se. To generate evidence that could better facilitate *PLaTO-Net* agendas, future work related to PLaTO as well as to outdoor leisure and work may consider the PLaTO ontology model to develop more inclusive programs and policies. Future research is required to clarify the roles that outdoor activities play on healthy living, environmental stewardship, climate action, and planetary health. Our hope is that this simplified visual representation may aid such research endeavors through the explicit consideration of linkages between elements and phenomena that affect planetary health and their reciprocal relationships. In addition, the standardized concept can help secure funding and a wider more harmonized vision for communication in public health internationally.

This project aimed to achieve consensus on terminology, taxonomy and ontology of PLaTO to provide support for researchers, practitioners, educators, urban designers, health promoters, environmentalists, and policymakers to facilitate intersectoral and interdisciplinary understanding and avoid potential misinterpretation of terms which could otherwise widen the gaps between different sectors and fields of research. Though some may feel that conceptualizing, categorizing, and imposing terms and definitions could limit research and practice related to PLaTO and constrain human imagination and creative ways of interacting with the environment, it is also arguable that, without conceptualization, there would be no definitions or standard meanings [43] which may, in turn, limit the basis for shared ways of thinking and acting. By conceptualizing the interaction between the environment and human activities and providing harmonized, consensus-based terms and definitions, our work opens the possibility of better understanding the environment-human interaction that is conducive to healthy living, environmental stewardship, climate action, and planetary health. It is important to acknowledge that not all PLaTO activities will be directly implicated in these outcomes; therefore, future research should consider investigating mechanisms, rather than simple associations.

### Strengths and limitations

A major strength of this consensus project was the use of a Delphi approach to develop the PLaTO terminology, taxonomy, and ontology. The systematic scoping review we conducted to summarize the evidence is another major strength; however, limitations are that the searches were restricted to children and youth aged 2–18 years and English articles only and each article was screened by one screener only. Also, the literature search for the scoping review was last done in September 2019; therefore, literature published after this time point is not reflected in this work. In addition to seeking continued input, the project also requires an update based on more recent literature in the future.

Although it was our hope that this work represents all populations regardless of their identities (e.g., nationality, gender, age, ethnicity, ability), it is likely that, by generating universal knowledge, nuances that vary by cultures, languages, experiences, and historical perspectives may not be sufficiently represented. We also acknowledge that terminology, taxonomy, and ontology generated in this work may differ by context and will evolve over time; it is for this reason we added nuance where possible. Also, while consensus was achieved, there remained a view that using the tree analogy for the final ontology model could create more confusion due to different cultural meanings attached to the tree itself. Our work will benefit from ongoing input, and we encourage stakeholders, other academics, and the general public to join us and/or reach out through our webpage (www.outdoorplaycanada.ca/plato-net/) or Twitter (https://twitter.com/PLaTO_Net).

The selection of terms and elements included in our work was primarily based on a comprehensive review of existing literature (Phase 1 of this project) and, therefore, elements that could potentially be important and emerging may have been missed and require future consideration. For instance, blue space (e.g., lakes, rivers, oceans), wild or wilderness, and other natural elements such as desert or wind were noted by a few survey participants as important elements but not included in our work. We also acknowledge this is not the first work providing definitions for terms related to PLaTO; rather, this work builds on previous work [8, 18, 22, 29–32, 35] and comprehensively examined existing literature while actively seeking and incorporating expert opinions and feedback.

Despite our efforts in seeking diverse representation for the composition of the Steering Committee, this Committee lacked perspectives from researchers or practitioners in African countries, minority cultural groups, and immigrants/refugees in high-income countries, persons living with disability, and people from gender-diverse communities. Our efforts in disseminating knowledge and seeking public feedback will intentionally target more diverse communities of people globally via multiple channels post-publication. Also, Twitter analytics provided in this work might suggest that the impact of our knowledge sharing and dissemination activities to date is trivial; however, knowledge sharing and dissemination will be done on an on-going basis post-publication. This includes enhanced curation of the *PLaTO-Net* webpage and moderation of the Twitter account; leveraging Steering Committee members’ networks, affiliated organizations, and social media channels; developing public relations and dissemination plans while further exploring partnerships and collaboration with different organizations related to PLaTO; translating the outcomes of this project in different languages and making it available online for free; and intentionally creating spaces where non-academic audiences can be engaged and involved. Furthermore, though the outcome of this paper is consensus-based among international experts, an alternative approach to standardizing PLaTO terminology that is scientifically sound (e.g., machine learning) and more contextually and culturally specific should also be considered and pursued. Therefore, potential knowledge users of the outcome of this work are encouraged to use our knowledge product where appropriate to the context and population under investigation.

### Future research priorities

Despite the robustness of our efforts, several gaps were identified that warrant future work. For example, substantial heterogeneity was observed in the qualitative feedback from survey participants for *learning* and *teaching* sub-terms. Specifically, contention existed in distinguishing *outdoor classroom* from *outdoor education*. One participant indicated that “…early adopters of the *outdoor classroom* may have referred to their work as *outdoor education*, but this pedagogical approach has evolved and is far more complex than outdoor education.” Another participant indicated that “I would not add *outdoor classroom*, but leave the outdoor setting open in the last term, *outdoor education*…*outdoor classrooms* tend to simply take indoor teaching/pedagogy outside.” There were also comments indicating the need for clearer contrast between *outdoor learning* and *outdoor education*. The most apparent difference is the context in which each activity occurs and who leads these activities; however, interpretations appear to differ by cultures. For instance, one survey participant from Canada suggested that *outdoor learning* can occur for anyone in any outdoor context whereas *outdoor education* is specific to the school context and is typically led by educators. Another survey participant suggested otherwise, indicating that, in the United Kingdom, *outdoor education* relates mainly to adventurous activities provided outside of school by specialized instructors, while *outdoor learning* is often school-based and led by mainstream teachers but includes independent learning in outdoor contexts. These examples demonstrate the difficulties in creating harmonized terms and definitions while accommodating different cultural nuances. Such distinctions were reflected in our definitions but need to be more clearly articulated in future work.

There still are terms that require further clarification. For example, *school ground* is often in a plural form, *school grounds*; however, we used *school ground* in our work for consistency with other terms that are similar in nature (e.g., playground). *Loose parts* were included as part of the outdoor settings in our ontology model, but the term is often used as a pedagogical approach to early childhood education that focuses on inspiring imagination and creativity [44]. There was also confusion on the relationship between *play* and *learning*. In outdoor spaces, *play* and *learning* are inextricably interrelated and intertwined, and *play* can serve as a vehicle for *learning*, particularly in the early childhood education and care context [45] wherein reference to “play-based learning” [46], “playful learning” [22] or “play-based approaches” (Nolan & Paatsch, 2017 [47]) is common. Our work was not to challenge the role of play in learning, but to assert that not all play explicitly promotes learning or has specific aims; rather, play can be non-purposeful and intrinsically motivated. Furthermore, engaging in *play* is not exclusive to children. People of any age can voluntarily engage in activity that is playful, fun, and/or rewarding. Our play-related terms and definitions reflect how these words can be operationalized in investigating *play* among adolescent and adult population groups, but the overall concept requires further development in the context of this field.

Developing valid and reliable measurement tools for PLaTO activities is still required. For instance, accelerometers are often used to measure *active outdoor play* among children, however, accelerometers do not accurately detect the intensity of the range of movement that a child may engage in through play (e.g., climbing a tree). Valid and reliable measurement tools for PLaTO activities are also necessary to help clarify the links between the exposure to varying PLaTO activities and the synergistic linkages with healthy living, environmental stewardship, climate action, and planetary health. In addition to advancing measurement of PLaTO for quantitative research, more qualitative work that could complement quantitative evidence is warranted. In particular, research projects in collaboration with participants themselves as researchers would greatly benefit the field to move forward. Lastly, the terminology, taxonomy, and ontology of PLaTO should continue to evolve and transform with changing times and spaces. As a first step to address this need, *PLaTO-Net* will translate PLaTO terminology, taxonomy, and ontology into different languages to reflect varying contextual, cultural, and historical perspectives and practices around the outdoors.

## Conclusion

The present consensus project provides clarification on terminology, taxonomy, and ontology of PLaTO through creating an opportunity to discuss and become aware of the nuances in how PLaTO is conceptualized and enacted in the literature. This work has a number of implications for research, policy, and practice as it advances PLaTO-based research and facilitates intersectoral and interdisciplinary collaboration, with PLaTO Net’s long-term goal of fostering and strengthening PLaTO’s synergistic linkages with healthy living, environmental stewardship, climate action, and planetary health agendas. Though consensus was achieved on PLaTO terminology, taxonomy, and ontology among experts who were part of this initiative, we recognize that these will evolve over time and space. *PLaTO-Net* is committed to working towards advancing and continually updating knowledge in the many interconnected and interrelated areas of PLaTO and in doing so to continuing to develop our capacity to communicate with, learn from, and engage with communities invested in the possibilities of PLaTO.

## Supplementary Information

**Additional file 1.**

**Additional file 2.**

**Additional file 3.**

**Additional file 4.**

**Additional file 5.**

**Additional file 6.**

**Additional file 7.**

## Data Availability

The data supporting the conclusions of this article are included within the article and its additional files.

## Abbreviations

PLaTO: Play, Learn, and Teach Outdoors
PLaTO-Net: Play, Learn, and Teach Outdoors Network
