# Correction: Play, Learn, and Teach Outdoors — Network (PLaTO-Net): terminology, taxonomy, and ontology

**DOI:** 10.1186/s12966-022-01403-z

**Published:** 2023-01-03

**Authors:** Eun-Young Lee, Louise de Lannoy, Lucy Li, Maria Isabel Amando de Barros, Peter Bentsen, Mariana Brussoni, Lindsay Crompton, Tove Anita Fiskum, Michelle Guerrero, Bjørg Oddrun Hallås, Susanna Ho, Catherine Jordan, Mark Leather, Greg Mannion, Sarah A. Moore, Ellen Beate Hansen Sandseter, Nancy L. I. Spencer, Susan Waite, Po-Yu Wang, Mark S. Tremblay, participating PLaTO-Net members

**Affiliations:** 1 School of Kinesiology & Health Studies, Queen’s University, Kingston, ON Canada; 2 Outdoor Play Canada, Children’s Hospital of Eastern Ontario Research Institute, Ottawa, ON Canada; 3 Instituto Alana, São Paulo, Brazil; 4 Copenhagen University Hospital – Bispebjerg and Frederiksberg and University of Copenhagen, Copenhagen, Denmark; 5 School of Population & Public Health, University of British Columbia, Vancouver, BC Canada; 6 Nord University, Bodø, Norway; 7 Children’s Hospital of Eastern Ontario Research Institute, Ottawa, ON Canada; 8 Western Norway University of Applied Sciences, Bergen, Norway; 9 Singapore University of Social Sciences, Singapore & Ministry of Education, Singapore, Singapore; 10 University of Minnesota & Children & Nature Network, Minneapolis, Minnesota USA; 11 Plymouth Marjon University, Plymouth, UK; 12 Faculty of Social Sciences, University of Stirling, Stirling, Scotland; 13 School of Health and Human Performance, Dalhousie University, Halifax, NS Canada; 14 Queen Maud University College, Trondheim, Norway; 15 Faculty of Kinesiology, Sport, and Recreation, University of Alberta, Edmonton, AB Canada; 16 University of Plymouth, United Kingdom & Jonkoping University, Jönköping, Sweden; 17 Department of Recreational Sport, National Taiwan University of Sport, Taiwan, Taichung, Republic of China; 18 Healthy Active Living and Obesity Research Group, CHEO Research Institute, 401 Smyth Rd, Ottawa, Ontario K1H 8 L1 Canada

**Correction: Int J Behav Nutr Phys Act 19, 66 (2022)**

**https://doi.org/10.1186/s12966-022-01294-0**

Following publication of the original article [1], the authors identified that Lindsay Crompton was omitted from the article.

The author group has been updated above and the original article [1] has been corrected.
