# Supplementary material for: Play, Learn, and Teach Outdoors—Network (PLaTO-Net): terminology, taxonomy, and ontology
Source: Int J Behav Nutr Phys Act. 2022 Jun 15;19:66. doi: 10.1186/s12966-022-01294-0 (PMC9199154; doi:10.1186/s12966-022-01294-0)
Supplement: Supplementary file 2 — Additional file 2. [file 12966_2022_1294_MOESM2_ESM.docx]

**Supplementary Table 1.** Literature search strategies.

| **Ovid MEDLINE® (1946–)** | **ERIC (1965–)** |
| --- | --- |
| 1. ((outdoor* or outside* or street* or green* or natur* or forest*) adj2 (play* or recess* or exercis* or physical activit*)).ti,ab,kf. | 1. ((outdoor* or outside* or street* or green* or natur* or forest*) adj2 (play* or recess* or exercis* or physical activit*)).tw. |
| 1. ((outdoor* or outside* or green* or natur* or forest*) adj2 (teach* or learn* or classroom* or school* or preschool* or educat*)).ti,ab,kf. | 1. ((outdoor* or outside* or green* or natur* or forest*) adj2 (teach* or learn* or classroom* or school* or preschool* or educat*)).tw |
| 1. (EOtC or udeskole or Friluftsliv).ti,ab,kf. | 1. (EOtC or udeskole or Friluftsliv).tw. |
| 1. "Play and Playthings"/ and outdoor*.tw,kf. | 1. playgrounds/ or outdoor education/ or adventure education/ |
| 1. 1 or 2 or 3 or 4 | 1. 1 or 2 or 3 or 4 or 5 |
| 1. (child* or adolescen* or toddler* or juvenile* or teen* or youth* or kid or kids or girl* or boy* or minor*).ti,ab,kf. | 1. (child* or adolescen* or toddler* or juvenile* or teen* or youth* or kid or kids or girl* or boy* or minor*).tw. |
| 1. ((pre-school* or kindergarten* or school* or highschool*) adj2 age*).ti,ab,kf. | 1. ((pre-school* or kindergarten* or school* or highschool*) adj2 age*).tw. |
| 1. exp child/ or adolescent/ | 1. children/ or young children/ or preschool children/ or toddlers/ or preadolescents/ or adolescents/ or early adolescents/ or late adolescents/ or youth/ or high school students/ or secondary school students/ or junior high school students/ or middle school students/ or elementary school students/ or child development/ |
| 1. (pupil* or first-grader* or second-grader* or third-grader* or fourth-grader* or fifth-grader* or sixth-grader* or seventh-grader* or eighth-grader* or ninth-grader* or tenth-grader* or eleventh-grader* or twelfth-grader*).ti,ab,kf. | 1. (pupil* or first-grader* or second-grader* or third-grader* or fourth-grader* or fifth-grader* or sixth-grader* or seventh-grader* or eighth-grader* or ninth-grader* or tenth-grader* or eleventh-grader* or twelfth-grader*).tw. |
| 1. 6 or 7 or 8 or 9 | 1. 7 or 8 or 9 or 10 |
| 1. 5 and 10 | 1. 6 and 11 |
| 1. 11 not (Animal not Human) | 1. limit 12 to (english language and journal articles and yr="1985 -Current") |
| 1. limit 12 to (journal article or published erratum or "retraction of publication") |  |
| 1. limit 13 to (english language and yr="1985 -Current") |  |

**Supplementary Table 2.** Members of the PLaTO-Net International Consensus Project Steering Committee.

| **Name** | **Country** | **Institution** |
| --- | --- | --- |
| Maria Isabel Amando de Barros | Brazil | Instituto Alana, Brazil |
| Peter Bentsen | Denmark | Bispebjerg and Frederiksberg Hospital |
| Mariana Brussoni | Canada | University of British Columbia |
| Tove Anita Fiskum | Norway | Nord University |
| Michelle Guerrero | Canada | Children’s Hospital of Eastern Ontario Research Institute |
| Bjørg Oddrun Hallås | Norway | Western Norway University of Applied Sciences |
| Susanna Ho | Singapore | Singapore University of Social Sciences, Ministry of Education |
| Catherine Jordan | United States of America | University of Minnesota; Children & Nature Network |
| Mark Leather | United Kingdom (England) | Plymouth Marjon University |
| Eun-Young Lee | Canada, South Korea | Queen’s University |
| Libby Lee-Hammond | Australia | Murdoch University |
| Gregory Mannion | United Kingdom (Scotland) | University of Stirling |
| Shawn Marsolais | Canada | Blind Beginnings |
| Sarah A. Moore | Canada | Dalhousie University |
| Ellen Beate Hansen Sandseter | Norway | Queen Maud University College |
| Nancy Spencer | Canada | University of Alberta |
| Mark Tremblay | Canada | Children’s Hospital of Eastern Ontario Research Institute |
| Susan Waite | United Kingdom (England) | University of Plymouth, UK; Jonkoping University, Sweden |
| Claire Warden | United Kingdom (Scotland) | Mindstretchers Academy |
| Po-Yu (Eric) Wang | Taiwan | National Taiwan University of Sport |
| Ylva Jannok Nutti | Norway | Sami University |
| Louise Zimanyi | Canada | Humber College |

**Supplementary Table 3.** Members of the PLaTO-Net International Consensus Project during Phase 2.

| **Name** | **Country** | **Institution** |
| --- | --- | --- |
| *Sub-committee A participants* | | |
| Eun-Young Lee (Facilitator) | Canada, South Korea | Queen’s University |
| Peter Bentsen | Denmark | Bispebjerg and Frederiksberg Hospital |
| Mariana Brussoni | Canada | University of British Columbia, Canada |
| Susanna Ho | Singapore | Singapore University of Social Sciences, Ministry of Education |
| Gregory Mannion | United Kingdom | University of Stirling |
| Lærke Mygind | Denmark | Deakin University |
| Matthew Peter Stevenson | Denmark | Steno Diabetes Center Copenhagen |
|  | | |
| *Sub-committee B participants* | | |
| Susan Waite (Facilitator) | United Kingdom | University of Plymouth, UK; Jonkoping University, Sweden |
| Maria Isabel Amando de Barros | Brazil | Instituto Alana, Brazil |
| Ellen Beate Hansen Sandseter | Norway | Queen Maud University College |
| Peter Elsborg | Denmark | Steno Diabetes Center Copenhagen |
| Catherine Jordan | United States of America | University of Minnesota; Children & Nature Network |
