# Supplementary material for: Play, Learn, and Teach Outdoors—Network (PLaTO-Net): terminology, taxonomy, and ontology
Source: Int J Behav Nutr Phys Act. 2022 Jun 15;19:66. doi: 10.1186/s12966-022-01294-0 (PMC9199154; doi:10.1186/s12966-022-01294-0)
Supplement: Supplementary file 3 — Additional file 3. [file 12966_2022_1294_MOESM3_ESM.pdf]

PLaTO-Net (Play, Learn, and Teach Outdoors Network) Global Harmonization Project - Membership Consultation Questionnaire

A major initiative of PLaTO-Net is to achieve international consensus on terminology, taxonomy, and ontology for outdoor play, learning, and teaching. There is considerable diversity in how approaches, contents, and contexts of outdoor play, learning, and teaching are conceptualised and enacted, and evolve across time and different cultures. Developing international consensus about key terminology, taxonomy, and ontology would facilitate sharing evidence and ideas in the field, thus contributing to advancing research, policy, and practice in outdoor play, learning, and teaching.

An International Steering Committee was formed and has begun initial work on developing a conceptual model and identifying key terms related to play, learning, and teaching outdoors. We are now seeking input on these initial efforts from the PLaTO-Net membership. We hope that the wide reach of our membership (currently 405 members from 45 countries) will assist us in achieving international consensus.

We invite you to participate in this project by completing this initial questionnaire. We plan to publish the results of our efforts. PLaTO-Net members who participate in this questionnaire and continue to participate in the consensus process will be invited as co-authors. Subsequent correspondence regarding this project will be with those who completed the questionnaire only. For this reason, responses will not be anonymous.

* Required

1. Email *

____

Part 1: Conceptual model of activities that occur in outdoor spaces, including play, learning, and teaching.

We have developed a conceptual model (please see below) to capture all aspects of activities that occur in outdoor spaces and their relation to one another. For the consensus project, our intention is to highlight the key aspects of this model that our project addresses (i.e., play, learning, teaching), while incorporating other concepts in our overall conceptual model to acknowledge and express that all the aspects included in the model are interrelated and interconnected. It is our aim that this model be applicable to all ages, genders, cultures, and abilities. Please note that this will not be the only model included in the publication; we plan on developing additional illustrations and/or diagrams related to specific aspects of play, learning, and teaching outdoors.

Model Caption: This is a conceptual model of activities that occur in outdoor spaces.

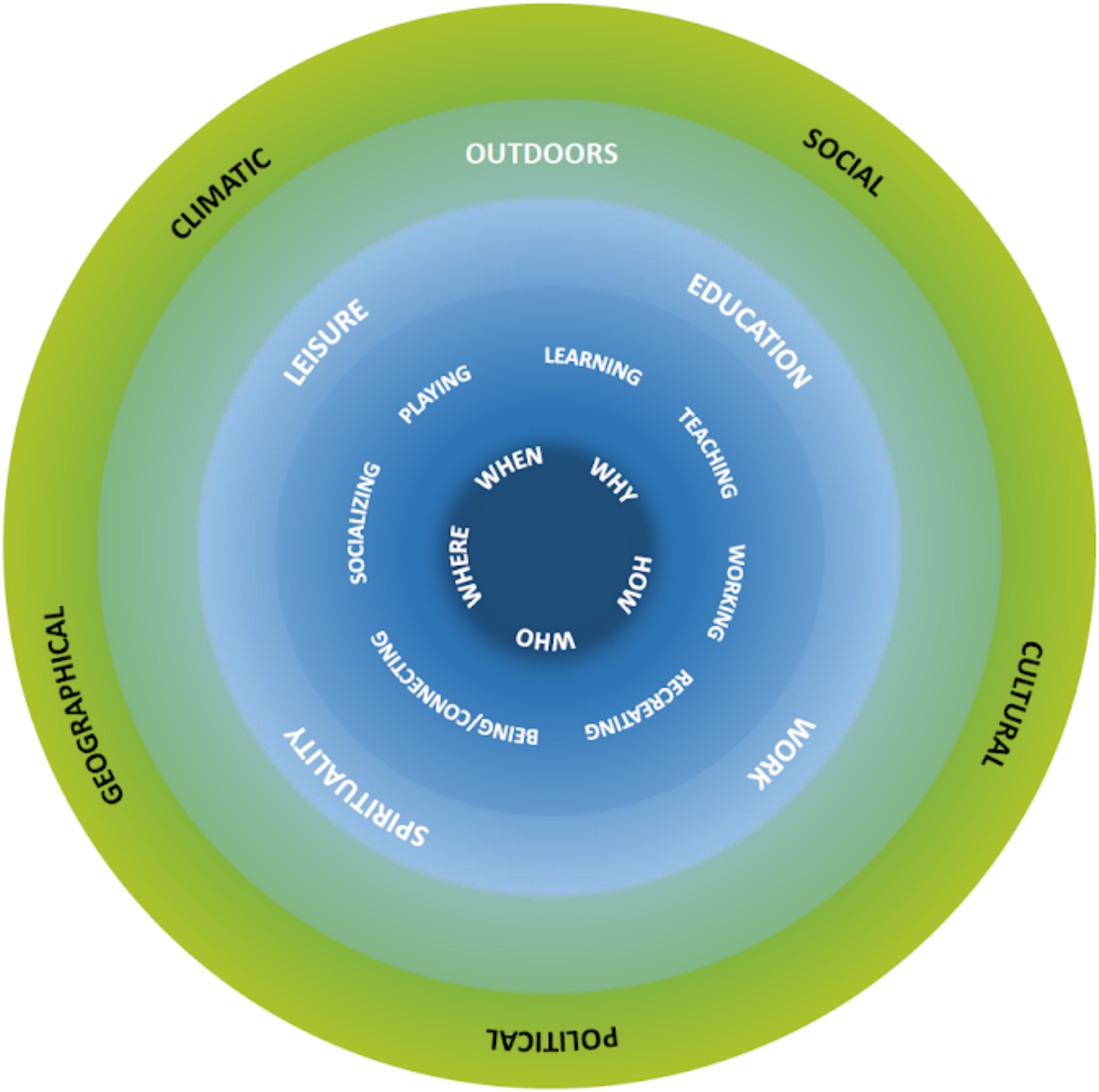

Image Description: A Conceptual model of activities that occur in outdoor spaces.

The model is circular with 5 concentric rings. The centre ring is dark blue with text ‘who, where, when, why, and how’ to denote the context. The next ring is a medium shade of blue with text ‘playing, learning, teaching, working, recreating, being/connecting, and socializing' to indicate the dynamicity of each context (actions expressed using verbs). The next ring is light blue with text 'education, work, spirituality, and leisure' to indicate general/thematic activities that are performed outdoors. The next ring is mint green with text 'outdoors' to indicate the main setting of interest. The outermost ring is emerald green with text 'social, cultural, political, geographical, and climatic' to indicate influences on/consequences of outdoor activities. The rings are interpreted as circulating around each other to indicate the dynamicity and fluidity within and between rings. The edges between each ring are purposefully blurred to highlight the interconnectivity of terms and acknowledge that there may be other terms/concepts not included in the model.

2. Do you like the overall design of the model?

Mark only one oval.

	1	2	3	4	5	
Strongly dislike	⬭	⬭	⬭	⬭	⬭	Strongly like

3. Do you have any specific comments or suggestions regarding the model (e.g., likes/dislikes, important features, improvements, utility; all are welcome!)?

Part 2: Key terms related to play, learning, and teaching outdoors.

We aim to achieve clear, common, harmonized, and widely accepted terminology, taxonomy, and ontology related to play, learning, and teaching outdoors that have utility across all ages, genders, cultures, and abilities, adapted to our contemporary understanding. As a first step, we have undergone a scoping review to identify a comprehensive list of key terms for which we will determine consensus definitions.

For each term please indicate whether you think it should be included in the list of key terms related to play, learning, and teaching outdoors.

We have organized the terms into the following taxonomic categories: play, learning, teaching/education, school, and outdoors.

4. Play (outdoors): Agreement with inclusion of the following as key terms:

Mark only one oval per row.

	Yes	No
Outdoor play (Hybrid terms (i.e. outdoor free play, outdoor active free play) will fall under outdoor play)		
Free play		
Land-based play		
Nature play		
Risky play		
Free-flow play		
Outdoor recess/break time		
Active play		
Social play		

5. Learning (outdoors): Agreement with inclusion of the following as key terms:

Mark only one oval per row.

	Yes	No
Outdoor learning	⬭	⬭
Nature-based learning	⬭	⬭
Land-based learning	⬭	⬭
Place-based learning	⬭	⬭
Environmental learning	⬭	⬭
Informal learning	⬭	⬭
Learning outside the classroom/ Education outside the classroom	⬭	⬭
Service learning	⬭	⬭

6. Teaching/Education (outdoors): Agreement with inclusion of the following as key terms:

Mark only one oval per row.

	Yes	No
Outdoor teaching/Teaching (outdoors)	⬭	⬭
Outdoor education	⬭	⬭
Nature-based education	⬭	⬭
Place-based education	⬭	⬭
Adventure-based education	⬭	⬭
Environmental education	⬭	⬭

7. (Pre)School (outdoors): Agreement with inclusion of the following as key terms:

Mark only one oval per row.

	Yes	No
Outdoor school	⬭	⬭
Forest school	⬭	⬭
Nature school	⬭	⬭

8. Outdoors : Agreement with inclusion of the following as key terms:

Mark only one oval per row.

	Yes	No
Home range	⬭	⬭
Nature	⬭	⬭
Natural environment	⬭	⬭
Built environment	⬭	⬭
Green schoolyards	⬭	⬭
Outdoor play-spaces	⬭	⬭
Nature play-spaces	⬭	⬭
Playground	⬭	⬭
Nature playground	⬭	⬭
Garden	⬭	⬭

9. Are there other terms that should be included as key terms? If so, please list below.

10. Additional comments regarding the key terms? (e.g., redundancies, renaming of terms, taxonomy/grouping of terms)

11. Do you have any additional comments, suggestions, and/or considerations in relation to making this project applicable to all ages, genders, cultures, and ability levels?

PLaTO-Net Logo

We have developed the following logo for PLaTO-Net. Please provide your feedback on the logo below.

Logo Caption: This is a logo for PLaTO-Net (Play, Learn, and Teach Outdoors Network)

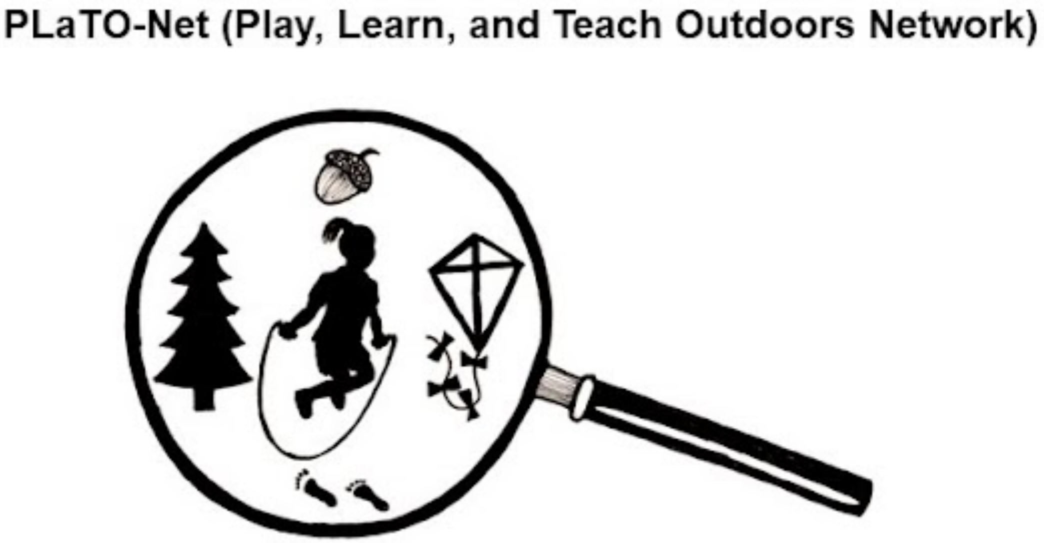

Image Description: A logo for PLaTO-Net (Play, Learn, and Teach Outdoors Network)
The black-and-white logo is a magnifying glass with five items inside the glass: in the centre there is a person skipping rope, above them there is an acorn, and moving clockwise at 3 o'clock there is a kite, at 6 o'clock a pair of children's footprints, and at 9 o'clock a coniferous tree.

12. Do you like the overall design of the logo?

Mark only one oval.

	1	2	3	4	5	
Strongly dislike	◯	◯	◯	◯	◯	Strongly like

13. Do you have any specific comments or suggestions regarding the logo (e.g. likes/dislikes, important features, improvements; all are welcome!)?

____
____
____
____
____

Part 4: Demographics

14. Please indicate your sector of work/study (select all that apply):

Check all that apply.

- [ ] Research
- [ ] Education - (pre)school
- [ ] Education - post-secondary
- [ ] Play work
- [ ] Policy/Government
- [ ] For-profit organization
- [ ] Not-for-profit organization

Other: [ ] ______________________

15. Please indicate your years of experience working in your field:

Mark only one oval.

- ( ) <1 year
- ( ) 1-5 years
- ( ) 6-9 years
- ( ) >10 years

If you feel comfortable sharing, please respond to the following with regard to how you self-identify:

16. Gender

17. Disability

18. Culture, race, and/or ethnicity

19. Country of origin

20. Are there any other ways you identify and would like to share?

Additional comments

21. Additional comments?

Thank you for completing the questionnaire!

This content is neither created nor endorsed by Google.

Google Forms

Round 2: PLaTO-Net (Play, Learn, and Teach Outdoors Network) Global Harmonization Project - Membership Consultation Questionnaire

Thank you for your extensive feedback on the PLaTO-Net conceptual model and key terms related to play, learning, and teaching outdoors. We read your comments and in response have developed an entirely new model and new strategy with regard to the key terms.

We are seeking your input on the new model and key terms. As a reminder, our aim with this project is to establish international consensus about key terminology, taxonomy, and ontology to facilitate sharing evidence and ideas in the field and advance research, policy, and practice in outdoor play, learning, and teaching. We plan to publish the results of our efforts. PLaTO-Net members who participate in this questionnaire and continue to participate in the consensus process will be invited as co-authors. Subsequent correspondence regarding this project will be with those who complete the questionnaire only. For this reason, respondents will not be anonymous, but individual responses will remain confidential.

* Required

1. Email *

Part 1: Conceptual model of activities that occur in outdoor spaces, including play, learning, and teaching.

We have developed a conceptual model (please see below) to capture all aspects of activities that occur in outdoor spaces and their relation to one another. For the consensus project, our intention is to highlight the key aspects that our project addresses (i.e., play, learning, teaching), while incorporating other relevant concepts to recognize that all the aspects included in the model are interrelated and interconnected. It is our aim that this model be applicable to all individuals with different identities (e.g., age, gender, culture) and abilities. Please note that this will not be the only or final model that will be included in the publication; we plan on improving this model and developing additional illustrations and/or diagrams related to specific aspects of play, learning, and teaching outdoors.

Model Caption: This is a conceptual model of activities that occur in outdoor spaces.

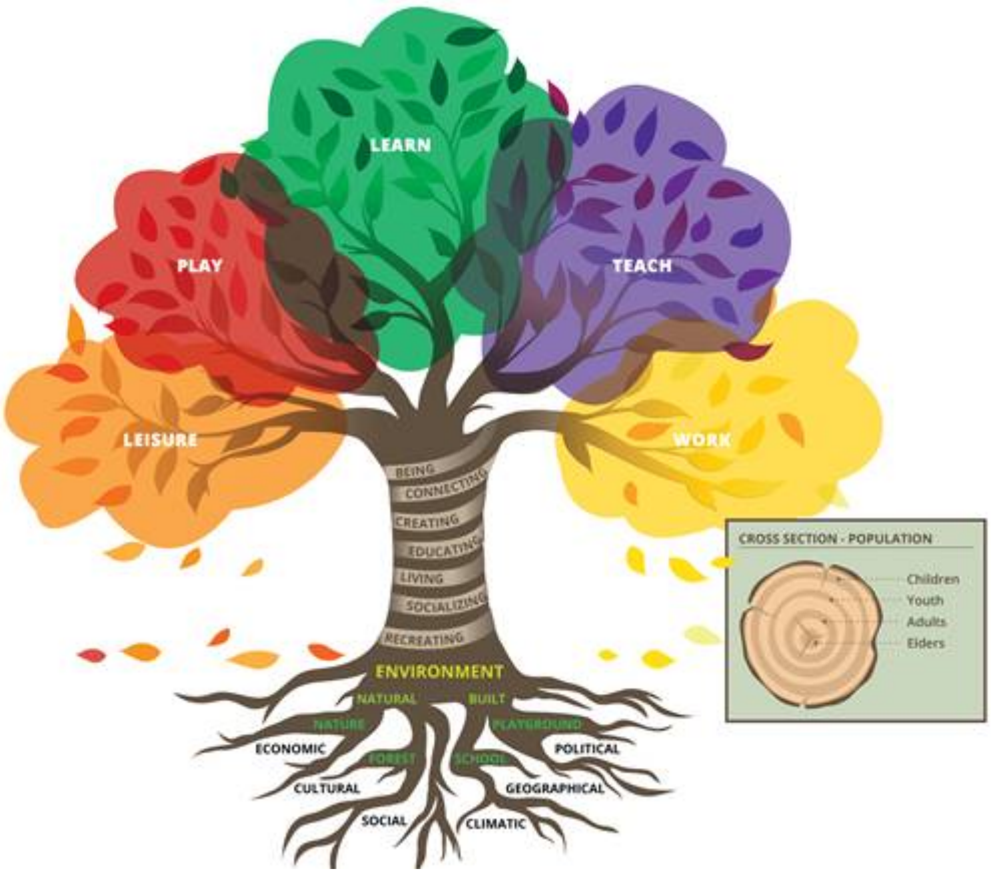

Image Description: The PLaTO-Net Tree Conceptual model of activities that occur in outdoor spaces.

In the soil surrounding the tree roots are the terms ‘economic, cultural, social, climatic, geographical, and political’ which denote influences on/consequences of outdoor activities. Along the brown roots are examples of outdoor settings, starting at the top of the roots with ‘environment’, branching out to the terms ‘natural’ and ‘built’, leading to ‘nature, forest’ and ‘school, playground’, respectively. Wrapped around the brown trunk of the tree are examples of actions that can be done in the outdoors (recreating, socializing, living, educating, creating, connecting, and being) which lead collectively to the main types of activities that can be performed outdoors, in the leaves. These activities are colour coded with ‘leisure’ in orange, ‘play’ in red, ‘learn’ in green, ‘teach’ in purple, and ‘work’ in yellow. Some leaves have fallen back down to the soil to indicate the cyclical and interconnected relationship between all elements. There is also a box to the right of the tree with a cross-section indicating the rings of the tree, with the labels ‘children’, ‘youth’, ‘adults’, and ‘elders’ in concentric rings moving from the outermost ring to the centre, just as the youngest tree rings are at the edge, and the oldest at the centre.

2. Do you like the overall design of the model?

Mark only one oval.

	1	2	3	4	5	
Strongly dislike	◯	◯	◯	◯	◯	Strongly like

3. Do you have any specific comments or suggestions regarding the model (e.g., likes/dislikes, important features, improvements, utility; all are welcome!)?

____
____
____
____
____

Part 2: PLaTO-Net Logo

We have developed a new logo for PLaTO-Net that is a simplified version of the conceptual model above. Please provide your feedback on the logo below.

Logo Caption: This is a logo for PLaTO-Net (Play, Learn, and Teach Outdoors Network)

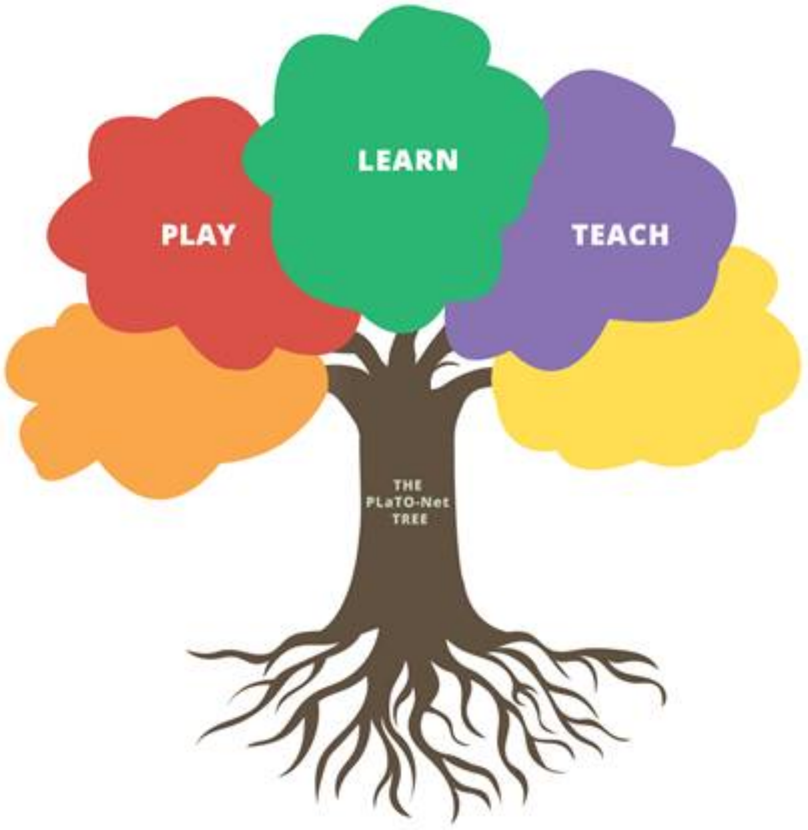

A logo for PLaTO-Net (Play, Learn, and Teach Outdoors Network).

The logo is developed by simplifying the conceptual model and in the shape of a tree. The tree has a brown trunk and roots and five colour-coded leaf sections in the branches. The words 'The PlaTO-Net Tree' are positioned at the centre of the trunk, and the terms 'play' (colour coded in red), 'learn' (colour coded in green) and 'teach' (colour coded in purple) are in the leaves.

4. Do you like the overall design of the logo?

Mark only one oval.

	1	2	3	4	5	
Strongly dislike	⬭	⬭	⬭	⬭	⬭	Strongly like

5. Do you have any specific comments or suggestions regarding the logo (e.g. likes/dislikes, important features, improvements; all are welcome!)?

Part 3: Glossary of the PLaTO (Play, Learn, and Teach Outdoors) Terms

We aim to achieve clear, common, harmonized, and widely accepted terminology, taxonomy, and ontology related to play, learning, and teaching outdoors that have utility across all ages, genders, cultures, and abilities, adapted to our contemporary understanding.

To do so, we propose that our project provides definitions for single key terms (outdoors, play, education, learning, teaching) and working definitions for hyponyms (e.g. nature, free play, experiential learning/education) based on their frequency and commonality according to our completed scoping review.

We have organized the terms according to the graph below, with the term 'outdoor' at the top, branching out to 'play' and 'education', with education branching out to 'learning' and 'teaching'. Please provide feedback on the overall layout of the terms, as indicated in the tables below. We will add definitions for each term once we decide on the terms to be included in the manuscript.

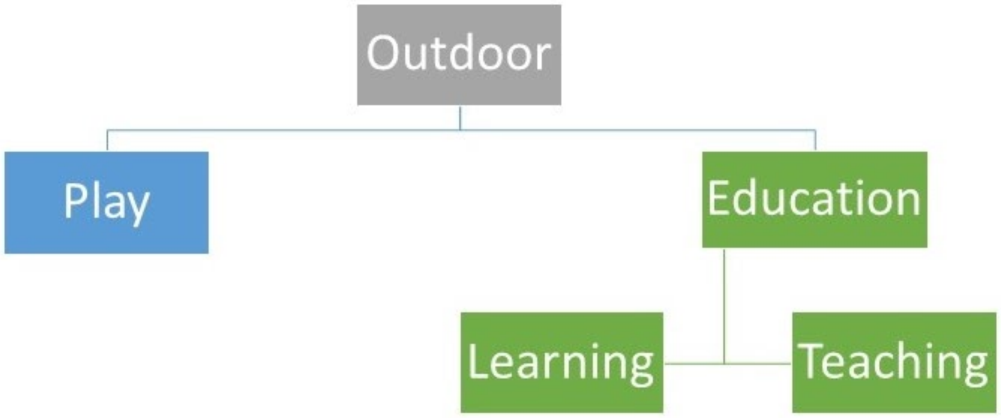

Table 1. PLaTO-Net root terms and sub-terms

Root and sub-terms	Examples
**Outdoors**	• Friluftsliv, open air, forested areas
**Nature**	• Plants, animals, the landscape, and other features and products of the earth
**Natural environment**	• Human-made: Gardens, nature playgrounds, and urban parks<br>• Wild: Wooded areas, meadows and beaches, wilderness
**Built environment**	• Parks, playgrounds
**Playground**	• Nature play-gardens
**Play**	• See Table 2
**Education**	• See Table 3
**Learning**	
**Teaching**	

PLaTO Terms (Hypernyms)	Pedagogical tools	Context
**Outdoor play**	None	• Outdoors
**Outdoor education**<br>**Outdoor learning**<br>**Outdoor teaching**	Experiential<br>• Play-based<br>• Environmental<br>• Place-responsive<br>• Place-based	• For school-aged children: Outdoor school, Udeskole (Denmark),<br>• Uteskole (Norway), utomhuspedagogik (Sweden), Draußenschule (Germany)<br>• For preschool children: Forest school, nature school, Forest and nature school, all weather school, bush school, rain or shine school

6. Do you like the overall structure of Table 1?

Mark only one oval.

	1	2	3	4	5	
Strongly dislike	◯	◯	◯	◯	◯	Strongly like

7. Do you have any specific comments or suggestions regarding Table 1 (e.g. likes/dislikes, important features, improvements; all are welcome!)?

Table 2. Outdoor Play Hyponyms

Outdoor Play Hyponyms	Synonyms	Antonyms
Free play	• Unstructured play<br>• Spontaneous play<br>• For children: child-directed play	• Structured play, guided play, purposeful play<br>• For children: Adult-directed play
Nature play	• Wild play<br>• Natural play	
Risky play		
Active play	• Vigorous play<br>• Physical activity play	• Non-active play
Social play	• Cooperative play<br>• Associative play<br>• Group play	• Solitary play
Pedagogical play	• Play for learning<br>• Purposeful play	

8. Do you like the overall structure of Table 2?

Mark only one oval.

	1	2	3	4	5	
Strongly dislike	◯	◯	◯	◯	◯	Strongly like

9. Do you have any specific comments or suggestions regarding Table 2 (e.g. likes/dislikes, important features, improvements; all are welcome!)?

Table 3. Outdoor Education Hyponyms

Outdoor Education Hyponyms	Synonyms	Common attribute
Experiential learning/education	• Hands-on<br>• Non-formal<br>• Erlebnispädagogik (Germany)<br>• Erfahrungs Lernen (Germany)	Methods
Adventure-based learning/education		Approach
Environmental learning/education		Process
Place-based learning/education	• Land-based<br>• Place-responsive	Approach
Nature-based learning/education	• Learning through exposure to nature<br>• Learning with nature	Approach
Play-based learning	• Learning through play	Methods
Outdoor school	• Draußenschule (Germany)<br>• Udeskole/Uteskole (Scandinavian)<br>• Education Outside the Classroom (EOtC)<br>• Outdoor classroom	Context
Other hybrid terms: free active play, active outdoor play, unstructured outdoor play		

10. Do you like the overall structure of Table 3?

Mark only one oval.

	1	2	3	4	5	
Strongly dislike	○	○	○	○	○	Strongly like

11. Do you have any specific comments or suggestions regarding Table 3 (e.g. likes/dislikes, important features, improvements; all are welcome!)?

____

____

____

____

____

Additional comments

12. Additional comments?

Thank you for completing the questionnaire!

This content is neither created nor endorsed by Google.

Google Forms

Round 2: PLaTO-Net (Play, Learn, and Teach Outdoors Network) Global Harmonization Project - Membership Consultation Questionnaire

Thank you for your extensive feedback on the PLaTO-Net conceptual model and key terms related to play, learning, and teaching outdoors. We read your comments and in response have developed an entirely new model and new strategy with regard to the key terms.

We are seeking your input on the new model and key terms. As a reminder, our aim with this project is to establish international consensus about key terminology, taxonomy, and ontology to facilitate sharing evidence and ideas in the field and advance research, policy, and practice in outdoor play, learning, and teaching. We plan to publish the results of our efforts. PLaTO-Net members who participate in this questionnaire and continue to participate in the consensus process will be invited as co-authors. Subsequent correspondence regarding this project will be with those who complete the questionnaire only. For this reason, respondents will not be anonymous, but individual responses will remain confidential.

* Required

1. Email *

____

Part 1: Conceptual model of activities that occur in outdoor spaces, including play, learning, and teaching.

We have developed a conceptual model (please see below) to capture all aspects of activities that occur in outdoor spaces and their relation to one another. For the consensus project, our intention is to highlight the key aspects that our project addresses (i.e., play, learning, teaching), while incorporating other relevant concepts to recognize that all the aspects included in the model are interrelated and interconnected. It is our aim that this model be applicable to all individuals with different identities (e.g., age, gender, culture) and abilities. Please note that this will not be the only or final model that will be included in the publication; we plan on improving this model and developing additional illustrations and/or diagrams related to specific aspects of play, learning, and teaching outdoors.

Model Caption: This is a conceptual model of activities that occur in outdoor spaces.

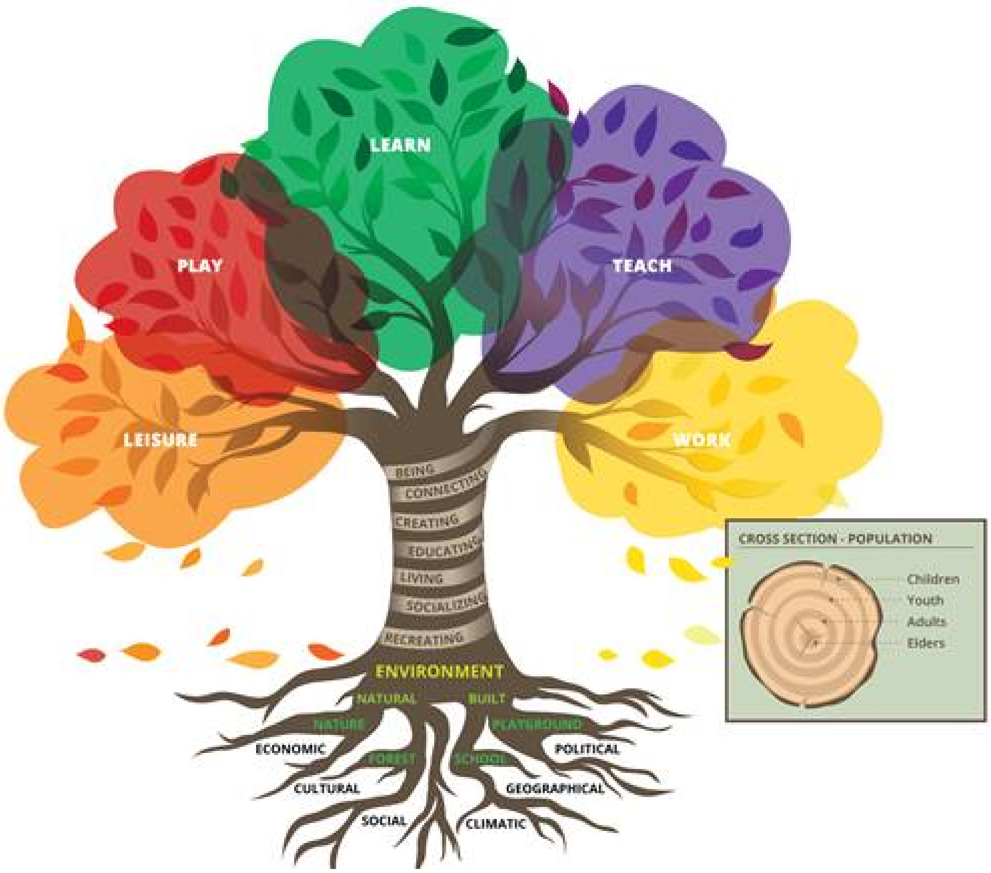

Image Description: The PLaTO-Net Tree Conceptual model of activities that occur in outdoor spaces.

In the soil surrounding the tree roots are the terms ‘economic, cultural, social, climatic, geographical, and political’ which denote influences on/consequences of outdoor activities. Along the brown roots are examples of outdoor settings, starting at the top of the roots with ‘environment’, branching out to the terms ‘natural’ and ‘built’, leading to ‘nature, forest’ and ‘school, playground’, respectively. Wrapped around the brown trunk of the tree are examples of actions that can be done in the outdoors (recreating, socializing, living, educating, creating, connecting, and being) which lead collectively to the main types of activities that can be performed outdoors, in the leaves. These activities are colour coded with ‘leisure’ in orange, ‘play’ in red, ‘learn’ in green, ‘teach’ in purple, and ‘work’ in yellow. Some leaves have fallen back down to the soil to indicate the cyclical and interconnected relationship between all elements. There is also a box to the right of the tree with a cross-section indicating the rings of the tree, with the labels ‘children’, ‘youth’, ‘adults’, and ‘elders’ in concentric rings moving from the outermost ring to the centre, just as the youngest tree rings are at the edge, and the oldest at the centre.

2. Do you like the overall design of the model?

Mark only one oval.

	1	2	3	4	5	
Strongly dislike	◯	◯	◯	◯	◯	Strongly like

3. Do you have any specific comments or suggestions regarding the model (e.g., likes/dislikes, important features, improvements, utility; all are welcome!)?

____

____

____

____

____

Part 2: PLaTO-Net Logo

We have developed a new logo for PLaTO-Net that is a simplified version of the conceptual model above. Please provide your feedback on the logo below.

Logo Caption: This is a logo for PLaTO-Net (Play, Learn, and Teach Outdoors Network)

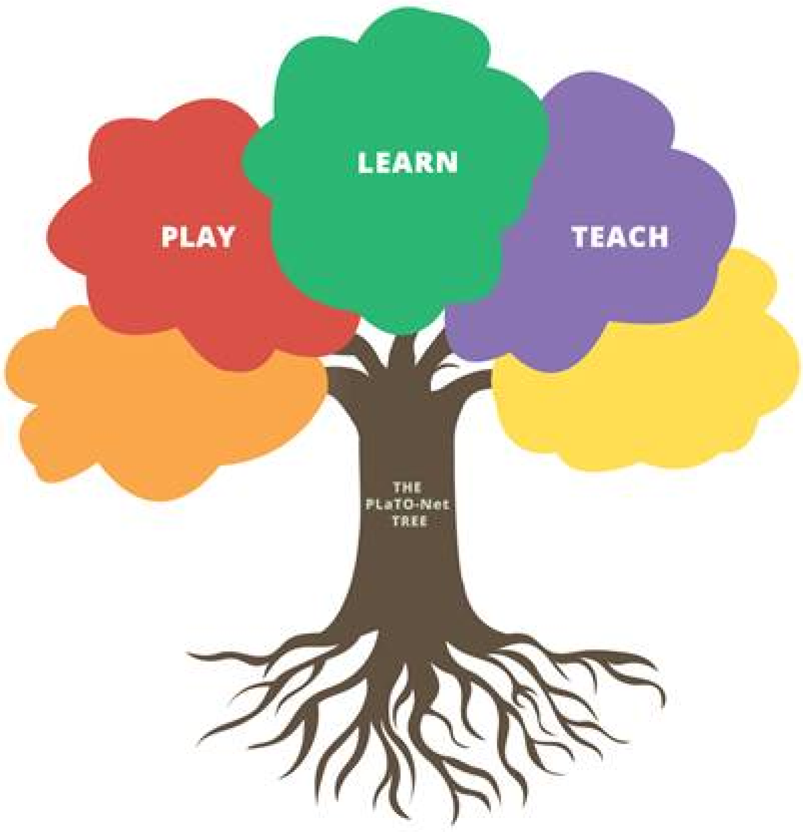

A logo for PLaTO-Net (Play, Learn, and Teach Outdoors Network).

The logo is developed by simplifying the conceptual model and in the shape of a tree. The tree has a brown trunk and roots and five colour-coded leaf sections in the branches. The words 'The PlaTO-Net Tree' are positioned at the centre of the trunk, and the terms 'play' (colour coded in red), 'learn' (colour coded in green) and 'teach' (colour coded in purple) are in the leaves.

4. Do you like the overall design of the logo?

Mark only one oval.

	1	2	3	4	5	
Strongly dislike	◯	◯	◯	◯	◯	Strongly like

5. Do you have any specific comments or suggestions regarding the logo (e.g. likes/dislikes, important features, improvements; all are welcome!)?

Part 3: Glossary of the PLaTO (Play, Learn, and Teach Outdoors) Terms

We aim to achieve clear, common, harmonized, and widely accepted terminology, taxonomy, and ontology related to play, learning, and teaching outdoors that have utility across all ages, genders, cultures, and abilities, adapted to our contemporary understanding.

To do so, we propose that our project provides definitions for single key terms (outdoors, play, education, learning, teaching) and working definitions for hyponyms (e.g. nature, free play, experiential learning/education) based on their frequency and commonality according to our completed scoping review.

We have organized the terms according to the graph below, with the term 'outdoor' at the top, branching out to 'play' and 'education', with education branching out to 'learning' and 'teaching'. Please provide feedback on the overall layout of the terms, as indicated in the tables below. We will add definitions for each term once we decide on the terms to be included in the manuscript.

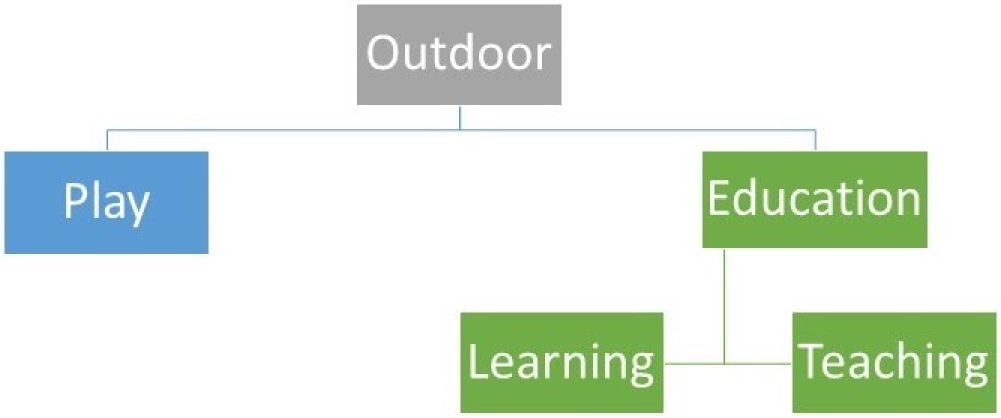

Table 1. PLaTO-Net root terms and sub-terms

Root and sub-terms	Examples
**Outdoors**	• Friluftsliv, open air, forested areas
**Nature**	• Plants, animals, the landscape, and other features and products of the earth
**Natural environment**	• Human-made: Gardens, nature playgrounds, and urban parks<br>• Wild: Wooded areas, meadows and beaches, wilderness
**Built environment**	• Parks, playgrounds
**Playground**	• Nature play-gardens
**Play**	• See Table 2
**Education**	• See Table 3
**Learning**	
**Teaching**	

PLaTO Terms (Hypernyms)	Pedagogical tools	Context
**Outdoor play**	None	• Outdoors
**Outdoor education**	Experiential	• For school-aged children: Outdoor school, Udeskole (Denmark),<br>• Uteskole (Norway), utomhuspedagogik (Sweden), Draußenschule (Germany)<br>• For preschool children: Forest school, nature school, Forest and nature school, all weather school, bush school, rain or shine school
**Outdoor learning**	• Play-based	
**Outdoor teaching**	• Environmental<br>• Place-responsive<br>• Place-based	

6. Do you like the overall structure of Table 1?

Mark only one oval.

	1	2	3	4	5	
Strongly dislike	◯	◯	◯	◯	◯	Strongly like

7. Do you have any specific comments or suggestions regarding Table 1 (e.g. likes/dislikes, important features, improvements; all are welcome!)?

Table 2. Outdoor Play Hyponyms

Outdoor Play Hyponyms	Synonyms	Antonyms
**Free play**	• Unstructured play<br>• Spontaneous play<br>• For children: child-directed play	• Structured play, guided play, purposeful play<br>• For children: Adult-directed play
**Nature play**	• Wild play<br>• Natural play	
**Risky play**		
**Active play**	• Vigorous play<br>• Physical activity play	• Non-active play
**Social play**	• Cooperative play<br>• Associative play<br>• Group play	• Solitary play
**Pedagogical play**	• Play for learning<br>• Purposeful play	

8. Do you like the overall structure of Table 2?

Mark only one oval.

	1	2	3	4	5	
Strongly dislike	◯	◯	◯	◯	◯	Strongly like

9. Do you have any specific comments or suggestions regarding Table 2 (e.g. likes/dislikes, important features, improvements; all are welcome!)?

Table 3. Outdoor Education Hyponyms

Outdoor Education Hyponyms	Synonyms	Common attribute
Experiential learning/education	• Hands-on<br>• Non-formal<br>• Erlebnispädagogik (Germany)<br>• Erfahrungs Lernen (Germany)	Methods
Adventure-based learning/education		Approach
Environmental learning/education		Process
Place-based learning/education	• Land-based<br>• Place-responsive	Approach
Nature-based learning/education	• Learning through exposure to nature<br>• Learning with nature	Approach
Play-based learning	• Learning through play	Methods
Outdoor school	• Draußenschule (Germany)<br>• Udeskole/Uteskole (Scandinavian)<br>• Education Outside the Classroom (EOtC)<br>• Outdoor classroom	Context
Other hybrid terms: free active play, active outdoor play, unstructured outdoor play		

10. Do you like the overall structure of Table 3?

Mark only one oval.

	1	2	3	4	5	
Strongly dislike	◯	◯	◯	◯	◯	Strongly like

11. Do you have any specific comments or suggestions regarding Table 3 (e.g. likes/dislikes, important features, improvements; all are welcome!)?

____

____

____

____

____

Additional comments

12. Additional comments?

____

____

____

____

____

Thank you for completing the questionnaire!

This content is neither created nor endorsed by Google.

Google Forms

Round 3: PLaTO-Net (Play, Learn, and Teach Outdoors Network) Global Harmonization Project - Membership Consultation Questionnaire

Thank you for your extensive feedback on the PLaTO-Net conceptual model and key terms related to play, learning, and teaching outdoors. We read your comments and in response have made several changes to the model, logo and have re-structured the key terms.

We are seeking consensus on the model, logo, and key terms. As a reminder, our aim with this project is to establish international consensus about key terminology, taxonomy, and ontology to facilitate sharing evidence and ideas in the field and advance research, policy, and practice in outdoor play, learning, and teaching. We plan to publish the results of our efforts. PLaTO-Net members who participate in this questionnaire and continue to participate in the consensus process will be invited as co-authors. Subsequent correspondence regarding this project will be only with those who complete the questionnaire only. For this reason, respondents will not be anonymous, but individual responses will remain confidential.

* Required

1. Email *

____

Part 1: Conceptual model of activities that occur in outdoor spaces, including play, learning, and teaching.

We have developed a conceptual model (please see below) to capture all aspects of activities that occur in outdoor spaces and their relation to one another. For the consensus project, our intention is to highlight the key aspects that our project addresses (i.e., play, learning, teaching), while incorporating other relevant concepts to recognize that all the aspects included in the model are interrelated and interconnected. It is our aim that this model be applicable to all individuals with different identities (e.g., age, gender, culture) and abilities. Please note that this will not be the only model included in the publication; we plan on improving this model and developing additional illustrations and/or diagrams related to specific aspects of play, learning, and teaching outdoors.

Model Caption: This is a conceptual model of activities that occur in outdoor spaces.

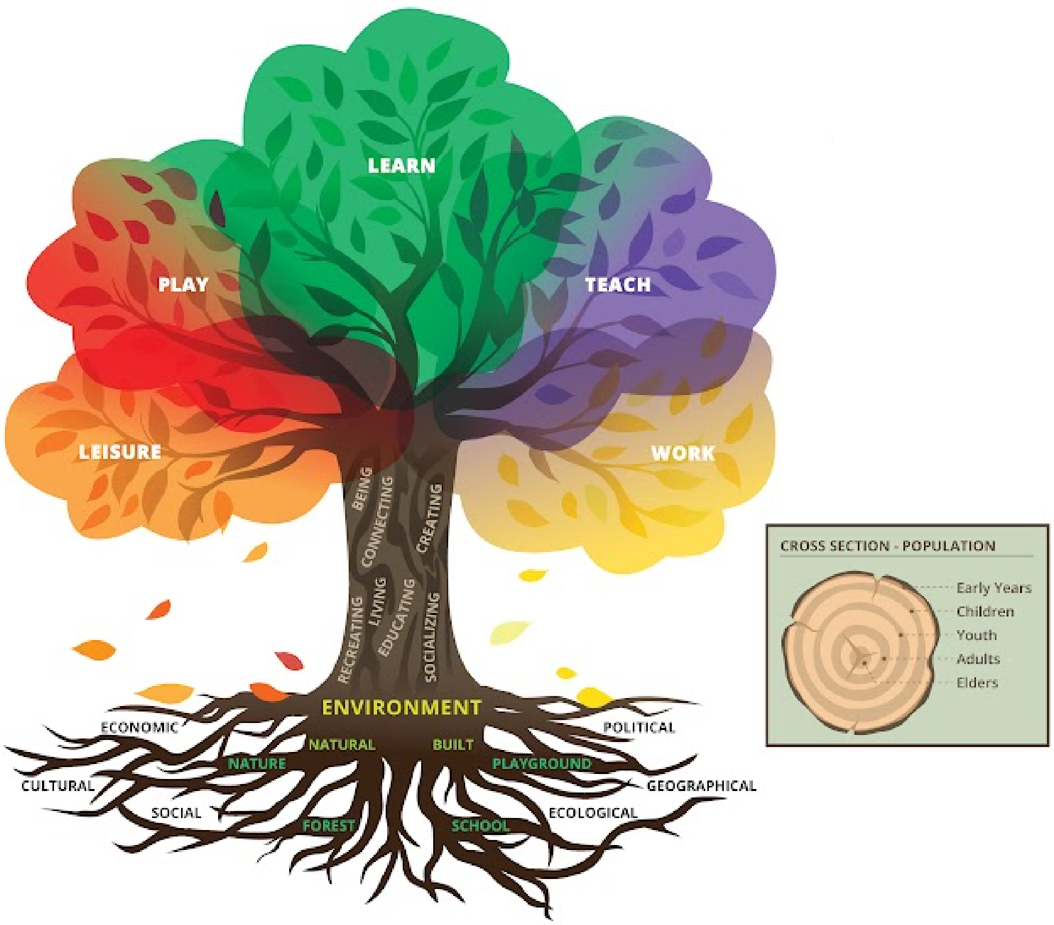

Image Description: The PLaTO-Net Tree Conceptual model of activities that occur in outdoor spaces.

Starting at the bottom of the model, surrounding the tree roots in the earth, are the terms ‘economic, cultural, social, ecological, geographical, and political’ which denote some of the main influences on/consequences of outdoor activities. Along the roots are examples of outdoor settings, starting at the top of the roots with ‘environment’, branching out to the terms ‘natural’ and ‘built’, leading to ‘nature, forest’ and ‘school, playground’, respectively. The roots overlap with each other to indicate inter-relationships between all terms in the earth and roots. Along the bark of the tree trunk are examples of actions that can be done in the outdoors (recreating, socializing, living, educating, creating, connecting, and being), where the bark again overlaps with the different terms to indicate the connectivity of and fluidity between these terms. The trunk leads into the main types of activities that can be performed outdoors, in the leaves. These activities span across a range of colours that blend into and overlap with each other, with ‘leisure’ in orange, leading into ‘play’ in red, ‘learn’ in green, ‘teach’ in purple, and ‘work’ in yellow. Some leaves have fallen back down to the soil to indicate the cyclical and interconnected relationship between all elements. There is also a box to the right of the tree with a cross-section indicating the rings of the tree, with the labels ‘early years', children’, ‘youth’, ‘adults’, and ‘elders’ in concentric rings moving from the outermost ring to the centre, just as the youngest tree rings are at the edge, and the oldest at the centre.

2. Do you agree with the design of the PLaTO-Net model?

Mark only one oval.

	1	2	3	4	5	
Strongly disagree	◯	◯	◯	◯	◯	Strongly agree

3. Do you have any final comments regarding the model?

____
____
____
____
____

Part 2: PLaTO-Net Logo

The logo for PLaTO-Net is a simplified version of the conceptual model above. Please provide your feedback on the logo below.

Logo Caption: This is a logo for PLaTO-Net (Play, Learn, and Teach Outdoors Network)

PLAY · LEARN · TEACH · OUTDOORS
PLaTO-Net

Image Description: A logo for PLaTO-Net (Play, Learn, and Teach Outdoors Network)

The logo is a simplified version of the PLaTO-Net conceptual tree model. The tree has a brown trunk and roots and three coloured leaf sections (from left to right: red, green, and purple) that overlap with each other. There is a brown ring encircling the tree. Along the top of the outside of that ring are the words: Play Learn Teach Outdoors in brown font. Along the bottom of the outside of the ring is the word 'PlaTO-Net' in larger brown font.

4. Do you agree with the design of the logo?

Mark only one oval.

	1	2	3	4	5	
Strongly disagree	⬭	⬭	⬭	⬭	⬭	Strongly agree

5. Do you have any final comments regarding the logo?

____

____

____

____

____

Part 3: Glossary of the PLaTO (Play, Learn, and Teach Outdoors) Terms

We aim to achieve clear, common, harmonized, and widely accepted terminology, taxonomy, and ontology related to play, learning, and teaching outdoors that have utility across all ages, genders, cultures, and abilities, adapted to our contemporary understanding.

Primarily driven by the scoping review on terminology of outdoor play, learning, and teaching that we conducted over the past year, we propose that our project provides definitions for single root terms (outdoors, outdoor play, outdoor learning, and outdoor teaching) and sub-terms based on their frequency and commonality according to our completed scoping review, and other relevant terms either identified by our scoping review or suggested by the survey respondents in the previous round.

Please see below to know about our process of identifying root terms, sub-terms, and other relevant terms:

1. Systematic coping review: A systematic scoping review was conducted between August 2019 and March 2020. The goal of the scoping review was to objectively identify a comprehensive list of key terms and definitions on playing, learning and teaching in the outdoors. From this review, we identified a total of 428 terms with 984 definitions, representing 35 unique countries from 441 studies.
2. Subcommittee for ontology, taxonomy, and terminology: The goal of this subcommittee work was to select and classify key terms and definitions from the list created based on the scoping review.
3. Two previous surveys: Based on the feedback received from the respondents who participated in the previous two rounds of surveys, we have added terms that are less common but identified as important.

For each term please indicate whether you agree it should be included in the list of terms related to play, learning, and teaching outdoors.

Note:
1. These terms are identified as commonly used terms in a scoping review of terminology used in outdoor play, learning, and teaching research.
2. Terms included in this table do not have hierarchical relationships with each other; rather, they are interconnected and not mutually exclusive.
3. Working definitions, key references, terms used in/translated into different languages, and concrete examples that touch on equity, diversity, and inclusion and are representative of varying age groups, cultures, climate, geography, etc. will be added after this survey and you will have an opportunity to comment on those in the next round.

6. Please indicate your agreement with the inclusion of the following as key terms:

Mark only one oval per row.

	Strongly disagree	Disagree	Neutral	Agree	Strongly agree
Outdoors	◯	◯	◯	◯	◯
Outdoor play	◯	◯	◯	◯	◯
Outdoor learning	◯	◯	◯	◯	◯
Outdoor teaching	◯	◯	◯	◯	◯

7. Do you have any final comments regarding the key root terms?

____
____
____
____
____

8. Please indicate your agreement with the inclusion of the following as 'Outdoors' sub-terms:

Mark only one oval per row.

	Strongly disagree	Disagree	Neutral	Agree	Strongly agree
Built environment	◯	◯	◯	◯	◯
Green space	◯	◯	◯	◯	◯
Loose parts	◯	◯	◯	◯	◯
Playground	◯	◯	◯	◯	◯
School grounds	◯	◯	◯	◯	◯
Natural environment	◯	◯	◯	◯	◯

9. Please indicate your agreement with the inclusion of the following as 'Outdoor Play' sub-terms:

Mark only one oval per row.

	Strongly disagree	Disagree	Neutral	Agree	Strongly agree
Active play	⬭	⬭	⬭	⬭	⬭
Free play	⬭	⬭	⬭	⬭	⬭
Nature play	⬭	⬭	⬭	⬭	⬭
Risky play	⬭	⬭	⬭	⬭	⬭
Unstructured play	⬭	⬭	⬭	⬭	⬭

10. Please indicate your agreement with the inclusion of the following as 'Outdoor teaching/learning' sub-terms:

Mark only one oval per row.

	Strongly disagree	Disagree	Neutral	Agree	Strongly agree
Environmental education	⬭	⬭	⬭	⬭	⬭
Forest kindergarten	⬭	⬭	⬭	⬭	⬭
Forest school	⬭	⬭	⬭	⬭	⬭
Outdoor classroom	⬭	⬭	⬭	⬭	⬭
Outdoor education	⬭	⬭	⬭	⬭	⬭

11. Do you have any final comments regarding the sub-terms?

12. Please indicate your agreement with the inclusion of the following as other relevant terms:

Mark only one oval per row.

	Strongly disagree	Disagree	Neutral	Agree	Strongly agree
Adventure therapy	◯	◯	◯	◯	◯
'Shinrin yoku' (forest bathing)	◯	◯	◯	◯	◯
Free-flow play	◯	◯	◯	◯	◯
'Friluftsliv	◯	◯	◯	◯	◯
Home range	◯	◯	◯	◯	◯
Garden	◯	◯	◯	◯	◯
Land-based education	◯	◯	◯	◯	◯
Nature	◯	◯	◯	◯	◯
Nature-based (early childhood) education	◯	◯	◯	◯	◯
Nature-based learning	◯	◯	◯	◯	◯
Nature-based preschool	◯	◯	◯	◯	◯
Nature-based recreation	◯	◯	◯	◯	◯
Nature experiences	◯	◯	◯	◯	◯
Nature-enhanced early childhood education	◯	◯	◯	◯	◯
Nature preschool	◯	◯	◯	◯	◯
Social play	◯	◯	◯	◯	◯
Sustainability learning	◯	◯	◯	◯	◯
Outdoor activities	◯	◯	◯	◯	◯
Outdoor early childhood programs (including outdoor	◯	◯	◯	◯	◯

kindergarten, outdoor preschool)					
Outdoor play spaces	⬭	⬭	⬭	⬭	⬭
Outdoor recess/playtime	⬭	⬭	⬭	⬭	⬭
Outdoor recreation	⬭	⬭	⬭	⬭	⬭
Outdoor time	⬭	⬭	⬭	⬭	⬭
Place-based learning	⬭	⬭	⬭	⬭	⬭
Play-based learning	⬭	⬭	⬭	⬭	⬭
Playwork	⬭	⬭	⬭	⬭	⬭
Service learning	⬭	⬭	⬭	⬭	⬭
Wilderness schooling	⬭	⬭	⬭	⬭	⬭
Wilderness therapy/treatment	⬭	⬭	⬭	⬭	⬭

13. Do you have any final comments regarding the other relevant terms?

Additional comments

14. Additional comments?

Thank you for completing the questionnaire!

This content is neither created nor endorsed by Google.

Google Forms

Round 4: PLaTO-Net (Play, Learn, and Teach Outdoors Network) Global Harmonization Project - Consultation Questionnaire

Thank you for your extensive feedback on the PLaTO-Net conceptual model, logo, and key terms related to play, learning, and teaching outdoors. We are now seeking feedback on the definitions of the key terms and sub-terms. The inclusion of terms in this survey was determined based on >75% consensus for inclusion in the previous survey (Round 3).

As a reminder, our aim is to achieve clear, common, harmonized, and widely accepted terminology, taxonomy, and ontology related to play, learning, and teaching outdoors that have utility across all ages, genders, cultures, and abilities, adapted to our contemporary understanding.

We plan to publish the results of our efforts. PLaTO-Net members who participate in this questionnaire and continue to participate in the consensus process will be invited as co-authors. Subsequent correspondence regarding this project will be with those who complete the questionnaire only. For this reason, respondents will not be anonymous, but individual responses will remain confidential.

* Required

1. Email *

____

Part 1: Definitions of the PLaTO (Play, Learn, and Teach Outdoors) Root Terms

Based on the scoping review on terminology of outdoor play, learning, and teaching we conducted over the past year and additional research done by the leadership group, we propose that our project provides definitions for root terms (outdoors, play, education, learning, teaching, outdoor play, outdoor education, outdoor learning, outdoor teaching) and sub-terms based on their frequency and commonality according to our completed scoping review or suggested by the survey respondents in the previous round.

Please see below to learn more about our process of identifying and defining root terms and sub-terms:

1. Systematic coping review: A systematic scoping review was conducted between August 2019 and March 2020. The goal of the scoping review was to objectively identify a comprehensive list of key terms and definitions on playing, learning and teaching in the outdoors. From this review, we identified a total of 428 terms with 984 definitions, representing 35 unique countries from 441 studies.
2. Subcommittee for ontology, taxonomy, and terminology: The goal of this subcommittee work was to select and classify key terms and definitions from the list created based on the scoping review.
3. Three previous surveys: Based on the feedback received from the respondents who participated in the previous three rounds of surveys, we have added terms that are less common but identified as important.
4. All terms included in this survey has received >75% consensus for inclusion in the previous round of survey (Round 3).

For each root term please indicate your level of agreement with its definition.

Note:
1. These root terms are identified as commonly used terms in a scoping review of terminology used in outdoor play, learning, and teaching research.
2. Terms included here do not have hierarchical relationships with each other; rather, they are interconnected and not mutually exclusive.

2. Outdoors: any open-air, wild, natural, or human-made space which may have a temporary or fixed cover (e.g. awning or roof).

Mark only one oval.

	1	2	3	4	5	
Strongly disagree	◯	◯	◯	◯	◯	Strongly agree

3. Play: voluntary engagement in activity that is freely chosen, fun, and driven by intrinsic motivation.

Mark only one oval.

	1	2	3	4	5	
Strongly disagree	◯	◯	◯	◯	◯	Strongly agree

4. Outdoor Play: play that takes place outside in a very broad continuum of spaces that include urban, rural, suburban, and wilderness settings.

Mark only one oval.

	1	2	3	4	5	
Strongly disagree	◯	◯	◯	◯	◯	Strongly agree

5. Teaching: to cause someone to learn or understand something by example or experience.

Mark only one oval.

	1	2	3	4	5	
Strongly disagree	◯	◯	◯	◯	◯	Strongly agree

6. Outdoor Teaching: a pedagogical approach where teaching activities take place outdoors.

Mark only one oval.

	1	2	3	4	5	
Strongly disagree	◯	◯	◯	◯	◯	Strongly agree

7. Learning: the acquisition of knowledge or skills through experience, study, or by being taught.

Mark only one oval.

	1	2	3	4	5	
Strongly disagree	◯	◯	◯	◯	◯	Strongly agree

8. Outdoor Learning: an experiential process of learning by doing, which takes place primarily through exposure to the out-of-doors, usually on school grounds or other nearby locations.

Mark only one oval.

	1	2	3	4	5	
Strongly disagree	⬭	⬭	⬭	⬭	⬭	Strongly agree

9. Education: the process of facilitating learning and the acquisition of knowledge, skills, values, morals, beliefs, and habits on behalf of the learner.

Mark only one oval.

	1	2	3	4	5	
Strongly disagree	⬭	⬭	⬭	⬭	⬭	Strongly agree

10. Outdoor Education: learning about and for the outdoors that usually takes place in an outdoor setting such as an outdoor classroom.

Mark only one oval.

	1	2	3	4	5	
Strongly disagree	⬭	⬭	⬭	⬭	⬭	Strongly agree

11. Do you have any comments or suggested changes for the root terms?

Part 2: Definitions of the PLaTO (Play, Learn, and Teach Outdoors) Sub-terms

Note:
a) These sub-terms are identified as commonly used terms in a scoping review of terminology used in outdoor play, learning, and teaching research or were suggested by survey respondents in a previous round, with >75% consensus for inclusion.
b) Terms included here do not have hierarchical relationships with each other; rather, they are interconnected and not mutually exclusive.

Outdoors Sub-terms

12. Nature: the phenomena of the physical world collectively, including plants, animals, the landscape, and other features and products of the earth.

Mark only one oval.

	1	2	3	4	5	
Strongly disagree	◯	◯	◯	◯	◯	Strongly agree

13. Green space: any vegetated land, an area of grass or trees, that may contain water (e.g., pond, creek) and is set apart for recreational or aesthetic purposes in an otherwise urban environment.

Mark only one oval.

	1	2	3	4	5	
Strongly disagree	◯	◯	◯	◯	◯	Strongly agree

14. Built environment: human-modified physical surroundings (e.g., structures, features, facilities) in which people live, learn, work and play.

Mark only one oval.

	1	2	3	4	5	
Strongly disagree	◯	◯	◯	◯	◯	Strongly agree

15. Natural environment: non-human-made surroundings and conditions in which all living and non-living things exist.

Mark only one oval.

	1	2	3	4	5	
Strongly disagree	◯	◯	◯	◯	◯	Strongly agree

16. Garden: planted, developed, or treated land used to grow vegetables, fruit, herbs, flowers, and other living plants.

Mark only one oval.

	1	2	3	4	5	
Strongly disagree	◯	◯	◯	◯	◯	Strongly agree

17. Loose parts: materials used in play that can be moved, carried, combined, redesigned, lined up, and taken apart and put back together in multiple ways. They are materials with no specific set of directions that can be used alone or combined with other materials.

Mark only one oval.

	1	2	3	4	5	
Strongly disagree	◯	◯	◯	◯	◯	Strongly agree

18. Outdoor play space: an outdoor area within which children can play (e.g., playground, school ground, greenspace).

Mark only one oval.

	1	2	3	4	5	
Strongly disagree	◯	◯	◯	◯	◯	Strongly agree

19. Playground: a piece of land usually equipped with facilities and/or equipment that is used for outdoor play and recreation, especially by children.

Mark only one oval.

	1	2	3	4	5	
Strongly disagree	◯	◯	◯	◯	◯	Strongly agree

20. School ground: proprietary outdoor area adjacent to educational institution buildings.

Mark only one oval.

	1	2	3	4	5	
Strongly disagree	◯	◯	◯	◯	◯	Strongly agree

21. Do you have any comments or suggested changes regarding the Outdoors sub-terms?

Outdoor Play Sub-terms

22. Active play: a form of play that involves gross motor or total body movement.

Mark only one oval.

	1	2	3	4	5	
Strongly disagree	◯	◯	◯	◯	◯	Strongly agree

23. Free play: unstructured, voluntary, child-initiated activity that allows children to develop their imaginations while exploring and experiencing the world around them.

Mark only one oval.

	1	2	3	4	5	
Strongly disagree	⬭	⬭	⬭	⬭	⬭	Strongly agree

24. Nature play: a form of play that happens primarily outside in a natural environment and/or involves play with natural elements and features, such as water and mud, rocks, hills, forests, and natural loose parts, such as sticks, pine cones, leaves, and grass.

Mark only one oval.

	1	2	3	4	5	
Strongly disagree	⬭	⬭	⬭	⬭	⬭	Strongly agree

25. Risky play: a thrilling and exciting form of play that involves uncertainty, unpredictability, and risk of injury.

Mark only one oval.

	1	2	3	4	5	
Strongly disagree	⬭	⬭	⬭	⬭	⬭	Strongly agree

26. Social play: a form of play that involves interacting with others for games, activities, or sport for fun.

Mark only one oval.

	1	2	3	4	5	
Strongly disagree	⬭	⬭	⬭	⬭	⬭	Strongly agree

27. Unstructured play: a form of play that is not initiated in an organized, planned or formal way, and instead places emphasis on self-directed, emergent aspects of play that is not externally directed.

Mark only one oval.

	1	2	3	4	5	
Strongly disagree	⬭	⬭	⬭	⬭	⬭	Strongly agree

28. Outdoor activity: leisure, recreational, or health-enhancing activity engaged in the outdoors.

Mark only one oval.

	1	2	3	4	5	
Strongly disagree	⬭	⬭	⬭	⬭	⬭	Strongly agree

29. Outdoor recess/playtime: blocks of unstructured, regularly scheduled outdoor periods, where children freely choose activities and playmates during school hours.

Mark only one oval.

	1	2	3	4	5	
Strongly disagree	◯	◯	◯	◯	◯	Strongly agree

30. Outdoor recreation: leisure pursuit engaged in the outdoors.

Mark only one oval.

	1	2	3	4	5	
Strongly disagree	◯	◯	◯	◯	◯	Strongly agree

31. Outdoor time: time spent outdoors.

Mark only one oval.

	1	2	3	4	5	
Strongly disagree	◯	◯	◯	◯	◯	Strongly agree

32. Nature-based recreation: leisure pursuits in natural settings or involving elements of nature in some direct way (e.g., terrain, plants, wildlife, water).

Mark only one oval.

	1	2	3	4	5	
Strongly disagree	◯	◯	◯	◯	◯	Strongly agree

33. Do you have any comments or suggested changes regarding the Outdoor Play sub-terms?

Outdoor Education Sub-terms

34. Environmental education: a learning process aimed at increasing knowledge, awareness, and appreciation of the environment and developing skills for responsible environmental stewardship.

Mark only one oval.

	1	2	3	4	5	
Strongly disagree	◯	◯	◯	◯	◯	Strongly agree

35. Land-based education: an Indigenous approach to learning that recognizes a deep connection and relationship between people and the land.

Mark only one oval.

	1	2	3	4	5	
Strongly disagree	◯	◯	◯	◯	◯	Strongly agree

36. Nature-based (early childhood) education: an umbrella term that encompasses different types of education, including nature-based preschool and kindergarten and forest kindergartens, and denotes education that uses outdoor settings as a context for delivery of curricular lessons.

Mark only one oval.

	1	2	3	4	5	
Strongly disagree	◯	◯	◯	◯	◯	Strongly agree

37. Forest kindergarten: a kindergarten that is based around child-centred learning through play and offers young children frequent and regular outdoor play opportunities in a natural setting that is often in woodlands and is held in almost all weather throughout the year.

Mark only one oval.

	1	2	3	4	5	
Strongly disagree	◯	◯	◯	◯	◯	Strongly agree

38. Forest school: a school, based in the outdoors, that adopts an educational approach that includes regular and repeated access to natural space and child-directed, emergent and inquiry-based learning.

Mark only one oval.

	1	2	3	4	5	
Strongly disagree	◯	◯	◯	◯	◯	Strongly agree

39. Outdoor classroom: an outdoor setting where education and learning takes place.

Mark only one oval.

	1	2	3	4	5	
Strongly disagree	◯	◯	◯	◯	◯	Strongly agree

40. Outdoor early childhood program (including outdoor kindergarten, outdoor preschool): a child-centred nature-based early learning program for children aged 2.5-6 years that emphasizes experiences in the outdoors, nearby nature, contact with nature, and ecological experiences, such as encounters with plants, animals, insects, and outdoor seasonal climates.

Mark only one oval.

	1	2	3	4	5	
Strongly disagree	◯	◯	◯	◯	◯	Strongly agree

41. Nature-based preschool: an early childhood program for preschool-aged children, with at least 25-50% of the class day held outside each day, nature as the driving theme of the curriculum, and nature being infused into the indoor spaces.

Mark only one oval.

	1	2	3	4	5	
Strongly disagree	◯	◯	◯	◯	◯	Strongly agree

42. Learning for sustainability: a cross-curricular approach to life and learning which enables learners, educators, schools, and their wider communities to build a socially just, sustainable, and equitable society.

Mark only one oval.

	1	2	3	4	5	
Strongly disagree	◯	◯	◯	◯	◯	Strongly agree

43. Nature-based learning: learning that occurs in natural settings or where elements of nature such as plants, water, and animals have been brought into built environments such as classrooms.

Mark only one oval.

	1	2	3	4	5	
Strongly disagree	◯	◯	◯	◯	◯	Strongly agree

44. Place-based learning: learning that considers the importance of connecting learners with their community by anchoring pedagogy within the context of the local, natural, and social ecosystems.

Mark only one oval.

	1	2	3	4	5	
Strongly disagree	◯	◯	◯	◯	◯	Strongly agree

45. Do you have any comments or suggested changes regarding the Outdoor Education sub-terms?

Additional comments

46. Additional comments?

Thank you for completing the questionnaire!

This content is neither created nor endorsed by Google.

Google Forms
