# Supplementary material for: Play, Learn, and Teach Outdoors—Network (PLaTO-Net): terminology, taxonomy, and ontology
Source: Int J Behav Nutr Phys Act. 2022 Jun 15;19:66. doi: 10.1186/s12966-022-01294-0 (PMC9199154; doi:10.1186/s12966-022-01294-0)
Supplement: Supplementary file 5 — Additional file 5. [file 12966_2022_1294_MOESM5_ESM.docx]

**Supplementary Table 1. List of terms and definitions/ descriptions.**

Table of Contents

1. [Play](#Play)
2. [Outdoor play/time/playtime](#OutdoorPlayTime)
   1. [Outdoor play](#Outdoorplay)
   2. [Outdoor time](#Outdoortime)
   3. [Outdoor playtime](#outdoorplaytime2)
3. [Education/learning/School](#EducationLearningSchool)
   1. [Learning](#learning)
   2. [Education](#Education)
   3. [School](#Schools)
4. [Curriculum/Pedagogy/Program](#CurriculumPedagogyProgram)
5. [Therapy](#Therapy)
6. [Activity/adventure/expedition/recreation](#ActAdvExpRec)
   1. [Activities](#Activities)
   2. [Adventure](#Adventure)
   3. [Expedition](#Expedition)
   4. [Recreation](#Recreation)
7. [Environment/Location/Space](#EnvrionLocationSpace)
   1. [General Spaces/environment](#GeneralSpacesEnvironment)
   2. [Home environment](#HomeEnvironment)
   3. [Learning environment](#LearningEnvironment)
   4. [Nature environment](#NatureEnvironment)
   5. [School environment](#SchoolEnvironment)
   6. [Playground](#Playground)
   7. [Play spaces/environment](#PlayEnvironmentSpaces)
8. [Approach/Model/Theory](#ApproachModelTheory)
9. [Outcome/Component](#Outcome)
10. [Element/Feature](#ElementFeature)

| # | Term | | Definitions/Descriptions | Country/Age of children | Reference |
| --- | --- | --- | --- | --- | --- |
| **Play** | | | 1. "...kids tinkering and experimenting with materials and content or engaging in other hands-on, experiential learning opportunities freely…" pg 31 2. "Play differs from recreational activity: while both are voluntary, play is directed by children whereas recreation takes the forms of activities, sport, and games directed by adults." "Children’s play is generally defined by agency which is understood in terms of the child’s freedom to choose play and the child’s direction or control of play." 3. "Play serves as a developmental strategy to respond to a period of immaturity and need for protection, allowing for opportunities to interact with different stimulus and learn by trials and errors. As a natural and self-directed activity, play promotes cognitive, physical, social and emotional well-being, offering the necessary conditions for children to overcome personal barriers and evolve in skills and knowledge." pg 289 4. "including both physically active as well as more sedentary behaviours" pg 5 5. "Play is a tool which allows children to ‘assimilate’ or integrate stimuli and information from the external world into their existing schemas. Further, learning through play incorporates a significant social and environmental element, where children interact with others and their setting to modify existing and adopt new ways of thinking. While play, as a cognitive-behavioural construct, is difficult to define, it represents a multidimensional behaviour, often falling on a continuum between distinct play (free-play) and non-play (work/adult-directed) behaviours. However, it is marked by active engagement with the environment and other social actors, and can thus be considered a principle tool for experiential learning in childhood." pg 22 6. Common words used by participants in a survey to define 'play' include: "‘shared’; ‘fun’; ‘free’; and ‘informal experience’" pg 37; "Pellegrini (1995) says that play is an activity done for its own sake which is flexible and fun. Play can be contrasted with exploration which may lead to play, work which has a goal, or games which are organised with the aim of winning." pg 37 (also exploration, games) 7. " Key characteristics of play are that it is fun and also a foundation for building friendships." pg 25 8. "Play is an essential part of every child's life and vital to the processes of human development. It provides the mechanism for children to explore the world around them and the medium through which skills are developed and practiced. It is essential for physical, emotional, and spiritual growth, intellectual and educational development, and acquiring social and behavioural skill." pp. 260; "Play in these artificial environments can lack the diversity of learning experiences provided by their natural counterpart. The equipment is fized and cannot be manipulated and in most cases has limited value for exploration or imaginary play compared to more natural settings. Playgrounds also tend to be age specific..." pp.265; "Play is not confined to designated areas, but children play anywhere and everywhere." pp. 266 9. "Play is a series of movements and pauses that flows across a space or environment." pp. 16 10. "Children's play is an ideal time for assessing and fostering social skills." pp. 27 11. (Children’s play) "is self-motivated, and children engage in play for their own sake of enjoyment." pg.477 12. "… represents activities that were 'owned' by children and was described in terms of fun, spontaneity, interactions with friends and the absence if competition or aggression" pp.4 13. ."Play is a way for students to create unregulated strategies to deal with reality in a world where so much is regulated." pp.244 14. "… on the importance of play in overall development: play is seen as a crucial part of children's physical, cognitive, emotional and social development and it also encourages creativity and learning. " pp. 39 (Kieff 2000, Meadows, 92, Vygotsky 78, Wood 2005)   "Play covers a broad category of activities - social games, pretence games, playing with toys, and unspecified indoor and outdoor play -and it can be stimulated in different contexts." pp. 39 (Pellegrini 99, 2005, Wood 2005)   1. "Play, a dynamic, active and constructive behaviour - is an essential and integral part of all children's heathy growth, development and learning across all ages, domains and cultures." pp. 33; "Play enhances learning, and development for children of all ages, cultures and domains." pp. 34 2. "Play is defined as a behaviour that leads to all phases of development within the cultural contect in which players interact." pp. 47 3. " Play has the following characteristics: pleasurable and enjoyable; no goal imposed from the outside; spontaneous and voluntary; involving some active engagement on the part of the player; having certain systematic relations to what is not play; it can contrasted to non-play." pp.348   "Play should involve attention to the means over the end product of the action or activity; another is that it should be ﬂexible and changing; and the third is that it has a non-literal orientation." pp. 348-49  "In play a child always behaves beyond his average age, above his daily behaviour; in play it is as though he were a head taller than himself. As in the focus of a magnifying glass, play contains all developmental tendencies in a condensed form and is itself a major source of development." pp. 349   1. "Play is a volunteer and purposeless activity, driven by intrinsic motivation (Johnson et al., 2012; Lillemyr, 2009). " p.259 2. “The behaviors and activities that children engage in during childhood are broadly defined as ‘play.’1,2 Play is multidimensional, consisting of behavioral, motivational, and contextual components.3,4 Further, play is fun, enjoyable, flexible and spontaneous, encompasses a wide range of self-chosen activities stimulated by own ideas and interests, and is minimally constrained by adult demands.1,3 Play makes a unique contribution to children’s social, creative, physical, and emotional development,5–7 and is positively linked to self-perceptions,8 self-esteem,9 resilience,10 and conflict management skills.6 During school time play occurs during recess or, as known in the UK, playtime." p.406 3. "However, the definition of play that applies best to our study can already be found in the work of Frost (1979). The author describes play with a play–work continuum and characterizes play as active, spontaneous, fun, purposeless, self-initiated and serious (Frost, 1979, p. 21)." p.207 4. "Play is an integral part of a child’s learning experience"; “Play provides a vehicle for children to both develop and demonstrate knowledge, skills, concepts and dispositions (Dempsey & Frost 1993; Isenberg & Quisenberry, 2002).; “Play provides a non-threatening context for children to learn about their world and gain skills necessary for adult life (Bjorklund, 1997; Bruner, 1972).”; “play as a ‘scaffold for development, a vehicle for increasing neural structures, and a means by which all children practice skills they will need in later life’ (Isenberg & Quisenberry, 2002, p. 33).” 5. "Play involves unstructured activity that is freely entered into and intrinsically rewarding" p.21 6. "play is an activity that serves no apparent purpose, the activity being more important than its ends’ (Sandseter, 2009, p. 3)." p243 7. " Any spontaneous activities, in which the children engaged to enjoy and occupy themselves, were considered play, as defined by Rigby and Rodger (2006)." p128 8. "Benefits of play: (1) development of motor skills; (2) sharpening of the sense; (3) development of empathy; (4) understanding and practice of sharing, turn taking, and other peer cooperation skills; (5) increasing control of compulsive actions and learning to accept delayed gratification; (6) building ordering and sequencing skills; (7) increasing vocabulary; (8) increasing concentration skills; (9) learning to navigate assigned roles; (10) development of capacity to be flexible; (11) expansion of imagination, creativity, and curiousity; (12) reducing aggression." pp.68 9. "Play requires specific conditions of safety and psychological security that are essential for the child to engage in relaxed, open-ended, and exploratory behaviors. Play includes exploratory behaviors that involve manipulation of objects, toys, and other materials, and this exploratory nature of play often precedes actual focused play behavior. Play is an important evolutionary behavior that is essential for healthy development to occur across all areas: social, cognitive, language, physical, and creative. Play is behavior that sustains the healthy development of the individual and the larger sociocultural fabric of society and reflects the contexts in which the child lives (home, community, and the larger society)." pg. 5 10. “engaging in an activity for enjoyment and recreation rather than a serious or practical purpose.”p.157 11. "Play is intentional and involves children acting with a purpose and goal for personal learning as they actively explore, discover, imagine and interact with objects, people and their natural world. Educators have a role in play and can foster children's holistic development by intentionally sustaining children's thinking and involvement in play-based learning environments." p.30 12. "The very definition of play is that children initiate an activity they are interested in doing and seek ways to be inventive, creative and exploratory." pp. 164 | 1. USA, NR 2. Canada, NA 3. Portugal, 4 months-10 years 4. UK, 10-11 years 5. UK, 4-9 years 6. New Zealand, NA 7. New Zealand, NA 8. NA, NA 9. NA, NA 10. NA, NA 11. Canada, NA 12. Canada, 6-12 years 13. Australia, 5-12 years 14. Finland, NA 15. NA, NA 16. Japan/South Korea, 5 years 17. NA, NA 18. Norway, 1-3 years 19. UK, 7-11 years 20. USA, NA 21. Germany 2013 22. USA, NA 23. UK, 4-5 years 24. Uganda, infancy-pre pubescent 25. NA, NA 26. UK, NA 27. Multiple (review), 2-6 years 28. UK, 8-11 years 29. NA, NA | 1. Bradshaw 2018 2. Alden 2019 3. Bento 2018 4. Brockman 2011 5. Coates 2019 6. Couper 2011 7. Couper 2013 8. Freeman 1995 9. Guddemi 1992 10. Hartle 1993 11. Herrington 2015 12. Holt 2008 13. Hyndman 2018 14. Hyvonen 2007 15. Isenberg 2002 16. Izumi-Taylor 2017 17. Jordan 2003 18. Kleppe 2018 19. Knowles 2013 20. Ko 2007 21. Little 2008 22. Luchs 2013 23. Lyons 2005 24. McArdle 2013 25. Njelesani 2011 Oliver 2002 26. Stegelin 2005 27. Truelove 2017 28. Turtle 2015 29. Ward 1987 |
|  | Active play | | 1. "...outdoor play as a strategy to increase physical activity, and as such, promotes active play..." 2. "...vigorous form of play termed ‘physical activity play’, or ‘active play’." pg 2   "Active play may involve symbolic activity or games with rules; the activity may be social or solitary, but the distinguishing features are a playful context, combined with activity that is significantly above resting metabolic rate. Active play tends to occur sporadically, with frequent rest periods, which makes it difficult to record." pg 2  "...“unstructured physical activity which takes place outdoors in a child’s free time”. A characteristic of outdoor active play in comparison to indoor active play, is that it often takes place in the absence of parents, providing opportunities for children to ‘make it on their own’ and develop a sense of independence." pg 2; Examples given by children: running around, playing tag, kick around a football, jumping, mucking about, messing around, ride bikes, and playing with a dog. pg 3   1. "...activities that involve moderate to high levels of physical activity in the context of play with an energy expenditure above the physiological (i.e. muscular and cardiorespiratory) norm" pg 307 2. "Physically active play can be understood as activities that involve moderate to high levels of physical activity in a context of play with energy expenditure above the physiological (muscular, cardiorespiratory) norm." pg 258 3. "Children experience active play through lifting, pushing, and pulling." pg 2 4. "[when a} child play outdoors; in the following locations?” The seven locations included “the yard at your home,” “the yard at someone else’s home (friend, neighbor or relative),” “the street or cul-de-sac your home is on,” “other streets or cul-de-sacs,” “parks and playgrounds outside of school hours,” “school grounds outside of school hours,” and “other places where your child can be active (e.g., field, parking lot, forested area).” pg.3."e.g., tag, road hockey, hide and seek. In the context of active play, encouragement could consist of parents giving verbal cues for their child to engage in play such as telling them to go to the park or to ride their bicycle with friends in the neighborhood." pg.2 5. "During playtime, students can stay fit, learn self-control and compromise, enliven their imaginations, enhance independent thinking, develop self-confidence, and experience accomplishment by conquering physical challenges." 6. " Active play involves physical activity that produces moderate to vigorous spurts of energy that can increase a child’s heart rate. The duration and intensity of active play changes as children develop." pg. 477 7. "Active play is regarded as the diverse range of unstructured, spontaneous physical activities and behaviour that children engage in." pp.56; "Children have limited access to opportunities for active play in areas other than playgrounds during school breaks" pp. 57 8. "‘a form of gross motor or total body movement in which young children exert energy in a freely chosen, fun and unstructured manner’. It is often engaged in outdoors, which is associated with higher habitual physical activity and MVPA levels and is suggested to be one of the factors explaining the higher levels of physical activity in low–middle-income countries compared to high-income countries." pp.2 9. "The children’s creative play included coming up with new ways of using materials and making and reshaping toys." p.128 10. "Questions assessing the frequency (≤once/mo vs ≥2 times/mo) of equipment use for indoor and outdoor activities included use of active video games (AVG); bicycles; skating equipment (eg, skateboards, roller blades); and fixed play equipment (eg, jungle gym). The frequency of physical activity participation in various indoor (home, indoor pool, and recreation center) and outdoor (residential yard, residential driveway, trails, playing fields, public playgrounds, and outdoor pools) locations was also assessed" p.581-582 11. "Based on the summation of extracted data, it is apparent that many of the reviewed studies share similarities with regard to how active play was defined/described (see Table 4 for common themes/concepts). Most frequently, active play was defined as a form of physical activity (n = 13). Definitions included terms such as: gross motor movement, unstructured activity, freely chosen, and occurs outside. Examples of active play behaviors included: swinging, climbing, pulling, balancing, jumping, rolling, running, and skipping. p.162 | 1. Canada, NA 2. UK, 10-11 years 3. Norway, 3-5 years 4. Norway, 1-6 years   22-63 years (teachers / assistants)   1. Australia, 5-7 years 2. Canada, 7-12 years 3. USA, NA 4. Canada, NA 5. NA, NA 6. Scotland, 3-7 years 7. Uganda, infancy-pre pubescent 8. USA, 7-12 years 9. Multiple (review), 2-6 years | 1. Alden 2019 2. Brockman 2011 3. Bjorgen 2016 4. Bjorgen 2015 5. Bundy 2011 6. Ferrao 2015 7. Hall 2007 8. Herrington 2015 9. Hyndman 2015 10. Johnstone 2019 11. Njelesani 2011 12. Puette 2019 13. Truelove 2017 |
|  | Active free play | | 1. "Active free play usually occurs outdoors and has been associated with increased moderate- to vigorous-intensity physical activity (MVPA)." pg 146; "Aside from time in daycare and preschool, on a typical weekday, how much time does your child spend outside in “unstructured free play?”’ Parents were instructed to provide their response in minutes per day." pg 146 2. “a form of gross motor or total body movement in which young children exert energy in a freely chosen, fun, and unstructured manner” p 2 | 1. Canada, 1-5 years 2. Italy, 4-5 years | 1. Carsely 2017 2. Tortilla 2019 |
|  | Associative play | | 1. "Child and play partner play the same game and converse but do not work together. If they are working together, there are not assigned roles. The children must be playing with each other not just near each other." p.6 | 1. USA, 3-15 years | 1. Miller 2017 |
|  | Child’s directed play | | 1. "...distinguish play from adult-directed activities and organized sport" | 1. Canada, NA | 1. Alden 2019 |
|  | Construction play | | 1. Experimentation with materials within the natural environment, such as sticks, leaves, stones, dirt and sand, can lead to construction play with these natural materials. Construction may also occur with manufactured material provided outdoors, for example carpentry material, small and large blocks, boxes, crates, and planks. " pg 39; "constructions, which may represent dams, rivers, cars, trains, space ships, houses, or forts, often become the focus of sociodramatic play themes." pg 39 2. "The random movements of practie play take on direction, as in building with blocks, manipulative activities and art projects." pp.18 3. (constructive play)"Characterized by: (1) safe, wholesome and non-violent; (2) it stimulates children to develop skills and positive relationships; (3) it inspires children to learn more about themselves and the world around them; (4) it encourages and enables children to fully realize their potential; (5) it encourages creativity; (6) it develops a child's personality; (6) it makes learning fun." pp. 67 4. (constructive play) "Involves using materials in a purposeful way in order to create. This includeas with sand, constructive art, and using boxes, crates, and other loose materials to build structures." pp. 20 | 1. Australia, NA 2. NA, NA 3. NA, NA 4. NA, NA | 1. Davies 1996 2. Guddemi 1992 3. Oliver 2002 4. Vaughn 1990 |
|  | Cooperative play | | 1. "Child and play partner play the same game, converse, and work together using assigned roles." p.6 | 1. USA, 3-15 years | 1. Miller 2017 |
|  | Creative(-based) play | | 1. "...engaging in creative endeavours, including: making things from natural materials; using saws, knives and other tools to sculpt wood; and building shelters. Being creative involved engaging in activities which stretched children’s imaginative abilities, challenging them physically and cognitively" pg 30 2. . "self-managed play in natural landscapes." pg. 318; "encourage[s] experimentation, discovery and experience of self and the world, through self-activated play (Steinsholt, 2014)" 3. "…enables children to test their skills, try new ideas, and seek challenges that cannot be duplicated in other environments." pg.85 4. "Creative play is vital for stimulating flexibility, curiosity, improvising, adaptation to change, learning and solving problems." pp. 244 5. "The children’s creative play included coming up with new ways of using materials and making and reshaping toys." p.128 | 1. UK, 4-9 years 2. Norway, NA 3. NA, NA 4. Australia, 5-12 years 5. Uganda, infancy-pre pubescent | 1. Coates 2019 2. Gurholt 2016 3. Hendy 2000 4. Hyndman 2018 5. Njelesani 2011 |
|  | Discovery play | | 1. "Children love to discover new materials, equipment, words, and feelings. Incorporating mathematics, literacy, science, and other curricular goals outside encourages discovery play. For instance, life science gives children a chance to closely observe, care for, and enjoy living things. " p.15 | 1. USA, early childhood | 1. Oliver 2013 |
|  | Dramatic play | | 1. Examples: "For dramatic play try setting up a car wash for riding toys, including brushes, buckets of both soapy and clear water, plastic spray bottles, chamois, sponges, squeegees, and old towels. Another dramatic-play area most suited to the outdoors is a laundry for doll clothes. Give everything a good washing in a big tub of warm, sudsy water, a rinse in clear water, and a hang-up with clothespins to dry in the sun. On another day try setting up a paint shop, using buckets of water and wide brushes to "paint" the sidewalk, playground, and fence. Try cleaning up spilled "paint" with squeegees" pg 31 2. "Dramatic play is key to success in later formal education. The young child who can readily manipulate symbols in dramatic play is much more likely to accept and effectively use the arbitrary symbol systems of mathematics and written language." pg.88 3. " Pretend or dramatic play, a higher order play behavior (Maxwell et al., 2008), provides an important connection to performance on cognitive tasks including language and scholastic achievement. Language is more complex as children experiment with humor, rhyme, negotiation" p.29 4. “Children are born with a natural curiosity about the world. It is through discovery and pretend play that they can learn about themselves, their peers, and their world." P.14-15 5. "Supported by provision of enclosed structures for buildings and varying dramatic play props" pp.20 | 1. NA (maybe USA), NA 2. USA, NA 3. USA, NA 4. USA< early childhood 5. NA, NA | 1. Crosser 1994 2. Henniger 1994 3. McClintic 2015 4. Olsen 2013 5. Vaughn 1990 |
|  | Group play | | 1. Rubin (2001) considered group play to be the category involving the highest degree of social participation, since it refers to several children engaging in the same play activity with a shared objective. This notion of group play encompasses the categories of associative and cooperative play that were described previously by Parten (1932). 2. "Requires two or more children to work together." pp.20 | 1. Spain, 3-6 years 2. NA, NA | 1. Larrea 2019 2. Vaughn 1990 |
|  | Guided play | | 1. "Guided play retains many characteristics of free play, especially the enjoyable nature and the fact that it is child driven, but adds an additional focus on a targeted learning goal. Guided play involves children exploring their environment with adults through interactions focused on implicit learning goals | 1. USA, 8-13 years | 1. Hassinger-Das 2018 |
|  | Free play | | 1. "The Parten scale, adapted by Ballard (1981), categorizes children’s free play in accordance with Piaget’s developmental theory, and defines six categories of play: U unoccupied behaviour: score 1; S solitary independent play: score 2; O onlooker: score 3; P parallel activity: score 4; A associative play: score 5; C cooperative or organized supplementary play: score 6." 2. Play that is "protected from too much adult control and interruption". 3. "Free play is often used to emphasize a child’s choice in play, although the type of adult engagement and relative number of play options influence children’s perceptions of their choices across home, school and recreational play environments." 4. "Free play is when children play alone or with other children without adults initiating, facilitating or organising the play." pg 6 5. "free play is intrinsically motivated and not provoked by instrumental goal-directed behaviour. It is a goal in itself and lacks external rules and   structure. Thus, activities such as organized sports would not be considered free play." pg 3136   1. "...essential to define, inside the so-called ‘free play’, the conditions of the action in their twofold meaning: positive, because, to some extent, they can in fact be defined as the ensemble of those elements that enable to carry out the activity, and which constitute a setting made of times, spaces, tools, and of educational attentions; but we must also recall that the same elements restrain the activity of play, that they condition it in a negative way because they offer guidance that is strictly dependent upon rules (i.e. limitations) determined by the educators (teachers, assistants, etc.). The overall analysis of the various situations we have observed conveys the impression that we should not talk about ‘free play’ inside schools, but we should use a more correct definition: spontaneous play; even though freedom of play is assured, at least at the level of initial impulse (so that spontaneity is assured), it nevertheless seems to be extremely limited in the choices that are conditioned by the context to the point that we could here use the concept of probation." pg 72-73 2. "Free choice during free play enhances children’s decision-making skills and helps children discover their own areas of interest leading them to develop individual passions they wish to pursue." pg 10 3. "..."free play"—basically, unstructured time in which kids create their own scenarios, cooperate, and learn to assess risk" pg 33 4. "The merit of the Junkyard was viewed as providing an environment in which children were free to play as they choose, with minimal intervention on the part of adults. Children could play on their own or with others in any way that they chose – play did not have to adapt to any adult criterion other than not to violate the safety of the self or others." pg. 361 5. "Free play, whether with objects, pretend, or physical, is fun and voluntary, involves active engagement, without extrinsic goals, and often incorporates make-believe." pg. 3 6. (unstructured free play) Unstructured play or free play can occur anywhere. It is not planned or led by adults, but is spontaneous and directed by the children themselves." pg. 477 7. "Some believe free play in outdoor environments is the best way to promote physical development." pp.203 8. "Free play in outdoor environments is the best way to permit physical development when children are guided to freely access a broad range of physically challenging equipment and activities" pp. 55 (Bullard, 2012; Frost 2004) 9. (free play schedule/free-flow routine) "… survives provide children with opportunities to access outdoor play areas (whenever a structured activity is not in place), allowing flexible access between the indoor and outdoor environments to increase total outdoor free play time." pp.2 | 1. New Zealand, 3-7 years 2. Norway, NA 3. Canada, NA 4. Norway, 3-5 years 5. Multiple, NA 6. NA, NA 7. USA, Kinder to 4^th^ Grade 8. USA, NA 9. Israel, Kindergarten 10. USA, 8-13 years 11. Canada, NA 12. China, 3-6 years 13. China, Kindergarten 14. Australia, NA | 1. Anderson 2004 2. Aasen 2009 3. Alden 2019 4. Bjorgen 2016 5. Brussoni 2012 6. Ceciliani 2013 7. Clarke 2018 8. Clendaniel 2009 9. Golden 2018 10. Hassinger-Das 2018 11. Herrington 2015 12. Hu 2014 13. Hu 2015 14. Jones 2019 |
|  | Free explorative play | | 1. "Pretty et al. (2009) also argue that greater opportunities for free explorative play exist when natural environments are varied and changeable." pg 560 | 1. UK, 3-5 years | 1. Canning 2010 |
|  | Functional play | | 1. "Involves practice and repetition of gross motor activities such as climbing, swinging and sliding. This type of play is supported by much of the traditional playground equipment." pp. 20 | 1. NA, NA | 1. Vaughn 1990 |
|  | Group play | | 1. " Group play was defined as duration of activity when the participant engaged with the group, using the same types of equipment or toys as peers. For a detailed explanation of the paradigm, see previous reports." pg 337 2. Rubin (2001) considered group play to be the category involving the highest degree of social participation, since it refers to several children engaging in the same play activity with a shared objective. This notion of group play encompasses the categories of associative and cooperative play that were described previously by Parten (1932). | 1. USA, 8-14 years 2. Spain, 3-6 years | 1. Corbett 2017 2. Larrea 2019 |
|  | Imaginative play | | 1. "creation of new worlds" pg 30   Example: "That’s when me and Alex were getting the seats for fisherman camping. We are fishing to get a hot fish. (Interviewer: Fishing to get hot fish, and where were the fish?) In the sea, because there was a sea there; not a real one, a fake one [made out of] wood. (Interviewer: And what were the fish?) Some sticks and I got a swordfish!" pg 31   1. Example: "the construction of a shelter leaning on another tree and, inside, two students sat with large limestone rocks, grinding the limestone down to powder to (when asked) create a ‘potion’" pg 302 | 1. UK, 4-9 years 2. Australia, 1-12 years | 1. Coates 2019 2. Cumming 2015 |
|  | Indoor play | | 1. "In indoor settings, children frequently engage with conversations of peers and participated in dramatic play or games with toys." pp 1612 | 1. Japan, 3-5 years | 1. Hirose 2012 |
|  | Loose parts play | | 1. Loose parts on playgrounds "[fosters]...Creative, dynamic and...imaginative development". pg.87 | 1. Australia, 5-12 years | 1. Engelen 2018 |
|  | Nature play | | 1. "...is play in an environment that includes landform, vegetation/trees, natural materials (e.g. stone, water, sand, bark, moss, leaves, mud, logs, fruit, sticks) and moving/loose parts." 2. "...is play in an environment that includes landform, vegetation/trees, natural materials (e.g. stone, water, sand, bark, moss, leaves, mud, logs, fruit, sticks) and moving/loose parts." 3. "...is play in an environment that includes landform, vegetation/trees, natural materials (e.g. stone, water, sand, bark, moss, leaves, mud, logs, fruit, sticks) and moving/loose parts." (natural play) "...is play in an environment that includes landform, vegetation/trees, natural materials (e.g. stone, water, sand, bark, moss, leaves, mud, logs, fruit, sticks) and moving/loose parts." p.174 4. "Research shows that experiences in natural settings provide multiple benefits to young children including increased physical activity, reduced obesity, and improved concentration and enhanced social skills." pp. 4; "Nature play provides rich fodder for young imaginations, growing vocabularies, and budding social skills as children negotiate themes and scenarios and settle disputes. " pp. 6 5. (natural play) "Natural play will automatically occur when vegetation and other components of the natural world are incorporated into the design of the outdoor area. Trees and vegetation should be present in all early childhood programs. Moore et al. (1987) indicated landscaping can satisfy the need for shade, wildlife habitat, and sensory variety. Further, it has been noted that plants provide moods, offer seasonal interests, secret places to play and experience with, color, smells, sounds, and natural loose parts such as logs, stumps, stickers, or branches (Keller, 2008). Vegetation and other natural components should be selected that fit the mission and values of the program." p.14 6. (also wild play) "Structured programmes designed to facilitate children’s play in natural environments." p.8 7. (natural play) "whereby children explore and enjoy the natural environment through their freely chosen play activities" pg.2 | 1. Australia, 2-6 years 2. Singapore, NA 3. USA, 4-5 years 4. NA, NA 5. USA, early childhood 6. Multiple (Delphi), NA 7. UK, 7-9 years | 1. Dowdell 2011 2. Ebbeck 2019 3. Zamani, 2017 4. Jacobi-Vessels 2013 5. Olsen 2013 6. Shanahan 2019 7. Trapasso 2018 |
|  | Non-active play | | 1. "...sedentary activities, many of which involved the use of computers or games consoles..." pg 3   Examples given by children: video games, computer games, playing on laptop, playing music, board games, and role play. | 1. UK, 10-11 years | 1. Brockman 2011 |
|  | Non-play | | 1. "work/adult-directed" pg 22 | 1. UK, 4-9 years | 1. Coates 2019 |
|  | Object play | | 1. "e.g., manipulating objects, toys" pg 3136 | 1. Multiple, NA | 1. Brussoni 2012 |
|  | Open-ended play | | 1. "Open-ended play allows children to express themselves in play freely and in ways that are not bound by preset limitations. Playing with open-ended materials with multiple uses and limitless possibilities, such as sticks, wet earth, plants and trees, allows for imaginative play. There are no rules to follow, no expectations and no specific problems to solve, and there is no pressure to produce a finished product when engaging freely in open-ended play." p.243 | 1. UK, 4-5 years | 1. McArdle 2013 |
|  | Parallel play | | 1. Parallel play is when a child plays adjacent to another child or children in a similar activity, but does so independently. | 1. Spain, 3-6 years | 1. Larrea 2019 |
|  | Pedagogical play | | 1. "Consists of multiple forms of intentional educative activity, all of which off potential meaningful opportunities to engage children in developing awareness and understanding of their world" pp. 90 | 1. USA, 18 months-6 years | 1. Hunter 2017 |
|  | Physical activity play | | 1. "e.g., exercise play, rough-and-tumble play" pg 3136 2. (physically active play) "Active free play usually occurs outdoors and has been associated with increased moderate- to vigorous-intensity physical activity (MVPA)." pg 146; "Aside from time in daycare and preschool, on a typical weekday, how much time does your child spend outside in “unstructured free play?”’ Parents were instructed to provide their response in minutes per day." pg 146 3. "PAP is a 'playful context omvined with dimension of physical rigor.' Thus play is visibile in both PAP and defintion. In contrast, play is invisible in both PE and definition as 'planned, structured learning experiences in curriculum time." pp. 478 | 1. Multiple, NA 2. Canada, 1-5 years 3. Singapore, NA | 1. Brussoni 2012 2. Carsley 2017 3. Hussain 2018 |
|  | Physical play | | 1. "Pellegrini and Smith, analysing the developmental functions of physical play, defined it as a playful context combined with a dimension of physical vigour; they suggest that forms of physical activity play primarily serve immediate developmental functions." pg 69 2. “Stephenson (1998) describes three types of physical play that preschool children typically engage in outdoors. First is play which might be described as coaching, whereby children seek teachers’ assistance to either learn specific physical skills or attempt a particular physical activity. The second type of play combines aspects of physical play and dramatic play—physical activity incorporated with role-playing in dramatic play episodes. Chasing games, such as ‘What’s the time, Mr Wolf?’, are also included in this category. The third type of play relates to the children’s obvious desire to physically challenge themselves and extend their skills by ‘riding … the bikes very fast, climbing around the outside of the fort, running across the challenge course, swinging very high, dangling off the edge of the fixed slide and dropping to the ground’ (Stephenson, 1998, p. 127).” | 1. NA, NA 2. NA, children | 1. Ceciliani 2013 2. Little 2008 |
|  | Play for learning | | 1. "...is self-directed, creative, innovative, collaborative, and draws on multiple senses; the young learner is viewed as naturally intelligent, eager to learn and experiment, resourceful, active, flexible of mind, creative, willing and able to recognise and seize opportunities for learning." pg. 365 | 1. Israel, Kindergarten | 1. Golden 2018 |
|  | Play together | | 1. "Playing together is when several children play together where adults are present and the play has shared content. Playing together is voluntary, initiated by children or adults, and the children determine how much they wish to participate and how they move." pg 6 | 1. Norway, 3-5 years | 1. Bjorgen 2016 |
|  | Pretend play | | 1. "e.g., socio-dramatic" pg 3136 2. "Research examining social play behaviors determined pre-schoolers primarily engage in high amounts of pretend play behaviors." pg 258 3. "Pretend play (sometimes also called role play, makebelieve play, symbolic play or dramatic play) requires children to break down boundaries between imagination and reality (Bretherton 1989; Whitington and Floyd 2009)." p.61 4. " Pretend or dramatic play, a higher order play behavior (Maxwell et al., 2008), provides an important connection to performance on cognitive tasks including language and scholastic achievement. Language is more complex as children experiment with humor, rhyme, negotiation" p.29 | 1. Multiple, NA 2. USA, 0-3 years 3. USA, Mean 50.58 months 4. USA, NA | 1. Brussoni 2012 2. Dinkel 2019 3. Li 2016 4. McClintic 2015 |
|  | Practice play | | 1. "This type of play is characterized by repetitive, tactile activities. Running crawling, rolling and patty-cake are good examples." pp 18 | 1. NA, NA | 1. Guddemi 1992 |
|  | Purposeful play | | 1. "...purposeful play that pays attention to the students’ academic, physical, social, and emotional development. Purposeful play has a meaning behind it; the student has a reason for why he or she is doing what she or he is doing. For example, when a student is building a structure out of sticks, she might say that she is building a home for an animal, and, as she is doing so, she is learning engineering principles." | 1. USA, K-8 children | 1. Ashmann 2018 |
|  | Risky play | | 1. "...a means of promoting healthy development, and to learn about and manage risk to reduce and/or prevent injuries..."   "...risk as a vehicle for children to explore and test their capabilities, develop confidence, and learn to assess risk."   1. "Thrilling and exciting forms of play that involve a risk of physical injury. The risk can be real or perceived." pg 6425 2. "...risky play involves experimenting with uncertainty and overcoming fears. Sandseter outlines six categories of risky play, including play at speed (e.g. game of chase), heights (e.g. climbing trees), with tools (e.g. building a fort), near dangerous elements (e.g. fire, water, cliffs) and venturing out without adults (e.g. walking to school with friends)." pg 2 3. "Risky play is subsumed within physical activity play and has been defined as thrilling and exciting and where there is a risk of physical injury. Sandseter further categorizes risky play into play involving: heights, speed, dangerous tools, or near dangerous elements (e.g., fall into something), and where children can get lost." pg 3136 4. Measurement tool: Tolerance of Risk in Play Scale (TRiPS); "The scale is based on a Norwegian model of risky play." pg 7 5. "outdoor environment provides children with risky play opportunities" pg 51 6. "Through observations of children’s play and interviews with children and preschool staff, Sandseter identified six categories of risky play based on the physical activity and risk involved. These six categories of risky play were then validated and confirmed in a follow-up study. First, play with great heights – such as climbing, jumping down, hanging/dangling and balancing – involves the possibility of injury from falling. Second, play with high speed – such as sliding, running and swinging – involves the risk of uncontrolled speed that could lead to a collision with something or someone. Third, play with harmful or dangerous tools – such as using a knife for whittling or a saw for cutting wood – involves using instruments that could potentially cause physical harm. Fourth, play near dangerous elements – such as on top of a cliff or near deep water – involves the risk of falling into or from something. Fifth, rough-and-tumble play – such as play fighting, fencing with sticks or wrestling – involves the possibility that children could physically hurt or harm each other. Sixth, play where the children can ‘disappear’/get lost – such as exploring unknown areas or walking alone – involves a danger of getting lost. This systematic categorization of risky play aids in the conceptualization of risk in early childhood education and serves as a reminder that children have a strong desire to engage in various forms of risk-taking." pg 376   "Additionally, risk-taking and risky play experiences involve a borderline feeling of being out of control and a sense that one is overcoming fear. Moreover, risk-taking and risky play involve exposing oneself to hazards and the possibility of physical harm, with a perceived uncertainty regarding the outcome of the risky behaviour." pg 376  " Sandseter (2009a) identified two types of risk characteristics in children’s play. First, environmental characteristics are features of the environment, such as the height or steepness of a hill, that increase the chance of a child’s possible injury as a result of play. The amount of supervision by teachers and staff is considered an environmental characteristic. Second, individual characteristics are features of how play is carried out by the child, such as the height a child climbs, concentration or body control. Therefore, when examining children’s risk-taking and risky play experiences, it is essential to consider the types of activities they are engaging in, alongside the environmental and individual characteristics of those endeavours. Likewise, it is important to consider the individual differences with which children approach and engage in risk-taking and risky play experiences." pg 376   1. "Play that “involves thrilling and exciting forms of physical play that involve uncertainty and a risk of physical injury” (Sandseter, 2010b: 22). Six categories of risky play are also identified: (1) Play with great heights (danger of injury from falling), (2) play with high-speed (uncontrolled speed that can lead to collision), (3) play with dangerous tools (that can lead to injuries), (4) play near dangerous elements (such as fire, water, or heights), (5) rough-and-tumble play (where children can harm each other), and (6) play where the children can get lost." p.259 2. "wherein they can challenge themselves and engage in experiences that take them out of their ‘comfort zone’. Such play often occurs in the context of outdoor physical play and allows children to engage with uncertainty and experience alternating emotions of fear and exhilaration (Sandseter, 2009b). Risk-taking in play involves situations where children have an obvious desire to challenge themselves and extend their skills. In this context, children are acutely aware of their skill level and competence, and the aim of this type of play is to test their limits (Stephenson, 2003). A key element of ‘risky’ play is attempting something never done before, feeling on the borderline of being out of control (due to height or speed), and overcoming fear (Stephenson, 2003). Sandseter (2007) described risky play as experiences involving play with heights (risk of injury from falling) and speed (involving the risk of collision with other people or objects), rough and tumble play (where children can harm each other), play near dangerous elements (where children can fall into or from something) and with dangerous tools (risk of injury), and play in secluded environments (where children can disappear from the supervision of adults)." p.84-85 3. " Risky play involves testing boundaries and exploring risk (Ball, 2002), and attempting something beyond their current skill level (Stephenson, 2003)." p.320 4. "In general, ‘risky play’ might be defined as play that provides opportunities for challenge, testing limits, exploring boundaries, and learning about risk (Ball, 2002; Little & Wyver, 2008)." p.302 5. "In general, risky play might be defined as play that provides opportunities for challenge, testing limits, exploring boundaries and learning about injury-risk (Ball 2002; Little and Wyver 2008) and often includes the type of play that Caillois (1962) referred to as ilinx, which involves seeking states such as vertigo. Stephenson (2003) identified elements of four-year-old children’s play that were associated with risk-taking as ‘attempting something never done before, feeling on the borderline of “out of control” often because of height or speed, and overcoming fear’ (36). Children were noted to appear acutely aware of their own skill level and competence. The aim of this type of play was to test their own limits and display their physical skills. Similarly, Greenfield (2004) asked four-year-old children to convey their feelings and views about the outdoor playground. Favoured areas were the bikes, swings and ‘zoom slide’ which had common features – ‘risk, speed, excitement, thrills, uncertainty and challenge’ (4). The findings of Stephenson (2003) and Greenfield (2004) were further extended by Sandseter (2007a). In research conducted in Norwegian preschools with three to five year old children Sandseter identified six categories of risky play. Again height and speed and rough and tumble play were identified as significant elements, however, Sandseter also identified characteristics of the environment that contributed to risky play including dangerous tools that could cause injury, dangerous physical elements where children could fall, and secluded play where children could ‘disappear or get lost’. Regardless of the category, a common theme apparent from both observations of the children’s play and interview responses was the excitement and exhilaration experienced by the children and their obvious desire to seek out such experiences despite their acknowledgment that these could be frightening and associated with injury-risk. Overcoming fear and feeling ‘out of control’ was a significant element of the play." p.115 6. "risky play, defined as ‘thrilling and exciting forms of play that involve a risk of physical injury’ (p. 439)." p.33 7. "Risky play can be defined as a thrilling and exciting activity that includes some risk of injury.Often, risky play provides children with opportunities to challenge themselves, test limits, explore boundaries and learn to make decisions about injury and risk (Little and Wyver 2008; Sandseter 2007). Some “risky” activities include climbing, jumping, balancing, hanging upside down and sliding (Tovey 2010). Sandseter (2007) suggests that common themes in risky play are children’s sense of excitement, exhilaration, a desire to overcome fear and feeling “out of control”." p.159 8. "many children particularly seek ‘risky play’, the kind that most often occurs outdoors and beyond the purview of adults (Brown, 2009; Sandseter, 2007; Tovey, 2007). Within risky play, children experience ‘scary– funny’ feelings, the ambiguous emotional shifting back and forth between negative and positive emotions, pushing their limits and thereby strengthening physical and emotional skills (Sandseter, 2009)." p.224 9. "Risky play can generally be defined as thrilling and exciting forms of play that involve a risk of physical injury (Sandseter 2007)." 10. "Risk may be defined as a systematic way of dealing with hazards and insecurities induced and introduced by modernization (Beck 1992, 21)." p.1 11. "Thrilling and exciting play that can include the possibility of physical injury. Types of risky play include play at height, speed, near dangerous elements (e.g., water, fire), with dangerous tools, rough and tumble play (e.g., play fighting), and where there is the potential for disappearing or getting lost" pg6479 | 1. Canada, NA 2. International, children 3. Canada, 6-12 years 4. Multiple, NA 5. Australia, 5-7 years 6. Australia/Turkey, primary school students 7. Canada, 4-5 years 8. Norway, 1-3 years 9. Australia, early childhood 10. Australia, 4-5 years 11. Australia & Norway, preschool 12. Australia, 48-64 months 13. USA, 33-59 months 14. USA & Australia, 0-5 years 15. Australia, School children 16. Norway, 4-5 years 17. UK, 3-7 years 18. International, children | 1. Alden 2019 2. Brussoni 2015 3. Brussoni 2018 4. Brussoni 2012 5. Bundy 2011 6. Chancellor 2014 7. Coe 2017 8. Kleppe 2018 9. Little 2017 10. Little 2010 11. Little 2012 12. Little 2011 13. McClain 2016 14. McFarland 2018 15. Niehues 2013 16. Sandseter 2009 17. Savery 2017 18. Tremblay 2015 |
|  | Rough-and-tumble play | | 1. “Where children can be harmed. Example: Wrestling or play fighting with other children or parents.” Pg 6425 2. "while playing, he can be thrown down, pushed or pulled by another child" pg 74 3. "...wrestling and piling their bodies on top of one another. Similar to Sandseter (2007a, 2007b), Teacher Jane was aware of the ‘fine balance between play and real fighting’ (p. 246)." pg 381 | 1. Multiple, NA 2. NA, MA 3. Canada, 4-5 years | 1. Brussoni 2015 2. Ceciliani 2013 3. Coe 2017 |
|  | Sand play | | 1. "Sand play can enhance the environmental experiences of children when they use recycled plastic containers as sand scoops and play with natural materials such as stones, twigs, bark, and leaves in the sandbox.". pp. 222 | 1. USA, 3-4 years | 1. Jarrett 2010 |
|  | Social play | | 1. "Research examining social play behaviors determined pre-schoolers primarily engage in high amounts of pretend play behaviors." pg 258 2. "Social play is particularly important in this respect because it captures so many aspects of development. Once acquired, social play incorporates intention, interrelatedness, emotional directedness and narrative ability." 3. "Social play involves the coordinated participation of different individuals and it is thus an excellent opportunity for promoting psychosocial development. This kind of play occurs when a child is motivated to engage other children in shared playful activities, is able to regulate emotional arousal, and possesses the skills required to initiate interactions with others such that his or her social overtures are responded to in kind (Coplan, Rubin, and Findlay 2006)." p.186 4. "social play (i.e., associative and cooperative play; Parten, 1932)" p.247 | 1. USA, 0-3 years 2. NA, NA 3. Spain, 3-6 years 4. USA, 3-5 years | 1. Dinkel 2019 2. Jordan 2003 3. Larrea 2019 4. Martin 2015 |
|  | Solitary play | | 1. Solitary play occurs when children play alone and relatively apart from their peers, to whom they do not pay attention; it usually involves playing with different materials to what the others are using. 2. "solitary play (i.e., playing alone and independently with toys)" p.247 3. (solitary functional play) solitary-functional play (repeated sensorimotor actions with or without objects such as hopping or skipping)" p.813 | 1. Spain, 3-6 years 2. USA, 3-5 years 3. USA, mean 59.63 months | 1. Larrea 2019 2. Martin 2015 3. Nelson 2008 |
|  | Sociodramatic play | | 1. "constructions, which may represent dams, rivers, cars, trains, space ships, houses, or forts, often become the focus of sociodramatic play themes." pg 39 | 1. Australia, NA | 1. Davies 1996 |
|  | Spontaneous play | | 1. “…spontaneous play, which is organized by the children themselves…” pg 258 2. "...essential to define, inside the so-called ‘free play’, the conditions of the action in their twofold meaning: positive, because, to some extent, they can in fact be defined as the ensemble of those elements that enable to carry out the activity, and which constitute a setting made of times, spaces, tools, and of educational attentions; but we must also recall that the same elements restrain the activity of play, that they condition it in a negative way because they offer guidance that is strictly dependent upon rules (i.e. limitations) determined by the educators (teachers, assistants, etc.). The overall analysis of the various situations we have observed conveys the impression that we should not talk about ‘free play’ inside schools, but we should use a more correct definition: spontaneous play; even though freedom of play is assured, at least at the level of initial impulse (so that spontaneity is assured), it nevertheless seems to be extremely limited in the choices that are conditioned by the context to the point that we could here use the concept of probation." pg 72-73 | 1. Norway, 1-6 yrs   22-63 yrs (teachers / assistants)   1. NA, NA | 1. Bjorgen 2015 2. Ceciliani 2013 |
|  | Structured play | | 1. "Structured play which has an outcome in mind and often is adult led" pp.782 2. "as a planned movement time designed to incorporate opportunities to practicing basic motor skills and make use of large muscle groups." p.3 | 1. Multiple, under 12 years 2. Italy, 4-5 years | 1. Houser 2016 2. Tortilla 2019 |
|  | Structured educational play | | 1. "structured educational play that is initiated by practitioners may also be child-led. Here the adult may act as facilitator or, for example, may guide, scaffold or model. Importantly, while the adult may have specific planned learning outcomes, children are not made to pursue an activity and should be allowed to develop their own ideas (DCELLS 2008a; DCELLS 2008b) (see Maynard et al. 2013)." p.214 | 1. UK, 4-7 years | 1. Maynard 2013 |
|  | Symbolic play | | 1. "This occurs when a child can mentally visualize a symbol and use it in play. Symbolic play is particularly important because it develops skill and in the use of play symbols which is related to useage in reading." 2. "Child engages in pretend play using actions, objects, or ideas to represent other actions/objects/ideas." p.6 | 1. NA, NA 2. USA, 3-15 years | 1. Guddemi 1992 2. Miller 2017 |
|  | Unstructured play | | 1. "...children’s free play outdoors."; Examples: "...freedom, make up games, explore new places". pg 257 2. "Time spent outdoors has been proposed and validated as a surrogate measure of PA in children to capture unstructured play." pg 252 3. "...indicator of unstructured PA." pg 255 4. "By using Di Pietro’s schematic, which is based precisely on the paradoxical nature of play, we can assert that the various forms of play can be grouped into eight dimensions situated along a circular schematic (Figure 1), and diametrically opposed, which exemplify in a paradigmatic way the two-faced nature of play." pg 7; Figure 1, "The opposing dimensions of play", which includes: "rite", "vertigo", "cooperation", "freedom", "chance", "mimesis", "challenge" and "rule". pg 68 5. "Studies investigating unstructured play show that children play at more vigorous levels of exertion when they play outdoors as opposed to indoors. Activities of this type include biking, skateboarding, swimming, hiking, climbing trees, and playing noncompetitive games such as tag. During these activities, the participants determine their own level of involvement and establish parameters of any rules concerning time limits and any social interaction between participants." pg 510 6. "Unstructured play is described as child-led play with no specific outcome or rules in mind, allowing for the child to work on decision-making and discovery on their own" pp.782 7. "Unstructured active play is defined as the activities children participate in that are sponatneous and without a set regime or purpose." pp. 58 8. "Unstructured play opportunities have been sown to promote variety, choice and diversity to students' play engagement in primary school settings through the introduction of natural features trees, rocks, gardens), play pods (assortment of materials) and everyday equipment (pipes, crates, buckets)." pp. 243 | 1. Canada, NA 2. USA, 9-13 years 3. Switzerland, 6-14 years 4. NA, NA 5. USA, 10-14 years 6. Multiple, under 12 years 7. NA, NA 8. Australia , 5-12 years | 1. Alden 2019 2. Beyer 2015 3. Bringolf-Isler 2010 4. Ceciliani 2013 5. Christiana 2014 6. Houser 2016 7. Hyndman 2015 8. Htndman 2018 |
|  | Water play | | 1. Examples: "Puddles, spray bottles, garden sprinklers, and backyard wading pools bring back gleeful memories of childhood" pg 28   Author states that 'water play' can be used for: cognitive development, learning mathematics, learning science, learning physical skills, learning social skills and learning language. | 1. NA (maybe USA), NA | 1. Crosser 1994 |
| [Back to Table of Contents](#CONTENTTABLE) | | | | | |
|  | | | | | |
| **Outdoor Play/Time/Playtime** | | | | | |
| *Outdoor play* | | | 1. "Outdoor play is a cheap and natural way for children to be physically active… outdoor play is cheap, informal, and easily accessible." 2. "Recreation associated with outdoor play and activity in the natural forest landscape are part of the everyday kindergarten experience and hence provide the location for much of the children’s learning... irrespective or season or weather."   "Outdoor play and activity give children more options for choice and more freedom to play and explore, loudly and wildly if they choose, without the restraint of adult control."  "More vigorous than indoors and play forms take different group and gender constellations"  "Children are capable of making their own decisions in this environment where they can exert influence over their own and others’ actions, where their opinions are listened to and regarded as important"   1. "...participants articulated three interrelated elements central to outdoor play: child agency, play-rich environments, and adult support, grounded in a common view of children as competent and capable, and risk as inherent to children’s play."   "...outdoor play may be viewed as a means of enhancing learning and connecting children to nature."  "Risk, therefore, is inherent to children’s self-direction in outdoor play."  Outdoor play is comprised of three core elements: "child agency, play-rich environments, and adult support".   1. "Outdoor play was defined as hours per week spent outside playing." 2. Places that outdoor play can "occur include the home, early childhood education and care (ECEC), and community facilities such as parks, playgrounds, recreation centres and swimming pools". 3. "Outdoor play has a quality distinct from time spent indoors. The sights and sounds are different, and different standards apply. Activities that may be frowned on indoors can be safely indulged outside, and the child is generally allowed greater freedom not only to run and shout, but also to manipulate and transform the environment. Such activities are significant to the motor and cognitive development of young children, and also to their growing sense of self. Going outdoors means experimenting with separation not only in a physical way, but also symbolically. To be able to go freely back and forth between inside and out is a crucial and integral component in the child’s experience of autonomy." pg 39 4. Outdoor play activities noted in the case study include: slides, running fast, swing, merry-go-round, teeter-totter, acting as the 'leader', bubbles, playing outside with friends/family (social activity), holistic, and play involving the whole body (e.g., climbing on hands and feet).   "...outdoor play was more than a kinesthetic experience: her outdoor play had verbal, cognitive, and emotional elements as well." pg 49  "Outdoor play as a holistic experience." pg 46  "Outdoor play is not ‘nature’ play." pg 46   1. "The extent and frequency of time spent in outdoor play was measured by asking parents, “On a typical week, how many hours is your child outside playing?”" pg 114 2. "...outdoor play as a vehicle for supporting leadership and choice-making in the lives of young children." pg 149   "[Outdoor play] provides opportunities to engage in freely chosen play. Children can choose when and where they play during outdoor play. Though adults control indoor activities, children can freely choose their outdoor play. They can choose rough-and-tumble, pretend, or games with rules. This may be the only time during the day when they can control their activity level." pg 152  "…encourages different levels of social participation as children self-select playmates. . . . This is the richness of outdoor play. Children can disengage if they so desire. They can sit alone and stare at the sky if they choose. They select their level of social engagement." pg 153   1. "...during outdoor play, it is possible to adopt more flexible teaching strategies that may stimulate children’s autonomy and participation. In the study of Maynard, Waters and Clements (2013), teachers used outdoor play time to observe, interact and obtain important information about each child’s needs and interests, developing a different perspective about children’s achievements. Teachers recognised that during outdoor play, children were less pressured to respond and act accordingly to performance expectations, being able to show skills and knowledge rarely observed inside the classroom." pg 290 2. "...outdoor play such as sledding and rock climbing..." pg 2059 3. "...a broad range of activities, including but not limited to riding a bicycle, walking, hiking, water play, playing games with friends, climbing trees, and exploring." pg 255 4. "According to Veitch et al., outdoor play refers to any unorganized physical activity outdoors. Furthermore, outdoor play is freely chosen, spontaneous, self-directed, and includes activities the child enjoys. In contrast to organized physical activities, like sports conducted in sports clubs, playing outdoors is often cheap and freely accessible." pg 1 5. "Sobel (2012) posits that one of the barriers to adopting outdoor play as part of the educational experience is the inherently risky nature of that play." pg 1035 6. Example: "two activities were “Track Team,” which consisted of jogging around the playground with teachers and peers, and “Dance Party,” which entailed dancing to high-energy preschool music with teachers and peers in a specified area of the playground." pg 129 7. Locations of outdoor play include: playgrounds, streets, community/neighborhoods, yards. 8. "The unstructured nature of recess and outdoor play" pg 2 9. "Resmussen and Smidt (2003) stressed two key factors to outdoor play – the excitement factor with potential for challenge and risk and the imaginative factor where the outdoor environment can provide limitless opportunities to create games of fantasy and engage children’s imagination." pg 557 10. "Children self-reported whether they went for a walk in their neighborhood, took their dog for a walk, played in the street or played outside in the yard in the last week (percent agreement ≥ 60%; yes/no)." pg 260 11. "...freely chosen outdoor play activities..." pg 68 12. "Boys are more active than girls and spend more time outside than girls in programmes which offer indoor and outdoor activitiess concurrently Boys perform more fantasy play outdoors than girls, making use of large open spaces and apparatus which favour physical activity for this purpose." pp. 46 13. "child outdoor play or the time the child spent playing outdoors on a typical day, during the morning, afternoon, and early evening" pg 2 14. "...opportunities for independent play in natural outdoor spaces, and the associated opportunities to take and negotiate risk," pg. 721 15. "….like ‘physical action/body contact’, ‘rough and tumble play’ and ‘belief in skills and abilities of the children’". pg.550 16. "...time spent outdoors engaged in active play" p.179 17. "offers an enriched learning environment, where children’s imaginations, inventiveness, and creativity flourish....allowing children to have a wider range of gross motor movement and visual exploration, resulting in additional opportunities for decision-making, problem solving, and creative thinking." pg.645 18. "Children's play in an unstructured environment, preferably a natural one." pg.111 19. "…Children often experience a sense of freedom that encourages them to become involved in interactive games that foster language and create authentic opportunities for problem solving." pg.20 20. "uninhibited, unstructured tree-time play in natural or wild environments" pg.21 21. "...times we spent outdoors in natural, unstructured spaces." pg.42 "Outdoor time spent beyond the playground has added benefits. Development of physical skills is a major benefit of play in natural environments (Fjortoft & Sageie 2000; Wells 2000), and there is also evidence of increases in creative and imaginative play in these environments (Taylor et al. 1998)." pg. 42 22. (unscheduled outdoor play) ..."spend[ing] time doing activities such as jumping rope, rollerblading, riding a bike or playing at a playground outside of regular school hours or scheduled practices, games or classes...outdoors[.]” pg. 21 23. (Outdoor activities) "[activity] In their garden, in the playground, [or] on the street" pg.949 24. "Outdoor play provides open-ended, dynamic, varied opportunities which are unpredictable, and at times, risky. However, the risks and challenges of being outdoors provides rich opportunities for learning, problem-solving and developing social competence." pg.1 25. "... where children wearing accelerometers went outside and engaged in free choice activities, under teacher supervision but without any directions to be active." pg.533 26. "provide opportunities to enhance child growth and development in ways that are not provided by indoor play environments (e.g.,Henniger, 1980).pg.669 27. "...physical play, in particular unscripted play out of doors in nature." pg.1015 28. "Outdoor play consists of a combination of sedentary, light, moderate, and vigorous activity" pg. 478 29. “Outdoor play has been validated and is often used as a proxy measure of PA in this population. Outdoor play has been shown to facilitate interaction with peers, thereby providing more opportunities to support socialisation and development of social skills and children's overall social health. Therefore, outdoor play provides opportunities for children to be both physically active and socially engaged." pp. 2 30. "Programmes of outdoor play often focus on physical activities and recess, rather than encouraging cognitive skills and social interaction… One distinct features of outdoors is the greater space and freedom of movement available for children… another feature is different types of materials that can be provided in outdoor settings... In outdoor settings, children frequently engaged in exploration, manipulation of materials, functional play and locomotion" pp. 1612 31. "This statement recognizes that 'access to active play in nature and outdoors, with its risks, is essentual for healthy child development" and it recommends 'increasing children's opportunities for self-directed play outdoors in all settings - at home, at school, in child care, the community and nature" pp.782 32. "Outdoor play is regarded as either free structured physical activity taking place in an exterior environment." pp.203 33. "Outdoor play helps children develop an appreciation of nature, which often offers healing porwer and mediates stress; helps children develop a sense of season, and helps them see their personal connections to nature. It also increases [preschoolers] classroom learning and beneits their cognitively related performance." pp. 55 (Bohling, Holmes 2006, Werner 2006, Jarrett 2001 34. "studies suggest that outdoor play such as recess actually produces equally important cognitive, socio-emotional, and physical benefits as classroom schooling and academics." pp. 20 35. "Outdoor play is considered a natural way for children to be healthier and physically active." pp. 81; "Research also suggests that outdoor play is positively associated with children’s academic achievement and social–emotional intelligence. Exposure to the outdoor environment enhances children’s own investigations, supports motor development and eye–hand–foot coordination, reduces tension and affects personality formation and cognitive growth." pp. 82; "Compared to indoor environments, outdoor environments provide more fundamental stimulation for all aspects of children’s development. According to Dodge, Colker, and Heroman (2010), the outdoor environment is a more suitable setting than indoors for the development of large muscle-intensive and motor skills, allowing children to engage in a wide range of activities like playing in sand and water, swinging, climbing, sliding, jumping, hanging, digging and using wheeled toys." pp. 82 36. "Outdoor play also fosters social-emotional and cognitive development through exposure to nature, activities that engage problem-solving skills and creativity, and unique opportunities for imaginative group- and free-play." pp.2 37. "Outdoor play provides opportunities to stimulate problem solving and imagination.1 It is also commonly associated with physical activity.2 Thus, the time spent on outdoors frequently has the advantage of muscular activity and caloric expenditure." p.S218 38. " Physical exercise and opportunities for growth in all developmental areas. […]the amount of time that children spend engaged in unstructured, child-directed outdoor play […] whether on the streets and sidewalks of the city, in backyards, or in parks and undeveloped spaces." p.446 39. "Outdoor play was construed as providing a break from the more rigid work time schedules indoors." p.377 40. "Children’s play outdoors is often seamless, moving from one interest to another reflecting the natural rhythms of children’s concentration and curiosity (Nelson, 2012). When opportunities for play occur in nature-rich environments, children develop the skills across all learning domains." p.71 41. "Outdoor play activity is often physically active, with hands flying, feet kicking, and faces covered with mud. During normal outdoor play activities, children exhibit a wide variety of behaviors that may influence the amount of dermal contact they have with their environment." 42. “‘Outdoor play provides open-ended, dynamic, varied opportunities which are unpredictable and at times risky. However, the risks and challenges of being outdoors provide rich opportunities for learning, problem-solving, and developing social competence.”; “Outdoor play provides opportunities for children to learn and gain competence in a vast range of motor skills. This is particularly important during the early childhood years, a period hallmarked by significant development across all domains. Outdoor play provides occasions for children to develop and refine basic locomotor skills, including walking, running, jumping, climbing, hopping, skipping, sliding and tricycling; manipulative skills such as throwing, catching, kicking, striking and bouncing; and stability abilities including bending, stretching, swinging, twisting and beam-walking (Gallahue, 1993; Poest, Williams, Witt & Attwood, 1990).” 43. "Outdoor play is a particularly important context and opportunity in which children develop the ability to perceive and appraise risks as they learn to avoid injury whilst developing and mastering a wide range of gross motor skills." p.297 44. "Objectives for outdoor play for young children include cognitive, creative, physical and social-emotional outcomes occurring in an integrated fashion in either self-directed play or guided play (Bilton, 2010; Cullen, 1993; Frost & Norquist, 2007).[...]Outdoor play should involve elements of physical play, constructive play, social play, sociodramatic play, and games with rules (Bilton, 2010; Hendricks, 2001; Henniger, 1993)." p.28-30 45. "Outdoor play concerned exercise at school and outside school hours for an average week. At the age of 6 years, frequency (number of days) and duration (never, less than 30 min, 30–60 min, 1–2 h, 2–3 h, 3–4 h) were asked for weekdays and weekend days separately. The midpoint of each category (e.g. 45 min for 30–60 min) was used to estimate the duration of a session. The frequency was multiplied by duration, and estimates for weekdays and weekend days were summed to obtain the average time spent playing outdoors in minutes per week at the age of 6 years." p.3 46. "Location based outdoors play: Children were asked how often they play outdoors in the afternoon in three different types of locations, including: (1) recreational facilities (i.e., park, playground, courtyard); (2) public facilities (i.e., community center, library, culture center); and (3) streets within the home neighborhoods." p.5 47. The values of outdoor play are far more than giving children a break, or allowing them to run off steam or get fresh air. Research has shown that • outdoor play encourages children to communicate, to express their feelings, to discover and investigate the world around them (Guddemi, Jambor, & Moore, 1999), and that • play is an important vehicle for developing self-regulation, language, cognition, and social competence (NAEYC, 2008). 48. "Outdoor play was assessed using two items. The first asked students: “On weekdays, how many hours a day do you usually spend time playing outdoors outside school hours?” The second asked the same about weekends (Freeman et al., 2016) " p169 49. "How many days per week and how many hours per day does your child plays outdoor on weekdays/weekend days" p.632 50. Time spent playing outdoors. “On a typical weekday, how much time does your child spend playing outdoors?” pg 123 51. " Children outdoor play is often associated with physical play where uses of gross motor skills dominate."pg. 447 52. 2. "Outdoor play offers children opportunities to reach developmental milestones that may not be possible indoors. Outdoor play involves risk taking, gross motor development, and exploration. Outdoor play also offers children the opportunity to push their limits and learn about their capabilities" pg. 28 53. (vigorous outdoor play) "To obtain an overall estimate of time spent vigorously playing outdoors on weekdays and on the weekend, the categorized time-intervals were transformed into a continuous score using the intervals' midpoints and an assumed maximum of 8 hours. First, a weekly time and then a daily average were calculated. A sensitivity analysis of the timescores indicated that results did not change if the minimum or maximum value of each time-interval was used. In addition, the comparison of the score with accelerometer data in a subsample of 167 children showed an acceptable correlation (r= 0.52)." pg. 252 54. "Sobel (2012) posits that one of the barriers to adopting outdoor play as part of the educational experience is the inherently risky nature of that play." pg 1035 55. (outdoor free play) "Active free play usually occurs outdoors and has been associated with increased moderate- to vigorous-intensity physical activity (MVPA)." pg 146; "Aside from time in daycare and preschool, on a typical weekday, how much time does your child spend outside in “unstructured free play?”’ Parents were instructed to provide their response in minutes per day." pg 146 56. (vigorous outdoor play) "...run, climb, and jump in outdoor spaces..." pg 69 57. (outdoor free play) ''…unstructured physical activity that takes place outdoors in the child’s free time. It is also known as a voluntary, child-initiated activity that allows children to develop their imaginations while exploring and experiencing the world around them.'' p.132 58. (outdoor free play) "naturalistic sessions…where the child generally played with a parent or sibling (if at home) or with an aide or friend." pg.41 59. (outdoor playing activities) ."... the minutes per week (weekdays and weekend days combined) the child spent on the different leisure activities outdoors." pg.4 60. "Outdoor play consists of a combination of sedentary, light, moderate, and vigorous activity" pg. 478 61. "Outdoor play provides an important context for children to explore, to experiment, to move, be themselves and make the most of the opportunities afforded by the environment in a less restricted manner (Henniger, 1994; Rivkin, 1995)." p.301 62. "Objectives for outdoor play for young children include cognitive, creative, physical and social-emotional outcomes occurring in an integrated fashion in either self-directed play or guided play (Bilton, 2010; Cullen, 1993; Frost & Norquist, 2007).[...]Outdoor play should involve elements of physical play, constructive play, social play, sociodramatic play, and games with rules (Bilton, 2010; Hendricks, 2001; Henniger, 1993)." p.28-30 63. (outdoor free play) "children outdoors and could choose activities" p.2 64. "In the past month, how often did you take [child] outside for a walk or to play in yard, a park, or a playground" pg. 708 65. (outdoor free play) ''…is outdoor play happening outside of daycare or preschool hours. Additionally, the preferable variation is unstructured free play." p.446 | 1. Denmark, 4-12 yrs 2. Norway, NA 3. Canada, NA 4. Canada, 0-5 years 5. Australia, 2-5 years 6. USA, < 6 years 7. Canada, 4 years 8. USA, 3-5 years 9. USA, 3 years 10. Portugal, 4 months – 10 years 11. USA, 10-13 years 12. USA, 9-13 years 13. Multiple, high income countries, 0-12 years 14. Multiple, NA 15. USA, 4 years 16. NA, Young children 17. USA, NA 18. UK, 3-5 years 19. Australia, 10-12 years 20. USA, 3-12 years 21. Australia, 64.15 months for boys and 63.75 months for girls 22. USA, 2-5 years 23. England/UK, Primary school age 24. Norway & Austria, Preschool age 25. New Zealand, NA 26. USA, NA 27. Norway, 5-7 years 28. USA, NA 29. Australia, Grades 6-7 30. USA, preschool age 31. USA, 6-8 years 32. Greece, 2-14 years 33. Australia, 4 years 34. USA, 3-5 years 35. USA, preschool 36. USA, 2.5-5 years 37. Canada, NA 38. Australia, 2-5 years 39. Japan, 3-5 years 40. Various, Under 12 years 41. China, 3-6 years 42. China, Kindergarten 43. USA, Kindergarten to Grade 3 44. Oman, Kindergarten 45. USA, 3-5 years 46. USA. 5-7 years 47. USA, Young children 48. Ireland, 1-5 years 49. USA, 3-5 years 50. USA, NA 51. NA, children 52. Australia, 4-5 years 53. USA, NA 54. Netherland, 6 and 9.7 years at two timepoints 55. Israel, 10-12 years 56. USA, early childhood 57. Canada, 11-15 years 58. Netherland, 3-4 years 59. Canada, 11 years 60. Norway, 3-5 years 61. Canada, NA 62. Switzerland, 6-14 years 63. Multiple, NA 64. Canada, 1-5 years 65. USA, 3-12 years 66. USA, 4-5 years 67. USA, 8 months-6 years 68. Denmark, 2-6 years 69. Canada, NA 70. Australia & Norway, preschool 71. USA, NA 72. USA, 3-5 years 73. USA, Mean 3.47 years 74. Australia, NA | 1. Aarts 2010 2. Aasen 2009 3. Alden 2019 4. Anderson 2015 5. Armstrong 2010 6. Bartlett 1997 7. Beattie 2015 8. Becker 2018 9. Bentley 2012 10. Bento 2018 11. Beyer 2015 12. Beyer 2015 13. Boxberger 2019 14. Brown J 2017 15. Brown W 2009 16. Burdette 2005 17. Butler 1997 18. Canning 2010 19. Christian 2014 20. Clements 2004 21. Cullen 1993 22. Davison 2011 23. Elliott 2015 24. Emilsen 2010 25. Ergler 2013 26. Ernst 2012 27. Fjortoft 2001 28. Flynn 2002 29. Francis 2013 30. Galizio 2009 31. Galvez 2013 32. Grammatikopoulus 2018 33. Greenfield 2004 34. Hannon 2008 35. Hart 1986 36. Haywood-Bird 2017 37. Herrington 2015 38. Hinkley, 2018 39. Hirose, 2012 40. Houser 2016 41. Hu 2014 42. Hu 2015 43. Hughes 2017 44. Ihmeideh 2016 45. Jayasuriya 2016 46. Kalish 2010 47. Kemple 2016 48. Kernan 2010 49. Kiewra 2016 50. Ko 2007 51. Little 2008 52. Little 2010 53. McClintic 2015 54. Molenberg 2019 55. Moran 2017 56. Olsen 2011 57. Piccininni 2018 58. Sijtsma 2015 59. Stone 2014 60. Storli 2010 61. Turner 2009 62. Bringolf-Isler 2010 63. Brown 2017 64. Carsely 2017 65. Clements 2014 66. Zamani 2016 67. Guerette 2013 68. Handel 2017 69. Herrington 2015 70. Little 2012 71. McClintic 2015 72. Tandon 2015 73. Tandon 2012 74. Wolfenden 2016 |
|  | Outdoor active/active outdoor play | | 1. "...outdoor active play captured unstructured and informal physical activities that are freely chosen, typically child-led with little or no adult supervision, and which occur in a variety of outdoor locations (e.g., exploring nature, playing at recess, playing basketball in the driveway)." pg 178 2. "Outdoor active play refers to unstructured and informal physical activities that are freely chosen, typically child-led with little or no adult supervision, and which occur in a variety of outdoor locations" pg 2 3. " unstructured free play with an emphasis on running, tumbling, throwing, and catching." pg. 1 4. "...complex interaction of land use, infrastructure, and social factors". pg.1 5. "Outdoor active play (OAP) is a type of physical activity that is particularly low among children living in industrialized countries [11, 12]. OAP refers to physical activity that is comprised of games or symbolic play that occurs outdoors and includes playground activities, ball games played in the street, and backyard games like tag and red rover. It is typically self-directed by children and involves little or no adult supervision." p.1 6. "e.g., tag, climbing trees, road hockey" p.937 7. "Active play, particularly when performed outdoors, is a spontaneous, self-motivated, fun, and unstructured form of physical activity that provides children with physical, mental, and social health benefits (Tremblay et al., 2015; Burdette and Whitaker, 2005)." p.304 8. (outdoor active free play) “Aside from time in day care and preschool, on a typical weekday, how much time does 9. your child spend outside in unstructured free play?” This wording is from the validated Canadian Community Health Survey23 and is similar to wording in previous validated measures of outdoor play.21" p.446 10. " Active outdoor play, sometimes referred to as active free-play or self-directed play is defined here as, “unstructured physical activity that takes place outdoors in thechild’s free time.” pg.6479 | 1. Canada, 10-13 years 2. Canada, 10-13 years 3. USA, 2-5 years 4. Canada, Kindergarten to Grade 8 5. Canada, 10-13 years 6. Canada, children 7. Canada, 10-13 years 8. Canada, 1-5 years 9. Canada, children | 1. Borghese 2019 2. Borghese 2018 3. Grigsby-Toussaint 2011 4. Harper 2019 5. Lin 2018 6. Macgregor 2019 7. Nguyen 2018 8. Sharp 2018 9. Tremblay 2015 |
|  | Outdoor motor play | | 1. "...what defines the concept of motor play in a precise way is the fact that it depends upon the player’s movements, the way in which they are performed, so much so that it is not irrelevant to evaluate both modes and efficacy of all body movements to appreciate play in itself" pg 7   Examples (Figure 2): handling, running, jumping, hopping, jumping down, throwing, climbing, sliding, kicking, swinging, fighting (rough and tumble), pulling/pushing, cylcing, carrying. pg 70 | 1. NA, NA | 1. Ceciliani 2013 |
|  | Outdoor pretend play | | 1. "The majority of the research on pretend play in early childhood takes place in indoor settings, neglecting the wealth of pretend play opportunities that children may engage in outdoors. Outdoor environments permit children to engage in a wide range of pretend play scenarios" p. 62 | 1. USA, mean 50.58 months | 1. Li 2016 |
|  | Outside play | | 1. "Outside play (i.e., PA without any given tasks or goals; unstructured free play) " p.2 | 1. Netherland, 5-7 years | 1. Remmers 2014 |
|  | Cooperative outdoor play | | 1. " Cooperative play,,the most mature social play type defined by Parten (1932), can also be encouraged outdoors to strengthen young children's developing social skills. Although the sandbox has typically been one of the few designed spaces out doors where children can engage in this important activity, other options such as outdoor blocks and construction mate rials (both pretend and "real") are needed to create a variety of play options to stimulate this complex social play type." pg.11 | 1. USA, NA | 1. Henniger 1994 (Young Children) |
|  | Solitary outdoor play | | 1. " solitary play, although the least mature social play type (Parten, 1932), is an important experience for young children that can be encourage in outdoor settings. Children need quiet spaces outdoors for reflection and imagination."pg.11 | 1. USA, NA | 1. Henniger 1994 (Young Children) |
|  | Unstructured outdoor play | | 1. "Unstructured outdoor play consists of open-ended play that allows freedom to construct rules, goals, and meaning. Unstructured outdoor play provides children opportunities to create their own sensory experiences in various play environments, such as playing in a sandbox or running in the grass. Outdoor unstructured play also provides opportunities for improvement of physical and social competencies by playing with peers on a variety of surfaces and structures such as grass, sand, and slides. It supports children’s autonomy and imagination, as children get to select how and with what they play. It can also support overall health as pre-schoolers (3–5-year-olds), in outdoor play environments, when compared to indoor environments, have been shown to take part in more vigorous physical activity." pg 258 2. "Kids who don’t have a lot of unstructured outdoor play are at risk for a range of negative outcomes:3   • Greater risk for unhealthy lifestyles and childhood obesity  • Fewer sensory-rich experiences  • Higher diagnoses of ADHD-type symptoms  • Lower academic performance in school  • Less empathy for plants and animals  • More apathetic/destructive attitude toward others  • Lower self-confidence." pp.   1. " Data on frequency of outdoor play during leisure time ('how often do you play games outside after school?')" p.3 | 1. USA, 0-3 years 2. NA, NA 3. Norway, Estonia, Portugal, 9-15 years | 1. Dinkel 2019 2. Fish 2018 3. Nilsson 2009 |
|  | | | | | |
| *Outdoor Time* | | | 1. "Time spent outdoors was estimated using the PLAYCE Childcare Daily Schedule. On an hourly basis, educators documented the main activity types in which children were engaged. Each type of activity which included reference to outdoors (e.g. outdoor play, free play outdoors) was coded and added together to give an estimate of time spent outdoors for that day." pg 3 2. "the home outdoor area (e.g., presence of yard)" pg 26   "The outdoor home environment (i.e., front/backyard) is a particularly important space that provides opportunity for young children to play, explore and be active." pg 30   1. "outdoor time were assessed using questionnaires and physical activity was measured using the SC-StepRX pedometer" pg 1   "Outdoor time was assessed in the parent questionnaire through two categorical questions: (i) ‘on a typical weekday, how much time does your child spend playing outdoors at the moment?’ and (ii) ‘on a typical weekend day, how much time does your child spend playing outdoors at the moment?’" pg 3   1. "For weekdays, participants reported outdoor time for four periods of the day: before school, at school, after school, and after dinner. For each period, they were asked: “During the past month, on an average school day, how much time did you usually spend outside […]?” The response options were: none; 1 to less than 15 minutes; 15 to less than 30 minutes; 30 minutes to less than 1 hour; 1 to less than 2 hours; and 2 hours or more. Using the same response options, weekend outdoor time was assessed: “On an average day during the past month, when you did not go to school, for example, on the weekend, how much time did you usually spend outside?” p.4 2. "Outdoor time was assessed via the three following items developed specifically for this study: “On a school day how much time did you spend outside before school?” “On a school day how much time did you spend outside after school before bedtime?” and “On a weekend day, how much time did you spend outside?” For all items, response options were (1) <1 hour, (2) 1 hour, (3) 2 hours, (4) 3 hours, (5) 4 hours, and 6) 5 or more hours. Together, the three outdoor time items had good internal consistency (Cronbach’s α = .83)." p.119-120 3. Time spent outdoors doing MVPA (this is what it seems like they think it is but this def is not directly in the text) "Common recording periods for accelerometry and direct observation were 20 to 30 min outdoors, for 4 consecutive days." pg. 79; " There was a large degree of variability in the outdoor playtime reported in the 26 studies. Outdoor time measurements ranged from a minimum of 10 min to a maximum of 211 min, with a mean of 45.2 min, over a typical 7.5-h day at childcare. Common recording periods for accelerometry and direct observation were 20 to 30 min outdoors, for 4 consecutive days" pg.79 | 1. Australia, 2-4 years 2. Multiple, <= 7 years 3. Multiple, 9-11 years 4. Canada, 7-14 years 5. Multiple, 9-11 years 6. Canada, 2-5 years | 1. Christian 2019 2. Christian 2015 3. Delisle Nystrom 2019 4. Larouche 2016 5. Larouche 2019 6. Truelove 2018 |
|  | | | | | |
| *Outdoor Playtime* | | | | | |
|  | Outdoor playtime | | 1. " the number of minutes the child typically spent playing outdoors each day in the last month during the week and on the weekend." pg. 358 | 1. USA, 18 months-5 years | 1. Gross 2013 |
|  | Independent outdoor time | | 1. "Outdoor time, that is, characterized by independent, self‐directed activities without adult supervision, for example, free outdoor play, fort building, drawing, or bicycling with friends." pg.699 | 1. USA, adults | 1. Hecht 2019 |
|  | Modified outdoor playtime | | 1. "modified outdoor playtime (i.e., re-structuring the provincially required two 60-min outdoor sessions into four 30-min periods). Given recent evidence to suggest that preschoolers’ activity levels are highest during their first 10 min outdoors, and consequently, that simply extending outdoor playtime may not be adequate for promoting physical activity, the SPACE intervention consisted of a modified outdoor playtime schedule to increase the frequency, but not the duration, of unstructured outdoor playtime provided to young children in childcare" p.3 |  |  |
|  | Nature-based playtime | | 1. "...access to natural environments..." pg 202 | 1. UK, 8-9 years | 1. Barton 2015 |
|  | Playground-based playtime | | 1. "...equipment provision and playground markings…" pg 197 | 1. UK, 8-9 years | 1. Barton 2015 |
| [Back to Table of Contents](#CONTENTTABLE) | | | |  |  |
|  | | | | | |
| **Education/Learning/School** | | | | | |
|  | Nature-based education/  learning | | 1. (Nature-based learning model) "...this type of nature-based or outdoor-learning can be thought of as the Forest School approach." pg 38 2. "Nature-Based Learning is often grouped with similar concepts such as place-based education, environmental education, outdoor education and environment-based education (Louv, 2008). Despite subtle differences among them, they all emphasize a first-hand, experiential type of learning (Gautheron, 2014). Nature-based education uses outdoor settings as a context for delivery of curricular lessons (Edlund, 2001). Nature-Based Learning is described in various ways in the research literature because it is a more general concept compared to other educational concepts involving nature." p.328 | 1. Canada, 4 years 2. USA, NA | 1. Beattie 2015 2. Zimmerman 2013 |
|  | Outdoor teaching | | 1. "The value of outdoor teaching lies in its direct contact experiences that are impractical in regular teaching and that facilitate an experiential learning process. As written by Sharp in 1943, “that which ought and can best be taught inside the classroom should there be taught, and that which can best be learned through experience dealing directly with native materials and real-life situations outside the school should there be learned.” Outdoor teaching is the most widely implemented and effective method for enabling students to directly interact with their environment and acquire direct experience, which develops their attitude toward and values regarding the environment (Sharp, 1952). The National Audubon Society Manual of Outdoor Interpretation (Shomon, 1968) promotes the following conservation education goals: (1) obtainment of related knowledge from nature; (2) an understanding of conservation, and a development of outdoor skills on the basis of knowledge acquired from nature; (3) stimulation of interest in and understanding of nature; (4) shaping of appropriate attitudes on the basis of first-hand outdoor learning experiences (i.e., environmental ethics); (5) determination to engage in environmental conservation; and (6) performance of judicious conservation actions as required." p.3265 | 1. Taiwan, students | 1. Liu 2017 |
|  | Place-based education/  learning | | 1. "One field of outdoor education is the so called place-based approach. Place-based education also emphasises the cultural and social learning about children’s environment. It states that education should prepare pupils to live and work in harmony with their environment. They have to learn the ecological aspects and long term effects of human actions on nature." pg 4301. 2. "...‘place-based’ approach can lead to beneficial states of social capital generation within the learning group, the school and the community more broadly" pg. 185 3. "Using place-based education as a theoretical guide (e.g. Smith, 2002; Woodhouse and Knapp, 2000), data were reduced into three main themes: (a) lived experience; (b) connections to place and to community; and (c) learning, growth and development." pg 377 | 1. Hungary/Serbia, Teachers of children 6-10 years 2. Singapore, NA 3. Canada, 4-5 years | 1. Borsos 2018 2. Atencio 2015 3. Coe 2017 |
|  | | | | | |
| *Learning* | | |  |  |  |
|  | Accidental/incidental learning | | 1. "Incidental learning can cause relatively permanent changes in the way one thinks; accidental learning occurs as a result of experience." p.21 | 1. USA, NA | 1. Lyons 2005 |
|  | Active learning | | 1. "Outdoor instruction is an ideal place for active learning. The outdoors pulls at every sense making it virtually impossible to have a passive experience. A natural setting also invites curiosity, promotes multisensory learning, and provides the rich variety that middle school students need." pg 36 2. " Fantasy and role play, building and creating (large- and small-scale), games" pg 378, Table1 | 1. NA, Middle school students 2. Canada, 4-5 years | 1. Broda 2002 2. Coe 2017 |
|  | Adventure learning | | 1. ''...hybrid distance education approach that provides students with opportunities to explore real-world issues through authentic learning experiences within collaborative learning environments. AL (adventure learning) is grounded in two major theoretical approaches to learning—experiential learning and inquiry-based learning. The AL approach to design, development, and ultimately learning is based upon the understanding that experience rather than osmosis guides meaningful learning experiences.'' p.25 2. (adventure-based learning) "Adventure based learning is a sequence of highly structured physical activities and periods of reflections (ie debriefs) with the aim to promote personal and social development." pg. 27 3. "direct, active, and engaging learning experiences that involve the whole person and have real consequences’ p.3 | 1. USA, 9-11 years 2. USA, Grade 7-8 3. USA, 11-14 years | 1. Doering 2008 2. Stuhr 2015 3. Surtherland 2014 |
|  | Associative learning (perspectives) | | 1. Key features: "Learning as acquiring competence where learners: acquire knowledge by building associations between different concepts; gain skills by building progressively complex actions from component skills." pg 91   Pedagogical approaches: "Focus on competences; Routines of organised activity; Progressive difficulty; Clear goals and feedback; Individualised pathways matched to the individual’s prior performance" pg 91 | 1. UK, NA | 1. Dillon 2005 |
|  | Child-initiated learning outdoors | | 1. "At this point the teachers also began to introduce child-initiated learning in the outdoor environments of their settings. The kinds of activities undertaken outdoors varied and incorporated free play with natural resources (e.g. Schools A, F and H); growing vegetables (School C); problem-solving activities such as building an enclosure for the ducks (School B); and more structured investigations – for example, of snails (School D), air/wind (School E) and flight (School G)." p.216 | 1. UK, 4-7 years | 1. Maynard 2013 |
|  | Constructive learning | | 1. Key features: "Learning as achieving understanding where learners actively construct new ideas: by building and testing hypotheses; through collaborative activities and/or through dialogue." pg 91   Pedagogical approaches: "Interactive environments for knowledge building; Activities that encourage experimentation and discovery of principles; Support for reflection and evaluation; Interactive environments for knowledge building; Activities that encourage collaboration and shared expression of ideas; Support for reflection, peer review and evaluation." pg 91 | 1. UK, NA | 1. Dillon 2005 |
|  | Creative and playful learning | | 1. (within the PLE setting) " Creative and playful learning in the PLE setting refers to (1) learning that allows, stimulates and promotes learner creativity and knowledge co-creation, (2) learning through designing content for the PLE by using new technology, and (3) learning through a variety of playful and physical activities – hands-on and body-on – which take place in the PLE. Hence, learning is not only related to academic achievements, but also to all actions that take into account the person as a whole – body, mind and spirit – and the role of cultural tools (Säljö, 2004, 2005, 2006; Vygotsky, 1978; Wells & Claxton, 2002)" p.2 | 1. Finlandm 7-12 years | 1. Kangas 2010 |
|  | Cooperative learning | | 1. " student’s interpersonal skills and understanding of expectations are emphasized in cooperative learning." pg.380 | 1. Sweden, Grade 7-9 | 1. Fagerstam 2018 |
|  | Explorative learning | | 1. "We take ‘learning’ to mean a holistic process of explorative learning. Learning by discovery denotes a learning process in which the primary focus is on the learner and his or her own activities rather than on the teacher and the processes by which knowledge is imparted. The role of the teacher is to offer learning arrangements, which enable children to discover themselves and their environment (Huber, 2009). In the outdoor school, learning by discovery can occur in both formal and informal learning processes" p215 | 1. Germany, 7-8 years | 1. Sahrakhiz 2018 |
|  | Experiential learning | | 1. "...learning by doing..." pg 7 2. "...outdoor experience has to be a key component of a contextualized, place-based EE [environmental education]." pg 2 3. Examples: "...use data they have gathered outdoors to construct graphs, tables, and charts; find patterns occurring in nature and incorporate; them into their own creative works; explore the concept of camouflage by going outside to find real examples; use indirect measurement techniques to find the height of buildings or trees; explore the concept of texture in art by experiencing textures in nature; do team building activities outside; use natural objects as writing prompts; use a cemetery visit to spark an interest in local history; go out to the school grounds to explain concepts such as habitat and biodiversity..." pg 35 4. "the adult as an overseer, rather than an instructor, afforded children the opportunity to develop their social connection with peers, and for many children this was one of the most important aspects of their experience." (also effective learning) pg 32; "Experiential learning theory defines effective learning as ‘the process whereby knowledge is created through the transformation of experience. Knowledge results from the combination of grasping and transforming experience’, thus experiential approaches to learning tend to be those which are taken outside of the classroom and through which children are engaged actively in a learning experience." pg 35 5. "Experiential learning can be based on both real work/life experiences (e.g., working on a current project) and structured experiences that simulate or approximate real work/life (e.g., using a ﬂight simulator or engaging in a sexual harassment exercise, involving the abuse of distributing playing cards). Its range is enormous. It applies to content that is technical/hard (e.g., operating equipment) or non-technical/soft (e.g., selling skills). Moreover, experiential activity can be used for learning that is cognitive (under-standing information/concepts), behavioral (developing skills), and affective (examining beliefs)." p.8 6. (experiential environmental learning) "...engages children in learning through play, drama, story-telling and problem-solving. As Payne (2006) emphasises, “a strong presence of the acting, sensing, perceiving and ‘doing’ body”should be in experiential learning. Children are immersed in a narrative journey that takes them into the forest where they are involved in hands-on, sensory activities such as exploring the forest’s microstructure using magnifying devices, and constructing with natural materials." pg. 85 7. "As Gass (1993, p. 4) suggests, ‘Experiential learning is predicated on the belief that change occurs when people are placed outside positions of comfort (homeostasis) ... and into states of dissonance. In these states, participants are challenged by the adaptations necessary to reach equilibrium. Reaching these self-directed states necessitates ... resultant growth and learning." p.54 8. "John Dewey’s experiential learning theory describes an experience as a transactional activity; learning is not merely seen as a passive act of receiving, but involves action on the learner’s part. As Dewey himself put it, ‘To “learn from experience” is to make a backward and forward connection between what we do to things and what we enjoy or suffer from things in consequence’ (1916, p. 140)." p.110 9. "My working definition of experiential education: it promotes learning through direct experience, often outside the classroom, at times not directly related to academic courses, frequently not graded, and sometimes not mediated through language or academic discourse and practice." p.26 10. Experiential learning. Outdoor education field experiences often provide students with an opportunity to demonstrate their skills and abilities in ways they might not otherwise be able in a traditional classroom (Fagerstam, 2014). Such experiences allow students to expand on the knowledge they have learned in the classroom with application directly in an outdoor setting (Kisiel, 2005; O’Brien, 2009)." p. 326 11. "Experiential learning can be based on both real work/life experiences (e.g., working on a current project) and structured experiences that simulate or approximate real work/life (e.g., using a ﬂight simulator or engaging in a sexual harassment exercise, involving the abuse of distributing playing cards). Its range is enormous. It applies to content that is technical/hard (e.g., operating equipment) or non-technical/soft (e.g., selling skills). Moreover, experiential activity can be used for learning that is cognitive (under-standing information/concepts), behavioral (developing skills), and affective (examining beliefs)." p.8 | 1. USA, NA 2. USA, NA 3. NA, Middle school students 4. UK, 4-9 years 5. UK, NA 6. Australia, 4-5 years 7. Finland, pupils 8. Sweden, Grade 6 9. USA, children & adolescents 10. Multiple (review), NA 11. UK, 8-11 years | 1. Auer 2008 2. Bentley 2010 3. Broda 2002 4. Coates 2019 5. Dunkley 2018 6. Gambino 2009 7. Karppinen 2012 8. Manni 2017 9. Mackenzie 2013 10. Ray 2019 11. Turtle 2015 |
|  | Environmental learning | | 1. "...learning which accrues from an engagement with the environment or environmental ideas. Some important categories are : sharing the joy and fulfilment of nature, studying the process of nature, help achieve conservation of nature, utilizing nature as a metaphor for preferred social order, etc." p. 11-12 2. "...learning which accrues from an engagement with the environment or environmental ideas. Some important categories are : sharing the joy and fulfilment of nature, studying the process of nature, help achieve conservation of nature, utilizing nature as a metaphor for preferred social order, etc." p. 11-12 | 1. UK, 8-11 years 2. USA, 18-35 years | 1. Turtle 2015 2. Vadala 2007 |
|  | Formal learning | | 1. Children have contact with nature during structured activities in schools, preschools, and child care centers, or during outdoor ﬁeld trips." pp. 2 2. "Formal learning denotes learning processes that take place methodically and in specially designed educational settings. From the learner’s perspective, formal learning is goal-driven and geared towards formal qualifications and certifications (Overwien, 2007)." p.215 | 1. USA, NA 2. Germany, 8-9 years | 1. Jordan 2019 2. Sahrakhiz 2018 |
|  | Free choice learning | | 1. "Free-choice learning as presented in the preceding articles, usually occurs in and through informal learning settings or sectors such as museums, zoos, nature centres, the Internet, television, books and field trips. These free-choice learning settings provide learners with opportunities for direct experience with real objects, people or places. Learning in free-choice settings is voluntary, often socially mediated, and stimulated by the needs and interests of the learner; visitors come alone, in small or family groups of mixed sexes, ages and subject expertise, with very diverse learning styles and prior learning experiences (see for example Anderson, 1995; Falk et al., 1995; Hooper-Greenhill, 1995; Falk & Dierking, 2001; Falk & Dierking, 2001; Packer & Ballantyne, 2002). The social mediation of learning emphasizes its situatedness within a context and that it involves an open process of interaction with the environment. The objectives of free-choice learning are to encourage change in learning about the environment, to improve levels of interest and to increase the learner’s knowledge through contextual cues from the outside world. Free-choice learning offers exposure to practical and recreational as well as intellectual learning and, as noted above, is presented through a wide range of media as well as through conversations with friends and family." p.297 | 1. Canada, children | 1. Kola-Olusanya 2005 |
|  | Holistic learning | | 1. "...beyond preparing for tests and examinations..." pg. 183   Example: "...holistic learning aims found in the new Scottish national curriculum document, the ‘Curriculum for Excellence’..." pg. 185 | 1. Singapore, NR | 1. Atencio 2015 |
|  | Indoor field trip-based learning | | 1. "Science or art centers, museums, heritage temples." pp. 329 | 1. Hong Kong, NA | 1. Jong 2016 |
|  | Informal learning | | 1. "during children’s free play or discovery in nature in their yards, near their homes, in green schoolyards, on the naturalized grounds of child care centers, or in any other natural area." pp. 2 2. "Learning that takes place outside of a classroom environment, or rather ‘the sum of activities that comprise the time individuals are not in the formal classroom in the presence of a teacher.’" pp. 270-71 3. "Following Dohmen (2001, p. 19), informal learning is understood as denoting conscious and unconscious learning processes that are often unintentional and casually initiated. These processes also occur within the school context, (e.g. during peer interactions conducted parallel to the teaching activities) (Harring, Witte, & Burger, 2016, p. 18)." p.215 | 1. USA, NA 2. NA, 15-18 years 3. Germany, 8-9 years | 1. Jordan 2019 2. Jose 2017 3. Sahrakhiz 2018 |
|  | Inquiry-based learning | | 1. For example, in relation to the hydro-cycle: "Inquiry topics included ones such as: ‘‘What happens to the rain falling to the earth?’’ and ‘‘From where did the river’s water come from?’’" pg. 544; Can occur both indoors and outdoors. | 1. Israel, 4^th^ grade | 1. Assaraf 2010 |
|  | Learning in-nature | | 1. Examples: "Digging in the earth, looking for bugs, balancing on logs, jumping in puddles, singing songs, building fires, catching fish, planting a garden, observing wildlife on a nature walk, and rolling in the mud." | 1. USA, K-8 children | 1. Ashmann 2018 |
|  | Learning with-nature | | 1. Examples: "...add nature to a playground, use a natural playscape, have students make observations out the classroom window, or read a nature-based story..." | 1. USA, K-8 children | 1. Ashmann 2018 |
|  | Learning outside the classroom (LOtC) | | 1. "Learning Outside the Classroom (LOtC) is the use of places other than the classroom for teaching and learning. It is about getting children and young people out and about, providing them with challenging, exciting and different experiences to help them learn."   LOtC can take place: on or immediately surrounding the school grounds, hetitage sites, farms, sacred spaces, natural environment.   1. ''...education ‘in’ the outdoors (outdoor activities), ‘through’ the outdoors (perso- nal and social development) and ‘about’ the outdoors (environmental education). Effective outdoor learning needs to move away from fragmented, episodic arrangements towards more ongoing sustained place-based engagements whereby children negotiate what is learned.'' p.50 | 1. UK, NA 2. USA, NA | 1. Council for learning outside the classroom 2019 2. Dolan 2016 |
|  | Learning through play | | 1. "playing at FS was in fact also an opportunity to learn—both in terms of applying their existing knowledge and generating new skills and knowledge" pg 29 | 1. UK, 4-9 years | 1. Coates 2019 |
|  | Nature based learning | | 1. "Nature-based learning, or learning through exposure to nature and nature-based activities, occurs in natural settings and where elements of nature have been brought into built environments, suchasplants, animals, and water. It encompasses the acquisition of knowledge, skills, values, attitudes, and behaviors in realms including, but not limited to, academic achievement, personal development, and environmental stewardship. It includes learning about the natural world, but extends to engagement in any subject, skill or interest while in natural surroundings. NBL can occur with varying degrees of guidance or structure, across the age span, alone or with others, and in urban, suburban, rural, and wilderness settings. NBL occurs in informal, non-formal, and formal settings." pp.2 2. "Nature-Based Learning is often grouped with similar concepts such as place-based education, environmental education, outdoor education and environment-based education (Louv, 2008). Despite subtle differences among them, they all emphasize a first-hand, experiential type of learning (Gautheron, 2014). Nature-based education uses outdoor settings as a context for delivery of curricular lessons (Edlund, 2001). Nature-Based Learning is described in various ways in the research literature because it is a more general concept compared to other educational concepts involving nature." p.328 | 1. USA, NA 2. USA, NA | 1. Jordan 2019 2. Zimmerman 2013 |
|  | Nature learning through school grounds | | 1. '...education in the environment which is about providing children with opportunities to have contact with nature to foster wonder, empathy and love for the outdoors; education about the environment which involves activities occurring in the environment; and education for the environment which refers to taking action to care for the environment.'' p.26 | 1. Australia, 2-6 years | 1. Dowdell 2011 |
|  | Non-formal learning | | 1. "Siurala (2006) defines non-formal learning as a voluntary, situational and experiential learning process which is not easy to break down into measurable didactic phases leading to a clear-cut quantifiable certificate or a learning result (p. 12). Moreover, according to Siurala (2006), non-formal education is learner-centred, emphasizing intrinsic motivation, the usefulness of knowledge and critical thinking. He also argues that non-formal education can be an autonomous field of learning, but it can also be used as an alternative to formal learning or as complementary learning." p.27 | 1. UK, 6-12 years | 1. Stan 2009 |
|  | Outdoor field trip-based learning | | 1. "Class visits to outdoor sites with specific social, cultural, geographical, and/or heritatge setting." pp. 329 | 1. Hong Kong, NA | 1. Jong 2016 |
|  | Outdoor learning | | 1. "...outdoor learning activities involve many kinds of teaching and learning activities that take place outside the classroom."   "...outdoor learning is commonly associated with learning sustainability; meaning that, learning is developed from the students’ ability in managing their futures in a sustainable way."   1. "...involves practical and experiential activities conducted outdoors in school grounds and in other nearby locations...learning can be curricular-based, cognitive and related to indoor learning contexts" pg. 182   "...can encompass cross-disciplinary learning processes, as well as learning focused on pupils’ social, emotional and moral development." pg. 182  "...outdoor learning can also invoke more engaging reflective and problem-solving learning experiences, so that students can develop in more personally meaningful ways..." pg. 184  "...outdoor learning can be reframed in quite local terms in order to provide a viable space where more constructivist and student-driven learning can occur." pg. 185   1. (Outdoor based learning model) "...this type of nature-based or outdoor-learning can be thought of as the Forest School approach." pg 38 2. "Waite and Pratt (2011) proposed a relational model for outdoor learning that evokes the interaction of three key dimensions—child, others and space. According to this model, as children explore the space, their actions will progressively affect the environment. The child does not merely react to space but is capable of transforming it or using different elements according to his/her needs and interests. Simultaneously, the main features of the space influence the action possibilities of children, creating obstacles or opportunities for play. Also, teachers, family members and other children’s attitudes towards outdoor play will have an impact in how space is perceived and explored, considering that the child is not indifferent to what others do, say or think. At a macro level, a set of cultural norms, expectations, broad national guidelines, and standards also affect the outdoor learning expzimmermaeriences." pg 290 3. (also, udeskole theory/practice/teaching) "Figure 1. Curriculum domains and methods used to explore the nature of udeskole theory and practice in a Danish context." pg 201; Jordet’s ‘didactics of udeskole’. Jordet (2002a)   "...udeskole involves ‘regular activities outside the classroom’ and ‘integrated training where activities outof-doors and indoors are closely linked together’...comprising 10–20% of the teaching (i.e. half to one day per week)...focus on concrete experience and pupil activity... action-orientated type of teaching." pg 206  "...indoor and outdoor activities are supposed to be closely related..." pg 207  "‘The pupils learn in an authentic context: that is, they learn about nature in nature, about society in the society and about the local environment in the local environment’" pg 207  Took place in the following locations: green space, school grounds, museums, cultural/public institutions, nature schools, factories, etc. pg 211   1. (learning in the outdoors) "learning "in" the outdoors-the concept of using the outdoors as a magnificent audio-visual tool to facilitate learning in a wide range of content areas" pg 35 2. "...encompasses a spectrum of curricular school activities that take place in the natural environment within school grounds or in the context of the local area. This ranges from broad nature-based learning such as Forest Schools, residential trips and outdoor adventure, to learning programmes tailored specifically to the core curriculum. This huge variation in the practice and understanding of outdoor learning means that the evidence base, whilst growing, shows huge variability in terms of the duration and type of outdoor learning offered, the target population involved and the outcome measures assessed." p.2 3. "...encompasses a spectrum of curricular school activities that take place in the natural environment within school grounds or in the context of the local area. This ranges from broad nature-based learning such as Forest Schools, residential trips and outdoor adventure, to learning programmes tailored specifically to the core curriculum. This huge variation in the practice and understanding of outdoor learning means that the evidence base, whilst growing, shows huge variability in terms of the duration and type of outdoor learning offered, the target population involved and the outcome measures assessed." p.2 4. "outdoor learning, which is underpinned by play-pedagogy" pg 24 5. "Outdoor learning in its broadest sense takes place frequently in schools but much of this is only just ‘outside’; in other words, school grounds activity.... there is ‘much less [outdoor learning] actually away from the school." pg 180; "outdoor learning (especially ‘adventure’ outdoor learning) is often residential learning too" pg 172 6. "We define outdoor learning outcomes as changes in thinking, feeling and/or behaviour resulting directly or indirectly from outdoor education." pg 20 7. "...encompasses a spectrum of curricular school activities that take place in the natural environment within school grounds or in the context of the local area. This ranges from broad nature-based learning such as Forest Schools, residential trips and outdoor adventure, to learning programmes tailored specifically to the core curriculum. This huge variation in the practice and understanding of outdoor learning means that the evidence base, whilst growing, shows huge variability in terms of the duration and type of outdoor learning offered, the target population involved and the outcome measures assessed." p.2 8. ''...education ‘in’ the outdoors (outdoor activities), ‘through’ the outdoors (perso- nal and social development) and ‘about’ the outdoors (environmental education). Effective outdoor learning needs to move away from fragmented, episodic arrangements towards more ongoing sustained place-based engagements whereby children negotiate what is learned.'' p.50 9. "...encompasses a spectrum of curricular school activities that take place in the natural environment within school grounds or in the context of the local area. This ranges from broad nature-based learning such as Forest Schools, residential trips and outdoor adventure, to learning programmes tailored specifically to the core curriculum. This huge variation in the practice and understanding of outdoor learning means that the evidence base, whilst growing, shows huge variability in terms of the duration and type of outdoor learning offered, the target population involved and the outcome measures assessed." p.2 10. "...encompasses a spectrum of curricular school activities that take place in the natural environment within school grounds or in the context of the local area. This ranges from broad nature-based learning such as Forest Schools, residential trips and outdoor adventure, to learning programmes tailored specifically to the core curriculum. This huge variation in the practice and understanding of outdoor learning means that the evidence base, whilst growing, shows huge variability in terms of the duration and type of outdoor learning offered, the target population involved and the outcome measures assessed." p.2 11. "learning biology in an outdoor environment." pg.56; "...at specific places away from ordinary school surroundings, as field trips or visits to outdoor/environmental education centres." pg.58 12. (school-based outdoor learning) "A term used to describe regular outdoor learning that mainly uses the surrounding environments for learning, is ‘school-based outdoor learning’...When the term outdoor learning is used in this study it refers to school-based regular outdoor learning." pg.379 13. "to learn in an outdoor environment." pg.69 "outdoor aspects of learning are integrated into a broader learning experience (Rickinson et al., 2004)." pg.71 14. "Learning in the outdoors is only one ‘pedagogical site’—that is, a ‘site(s) that have (has) the power to teach, to engage “learners” in meaning making practices that they use to make sense of their worlds and their selves and thereby inﬂuence how they act on themselves and others’" pp. 184 15. "… there is clear development of 'personal traits such as honesty, trust, loyalty, compassion or care for nature in outdoor education." pp. 146; “For outdoor based programs to be experiential, their educator must also be mindful of the 'principle of continuity' in which the learning occurs in relation to past learning and future endeavors it is not a detached moment without context outside of time" pp.147 16. “Outdoor Learning is a broad term that includes discovery, experimentation, learning about and connecting to the natural world, and engaging in environmental and adventure activities.”; “Outdoor learning involves the transformation of knowledge, skills, attitudes and behaviours through direct engagement with the outdoor environment for the personal and social benefit of individuals, families, society and the planet.”; “Outdoor Learning is more than adventure sports or simply taking what could happen indoors outside. It can be found in a wide range of environments and situations, including personal adventures, outdoor recreation, informal education; non-formal settings, such as in clubs, Scouts and Guides; and formal settings such as those found in schools, colleges and universities.” Outdoor learning experiences are described in detail at the following webpage: https://www.outdoor-learning.org/Good-Practice/Research-Resources/About-Outdoor-Learning 17. For example; multi-day expeditions, woodland and coastal learning, youth or community activity courses, school residential trips, outdoor and adventure sports coaching, sail training experiences. 18. " Outdoors learning is generally described under the umbrella of theoretical perspectives of informal learning, which is “the most commonly applied term for the science learning that occurs outside the traditional, formal school realm” (Dierking, Falk, Rennie, Anderson, & Ellenbogen, 2003, p. 108). The term informal learning is based on the belief that learning occurs from various experiences and “is an organic, dynamic, never-ending, and holistic phenomenon of constructing personal meaning” (Dierking et al., 2003, p. 109). In 1999, the National Association for Research in Science Teaching (NARST) formed an Ad Hoc Committee in Informal Learning with the charge to clarify what constitutes informal learning (Rennie, Feher, Dierking, & Falk, 2003). According to the committee, the characteristics of informal learning include the following:   • Informal learning occurs out of school, is self-motivated, and is guided by learners’ needs and interests.  • It is strongly socio-culturally mediated.  • It is a cumulative process involving connections and reinforcement between the variety of learning experiences a person encounters in life (Rennie et al., 2003).  However, while we borrow this definition, we also use it in combination with the model proposed by Koliopoulos (2005) to refer to learning that occurs outside any physical context of schooling, which could include formal instructional activities, real-world activities, and/or the combination of both, defined as non-formal learning. According to this model, non-formal activities are organized outside the formal system and they also relate to extra-curriculum activities that “might become part of the curriculum and in a way that enhances the teacher’s role” (Koliopoulos, 2003, p. 86)." p.66   1. "Learning experiences conducted in the outdoors." p.28 2. "Curriculum stimulated by outdoor learning includes vocabulary development, motivation for learning, engagement to tasks, working scientifically skills, proficiency using geography fieldwork tools, a transfer of knowledge to written work, fine and gross motor skill development, and creativity completing artworks." p.54 3. "Outdoor learning, particularly in Early Years Foundation Stage (EYFS) (DfE 2013b) can be just playing outside with classroom resources or a structured activity that is usually adult directed (Bilton 2010)." p.177 4. "outdoor learning experiences contribute to students’ skills in ‘reading nature’ as a way to understand and value ecosystems and natural habitats." p.108 5. "Outdoor learning encompasses a spectrum of curricular school activities that take place in the natural environment within school grounds or in the context of the local area. This ranges from broad nature-based learning such as Forest Schools, residential trips and outdoor adventure, to learning programmes tailored specifically to the core curriculum." p. 2 6. "Cognitively, children’s outdoor learning is linked to making observations, investigations, and hypotheses (Thomas & Harding, 2011). The sensory experience of being outdoors encourages greater observation and sparks curiosity and questioning of the surrounding promoting cognitive learning (Thomas & Harding, 2011)." p.28-29 7. (school based) "Here, the use of the outdoor environment is seen as both teacher-directed activities and children’s free play-based activities, whether in schoolyards, playgrounds, or on the closest neighbourhoods of the schools. This is often referred to as school-based outdoor learning (Fägerstam 2013). (Hereafter, we use the terms schoolyard and playground somewhat interchangeably, or we use schoolyard when referred to compulsory schools and playground when referring to preschools.)" p.1 8. "Outdoor learning is one of the most practical educational methods for teaching a unique natural phenomenon in the world (Tan, Liu, & Chang, 2007). " p.139 9. "Our interest in outdoor learning is particularly focused upon the value of an authentic experience of animals and plants in their natural environment as part of formal (or informal) education related to nature, ecology, and environmental studies (e.g. Scott, Churchill, Grassam & Scott, 2011; Scott & Boyd, 2012; and Scott et al., 2012)." p.47 10. "to refer to learning and teaching that occurs in nature" pg 247 11. ''... is a complex combination of meaningful experiences that probably result in much environmental learning. Children manipulate objects, search for and capture animals, climb, and play with nature and with each other. Whether in interstitial or wild places, play seems to take on the characteristic of what Kyttä (2006) termed Bullerby affordances (child-friendly environments).'' p.14 12. ''...represents the deliberate learner-centred educational offer, where context, both social and environmental, play a significant role. The outdoors often foster greater co-construction of learning less determined by schooling norms which still often foreground acquisition of skills and knowledge through transmission teaching.'' p.869 13. "Outdoor learning encompasses a spectrum of curricular school activities that take place in the natural environment within school grounds or in the context of the local area. This ranges from broad nature-based learning such as Forest Schools, residential trips and outdoor adventure, to learning programmes tailored specifically to the core curriculum. This huge variation in the practice and understanding of outdoor learning means that the evidence base, whilst growing, shows huge variability in terms of the duration and type of outdoor learning offered, the target population involved and the outcome measures assessed." p.2 14. "...encompasses a spectrum of curricular school activities that take place in the natural environment within school grounds or in the context of the local area. This ranges from broad nature-based learning such as Forest Schools, residential trips and outdoor adventure, to learning programmes tailored specifically to the core curriculum. This huge variation in the practice and understanding of outdoor learning means that the evidence base, whilst growing, shows huge variability in terms of the duration and type of outdoor learning offered, the target population involved and the outcome measures assessed." p.2 15. "...encompasses a spectrum of curricular school activities that take place in the natural environment within school grounds or in the context of the local area. This ranges from broad nature-based learning such as Forest Schools, residential trips and outdoor adventure, to learning programmes tailored specifically to the core curriculum. This huge variation in the practice and understanding of outdoor learning means that the evidence base, whilst growing, shows huge variability in terms of the duration and type of outdoor learning offered, the target population involved and the outcome measures assessed." p.2 16. "...encompasses a spectrum of curricular school activities that take place in the natural environment within school grounds or in the context of the local area. This ranges from broad nature-based learning such as Forest Schools, residential trips and outdoor adventure, to learning programmes tailored specifically to the core curriculum. This huge variation in the practice and understanding of outdoor learning means that the evidence base, whilst growing, shows huge variability in terms of the duration and type of outdoor learning offered, the target population involved and the outcome measures assessed." p.2 | 1. Indonesia, Secondary school children 2. Singapore, NR 3. Canada, 4 years 4. Portugal, 4 months-10 years 5. Denmark, 27-62 years 6. NA, Middle school students 7. USA, NA 8. USA, NA 9. UK, 4-9 years 10. UK, 11-13 years 11. UK, NA 12. UK, 9-10 years 13. USA, NA 14. USA, 8-9 years 15. USA, NA 16. Sweden, 13-15 years 17. Sweden, Grade 7-9 18. UK, 7-9 years 19. UK, 6-10 years 20. Canada, NA 21. NA, NA 22. Cyprus, 18-35 years 23. Ireland, 10-12 years 24. Australia, 5-6 years 25. UK, Nursery class 26. Sweden, Grade 6 27. UK, 9-11 years 28. USA, NA 29. Iceland, 3-13 years 30. Finland, 7-8 years 31. UK, 10-11 years 32. NA, NA 33. USA, 18-35 years 34. Multiple, children 35. Denmark, NA 36. Turkey, 58-66 months old 37. USA, NA 38. USA, NA | 1. Asmara 2016 2. Atencio 2015 3. Beattie 2015 4. Bento 2018 5. Bentsen 2012 6. Broda 2002 7. Zimmerman 2014 8. Zimmerman 2013 9. Coates 2019 10. Cramp 2008 11. Dillon 2005 12. Dismore 2005 13. Dolan 2016 14. Eick 2012 15. Eick 2013 16. Fagerstam 2013 17. Fagerstam 2018 18. Harris 2019 19. Humberstone 2012 20. Humphreys 2018 21. Institute for Outdoor Learning n.d. 22. Kadis 2008 23. Kerr 2016 24. Lloyd 2018 25. Mackinder 2017 26. Manni 2017 27. Marchant 2019 28. McClintic 2015 29. Nordahl 2016 30. Rikala 2015 31. Scott 2013 32. Tal 2009 33. Vadala 2007 34. Waite 2016 35. Wistoft 2013 36. Yildirim 2017 37. Zimmerman 2013 38. Zimmerman 2014 |
|  | Physically active learning | | 1. Example: "children described FS as moving from one physically active task to the next" pg 31 | 1. UK, 4-9 years | 1. Coates 2019 |
|  | Place-based learning | | 1. "Place-based learning theory, which considers the importance of connecting learners with their community by anchoring pedagogy within the context of the local natural and social ecosystems (Ardoin, 2006; Gruenewald & Smith, 2014)" p.386 | 1. USA, 8-11 years | 1. McClain 2016 |
|  | Play-based learning | | 1. "Play based learning activites provide multiple ways for children to learn a variety of different skills and concepts. They allow children the opportunities to learn relevent skills and feel competent about their ability to learn." pp. 36 | 1. NA, NA | 1. Isenberg 2002 |
|  | Service learning | | 1. "Service learning, a proven, research-based teaching approach, continues to gain momentum as a valued pedagogy. Far from being a random "let's just do it" approach that usually looks like community service and lacks the benefits of academic connections, service learning has a framework that supports both the acquisition of knowledge and the application of new and prior knowledge to situations that improve communities while showing quantifiable gains and verifiable effects." p.15 | 1. USA, school students | 1. Kaye, 2012 |
|  | Situated learning | | 1. Key features: "Learning as social practice where learners: develop their identity through participation in and with specific communities and practices." pg 91   Pedagogical approaches: "Participation in social practices of enquiry and learning; Support for development of learning skills; Dialogue to facilitate the development of learning relationships." pg 91 | 1. UK, NA | 1. Dillon 2005 |
|  | Sociocultural science learning | | 1. "Sociocultural science learning includes a focus on the practices of science, such as honing observation skills (Eberbach & Crowley, 2009), collaborating about science to refine understandings (Roschelle, 1992), and making thinking visible through conversation with others (Collins, Brown, & Holum, 1991)." p.386 | 1. USA, 8-11 years | 1. McClain 2016 |
|  | Student-centred learning | | 1. "emphasize learning as a sociocultural activity where students learn together and from each other. Problem-solving in real-life activities, where students can take responsibility and make choices is emphasized in student-centred learning...autonomy is also characteristic of student-centred learning, and they conclude that autonomy could be considered as being automatically embedded in student-centred learning." pg.380 | 1. Sweden, G 7-9 | 1. Fargerstam 2018 |
|  | Wilderness-based experiential learning | | 1. Wilderness-based experiential learning involves primal experiences, investigation, improvisation, teamwork and problem solving." p.13 | 1. Canada, students | 1. Savole 2008 |
|  | | | | | |
| *Education* | | | | | |
|  | Adventure-based education | | 1. Example: Wilderness rites of passage program.   "A wilderness rites of passage programme as an adventure based education medium conducts a variety of physically or psychologically demanding outdoor activities in a remote and unfamiliar natural setting. The process engages experiential learning in a group setting and employs real and/or perceived physical or psychological risk." pg 65  Note: a rite of passage is defined as " a rite accompanying any kind of change in “social state, age, place, or life cycle stage, such as birth, puberty, marriage, or death”... The rites of passage were intended to prepare the youth for future membership within a society, and for accepting responsibility to participate actively towards maintaining and developing the society." pg 66   1. A key element of outdoor education. pg 185   "...typically offered in an ‘off-site’ and expert-driven manner..." pg. 186   1. (adventure education) "Model of adventure education in four levels: (1) play - person is working below ability; (2) adventure - person is working in their comfort zone; (3) Frontier Adventure - person is pushing their limits, learning is taking place; however they are still within safety limits; (4) Misadventure - person working over person ability and safety limit, no learning taking place and injury likely to occur." pp. 32 2. ''Adventure education is a form of experiential learning typically associated with activities involving risk, from cooperative games such as raft building to high-adventure activities such as rock climbing...Fundamentally, adventure education is a branch of experiential education and it should therefore centre upon the use of activities that are both engaging and compelling. If activities lack appropriate challenge they will be neither compelling nor engaging... It is based on the philosophy that individuals learn best through direct experience rather than more passive forms of education, such as classroom-based study.'' p.1, 28 and 63 | 1. South Africa, < 23 years 2. Singapore, NA 3. NA, NA 4. Denmark, NA | 1. Bosch 2010 2. Atencio 2015 3. Chapman 2000 4. Wistoft 2013 |
|  | Education outside the classroom (EOtC) | | 1. "...represents a variety of practices." 2. Takes place in "the nearby environment, on the school grounds and in nature and green spaces, as much as in the community (police stations and factories) or places of culture (churches, museums and libraries)". 3. "It can broadly be defined as relocating standard curriculum teaching to places outside the buildings and walls of the schools for a single or a few days per week as a supplement to indoor classroom teaching. Places used in EOtC include forests, school gardens, and museums." pg 210   "EOtC often includes activities described as child-led, hands-on problem solving and experimentation, peer collaboration, and inductive learning approaches, as well as physical activity, games, and play. Although EOtC can be regarded as a school-based health initiative integrated into academic curricular teaching, EOtC is distinguished by not involving explicit health education" pg 210   1. "Education Outside the Classroom (EOtC) is a teaching model in which teachers in primary and secondary schools relocate some of their curriculum-based teaching activities from the classroom to places outside the school's buildings. It takes place in locations close by such as parks, or in remote places that require transportation, such as forest, museums or factore; however, it is predominately conducted in nature and green places. EOtC is practiced in most subjects and is often interdisciplinary. The learning activities in EOtC are often led by students, who use their bodies and senses in collaborative work in "real-world" contexts with the purpose of obtaining concrete and personal experiences." pg 22-23 2. "Integrating evidence-based health promotion with schools’ main aims and objectives in such a way that teachers and students experience them as “add-ins” rather than addons may help the implementation. Education Outside the Classroom (EOTC), also called Learning or Teaching Outside the Classroom, [18] is an example of such an add-in, or holistic school-based health-promotion strategy, as it aims to promote learning, PA, social relations, motivation, and well-being [19]. EOTC activities are characterized by teachers using the local environment when teaching specific subjects and curriculum areas; for example, measuring and calculating the volume of trees in mathematics, writing poems in and about nature for language-related tasks, or visiting historically significant places in history education [18]. EOTC often involves innovative teaching methods, child-led approaches to problem-solving, experimentation, cooperation, PA, and play [19–21]. It is an educational approach characterized by action-centred and thematic learning processes involving outdoor activities [22], with the aim of promoting learning through practical observation and the use of one’s body and senses in authentic situations, and through the interaction between one’s actions and thoughts [22, 23]." p.2 3. (also outdoor school) "In Scandinavian primary schools, the regular relocation of teaching to places outside the school buildings has expanded from a grassroots movement to an increasingly widespread practice (Barfod, Ernst, Mygind, & Bentsen, 2016; Bentsen, Jensen, Mygind, & Randrup, 2010; Waite, 2011). Bentsen et al. (2010) described this practice as regular education outside the classroom (EOtC) (i.e. udeskole [literally meaning ‘outdoor school’]) and conceptualised it as “compulsory educational activities outside of school on a regular basis, e.g. one day weekly or fortnightly.” p.42 4. "Education outside the classroom (EOtC) is an example of an ‘add-in’ and holistic school-based PA promotion strategy as it aims to promote, in addition to PA, learning, social relations, motivation, and wellbeing [16]. EOtC changes the physical classroom to whatever is chosen by the teacher, such as a green area close to the school, and thereby offers teachers the possibility to use different pedagogy and in many cases provides extra space for children to be active [17, 18]. It can therefore be regarded as an educational approach to MI. " p.2 | 1. Denmark, NA 2. Denmark, NA 3. Denmark, 9-12 years 4. Denmark, 9-12 years 5. Denmark, 9-13 years 6. Denmark, Grade 3-6 7. Netherland, 17-22 years | 1. Barfod 2016 2. Barfod 2018 3. Mads 2019 4. Mads 2018 5. Neilson 2016 6. Otte 2019 7. Schreuder 2014 |
|  | Early childhood environmental education | | 1. " A range of programmatic approaches exist that support ECEE’s blended focus on early childhood and environmental education from forest kindergartens and nature-based preschools to more traditional early childhood programs that emphasize connecting with nature. One could look at a range of early childhood programs on a continuum from a sole focus on the social/emotional and/or academic education of young children to a sole focus on nature and the environment, with ECEE programs falling in the middle representing a focus on both areas." pg 9   "...developing an environmentally literate society who is motivated and committed to prevent and solve environmental challenges, the task of environmental education for young children focuses on emotional attachment to the natural world and the need “to forge the bond between children and nature" pg 11  "Ruth Wilson identified the following as key components of early childhood environmental education: (a) the development of a sense of wonder, (b) appreciation for the beauty and mystery of the natural world, (c) opportunities to experience the joy and closeness to nature, (d) respect for other creatures, (e) the development of problem-solving skills, and (f) the development of interest and appreciation in the world around us." pg 11  Figure 1, "Emphasis on: environmental literacy and sustainability." pg 10 | 1. NA, early years | 1. Carter 2016 |
|  | Education for sustainable development | | 1. "... an innovation in educational reform that has evolved from the better-known Environmental Education (EE) movement. It is hoped that the new generation will appreciate the need to preserve environmental resources for the present and future generations." pg.78   "Its main activities include tree planting and building knowledge on energy and water saving practices, waste management and the usage of recycled of resources." pg. 83 | 1. Malaysia, Secondary school | 1. Hanifah 2015 |
|  | Environmental education | | 1. "Environmental education encourages children’s curiosity and desire to understand their world, and to learn respect for the environment." 2. " ...provide enough access for pupils to interact with various aspects of environment." pg. 101 3. (early childhood) "Outdoor play is a crucial element of environmental education and outdoor learning…" pg 40   "Boileau (2011) indicated that verbal, cognitive, emotional, and kinesthetic skills are important elements of play, which should be included when developing outdoor learning or environmental education programs for young children." pg 49   1. "Van Weelie and Wals (2002) argue that environmental education has an important role in making biodiversity meaningful and concludes that despite ill-definition, the biodiversity concept allows learners to ‘construct, critique, emancipate and transform their world in an existential way’." pg 15   "...the recognized need to allow children to explore nature in order to build a personal and meaningful relationship with the natural world, an idea that is woven throughout the history of experiential education." pg 15   1. "...includes both cognitive and affective dimensions..." pg 5   "...personal and civic responsibility..." pg 5  "...environmental education is infused in an integrated curriculum that involves outdoor experiential learning." pg 12  Example provided in Figure 1 (Appendix).   1. (informal environmental education) "...programs and experiences developed outside the classroom by institutions and organizations that include: nature centers and environmental education centers, children’s and natural history museums, zoos and aquaria, botanical gardens and arboreta, parks, and scientific research laboratories; media (print, film, broadcast, the Internet and other electronic forms), and community-based organizations and projects, and youth organizations." pg 11 2. (urban setting) "With a goal and long history of engaging individuals in programming to facilitate the development of environmental literacy, as well as changes in knowledge, skills and behaviors to ultimately improve environmental health and sustainability." pg 2056 3. "Environmental education is defined as "a learning process that increases knowledge and awareness about the environment and develops skills that enable responsible decisions and actions that impact the environment"." pg 35 4. "Environmental education can be understood as the process of gaining awareness of the world around us, with its natural beauty and resources. Ideally, this understanding should not be only theoretical. It should lead the individual to value nature and to seek to preserve it." p.1 5. "Environmental education can be understood as the process of gaining awareness of the world around us, with its natural beauty and resources. Ideally, this understanding should not be only theoretical. It should lead the individual to value nature and to seek to preserve it." p.1 6. "Environmental education can be understood as the process of gaining awareness of the world around us, with its natural beauty and resources. Ideally, this understanding should not be only theoretical. It should lead the individual to value nature and to seek to preserve it." p.1 7. "Environmental education can be understood as the process of gaining awareness of the world around us, with its natural beauty and resources. Ideally, this understanding should not be only theoretical. It should lead the individual to value nature and to seek to preserve it." p.1 8. "Ultimate outcomes of environmental education are to develop evionmentally literate individuals, and responsible environmental behavior of those individuals" pg.2233 9. "child-directed play and exploration [outdoors] " pg.98 10. "can nurture a child’s creativity, sense of wonder, and apprecia- tion of beauty" p.643 11. "Anglo-Saxon tradition, the term outdoor education often concerns adventurous experiences that focus on team-building and development of leadership skills; quite often, such experiences are provided by a purpose-built outdoor education centre" pg.56; "In the Scandinavian context, the term outdoor education most often involves school-based learning outside the classroom, in the nearby natural or cultural landscape or on school grounds, often with a cross-curricular approach" pg.56 12. "Environmental educationists agree that EE should be a continuing learning process through which awareness, knowledge, skills and values are acquired that aid problem solving with a sustainable future in mind for the present and future generations."pg.254 "the communication of scientific knowledge to learners (Knapp and Poff 2001; Zuefle and Beck 1996) and hopefully the accomplishment of changes in [environmental] attitudes and behaviour (Corraliza and Berenguer 2000)."pg.254 13. "Environmental education can be understood as the process of gaining awareness of the world around us, with its natural beauty and resources. Ideally, this understanding should not be only theoretical. It should lead the individual to value nature and to seek to preserve it." p.1 14. a) "the opportunity to bond with the natural world, to learn to love it and feel comfortable in it, before being asked to heal its wounds” pg.129   b)"Research shows that providing children with early opportunities to play in nature has physical (Fjørtoft, 2001) as well as psychological and social benefits, including stimulating fantasy and imaginary activities, promoting discovery and exploration, encouraging risk-taking and the development of environmental competencies, and fostering a sense of autonomy" pg.129   1. "… its components that emphasize the knowledge of 'physucal and ecological' and 'social, cultural and political systems' and 'the appropriate behavioural strategies to apply such knowledge and understanding in order to make sound and effective decisions" pp. 41 2. "Requires a delicate pedological balance of knowledge, values and action in the experience of environmental education in early childhood education." pp 90 3. "Aim is to increase student awareness of the impact of human interaction with natural and social environments, and to engage students into action to address environmental issues. The environment is perceived holistically with the inclusion of all its dimensions: natural, artifical, structured, socioeconomic and historical." pp. 46 (OGG, 2003) 4. " Many studies indicate that outdoor education programs hold high emotional engagement for students and, as a result, increase motivation for the learning taking place in this environment. Experiential involvement in active, in-context, outdoor environmental education is exciting and emotionally engaging for children and consequently leads to deeper and more effective learning " pp. 60 5. . "Outdoor recreation and environmental education claim a number of adventurous founding fathers, men such as John Muir, Teddy Roosevelt, Gifford Pinchot, and David Brower. Twenty years ago outdoor recreation researchers began searching for causes of the apparent under-representation of people of color among outdoor recreationists." pp. 127 6. "help individuals and communities … acquire the knowledge, values, attitudes, and practical skills to participate in a responsible and effective way in anticipating and solving environmental problems, and in the management of the quality of the environment." pp.2   "Education about the environment, which builds awareness, understanding, and the skills necessary to obtain the understanding; education in (or from) the environment, where learning occurs outside of the classroom, e.g. in nature; education for the environment, which has objectives related to nature conservation and sustainable development." pp.2   1. "Some of the major goals of environmental education (EE)are to increase awareness of environmental problems, motivate action through awareness, and develop a commitment to solve and prevent local and global problems. " pp. 269 2. Environmental education (including those offered outdoors) - In 1976, The Belgrade Charter: A Global Framework for Environmental Education (UNESCOUNEP, 1976) summarized the goal of environmental education as to develop a world population that is aware of, and concerned about, environmental and associated problems and that has the knowledge, skills, attitudes, motivations, and commitment to work individually and collectively toward solutions of current problems and the prevention of new ones. Term: Outdoor learning - Outdoors learning is generally described under the umbrella of theoretical perspectives of informal learning, which is “the most commonly applied term for the science learning that occurs outside the traditional, formal school realm” (Dierking, Falk, Rennie, Anderson, & Ellenbogen, 2003, p. 108). 3. "The task of environmental education for young children is to forge the bond between children and nature” (NAAEE, 2010, p. 4)". p.73 4. "Environmental education involves the three dimensions of self, society, and nature. The purpose of environmental education is to promote active and positive attitudes as well as proactive participation in environmental actions. This is achieved by improving awareness of personal responsibility toward environmental problems, thus facilitating personal understanding and growth. The interaction between society and the natural environment is also explored. The discussion and resolution of daily concerns helps students develop life skills. The goals of the environmental education curricula are as follows (Education, 2008): (1) awareness of and sensitivity toward the environment; (2) conceptual knowledge regarding the environment; (3) suitable values and attitudes toward the environment; (4) environmental action skills; and (5) environmental action experiences." p.3264 5. "Non-Aboriginal scholars of outdoor and environmental education often allude to the possibility of learning from Aboriginal epistemologies and their orientations to the natural world (Henderson, 2007; Sharkawy, 2008; Vikander, 2007). Many other contemporary scholars call for greater attention to a sense of place, a feeling of being at home in and connected to one’s geographical surroundings, in outdoor and environmental education (Brookes, 2006; Curthoys, 2007; Lugg, 2004; Wattchow, 2006)." p.43 6. " A noteworthy definition is that provided by Stapp (1969), who underlines that EE is an education that aims at developing citizens with knowledge related to the biophysical environment and its problems, citizens that are not only aware of how they can help, so those problems can be solved, but are also willing to contribute to solving them (Papadimitriou, 1998)"pg.417; " EE promotes development for economic, social, political and ecological interrelationship in urban and rural areas. It provides each person the opportunity to acquire knowledge, values, attitudes, commitment and skills needed to protect and improve the environment. It helps to create new patterns of behaviour for individuals, groups, societies, for the environment (Kalaitzidis & Ouzounis, 1999)." pg. 418; " EE is defined as a lifelong learning process that leads to the creation of informed and active citizens who have creative problem-solving skills, have scientific and social education and are committed to engaging in responsible individual and/or collective actions. These actions are believed to promote an environmentally sound and economically prosperous future" (Kalaitzidis & Ouzounis, 1999) pg. 418 7. "Environmental education can be understood as the process of gaining awareness of the world around us, with its natural beauty and resources. Ideally, this understanding should not be only theoretical. It should lead the individual to value nature and to seek to preserve it." p.1 8. "Environmental education can be understood as the process of gaining awareness of the world around us, with its natural beauty and resources. Ideally, this understanding should not be only theoretical. It should lead the individual to value nature and to seek to preserve it." p.1 9. "Environmental education can be understood as the process of gaining awareness of the world around us, with its natural beauty and resources. Ideally, this understanding should not be only theoretical. It should lead the individual to value nature and to seek to preserve it." p.1 10. (outdoor) "Environmental education can be understood as the process of gaining awareness of the world around us, with its natural beauty and resources. Ideally, this understanding should not be only theoretical. It should lead the individual to value nature and to seek to preserve it." p.1 11. "Environmental education can be understood as the process of gaining awareness of the world around us, with its natural beauty and resources. Ideally, this understanding should not be only theoretical. It should lead the individual to value nature and to seek to preserve it." p.1 12. "has a much more explicit focus on the outdoor environment with aims geared towards enhancing students’ awareness and knowledge of both the environment and their relationship with the environment." p.252 | 1. Australia, 6-7 years 2. Indonesia, NR 3. Canada, 4 years 4. Sweden & Norway, Adults (Sweden), 1-6 years (Norway) 5. USA, NA 6. USA, NA 7. USA, 10-13 years 8. NA, Middle school students 9. USA, NA 10. USA, NA 11. Germany, 10-12 years 12. UK, NA 13. Turkey, 8-13 years 14. Sweden, NA 15. USA, NA 16. Australia, 13-18 years 17. South Africa, NA 18. USA, 4-5 years 19. USA, NA 20. USA, Mean 6^th^ Grade 21. USA, 8 months-6 years 22. Greece, 2.5-6 years 23. USA, 7-8^th^ graders 24. USA, NA 25. Finland, NA 26. NA, 15-18 years 27. Cyprus, 18-35 months old 28. USA, 1-3 years 29. Taiwan, students 30. Canada, NA 31. UK, NA 32. Turkey, NA 33. USA, 4-5 years 34. USA, NA 35. USA, NA 36. USA, NA 37. New Zealand, 11-18 years | 1. Alexander 2010 2. Atmodiwirjo 2013 3. Beattie 2015 4. Beery 2018 5. Bentley 2010 6. Bentley 2010 7. Beyer 2015 8. Broda 2002 9. Zimmerman 2014 10. Zimmerman 2013 11. Dissner 2010 12. Dunkley 2018 13. Erdogan 2011 14. Ericsson 2014 15. Ernst 2012 16. Fagerstam 2014 17. Ferreira 2012 18. Zamani 2016 19. Green 2016 20. Hougham 2018 21. Hunter 2017 22. Iscos 2015 23. James 2017 24. James 1996 25. Jeronen 2009 26. Jose 2017 27. Kadis 2008 28. Kharod 2015 29. Liu 2017 30. Lowan 2009 31. Stavrianos 2016 32. Yilmaz 2016 33. Zamani 2016 34. Zimmerman 2016 35. Zimmerman 2014 36. Zimmerman 2013 37. Zink 2008 |
|  | Experiential education | | 1. "Experiential education is the primary pedagogical tool utilised at the school and is commonly defined as learning that involves participants in direct experience andfocused reflection (Association of Experiential Education, n.d.). There are many models of reflection in experiential education, but they generally follow John Dewey’s (1938/1997)3 model where there is an action directed by an idea, an event or action with consequences takes place, followed by reviewing or reflecting to identify what occurred and what can be taken from this experience onto other events." p.211 | 1. Germany, 11-13 years | 1. Eckstein 2015 |
|  | Field trips | | 1. "Field trips can take a wide range of configurations, which may include mixtures of venues, pedagogical approaches, and learning goals (Bell et al., 2009; Rennie, 2007; Rickinson et al., 2004). One commonly reported field trip configuration is a class or school visit to a museum, science center, or some other established informal educational center or institution (Bamberger & Tal, 2008; Cox-Petersen et al., 2003; Flexer & Borun, 1984; Rennie & McClafferty, 1996; Stronck, 1983; Wright, 1980). [...] Another common field trip configuration involves visits to outdoor or field locations, such as wildlife preserves, in which ecological or environmental education can take place (Knapp & Barrie, 2001; Knapp & Poff, 2001; Rickinson et al., 2004). Field trips to these locations frequently require students to engage in experiences that are intended to increase their knowledge about the environment particular to the region in which the destination is located (Bell et al., 2009). In contrast to museums and science centers, field trips to parks, forests, watersheds, preserves, gardens, grasslands, or other natural settings are typically less structured and less predictable, which increases the range of possible learning outcomes (Orion & Hofstein, 1994). The potential for a wide variation of student learning outcomes resulting from environmental field trips provides the warrant for continuing to study the influence of these activities on student attitudes and retention of event related content." p. 221 2. "Field trips are usually arranged by schools, have educational purposes, and take place in interactive settings all of which have been shown to increase learning." pg. 246 | 1. USA, Grade 6 2. NA, NA | 1. Nadelson 2012 2. Tal 2009 |
|  | Nature education | | 1. "giving meaning to organism and goes beyond to give meaning to nature as a whole. Nature education help develop environmental awareness, sense of resposibility, environmental knowledge, affect , and thus, responsible behavior". Pg.2233 2. (education in nature) "Western (UK, USA/Canada, Australia/New Zealand) ‘outdoor education’ traditions often emphasize adventure and challenge education, inseparable from physicallydemanding activities in natural environments (Wurdinger, 1994). The activity is often foremost with a ‘destination’ to be reached. In contrast the characteristic features of some of Eastern Europe and Scandinavia’s (involving friluftsliv) ‘education in nature’ traditions are strongly connected to a ‘journey’ (Henderson & Vikender, 2007). In Czech, education in nature (výchova v pˇrírodˇe) involves turistika activities, travelling for fun and playing games with the aim of learning about nature and its beauty. Turcová et al. (2005) point out that while the English term ‘outdoor education’ is accepted in Czech, as výchova v pˇrírodˇe, its translation is ‘education in nature’." p.68 3. A recognised inspiration for nature education is the European Forest School movement (Elliott and Chancellor 2014; MacQuarrie, Nugent, and Warden 2015); While the concept of ‘unstructured play’ is fundamental in nature education, this does not mean that it is without curriculum. Kelly and White (2013) pointed out that educators draw on their expertise in the natural setting to ‘uphold their pedagogical obligations’ (50).   It is essential that educators identify learning moments during nature sessions and then situate the children’s experiences in the curriculum. This includes mapping learning against the curriculum framework and documenting learning experiences (Elliott and Chancellor 2014). | 1. Turkey, 8-13 years 2. New Zealand, adults 3. Australia & New Zealand, early childhood, Kinder | 1. Erdogan 2011 2. Martin 2011 3. Masters 2018 |
|  | Outdoor early childhood education and care | | 1. "outdoor ECEC environments provide increased opportunities for active play through activity promoting play equipment, natural features and varying surfaces when compared with indoor environments." pg 2 | 1. Australia, 2-4 years | 1. Christian 2019 |
|  | Outdoor education | | 1. "...an idea and put into practice with a range of diverse locations, focuses and promising learning outcomes."   "Focus of outdoor education can include:  · learning on the subject of nature, for instance, in an ecological or horticultural study  · learning concerning to civilization, for example, in community-based gardening initiatives or conservation projects  · learning in relation to nature-society interactions, for example, in visits to outdoor nature centre or areas of outstanding natural setting  · learning on character, for example, in personal fulfillment through challenging adventure education  · learning on the subject of cooperation with others, for example, in small-group fieldwork or residential experience  · learning new skills, for example, through fieldwork or practical activities in school grounds  · learning about practical conservation, for example, through focused activities in the countryside or on city farms  · learning with reference to influencing civilization, for example, by campaigning on controversial issues  · learning research skills, for example, through action research on field."  "Intended outcomes such experiences can include:  · knowledge, for example, geographical processes, ecology or the technique for increase food  · attitudes towards, for example, intensive stock rearing, access to the countryside or fair trade  · values about, for example, the value of the environment, one’s relationship to it, or biodiversity loss  · personal development, for example, self-confidence, knowing fact from value, increasing personal effectiveness  · activities and behaviors, for example, pro-environment actions or making a personal commitment  · behaviors, for example, identification of species with map reading  · skills, for example, in clearing or removing invasive alien species  · social development, for example, working with others, or reducing racism  · improved competence efficiently to get an enquiry on one’s own or other’s work with children."   1. "...entails pupils’ meaningful engagement with lessons that are transferrable to broader social and educational contexts." pg. 182 2. "...learning in and about the natural environment as well as understanding sustainability issues through outdoor activities can occur in conjunction with key curricular frameworks." pg. 184   "...rely heavily upon the elements of risk and adventure, aligned with ideals of social and personal development." pg. 185This is a learning process that takes place outdoors in the nature, or it can also occur in a constructed environment, for example in the school yard. It is based on Dewey’s philosophy that the world is constantly changing and pupils can become familiar with it through experiences that are stimulated by the problems of everyday life." pg 430  "Outdoor education has three forms depending on how much time the pupils spend outside of the school: walks, visits and excursions. The shortest form is the walk, which usually lasts an entire school class (45 min). The visit takes longer, and its destination can be a natural locality (park, forest, river bank etc.) or an institution (museum, zoo etc.) (Brown 2008). The excursion is the longest form; lasting from half a day to a whole day. The destination of an excursion is farther away from the school; it can be a locality outside of the settlement." pg 430  "Outdoor education is an informal way of teaching, thereby; helping to pupils come closer to nature and the plants and animals living in it. In locations outside of the class room, the learning process is aided by the possibility of use greater of senses. In the case of outdoor education, pupils gain direct experiences during the learning process. In these circumstances, pupils are given a chance to observe and use their senses in the process of learning. They can smell, listen or even, in certain cases, taste the things in the nature. Knowledge gained in that way lasts longer and has stronger motivational power than knowledge that pupils gained in the classroom aided by classical, formal methods. The task of the teacher is to utilise the pupils’ curiosity and interest; and so build on the so-called inquiry method." pg 431   1. "Outdoor environmental education represents a distinctive and important active ‘mode of learning’". pg 899   Example: " This outdoor education company provides school fieldtrips that focus on the exploration of the surrounding natural environment." pg 902   1. "Outdoor education is a general term describing a number of fields of practice that are often associated with personal and social health education (PSHE), personal and social development (PSD) and related terms." pg 223 2. Outdoor education is a part of the physical education curriculum. pg. 182 3. Similar to "Friluftsliv" but "not exact translation". pg. 270; "Friluftsliv entails living and engaging in physical activity in the open air during leisure time in order to achieve a change of setting and to gain experience of our natural surroundings. Friluftsliv is ‘outdoor recreation with its heart within the land and linked to a tradition of being and learning with the land’." 4. ""Outdoor education means learning in and for the outdoors. It is a means of curriculum extension and enrichment through outdoor experiences". This definition includes the concept of using the outdoors as an instructional tool (learning in the outdoors) as well as the concept of teaching skills, attitudes, and appreciations necessary for the intelligent use of the outdoors (learning for the outdoors)." pg 35   "Although certainly embracing the concept of environmental stewardship, outdoor education as a teaching tool applies to a wide range of content areas and topics and is not narrowly limited only to environmental concerns." pg 35  "...outdoor education is not tied to any specific discipline, nor is it limited to nature study. It is appropriate for teaching a multitude of concepts from a wide range of curricular areas..." pg 35   1. "...six features of an outdoor education programme: 1. Education occurs outdoor. It does not have to be offered in a classroom. 2. Participants get directly involved in activities. 3. Real objects are used. Activities are implemented by using real objects and the senses. 4. Instead of memorising available knowledge, relations between objects or events are discovered and described. 5. Learning through practice and experience activates more than one sense. 6. Since the environment of an outdoor education programme is different from that of an enclosed classroom, children find outdoor activities interesting and fun." p.2 2. "Outdoor education is known in most European countries as a teaching method in the ordinary educational system for children and young persons. The method is often used as a complement to the regular classroom situation." pg 99   "Outdoor education is often, as in the Scandinavian countries, strongly connected to learning in nature and to environmental contexts. Concepts such as ‘learning by doing’ or ‘hands-on activities’ are often used to express the basic idea of outdoor education." pg 100  "Gair also states that outdoor education is an ongoing process where mutual support is standard and that it is rare that young persons are excluded in these contexts. " pg 100  "Hopkins and Putnam (1993) highlight three components of importance in outdoor education: self, others and the natural environment. ‘Self’ includes the individual participant and how the adventurous activities will enhance and increase the self-conception and self-awareness. ‘Others’ includes group development and co-operation in the group, as well as forging effective underlying social structures. ‘Natural environment’ provides adventure education with many challenges at the same time as the participants gain knowledge and awareness of nature." pg 101  "Outdoor education can therefore be regarded as a complement to traditional teaching methods, involving the use of outdoor environments to broaden and deepen the understanding of nature, social life and special phenomena in a wider perspective." pg 101  "My definition of outdoors means out in nature, in the open-air, and out-of-doors..." pg 102; Examples: "...working with maths in the garden." pg 101; "...gardening, working in the forest, farming, sawing wood, taking care of pets such as chickens or dogs, reclaiming rubbish in the environment, washing cars, clearing snow, delivering post to pre-schools and schools, and helping senior citizens. " pg 108; "...nature excursions, took daily walks and conducted nature circles (which were defined as outdoor education)." pg 108   1. "Multidisciplinary approach to learning which advocates outdoor direct experiences as extensions of school classroom." pp. 3 2. "Outdoor education contributes to learning in a range of areas, including: science and geography fieldwork; physical education; learning through outdoor play, particularly in the early years; history and citizenship, through visits to museums and heritage sites; art and design, through visits to galleries and experiences of the built environment; environmental and countryside education, and education for sustainable development; practical or vocational skills that cannot be practised in a classroom environment; group activities that build self-confidence and social skills; and the use of the environment as a tool to enrich the curriculum across subject areas." pg 35 3. "emphasises personal and social development, that is, learning in and through the outdoors. Where outdoors is is predominantly regarded as either comprising empty sites where context-free personal development can occur or as some sort of abstract form of nature" p. 252-254 4. ''emphasises personal and social development, that is, learning in and through the outdoors. Where outdoors is is predominantly regarded as either comprising empty sites where context-free personal development can occur or as some sort of abstract form of nature" p. 252-254 5. "[The Teacher is] in the background, [the students] are active." pg.8 6. "[When] school lessons are moved outside the school building." pg.78 7. "Outdoor education is an environment-focused educational approach characterized by action-centred and thematic learning processes frequently involving outdoor activities". Pg. 65; "...It aims to foster learning through the interactions between emotions, actions and thoughts, based on practical observation in authentic situations." pg. 65 8. "...allows children to experience nature, face challenges, and in the process develop teamworking and negotiating skills, engage in creative thinking, critically analyse situations, and develop problem-solving skills." pg. 274 9. "outdoor activities of a recreational or educational kind." pg.76 10. "Any outdoor context affords a different set of opportunities for affective, sensory, and participatory engagement in a learning than are possibe in a school classroom" pp. 41 11. "Outdoor education theoretically encompasses particular potential educational resources, forms and methods that are based not on the transmission of information, but on mediating experiences. In various cultures, outdoor education carries speciﬁc features of local tradition" pp. 321; Outdoor education translates in Czech to výchova v přírodě and adventure education to dobrodružná výchova. Výchova v přírodě has been translated recently to ‘education in nature’ involving turistika (active movement on foot, bikes, canoes or skies involving other outdoor and cultural activities, like learning about nature and local history, art, music and entertainment, mushroom and berry picking) to distinguish between the Czech approach and outdoor education. Another term used in the Czech Republic is zážitková pedagogika ), often translated to experiential education. pp. 221 12. "Outdoor education and experiential learning allows for, and actually encourages, different avenues of thought and methods of learning." p.24 13. "Non-Aboriginal scholars of outdoor and environmental education often allude to the possibility of learning from Aboriginal epistemologies and their orientations to the natural world (Henderson, 2007; Sharkawy, 2008; Vikander, 2007). Many other contemporary scholars call for greater attention to a sense of place, a feeling of being at home in and connected to one’s geographical surroundings, in outdoor and environmental education (Brookes, 2006; Curthoys, 2007; Lugg, 2004; Wattchow, 2006)." p.43 14. "Western (UK, USA/Canada, Australia/New Zealand) ‘outdoor education’ traditions often emphasize adventure and challenge education, inseparable from physically demanding activities in natural environments (Wurdinger, 1994). The activity is often foremost with a ‘destination’ to be reached. In contrast the characteristic features of some of Eastern Europe and Scandinavia’s (involving friluftsliv) ‘education in nature’ traditions are strongly connected to a ‘journey’ (Henderson & Vikender, 2007). In Czech, education in nature (výchova v pˇrírodˇe) involves turistika activities, travelling for fun and playing games with the aim of learning about nature and its beauty. Turcová et al. (2005) point out that while the English term ‘outdoor education’ is accepted in Czech, as výchova v pˇrírodˇe, its translation is ‘education in nature’." p.68 15. (outdoor ed programs) "Outdoor education programs go by a variety of names, including environmental education, conservation education, adventure education, experiential education, and environmental interpretation (Ford, 1986). They take place in nearly every geographic location, and are sponsored by various levels of educational institutions, state and local government agencies, and private entrepreneurs. Such programs commonly have no nationally standardized curriculum or measures of competency or knowledge (Ford, 1986)." p.428 16. "Outdoor education is a complex subject. Though it involves learning, outdoor education also contains elements of freedom, fun and recreation. Nevertheless, it is still education.". P.44 17. "Outdoor education is an experiential method for learning which occurs primarily in the outdoors and which emphasizes relationships between people and natural resources (Priest, 1986). " p.140 18. "Outdoor education (OE) has been defined as an education in, about and for the outdoors. OE is both a place and a topic, and has been linked to understanding and caring for oneself and others " pg. 420 19. ''Outdoor education is a form of learning system conducted in both outdoor and indoor settings which involves challenging or adventure activities as a medium to foster individual personal and social growth...purpose of assisting individuals in gaining a greater appreciation of nature and their relationship to it, and the focus of personal development through interactions with others, and responsible use of the natural environment.'' p.61 20. ''...School camping programmes are the most common form of Outdoor Education occurring in all types of schools….although Outdoor Education occurs at all levels of schooling, the majority occurs at years 9 and 10 especially in Government schools. The outdoor education program focuses on : group cooperation, improved self esteem, increased responsibility, fitness, survival skills, recreation/leisure skills and environmental appreciation and knowledge." p.45-47 21. "...six features of an outdoor education programme: 1. Education occurs outdoor. It does not have to be offered in a classroom. 2. Participants get directly involved in activities. 3. Real objects are used. Activities are implemented by using real objects and the senses. 4. Instead of memorising available knowledge, relations between objects or events are discovered and described. 5. Learning through practice and experience activates more than one sense. 6. Since the environment of an outdoor education programme is different from that of an enclosed classroom, children find outdoor activities interesting and fun." p.2 22. "...six features of an outdoor education programme: 1. Education occurs outdoor. It does not have to be offered in a classroom. 2. Participants get directly involved in activities. 3. Real objects are used. Activities are implemented by using real objects and the senses. 4. Instead of memorising available knowledge, relations between objects or events are discovered and described. 5. Learning through practice and experience activates more than one sense. 6. Since the environment of an outdoor education programme is different from that of an enclosed classroom, children find outdoor activities interesting and fun." p.2 23. "...six features of an outdoor education programme: 1. Education occurs outdoor. It does not have to be offered in a classroom. 2. Participants get directly involved in activities. 3. Real objects are used. Activities are implemented by using real objects and the senses. 4. Instead of memorising available knowledge, relations between objects or events are discovered and described. 5. Learning through practice and experience activates more than one sense. 6. Since the environment of an outdoor education programme is different from that of an enclosed classroom, children find outdoor activities interesting and fun." p.2 24. "emphasises personal and social development, that is, learning in and through the outdoors. Where outdoors is is predominantly regarded as either comprising empty sites where context-free personal development can occur or as some sort of abstract form of nature" p. 252-254 | 1. Indonesia, secondary school children 2. Singapore, NR 3. Hungary/Serbia, Teachers of children 6-10 years 4. Bangladesh, Singapore, Malaysia,   Germany, 7-18 years   1. UK, NA 2. Singapore, NR 3. Sweden, NA 4. NA, middle school students 5. USA, NA 6. Sweden, Finland, Latvia, Germany , Greece, 0-25 years 7. USA, NA 8. UK, NA 9. UK, 9-10 years 10. USA, NA 11. Sweden, NA 12. Norway, 5^th^ Grade 13. Sweden, preschool-Grade 6 14. UK, NA 15. Scotland, NA 16. Australia, NA 17. Czech Republic, NA 18. Canada, 13 years 19. Canada, NA 20. New Zealand, adults 21. Israel, school students 22. Canada, Grade 5 23. Finland, 7-8 years 24. UK, NA 25. Singapore, 12-16 years 26. Australia, 14-16 years 27. Denmark, NA 28. Turkey, 58-66 months old 29. USA, NA 30. New Zealand, 11-18 years | 1. Asmara 2016 2. Atencio 2015 3. Borsos 2018 4. Braun 2018 5. Allison 2010 6. Atencio 2015 7. Backman 2011 8. Broda 2002 9. Zimmerman 2014 10. Brodin 2009 11. Carlson 2000 12. Dillon 2005 13. Dismore 2005 14. Dolan 2016 15. Ericsson 2012 16. Fiskum 2013 17. Gustafsson 2012 18. Harris 2017 19. Higgins 2000, 2002 20. Hoad 2013s 21. Jirasek 2017 22. Kuntz 2007 23. Lowan 2009 24. Martin 2011 25. Morag 2013 26. Nicholas 1989 27. Rikala 2015 28. Stavrianos 2016 29. Wang 2004 30. Williams 2018 31. Wistoft 2013 32. Yildirim 2017 33. Zimmerman 2016 34. Zink 2008 |
|  | Outdoor education intensity | | 1. "...the magnitude of an experience that falls outside of everyday teaching to possibly include moments of heightened focus, thrill, vigor, or the direct focus of our being-in-the-moment." pg.152; In outdoor education, the intensity is a constant "pathway connection" that exists between teacher and student, resulting in a teaching identity that is weaved with relationships, places of learning, events, memories and reflections; constructing a past, informing the pedagogic present. | 1. Canada, High school | 1. Foran 2005 |
|  | Outdoor adventure education | | 1. "Outdoor adventure education and learning is an alternative method in formal education. It is flexible, experiential-based, non-formal and supportive. It is a constructive ‘expedition’ into oneself and into the social environment and reality. It is a rehabilitative and a ‘holistic’ method to transfer knowledge, personal development and social growth directly in the everyday life and connect them with formal learning by constructivistic and reflective learning theory.[...] It is a sustainable method to help pupils to use their five senses bodily to get into contact with nature, environment and social reality (Karppinen, 1998, 2005, 2008, 2010)" p.58   “Adventure outdoor education + Erlebnispädagogik/therapie + experiential learning pg. 45 two main pedagogical approaches—the German semantic concept of Erlebnispädagogik/Erfahrungs Lernen and the English/American semantic concept of Experiential Education/Learning—which have theoretical basics to understand outdoor adventure pg. 54 In the present study outdoor adventure approach is, in school context, regarded as learning, training, education or even rehabilitation. As Gass (1993, p. 4) suggests, ‘Experiential learning is predicated on the belief that change occurs when people are placed outside positions of comfort (homeostasis) . . . and into states of dissonance. In these states, participants are challenged by the adaptations necessary to reach equilibrium. Reaching these self-directed states necessitates . . . resultant growth and learning”   1. "Outdoor adventure education (sometimes referred to as the “Outward Bound” experience) is an innovative method used by various training programs to enhance participant skills such as collaboration, cooperation, leadership, self-reliance, self-esteem, and communication (Friese, Hendee, & Kinziger, 1998; Herbert, 1998; Sibthorp, 2003). In these day-long programs, participation by groups in experiential exercises and team challenges is typically focused on activities designed to enhance personal and social growth amongst peers in an informal setting. The instructional approach features team activities that are structured to push participants to the edge of their comfort level in order to complete novel problem-solving tasks." p.320-321 2. "outdoor adventure education (OAE). OAE uses remote outdoor environments, activities like backpacking and sea kayaking, and other curricular components (e.g. leadership development) to foster skill development and personal growth (Priest & Gass, 2005). [...] Set within an outdoor educational context that uses adventure activities like backpacking and sea kayaking as a medium for teaching a range of technical, interpersonal and intrapersonal skills, OAE provides powerful opportunities for learning and development. " p.37 | 1. Finland, pupils 2. USA, early childhood 3. USA, 13-18 years | 1. Karppinen 2012 2. Lamorey 2013 3. Richmond 2018 |
|  | Outdoor experiential education | | 1. "Outdoor and Experiential Education (OEE) is a vital learning methodology for today’s children and young people. Its provision of safe, educator-framed and hands-on experiences in outdoor settings provides unique, vital and lasting benefits in terms of education for curriculum and community, education for character, education for wellbeing and education for environment." p.33 2. "Distinctive for its emphasis on the child's natural inquisitiveness, encouragement of learning for everyday life, and promotion of a conneciton between children and their natural environments, outdoor experiential education can encourage children to learn in, through, and about nature in outdoor spaces and on the schoolgrounds." pp.135 | 1. Canada, NA 2. NA, NA | 1. Linney 2007 2. MacDonald 2018 |
|  | Outdoor environmental education | | 1. "...outdoor environmental education of children involves not just lessons about natural phenomena but also, about ethics…" pg. 6   "...instruction directed toward developing a citizenry prepared to live well in a place without destroying it…" p. 6  "...Outdoor environmental education – that subset of environmental learning that involves direct exposure to physical and biological phenomena in the field – addresses not only ‘the facts’ about interactions between people and nature, but also, about the imperative of caring for the environment." p. 6   1. Example: "...involved teaching units concerning the nutrient cycle in temporal forests and rainforests, adaptations of plants and animals to the life in the forest and harmful pollution. All participants worked on the different forest layers, food webs in the forest and ways of sustainable use of ecosystem services" pg 941 2. "Environmental education can be understood as the process of gaining awareness of the world around us, with its natural beauty and resources. Ideally, this understanding should not be only theoretical. It should lead the individual to value nature and to seek to preserve it." p.1 | 1. USA, NA 2. Singapore, 7-18 years 3. USA, NA | 1. Auer 2008 2. Braun 2017 3. Zimmerman 2014 |
|  | Personal and social health education | | 1. "... enable them [individuals] to take an overview of the whole range of values impinging on their lives, to help them find an orientation and direction and to give them some basis for the choices they have to make." pg 225 | 1. UK, NA | 1. Allison 2010 |
|  | Place-based education | | 1. "…usually includes conventional outdoor education and experiential methodologies, such as those advocated by John Dewey, to help students connect with their unique context in the world."pg.149 2. “rooted in the unique history, culture, environment and economy of a particular place. The community provides a context for learning, student work focuses on community needs and interests, and community members serve as resources and partners in every aspect of teaching and learning. The local focus has the power to engage students academically, pairing relevance with rigor, while opening windows to the world and promoting genuine citizenship (The Rural School and Community Trust, 2009).” 3. "Place-based education emphasises using the places where children live, the local environment, and the community in the school curriculum. By this, place-based education addresses one of Dewey’s central concerns that schools should not be isolated from the community around them but rather use it both as physical and social learning environment and participate in it (Smith 2013). Greenwood (2013, 93) argues that place-conscious education can contribute to environmental education ‘that is culturally responsive, and committed to care for land and people, locally and globally’."p.3 | 1. Canada, High school 2. NA, NA 3. Iceland, 3-13 years | 1. Foran 2005 2. Gray 2015 3. Nordahl 2016 |
|  | | | | | |
| *Schools* | | | | | |
|  | All weather schools | | 1. “…‘all-weather schools’ where nature is the classroom and outdoor education the aim.” Pg 101 | 1. Sweden, Finland, Latvia, Germany , Greece, 0-25 years | 1. Brodin 2009 |
|  | Bush schools | | 1. "...an Australian interpretation of the forest school..." pg 29   "students participated once a fortnight for a period of 10 weeks, outside in the local nature reserve. Students would walk down to the reserve at the beginning of lunchtime and return at the end of the school day, allowing approximately three hours outside in the bush reserve. Students would eat lunch and have free time to play and explore within the boundaries of the area. Examples of activities included sandcastle building, running down sand dunes, climbing trees and building a cubby. Additional activities were often included that incorporated other curriculum areas. Examples of additional activities include exploring insects and plant types found in the reserve, using microscopes to observe and discuss findings and measuring tree trunks to calculate the height of trees. Local history was learnt by stories told by staff, and students also wrote about their experiences on return to school, drawing pictures of plants and places within the bush setting." pg 299 | 1. Australia, 10-12 years | 1. Cumming 2015 |
|  | Forest kindergartens | | 1. "Children are encouraged to play, explore, and learn in all kinds of weather in a forest or other natural environment. Adults organize lessons not through highly structured activities, but through assisting students in their learning, using their natural curiosities and exploration as the foundation. The emphasis is on play with toys fashioned from natural objects, such as sticks, small rocks, hardened mud, grasses, etc. Common activities include playing imaginative games, role playing, building structures, counting objects, looking for patterns, and memory games, all with natural materials found in the local environment. Even though many differences exist in the methods between forest kindergartens and school-based kindergartens, the ultimate goal of educating young children remains the same."   "Seven pedagogical principles of practice that guide the forest kindergarten approach to education: 1. A holistic approach to children’s learning and development; 2. Each child is unique and competent; 3. Children are active and interactive learners; 4. Children need real-life, first-hand experiences; 5. Children thrive in child-centered environments; 6. Children need time to experiment and develop independent thinking; 7. Learning comes from social interactions."   1. "The learning environment at the Forest Kindergarten consisted of 190 acres of woodland, with two canvas tents providing an indoor retreat and learning environment for the preschool and Kindergarten children. Typically, children spent the majority of the school day outside in the Canadian woodland, retreating indoors for lunch each day and when children required refuge from the cold or dangerous weather (e.g. hail, torrential downpour). The natural outdoor space provided a unique learning environment, consisting of trees, hills, cliffs, boulders and open areas. In addition, numerous wetland areas were scattered throughout the forest, providing children with access to several lakes, creeks and marshlands." pg 377 2. "In the Scandinavian countries and growing elsewhere, there are efforts to provide these nature experiences for young children through an educational context, using mediums such as Forest Kindergardens." pg 30 3. (also Wardindergarten) "...where children aged 3–6 were able to spend most or all of their time outside before they entered formal schooling". Pg.115 4. (also Waldindergarten) "They must be place based. Ours is a discussion of an educational experiment connected to a local situation. Following the BC Curriculum, (BC Ministry of Education 2017) interacting with the natural setting and the living beings found there, physical exercise and being welcomed to kindergarten/school were put forth as important elements for the Nature Kindergarten." pg. 378 5. "Forest kindergartens have been defined as educational programs which provide daily outdoor experiences for children 3-6 year olds, with limited or no indoor facilities. Children in such programs tend to spend 70-100% of their time outdoors, in a nature immersion experience, in which the curriculum emerges from the daily activities (Fritz, Smyrni, & Roberts, 2014; Kenny, 2013; Moore, 2014; Sobel, 2014; Warden, 2012). Forest kindergartens are sometimes known by other names including “waldkindergartens” or “nature kindergartens” (Fritz et al., 2014; Sobel, 2014; Warden, 2012)." p.33 6. "Forest kindergartens, a common practice in Germany, Scandinavia and other European countries, feature year-round early childhood care in an outdoor environment, regardless of the weather. These forest kindergartens typically have no indoor facility at all, simply a three-sided shelter and meeting place, though most of their activity takes place in unstructured activities in a natural environment. Children are free to build, to climb, to use fantasy play, to interact with the flora and fauna of the forest, all day, every day" pg 1038   "an exclusively outdoor experiential and immersive model’. Three- and four-year-old children are regularly offered a chance for first-hand experiences in nature. Filler explains that ‘These are self-regulated, resilient, physically fit and capable preschool-aged children, who are also learning to navigate risks and make decisions which impact both themselves and their group’ (p. 1)." pg 1038 | 1. USA, K-8 children 2. Canada, 4-5 years 3. USA, 3-6 years 4. Canada, 3-6 years 5. Canada, 3-6 years 6. USA, Kindergarten 7. Multiple, NA | 1. Ashmann 2018 2. Coe 2017 3. Desrochers 2001 4. Elliot 2018 5. Elliot 2017 6. Larimore 2016 7. Brown 2017 |
|  | Forest schools | | 1. "Characteristics of forest schools include “wild spaces”—natural spaces that offer children the promise of interaction, loose parts, and manageable risk—unstructured time to observe and play independently, play and experience as guides for learning, and a sense of community among peers and teachers (Figure 1). Knowledge grows from independent observations and interest, to organic group collaboration, and can then be expanded on and developed by the added knowledge base of the teacher. During a school day, students and teachers may take hikes, observe their surroundings in sit spots, build forts or fairy houses, tell stories, learn outdoor skills, make fires, prepare snacks or tea, make art with leaves and stones, use tools, run, jump, sing, pretend, discuss, and discover. Teachers both facilitate and allow students to create learning experiences, ask questions to help students discover knowledge, promote further research on a topic, and add their expertise or insight to help students progress from the known to the unknown and back again." pg 32   "A forest school provides a safe space for students to experience adventure and develop knowledge independently." pg 32  Example: "...outdoor classroom that includes a fire pit and benches surrounded by berry bushes and trees. I was amazed at how freely so many of my older students took to running around and playing tag, eating berries, and even building a structure from fallen branches! Even a small “wild space” (Knight, 2011, p. 2) was enough for them to explore and move more freely. The goal of this exercise was to encourage play and spark creative energy that my students and I could reference and draw from in future class periods." pg 33  Synonyms introduced: Waldkindergartens (Ashmann 2018), Open-air nursery, I Ur och Skur (Rain or shine school), Wild space, Outdoor classroom   1. "Fostering self-esteem, developing self-confidence, creating independence, learning about the environment, and taking risks have been foregrounded in these programs. Of particular importance is for students to meet both physical and emotional risks and challenges head-on – climbing a tree, balancing on a log, jumping into a pond, etc. It is critical that children learn how to assess risks and take challenges to not only spur on their development, but because these are crucial life skills. The adult’s role is to support and guide the students in how to assess challenges and risks for themselves. The setting is made as safe as reasonably possible, in order to facilitate risk-taking. Trust among students and between an individual student and teacher is central to many components of a forest school." 2. "...offers opportunities for children and young adults to come into regular contact with nature." pg 39   "...which nurtures children’s curiosity and inherent tendencies to learn and explore the world around them, in a natural setting." pg 40  "FS is a specific form of outdoor learning which can be distinguished from other outdoor learning initiatives. Although FS sits underneath the greater umbrella of outdoor education, it has been described as a Bspecialised learning approach" pg 41  "FS is predominantly child-led, with curriculum negotiated between adults and children. It is an approach that can be suitable for learners of all ages, but one which is overwhelmingly used with younger children (Knight 2011). It has the express aim of helping people – predominantly younger children – grow as individuals, develop skills and confidence, as well as healthy relationships amongst themselves and with the environments they live in, especially focusing on more natural environments." pg 42  "FS is a vehicle for curriculum and not a curriculum in itself." pg 42  "The purpose of FS, as described by Waite et al. (2016), can be seen as twofold. On the one hand, FS is a way to increase children’s connections with nature, within the cultural and social context of an ever-urbanized and indoor society. On the other hand, FS aims to increase young children’s motivation to learn, mainly by stimulating their interests." pg 43   1. (also outdoor school) "...school-based outdoor learning as a weekly or biweekly ‘outdoor school’ day for school children..." pg 199   "Udeskole is characterised by compulsory and regular educational activities outside the school buildings, and it takes place in both natural and cultural settings; for example, forests, parks, local communities, factories and farms. Udeskole activities are characterised by teachers making use of the local environment when teaching specific curriculum subjects. They may, for example, illustrate mathematical concepts by measuring and calculating the volume of trees in mathematics, write poems in and about nature for language-related tasks or contextualise history by visiting historically significant places or buildings. Hence, the approach is to work with an academic subject matter or concept in its real, concrete form to facilitate learning and understanding. Teaching and learning activities, however, are often cross-disciplinary." pg 200  "Outdoor schooling is a working method where parts of the everyday life in school [are] moved out of the classroom—into the local environment. Outdoor schooling implies regular activities outside the classroom. The working method gives the pupils the opportunity to use their bodies and senses in learning activities in the real world in order to obtain personal  and concrete experiences. Outdoor schooling allows room for academic activities, communication, social interaction, experience, spontaneity, play, curiosity and fantasy. Outdoor schooling is about activating all the school subjects in an integrated training where activities out-of-doors and indoors are closely linked together. The pupils learn in an authentic context: that is, they learn about nature in nature, about society in the society and aboutthe local environment in the local environment." pg 205  "...udeskole is more than a method; it builds on a fundamental way of thinking and a philosophy about teaching and learning—an understanding that education exists in a social, political and geographical context; for example, ‘... learning activities in the real world to obtain personal and concrete experiences’" pg 206   1. "FS is an approach to learning which is considered ‘informal’ or ‘alternative’ but is blended with traditional curricula to create opportunities for children to experience learning outside of the classroom. In the 2006 Learning outside the classroom manifesto, it was branded as the gold standard in outdoor learning and has been recognised for easing ‘parental hypervigilance and a narrowing of the focus of education’. FS uses natural environments to engage children in a range of play-focused learning activities (e.g. den building, fire lighting, tool use, etc.) intended to develop their problem-solving skills and co-operation abilities; confidence, self-motivation and self-esteem. In the UK, FS is recognised as a distinct programme facilitated by trained and accredited practitioners, although such programmes are being developed internationally. The values which underpin FS are inclusive, child-focused, offering the opportunity to engage in supported risky play and providing a range of play-based activities which foster development beyond academic skills in an outdoor, woodland setting." pg 23   "FS gave them more freedom and increased autonomy. Freedom was perceived in two distinct ways—psychological freedom (freedom from the pressures associated with school) and freedom to make their own choices." pg 2   1. "In the context of the United Kingdom, a forest school is an educational approach to learning facilitated through outdoor experiences that commence in the school grounds and then progress to local bushland or natural parkland settings." pg 297   "A forest school utilises the school grounds as a means of familiarising students with the outdoors when introducing the programme and encourages play in natural spaces within the school grounds. Many of the studies concerning forest schools are focused on the early years of learning and conducted in the United Kingdom. These programmes vary in length and can run continuously over a relatively long period of time but typically run for a minimum of 10 weeks." pg 298  " The forest school approach offers an alternative way of delivering the curriculum and embedding outdoor learning pedagogies into the school’s education framework as a whole. Teaching a range of curriculum areas such as science, mathematics, society and the environment and the arts within the surrounds of the forest or outdoor class, and in the context of the natural environment, is suggested as a further option when structuring a forest school programme. The forest school approach accommodates different learning styles through a variety of activities in a space less defined than a classroom setting. The philosophy underlining the approach is to provide enjoyable learning experiences outside that offer opportunities for risk-taking and decision-making that, in turn, enhance positive attitudes and intrinsic motivation to learn. Activities may include, but are not limited to, creating outdoor learning spaces and play spaces within the school grounds. Further examples of activities that vary among programmes include allowing children to build shelters or cubbies (also known as ‘dens’ and described by Sobel [1993] as a shelter or playhouse built by children for special games or as a secret place), learn to use tools, have free time to explore or simply climb trees." pg 298   1. "Forest School is an approach to outdoor education which offers an alternative teaching environment, to complement the indoor curriculum.…characteristics of forest schools as ‘creative environments’ include: use of a local woodland (therefore ‘wild’) setting; regular, frequent contact in the same setting over a significant period of time; providing freedom to explore using multiple senses and intelligences; time and space for individual learning styles to be recognised and nurtured; a low pupil:adult ratio." pg 85 2. a) "an inspirational process that offers children, young people and adults regular opportunities to achieve and develop confidence and self-esteem through hands on learning experiences in a woodland environment".pg. 720; b) "children in early years settings using the outdoors everyday, all year round as part of their pre-school education." pg.721; c) "a dedicated area of developed woodland and scrub, accessed on a regular basis and in all weathers by Early Years and primary-aged children accompanied by adults, in order to try out ‘hands-on’ learning experiences, some of which involved taking risks." pg.721 3. "The Forest School approach is also equivalent to school-based outdoor learning but with a focus on learning in woodland environments. The Forest School embraces regular curriculum-linked learning, not only focusing on learning about nature and environment but also on subjects such as English, mathematics and science". pg.57 4. "a movement within education in which children engage in regular, repeated outdoor learning facilitated by a qualified forest school leader (Knight 2009)." "Forest school takes place within the context of multiple demands to teach defined curricula and achieve targets for attainment while nurturing children’s physical health and social well-being." pg. 272 5. "...regular and repeated access to the same natural space and emergent, experiential play-based and place-based learning". Pg.340 6. (forest school programs) "… have derived from the Nordic approach to outdoor learning developed in the late 1950s which has traditionaused nature as a learning resource and not simply as a place for children to 'let off steam'." pp.90 7. "Forest schools, yet another early childhood environmental education program type, take children to a nearby natural area on a regular basis for half to a whole day at a time, are more broad in the ages they serve with some even extending beyond early childhood, involve public schools, and have the goal of building a relationship with a particular natural space through regular visits over time (Andrachuk et al., 2014; Maceachren, 2013; Moore, 2014; Warden, 2012). How long these visits occur varies among programs, but Knight (2013) suggests six weeks minimum and adds that many forest school practitioners recommend a minimum of 10 weeks. Either way the emphasis is clearly on several weeks of regular visits not just once or twice a school year, or even a visit once each season." p.33 8. "The professional body, the Forest School Association (FSA 2007), defines Forest School as an: Inspirational process that offers children, young people and adults regular opportunities to achieve and develop confidence and self-esteem through hands on learning experiences in a woodland environment. [...] ‘Forest School’ indicates a session of outdoor activities based on Forest School principles (Knight 2009, 2012, 2013) that aim to ‘encourage and inspire individuals of any age through positive outdoor experiences’ (FSA 2007). [...[ Forest School is a learner-centred pedagogical approach (Knight 2009, 2012, 2013; Williams-Siegfredson 2012), and as such learning should be participant initiated." p.177 9. "Forest School is defined as ‘an inspirational process that offers ALL learners regular opportunities to achieve and develop confidence and self-esteem through hands-on learning experiences in a woodland or natural environment with trees’ (Forest School Association 2017). Waite, Bølling, and Bentsen (2016) outlined typical Forest School characteristics as:   A long-term regular programme of visits;  A woodland or wooded context;  Holistic development of the child;  Qualified Forest Schools practitioners; and  Child-centred processes to encourage a community of learners (Waite, Bølling, And Bentsen 2016 p. 876)" p.234   1. "Maynard (2007b) suggests that the primary aim of Forest School appears to be the development of children’s self-esteem, self-confidence and independence skills. A second aim is to encourage children to appreciate, care for and respect the natural environment. Children normally attend Forest School for a half or whole day a week, regardless of weather conditions and ideally throughout the year, so enabling them to experience the outdoor environment in all seasons. In the initial visits to the woodlands, they are encouraged to explore and play in the natural environment and to take part in games such as ‘one, two, three, where are you?’ (a form of Hide and Seek) as well as practical activities such as hunting for mini-beasts, using mirrors, collecting sticks of different lengths and sharing and acting out stories (Maynard, 2007b)." p.382 2. "Forest School involves regular visits to natural, ‘wild’ environments usually with a trained Forest School leader. Forest Schools have a long tradition in Scandinavia as a way of giving children play and learning experiences in the natural world. By the 1980s, they became an integral part of the Danish early years programme and influenced the development of the Forest School movement in the UK (Knight, 2009; Maynard, 2007). The Forest School movement has grown substantially in recent years and there are now sites in urban as well as rural areas." p. 2011 3. "Forest school is a 'innovative educational approach to outdoor play and learning' (www.forestschool.com), which usually takes place in woodland environment (Nilson et al. 2010)." p. 499 4. "A definition of Forest School has been developed by the Forest School network; it states that it is an ‘inspirational process that offers children, young people and adults regular opportunities to achieve and develop confidence and self-esteem through hands on learning experiences in a woodland environment’ (Murray and O’Brien 2005; Forest Education Initiative 2007)." p.45 5. "Forest School is an approach to education that is derived from observations of Scandinavian early years education. It embraces the outdoors as a learning environment, with regular outdoor sessions, planned around the interests of the children. As Knight (2009) states, ‘it resonates with those of us who spent our childhoods either in woods and field or around an area of streets, messing about with mud and sticks and learning without noticing’ (p. 2). The Forest School approach is defined by the Forest School Association (FSA) (2012) as, … an inspirational process, that offers ALL learners regular opportunities to achieve and develop confidence and self-esteem through hands-on learning experiences in a woodland or natural environment with trees (n.p.)" p.4 6. "Programme of education in the outdoors (rather than about the outdoors). Typically children spend a period of their schooling (ranging from a couple of hours a week to all their time) undertaking outdoor activities. Forest school is both a pedagogy and a physical entity, with the use often being interchanged." p.8 7. "is a regular, hands-on, child-led learning approach, set within a wooded environment, run by a trained school staff leader (or external FS leaders), which within a school setting is curriculum mapped and typically takes place during the school day" p.2 8. (forest school programs) "It seems almost enough that children are given the time and space to play in the woods, to throw armfuls of leaves in the air or watch them falling from trees, or to lie on their backs and watch the clouds. To assess hazards, to identify risk and reduce that risk becomes second nature. But the benefits do not stop when they leave the wood behind. Increase self-esteem, healthy curiosity, stronger physical stamina, cooperative skills and habits of observation have all been recorded in children attending Forest Schools." pp.16 9. At forest school, children attend outdoor learning sessions regularly and repeatedly (e.g. weekly or bi-weekly) for a minimum of six weeks and possibly extending throughout the school year. Children are given freedom to pursue a range of activities such as den building, fire lighting, nature craft, or climbing. Initially aimed at younger children in the early years/foundation stage of education (Davis and Waite 2005), with a focus on learning through play (Fjortoft 2001) it is now being used with older groups of children from across the primary school years, and also with older children with specific needs (see Knight 2009, 2011). Forest school is described as ‘constructivist education’ (O’Brien 2009) with children constructing meaning through interaction with each other and the natural environment. The outdoor learning environment is seen to provide a flexible social space with multiple opportunities for learning and interacting with others.'' p.275-276 10. ''At forest school, children attend outdoor learning sessions regularly and repeatedly (e.g. weekly or bi-weekly) for a minimum of six weeks and possibly extending throughout the school year. Children are given freedom to pursue a range of activities such as den building, fire lighting, nature craft, or climbing. Initially aimed at younger children in the early years/foundation stage of education (Davis and Waite 2005), with a focus on learning through play (Fjortoft 2001) it is now being used with older groups of children from across the primary school years, and also with older children with specific needs (see Knight 2009, 2011). Forest school is described as ‘constructivist education’ (O’Brien 2009) with children constructing meaning through interaction with each other and the natural environment. The outdoor learning environment is seen to provide a flexible social space with multiple opportunities for learning and interacting with others.'' p.275-276 | 1. USA, NA 2. USA, K-8 children 3. Denmark, NA 4. Denmark, 27-62 years 5. UK, 4-9 years 6. Australia, 10-12 years 7. Scotland, NA 8. UK (England), Primary school 9. Australia, 13-18 years 10. UK, NA 11. Canada, NA 12. Australia, NA 13. USA, Kindergarten 14. UK, Nursery class 15. Australia & New Zealand, Early childhood/kinder 16. UK, early years children 17. South Korea & UK (England), 3-4 years 18. UK, 5-15 years 19. UK, 3-13 years 20. UK, 3-7 years 21. Multiple (Delphi), NA 22. UK, 7-9 years 23. NA, NA 24. UK, 8-11 years 25. Multiple, children | 1. Bradshaw 2018 2. Ashmann 2018 3. Barrable 2019 4. Bentsen 2012 5. Coates 2019 6. Cumming 2015 7. Davies 2013 8. Elliot 2015 9. Fagarstam 2014 10. Harris 2017 11. Harwood 2017 12. Jackson-Barret 2018 13. Larimore 2016 14. Mackinder 2017 15. Masters 2018 16. Maynard 2007 17. Nah 2016 18. Nawaz 2014 19. O’Brien 2009 20. Savery 2017 21. Shanahan 2019 22. Trapasso 2018 23. Trotman 2010 24. Turtle 2015 25. Waite 2016 |
|  | Green classroom | | 1. ''...environmental education program that wants to address knowledge, skills and attitude at the same time. It was founded 10 years ago and is visited by about 2,500 preschool children and school students yearly. Learning in the ‘Green Classroom’ is organised in such a way that students encounter animals in their natural habitat, and what they observe is explained and put into context. Questions that arise from these encounters are addressed immediately. The topics that students deal with are about small animals that can be found in the environment of the children.'' p.181-182 | 1. Germany, 10-12 years | 1. Dissner 2010 |
|  | Junkyard kindergarten | | 1. Activities emphasize unstructured, creative exploration with diverse materials. | 1. Israel, 3-6 years | 1. Haas 1996 |
|  | Museum school | | 1. "There is no commonly accepted definition of a museum school, but such schools typically involve partnerships with local institutions of informal learning and offer a curriculum with long-term projects that involve students in working with objects, exhibits, or museums." pg 12 | 1. USA, NA | 1. Bentley 2010 |
|  | Natural classroom | | 1. "The many sights, sounds, smells, and textures of the outdoors make it a natural classroom, and one that fits the exuberant gross motor, construction play, and symbolic play instincts of preschoolers. In fact, research indicates that preschoolers engage in richer symbolic play outdoors than indoors thanks to the relatively greater availability of low-realistic, low-structured, natural materials, spaciousness, and teacher involvement” found outdoors as compared to indoors." pp. 7 | 1. NA, NA | 1. Oliver 2005 |
|  | Nature (based) preschool | | 1. "Nature-based preschools have typically been defined as a licensed early childhood program for 3-5 year olds, with 25-50% of the class day held outside each day, nature as the driving theme of the curriculum, and nature being infused into the indoor spaces (Bailie, 2010; Green Hearts, 2014; Larimore, 2011a, 2011b; Moore, 2014). For clarification, it should be noted that “nature-based preschool” and “nature preschool” are used interchangeably in the literature. Recently, Bailie and Finch provided a definition of nature-based preschools on the Natural Start Alliance website. A detailed and expanded version on the definition is provided in Figure 1. Notice there is no mention of specific ages or time spent outdoors, unlike the definitions of other early childhood environmental education programs." p.32-33 (content of figure 1 is presented in comments!) | 1. USA, Kindergarten | 1. Larimore 2016 |
|  | Nature classes | | 1. "In Denmark udeskole has mainly been practised in natural settings to date and the term nature classes has been used and could be understood as a subset of the term udeskole." pg 32 | 1. Denmark, Norway, & Sweden, 7-16 years | 1. Bentsen 2009 |
|  | Nature kindergarten | | 1. " I spent three days at the kindergartens and tagged along with the kids (children ages 3-5). They spend nearly all day outside. They run up hills, dig in the mud, swing in hammocks, climb trees, and play in living willow huts. They pick berries, play music, cook food, and eat snack, all outside. Everything happens inside a fencedin area. They also have access to adjacent woods for play and exploration." p.67 2. "Nature kindergartens may be considered distinctive early-childhood provisions that use nature environments season round, as a setting and resource for nature-based pedagogies." p.468 3. "Nature kindergartens are specialist childcare institutions that, relative to the majority of early childhood institutions, are based in wilder, more untamed sites. Settings are troves rich in nature’s resources that facilitate exploration and offer diversity for “treasure hunting” (Kramer, 2011; Lewis-Stempel, 2012), as “you can’t just go to your [vegetable] patch and grab it” (Bird, 2014, p. 9). These real settings promote first-hand, season-round, meaningful participation in the “intangible ‘utilities’ associated with nature and experiencing nature” (Henwood & Pidgeon, 2001, p. 136)." p.79 | 1. Scotland, 3-5 years 2. Denmark, Finland, & Scotland, Kindergarten 3. Scotland, Kindergarten | 1. Keeler 2011 2. Nugent 2018 3. Nugent 2015 |
|  | Nature preschool | | 1. "Nature preschools have several reference areas and spend more time away from the preschools while visiting these reference areas. Although great importance is placed on the outdoors in Norwegian preschools (Moser & Martinsen, 2010), the differences between nature preschools and other preschools point to the same conclusion – nature preschools are organized so that children spend more time in natural settings. Nature preschools have made certain adaptions such as rules for the children and organizational schemes. This enables them to use nature as a pedagogical playground and to spend large amounts of undisturbed time in nature." p.20 | 1. Norway, 1-5 years | 1. Lysklette 2017 |
|  | Udeskole/  Uteskole | | 1. "In Denmark and Norway...regular educational activities outside the classroom in the nearby environment (pg. 277) or on school grounds… (pg. 277)” 2. "...udeskole is defined by its duration and place, as well as pedagogical approach. Udeskole is distinct in its regularity, e.g. once or twice a week or every two weeks for an extended period, such as half a year. Thus, it is not an ‘experiential exceptionalism’ but rather a regular variation in everyday practice. The teaching is carried out by the teacher (not outdoor professionals) in—as with EOtC—the nearby environment, on the school grounds and in nature and green spaces, as much as in the community (police stations and factories) or places of culture (churches, museums and libraries). Depending on the curricular goals, appropriate locations are chosen to support pupils’ in-depth understanding of the subject. Moreover, udeskole practice addresses goals encompassed in the curriculum, thereby contributing to the academic content—but also to the motivational, social and health aspects of pupils in compulsory school. Besides the description of time and place, udeskole is based on ‘a progressive reform pedagogical tradition’, with practitioners relying on interpretations by the pedagogical philosopher John Dewey and referring to their teaching as ‘education for life’ and ‘holistic education’. The instructional methods have been described as initiating inquiry-based, problem-solving activities, with pupils reflecting upon their experiences during the activity." 3. "Udeskole is a term that not only refers to a method of teaching but also a movement to redefine school, and a theory about how education should be viewed: an understanding that education exists in a social, economical, political and geographical context. This concept of out-of-school-teaching stems from Norway (Jordet 2007, 1998), where teachers and pupils also use natural surroundings or culture settings, i.e. museums, companies, factories, churches etc as ‘outdoor’ classrooms on a regular basis." pg 32   "...target group of school children aged 7–16, and is characterised by the fact that compulsory educational activities take place outside the walls/buildings of the school and are done on a regular basis (i.e. a day every or every other week) and can take place in nature, local communities, factories, farms etc." pg 32  "Examples of teachers’ and children’s -udeskole-activities could be work within specific subjects and curriculum areas, e.g. mathematics by measuring the height and volume of trees, language by writing poems in and about nature, or history or religion by visiting historical significant places etc, but are very often also cross-disciplinary and cross-curricular activities." pg 32  "The concept of udeskole characterises regular compulsory curriculum-based outdoor teaching and learning in schools and is not captured by concept like forest school, fieldwork and outdoor visits, outdoor adventure education, school ground/community projects etc." pg 40   1. "The Scandinavian concept udeskole (meaning ‘outdoor school’; see Box 1) is a particular type of approach to outdoor learning, which has emerged as a way to engage children with nature through educational contexts." pg 235; "School-based outdoor teaching in the local outdoor environment as an integrated part of the school system is a relatively new form of teaching and learning activity in Scandinavia (i.e. udeskole in Danish; uteskole in Norwegian; utomhuspedagogik in Swedish). The concept was described in a Norwegian context by Jordet (1998), in a Swedish context by Dahlgren and Szczepanski (1998), and recently Bentsen et al. (2009b) described and defined udeskole situated in the socio-cultural context of Danish education and Scandinavian society. Udeskole targets children aged 7–16, and is characterised by compulsory educational activities outside of school on a regularly basis, e.g. one day weekly or fortnightly. Udeskole can take place in both natural and cultural settings, i.e. forests, parks, local communities, factories, farms, etc. (Jordet, 1998, 2007). However, in Denmark udeskole has mainly been practised in natural settings to date, and the term nature classes has been used and could be understood as a subset of the term udeskole (Mygind, 2005). Udeskole activities are characterised by making use of the local environment when teaching specific subjects and curriculum areas by, for example, measuring and calculating the volume of trees in mathematics, writing poems in and about nature when teaching languages, or visiting historical significant places in history education – teaching and learning activities are however often inter-disciplinary. Thus, the approach is often to deal with an academic subject matter or concept in its real concrete form to facilitate learning, motivation, and understanding." pg 236 2. "The Danish model of ‘‘Udeskol’’, education outside the classroom" p.956 3. “…a working method where parts of the everyday life in school is moved out of the classroom – into the local environment. Udeskole implies regular activities outside the classroom. The working method gives the pupils the opportunity to use their bodies and senses in learning activities in the real world in order to obtain personal and concrete experiences. Udeskole allows room for academic activities, communication, social interaction, experience, spontaneity, play, curiosity and fantasy. Udeskole is about activating all the school subjects in an integrated training where activities out-of-doors and indoors are closely linked together. The pupils learn in an authentic context: that is, they learn about nature in nature, about society in the society and about the local environment in the local environment." p.205 | 1. Denmark, NA 2. Denmark, NA 3. Denmark, Norway, & Sweden, 7-16 years 4. Denmark, NA 5. Multiple (review), childhood 6. Multiple, children | 1. Barfod 2016 2. Barfod 2018 3. Bentsen 2009 4. Bentsen 2010 5. Sharma-Brymer 2016 6. Waite 2016 |
|  | Outdoor classroom | | 1. "This means that beyond the playground and sports areas, educators use the school grounds as potential learning spaces for children. Urban, suburban, and rural school settings maximize the available landscape, resources, and space for of a range of developmental levels." pg 4   "The National Clearinghouse for Educational Facilities (NCEF) highlights the quality of openendedness as critical in designing space for children’s outdoor learning (Wagner, 2000)." pg 4  "outdoor classrooms include adjacent covered porches, amphitheaters, or picnic tables" pg 4   1. "we are defining the setting of the outdoor classroom as those spaces where students can experience familiar and unfamiliar phenomena beyond the normal confines of the classroom. We note that this is not an entirely satisfactory definition as much of the learning that we have seen taking place was prepared for, followed up and reinforced in the classroom; and not all of it actually took place out of doors." pg 19 2. "An outdoor classroom is a space, as its name suggests, that is outside. It can be interpreted and created in a way that suits each individual establishment and the children who visit. No two outdoor classrooms will ever be the same and on each visit the environment will have changed. It doesn’t have a roof, or walls. The floor may be muddy, grassy, covered in autumn leaves or new spring grass. The light is entirely from the sun and the natural, hands-on displays are interactive and ever-changing. The activities on offer can be self-chosen, adult-initiated or child-led. But quite simply it is outside." p.5 3. "An outdoor classroom is a space, as its name suggests, that is outside. It can be interpreted and created in a way that suits each individual establishment and the children who visit. No two outdoor classrooms will ever be the same and on each visit the environment will have changed. It doesn’t have a roof, or walls. The floor may be muddy, grassy, covered in autumn leaves or new spring grass. The light is entirely from the sun and the natural, hands-on displays are interactive and ever-changing. The activities on offer can be self-chosen, adult-initiated or child-led. But quite simply it is outside." p.5 4. "An outdoor classroom is a place for small- and large-group gatherings, readings, and discussions. This classroom also could be a place that allows activities that traditionally occur inside to move outside by providing chalkboards and traditional seating (but on stumps and logs!), or the classroom itself could be a garden, woods, wetlands, or creek." p.20 5. "Learning experiences conducted in the outdoors." p.28 6. (nature outdoor classroom) "The Learning with Nature Idea Book includes guiding principles for designing effective natural outdoor classrooms as learning environments (Arbor Day Foundation & Dimensions Educational Research Foundation, 2007). Natural outdoor classrooms designed with those principles in mind are environments that stimulate children’s creativity and enhance their learning opportunities. Research on these natural outdoor classrooms indicates that teachers who use them credit the enhanced aesthetic value and natural, open-ended materials available to children in these spaces as two key ingredients for supporting all learning opportunities, including creativity. A recent post-occupancy study describing the attributes of natural outdoor classrooms that teachers describe as effective states, “The most successful outdoor classrooms provided: maximum choice, many child-sized spaces, pathways and borders as play affordances, flexible spaces, and support for stakeholder engagement” (Dennis, Wells, & Bishop, 2014). An earlier study conducted at a Nature Explore Classroom in Minnesota also stated that the well-designed environment filled with natural materials supported creative play. The implementation of the guiding principles for designing outdoor classrooms supported the children’s freedom to choose what, how, and where to play with materials (Bohling, Saarela, & Miller, 2010)." p71 7. "Programme of education in the outdoors (rather than about the outdoors). Typically children spend a period of their schooling (ranging from a couple of hours a week to all their time) undertaking outdoor activities. Forest school is both a pedagogy and a physical entity, with the use often being interchanged." p.8 8. "The term ‘outdoor classroom’ was used by Rickinson et al. (2004) in their meta-analysis of research on outdoor learning, where they emphasize among other things the need for a deeper understanding and more reliable research evidence of the teachers’ conceptions of ‘the outdoor classroom’, as well as the curricular aims and pedagogical strategies that are seen as significant for effective teaching therein. I found this term to be appropriate for my study, as it reflects both the setting, i.e. the outdoors and the phenomenon I was exploring, i.e. the educational process." p.26 | 1. USA, NA 2. UK, NA 3. USA, 8-9 years 4. USA, NA 5. USA, school children 6. Ireland, 10-12 years 7. USA, 3-5 years 8. Multiple (Delphi) 9. UK, 6-12 years | 1. Burriss 2011 2. Dillon 2005 3. Eick 2012 4. Eick 2013 5. Keeler 2015 6. Kerr 2016 7. Kiewra 2016 8. Shanahan 2019 9. Stan 2009 |
|  | Outdoor kindergarten (friluftzvarnehager) | | 1. "Norway has a large population of nature- and outdoor kindergartens (friluftsbarnehager)" p.4 | 1. Norway, Australia, Children | 1. Sandseter 2012 |
|  | Outdoor preschool | | 1. "...is a learning environment that provides daily opportunities for children’s learning, exploration, construction, and problem invention and solving." p.257 2. "...is a learning environment that provides daily opportunities for children’s learning, exploration, construction, and problem invention and solving." p.257 | 1. USA, 4-5 years 2. USA, 4-5 years | 1. Zamani 2017 2. Zamani 2016 |
|  | Outdoor school | | 1. "the “outdoor school”, which is known in Germany as Draußenschule (Armbrüster et al., 2016), in Scandinavia as Udeskole/Uteskole (Bentsen, 2010; Jordet, 2007) and in English-speaking countries by various terms including “outdoor education”, “outdoor learning” and “education outside the classroom” (Beames, Higgins, & Nicol, 2012; Waite, 2011). The outdoor school approach is becoming increasingly widespread, especially in northern European schools, as a way of holding regular classes outside the usual school-based learning locations. Most commonly, children have a fixed day of the week on which they have lessons at places of natural or cultural interest—like a forest, park, museum or factory. Teaching in the outdoor school is curricular-based, includes (almost) all subjects in an interdisciplinary manner and is closely related to the classes held inside the school building. Children are able to use all their senses in the outside learning environment and experience objects in a first-hand and vivid way. Linguistic communication is of central importance for teacher– pupil interaction and the way lessons evolve both inside and outside the school. " p.2 2. "The outdoor school days include teacher-directed activities as well as children’s free play-based activities. During structured activities, where children often work in groups, a variety of teaching methods is used. Following the example of the Scandinavian Udeskole model (Bentsen, 2016; Nielsen et al., 2016), the outdoor school stands for regular opportunities of education outside the classroom, the use of indoor and outdoor locations in the local environment (forests, factories or the schoolyard) [...] Draußenschule (outdoor school) that ran in Germany [...] Udeskole (outdoor school)" p.215 | 1. Germany, 7-8 years 2. Germany, 7-8 years | 1. Sahrakhiz 2017 2. Sahrakhiz 2018 |
|  | Outdoor schooling | | 1. "Sommerset (2000) defines outdoor schooling as learning both about and in the community. The outdoors becomes a natural extension of the indoor classroom. Children's outdoor experiences build on and serve to extend traditional indoor learning. Lieberman and Hoody (1998) believe using the Environment as an Integrating Context (EIC) serves as "a framework for interdisciplinary, collaborative, student-centered, hands-on, and engaged learning"." pg 1 | 1. USA, NA | 1. Burriss 2011 |
| [Back to Table of Contents](#CONTENTTABLE) | | | | | |
|  | | | | | |
| **Curriculum/Pedagogy/Program** | | | | | |
|  | Adventure curriculum for physical education | | 1. "Attributes of adventure: risk taking, fun, challenge, and safety." "[ACPE fosters] respect for differences, problem solving, self-esteem, and compassion. These themes are integrated throughout the cooperative tasks. For example, some lessons require problem solving and serve as a way for students to learn how to “identify the different strengths of individuals in the group,” “[l]earn techniques to solve problems to accomplish group tasks,” and “[u]nderstand the importance of contributing ideas in the process of solving problems” pg. 223 | 1. Canada, Grade 7-8 | 1. Gibbons 2018 |
|  | Constructivist pedagogy | | 1. "Constructivism is, at its core, a "meaning making theory". As such, within constructivist educational theories, children create meaning via their interactions with others around them, including other children and adults, as well as the local environment. Seeing learners as co-constructors and not as mere receivers of knowledge is central to the FS approach." pg 42 2. "...the idea that new knowledge depends upon, and builds upon, previous knowledge...The experiential nature of outdoor instruction does provide a powerful context in which to become involved with knowledge construction." pg 36 | 1. Denmark, NA 2. NA, Middle school students | 1. Barrable 2019 2. Broda 2002 |
|  | Cross curricular activities | | 1. "The emphasis of cross-curricular activities is specifically upon combining a physical activity component with other academic material. One example is incorporating physical challenges with studying for a spelling test or practicing math skills through interactive play. This curriculum also spotlights teamwork skills by having students work together in team settings to solve problems" pg.96 | 1. USA, 4^th^ Grade | 1. Greene 2013 |
|  | Curriculum based residential outdoor learning course | | 1. Example: "5-6 days" pg 3   "The research weeks combined social learning, personal development, and ecological knowledge on a regional level in an educational concept in order to achieve sustainable learning effects in “global learning.” During the research weeks, the students are split into groups of three to four and develop their knowledge in plant phenology, meteorology and mircoclimatology, glaciology, and pedology—subjects that are part of the official curriculum. Each micro-group is accompanied by either one of the two accompanying teachers, a pre-service teacher student, a staff-member of the science center, or one of the evaluative researchers/authors. On the first 2 days of the course, each group prepares for a research expedition in the lab, i.e., getting to know their specific indicator plant, learning about weather parameters and local weather history, or the geological conditions in the area. Students then complete a 2-day expedition that includes student-led organization of a research protocol and collection of data along a transection of approximately 1,000 m in altitude. Each student group is assigned one facilitator—an accompanying teacher, a student, or scientific staff from the Student Research Center—in order to empower the students to carry out the complex fieldwork and document their findings. A detailed graphical description of the program can be found in Supplementary Image 1." pg 3 | 1. Germany, 12-14 years | 1. Dettweilder 2017 |
|  | Eco-pedagogy | | 1. ''...is concerned with generating change through critical pedagogic processes, drawing upon the Freirean concept of conscientization. Conscientization regards learners as active agents who become “literate” through developing experiential awareness of practical issues confronting them existentially. Being in possession of a knowledge about themselves and wider social and cultural factors governing their situation is thought to enable human, environmental, and social relationship transformations.'' p.119 | 1. UK, NA | 1. Dunkley 2018 |
|  | Eco-psychology | | 1. "A scientific discipline that examines the effects of natural settings on psycho-spiritual growth and wellness. This   science explores causal conditions between environments and personal wellness." pg.293 | 1. USA, 11-14 years | 1. Flett 2010 |
|  | Ecotourism | | 1. "In 1965, Hetzer claimed that ecotourism must be considered from perspectives of culture, education, and tourism, and the author promoted the possibilities of ecological tourism (Hetzer, 1965). In 1980, the International Union for the Conservation of Nature, the UN Environment Programme, and the World Wildlife Fund developed the World Conservation Strategy, proposing direct links between environmental conservation and economic development to achieve the goal of “conservation that promotes development and development that strengthens conservation” (IUCN, 1991)." p.3265 | 1. Taiwan, students | 1. Liu 2017 |
|  | Greening the curriculum | | 1. "Greening the curriculum is to move beyond simply studying the environment to taking purposeful action with youth applying what they have learned to their environment while creating connections among subjects." p.10 | 1. USA, school students | 1. Kaye 2012 |
|  | Leisure centred curriculum | | 1. "...non-graded, multi-aged classes with a low pupil to teacher ratio. The curriculum is interdisciplinary and includes a strong experiential environmental education and community service component." pg 12 | 1. USA, NA | 1. Bentley 2010 |
|  | Nature as a teacher | | 1. "By moving freely in outdoor spaces, learning by looking into and with nature rather than at it, developing self-confidence in natural landscapes, engaging with the sensuality of nature, engaging in unstructured and spontaneous play, and enjoying the sensory awareness of being engaged outside will all provide a rich learning situation". pg.118 | 1. Canada, 3-6 years | 1. Elliot 2018 |
|  | Nature-based health interventions | | 1. "NBIs are programmes, activities or strategies that aim to engage people in nature–based experiences with the specific goal of achieving improved health and wellbeing." p.2 | 1. Multiple (Delphi), NA | 1. Shanahan 2019 |
|  | Nature-based pedagogies | | 1. "nature-based pedagogies are distinctive in that the environments where practice is situated, afford opportunities for pedagogues to encourage children to foreground meaning making through engagement with nature’s sensory and kinesthetic cues." p.468 | 1. Denmark, Finland & Scotland, Kindergarten | 1. Nugent 2018 |
|  | Nature study | | 1. "virtually everything found in the natural world. Indeed many of the elements and processes of nature made up the body of the studied curriculum, including plants, animals, rocks, weather, and astronomy; and all investigated through first-hand experiences in the outdoors." p.791 | 1. USA, 8-9 years | 1. Eick 2012 |
|  | Inclusive outdoor education and interpretive programs | | 1. "Inclusive outdoor education and interpretive programs and facilities would offer opportunities for everyone to become aware of and appreciate our natural and cultural resources. They would also provide opportunities for persons with disabilties to interact with persons who do not have a disability, opportunities to become acquainted with community recreation and educational resources, and the opportunity for resouce management agencies to serve a large area." pp.11 | 1. NA, 8-15 years | 1. Taproot 2001 |
|  | Outdoor adventure/experiential education program | | 1. "Many outdoor adventure programs incorporate the concept of experiential education, which suggests that people learn best by direct and purposeful contact with their learning experiences." pg. 41 2. "Evidently, the unique and effective component of outdoor experiential programmes such as wilderness challenge programmes (Wilson & Lipsey, 2000), wilderness therapy (Harper, Russell, Cooley, & Cupples, 2007), outdoor behavioural healthcare treatment (Russell, 2003) and adventure programming (Hans, 2000) is the emphasis on physically challenging activities in the outdoor environment, such as backpacking or rock climbing, combined with treatment grounded in the field of experiential learning (Gillis & Gass, 1993; Wilson & Lipsey, 2000)." p.140 3. (also outward bound) “The history of outdoor adventure programs dates back to 1941, when Kurt Hahn founded Outward Bound (which can be called the first version of adventure programming) in Wales (Priest & Gass, 2005). Hahn proposed Outward Bound as a way to combat perceived social ills resulting from industrialization, such as the decline of fitness, initiative, spirit of enterprise, and self-discipline (Freeman, 2011; Hopkins & Putnam, 1997; Priest & Gass, 2005). The first Outward Bound courses focused on seamanship and intended to impart the ability in young male seamen to survive harsh conditions at sea in the merchant marine during war time by teaching tenacity, perseverance, leadership and confidence (Kelly & Baer, 1969; Miner & Boldt, 2002). In subsequent years, however, the range of outdoor activities also included, for instance, backpacking, mountaineering and canoeing, and the program's goals were refined, with greater emphasis put on personal growth and self-discovery (Freeman, 2011)" p.105 4. "Since their very beginnings in the 1940s, when Kurt Hahn founded Outward Bound (which can be called the first version of adventure programming) in Wales, outdoor programs were designed to counter the assumed negative influences of modern life (Freeman, 2011; Hopkins & Putnam, 1997; Priest & Gass, 2005). Hahn proposed Outward Bound as a way to combat a perceived decline of fitness, leadership, spirit of enterprise and self-discipline, which he regarded as negative by-products of industrialization and urbanisation (Freeman, 2011)." p.57 5. (outdoor adventure programming) "OA relies upon the process of experiential learning within a dynamic, natural setting to generate adaptive skill sets for young people. Experiential learning represents a progressively staged mechanism whereby participants learn through direct immersion and reflection of experiences [33,34]. An array of unfamiliar experiences is purported to compel individuals to engage with risk and uncertainty [35]." p.2 | 1. USA, NA 2. Netherland, 17-22 years 3. Germany, 14 years & University students 4. Germany, 13-20 years 5. UK, mean 11 years | 1. Garst 2001 2. Schreuder 2014 3. Mutz 2016 4. Mutz 2019 5. Slee 2019 |
|  | Outdoor education schemes | | 1. "Schemes designed to introduce children/adults to nature with the purpose of altering their knowledge about, attitudes toward and contact with nature" p.9 | 1. Multiple (Delphi), NA | 1. Shanahan 2019 |
|  | Outdoor learning program | | 1. "a range of initiatives are offered to both primary and secondary schools in and out of school time (Rickinson et al., 2004), and these represent a spectrum of learning programmes from those that are tailored towards educational topics and the core curriculum, and broader programmes using the natural environment as a context for experiential purposes, engagement and socioemotional wellbeing. Outdoor learning programmes vary in delivery time, with most programmes lasting from a few hours to a full day; others such as forest or residential programmes last multiple days. " p.574 | 1. UK, 8-11 years | 1. Quibell 2017 |
|  | Pedagogy of listening | | 1. "It is this openness, this “pedagogy of listening,” that makes space and time for wonder on a busy street en route to our destination. This is a pedagogy that engages and embraces the possibilities inherent in interruptions. It is not always easy to stop in the middle of a trip to wonder at something. We are in a hurry. We have destinations to reach, and small feet to encourage along the way; thus, to stop midjourney to process all of the minutiae that children notice and comment on seems impractical. I fully understand the “we need to get going, you took forever to get ready, we’re already late and we really don’t have time for this” feeling, but I have learned to calm these voices of impatience with a few questions. Why are we in a hurry? Why do we need to get there? Is where we’re headed any more important to the children than what they are observing right now? These questions inevitably stop me in my tracks, commanding me to stop, listen, and honor the children’s observations." pg 148   "... the child’s perspective on the world takes precedence, thereby shaping the steps that we take. On these walks, the child has the opportunity to redefine the world—assigning stories, games, and perspectives that we would never know, had we kept up a hurried, “adult” pace." pg 154 | 1. USA, 3 years | 1. Bentley 2012 |
|  | Place responsive pedagogy | | 1. ''...considering how educators make explicit efforts to collaborate in assembling people, places and purposeful activities together, to produce viable and valuable environmental educational experiences.'' p.53 | 1. USA, NA | 1. Dolan 2016 |
|  | Play pedagogy | | 1. " Play-pedagogy refers to the systematic practice of facilitating children’s learning through play, with a focus on imaginative and creative endeavour. It encompasses freedom, flexibility and structure with a mix of adult- and child-initiated activity." pg 22   "a pedagogy which counters traditional classroom teaching, particularly for children beyond EY" pg 34 | 1. UK, 4-9 years | 1. Coates 2019 |
|  | Play work | | 1. "Playwork is a practical approach to play theory with origins in the United Kingdom..." Playwork principles include: "Play is a process that is freely chosen, personally directed and intrinsically motivated. That is, children and young people determine and control the content and intent of their play, by following their own instincts, ideas and interests, in their own way for their own reasons." 2. (playworker) " There are also trained "play workers" on site, supervising. But they supervise lightly, Donald says. "You kill their creativity by hovering too close. There is a fine line between what's being creative and what's being dangerous." pg 34 3. (playworker) "Specific to an adventure playground is the fact that these venues are only physically accessible for children to play in when a playworker (or volunteer) is on site. In comparison to traditional playground supervision, adventure playgrounds offer regular professional scaffolding for children by trained playworkers. Just as a scaffold on a construction site would allow for construction to safely take its course, play scaffolding enables children to take control of their own play experiences. The role of the playworker is that of an unobtrusive guide, a professional who “reinvigorates the process of child development through play” (Brown, 2003, p. 51). A playworker is a play mediator, or as Sturrock (2003) writes, a play shaman who’s principal role is to attend to the child’s developmental imbalance that is perceived to be caused predominantly by a shortage of play opportunities in the child’s life. In most cases, a playworker is a trained professional educated within the philosophy of playwork. The focus in playwork is on the individual child, and as Brown (2003) points out, there are two interrelated characteristics to playwork. In its simplest form, these characteristics include the removal of play barriers and the enrichment of the child’s play environment. By analyzing the child’s environment, the playworker is able to address and remove barriers of physical and/or emotional nature that prevent play from happening. At the same time, attention is focused on the stimulation and enrichment of the child’s developmental processes, using play as a core element in the healing process." p.271 | 1. Canada, NA 2. USA, NA 3. Multiple (review), children | 1. Alden 2019 2. Clendaniel 2009 3. Staempfli 2009 |
|  | School ground greening | | 1. "While there are important differences between each term, and while each term is itself somewhat contested, for the purpose of this paper ‘school ground greening’ will be used to describe collaborative efforts to improve school grounds [...] with green areas" p.178 | 1. Australia, 5-12 years | 1. Lucas 2010 |
|  | Scouting | | 1. “Exemplify science ideas encountered within the school curriculum” pp. 160 | 1. Mean 12.3-13 years | 1. Hintz 2012 |
|  | Standard-based curriculum | | 1. "Standards-based curriculum subtly communicates to the public the curriculum fallacy of universalism. This is the view that some particular content can be identified that is of fundamental and universal significance regardless of context or of the characteristics of the student." pg 6 | 1. USA, NA | 1. Bentley 2010 |
|  | Voyager wilderness program | | 1. "Some say Voyageur Wilderness Programme (VWP) virtually invented the concept of the “ecological education through wilderness experience” when, in 1960, we took our fi rst student group and eco-adventurers into Quetico Provincial Park. We’re proud to be pioneers in the field of wilderness-based experiential learning and eco-travel. We continue to dedicate ourselves to being one of Canada’s leading wilderness education and eco-adventure outfitting programs. [...] VWP sees wilderness education as providing a catalyst mechanism for change. Wilderness based experiential learning involves primal experiences, investigation, improvisation, teamwork and problem solving. Our approach in wilderness eco-travel emphasizes self-reliance, reflection, self-knowledge and cooperation. " p.12-13 | 1. Canada, students | 1. Savole 2008 |
|  | Wilderness based programs | | 1. "Wilderness-based programs for youth have a rich history in the United States. Outdoor adventure programs like Outward Bound, which has been in existence in the United States since 1963, utilize a wilderness environment, challenge and adventure activities, and cooperative peer and adult relationships to promote leadership and character development (Goldenberg, McAvoy, & Klenosky, 2005; Kellert & Derr, 1998)" p.339 2. "Programmes designed to challenge participants in natural environments." p.7 | 1. USA, 13-18 years old 2. Multiple (Delphi), NA | 1. Norton 2014 2. Shanahan 2019 |
| [Back to Table of Contents](#CONTENTTABLE) | | | | | |
|  | | | | | |
| **Therapy** | | | | | |
|  | Adventure therapy | | 1. Similar term as OAI 2. " Adventure therapy is used as an intervention within the field of therapeutic recreation where outdoor experiential activities can be used to accomplish treatment-related goals. Adventure therapy can occur in wilderness settings or within a variety of facility-based settings (e.g., inclusive, psychiatric, physical rehabilitation, educational, and correctional; Datillo, 2000). Outdoor experiential/adventure activities such as ropes course initiatives and outdoor activities (e.g., hiking,backpacking, rock climbing, camping, and canoe/raft trips) are often included in adventure therapy programs." pg. 299 3. (Therapeutic adventure, outdoor Programs) "Williams (2004) makes a distinction, adopted in the present article, between adventure therapy and TA. Therapy programs are based on identified or diagnosed disorders, by properly trained and credentialed individuals, with the systematic application of assessments and treatments. Such an approach is inherent in the mental health field. However, therapeutic programs are those that may promote positive change, but in a less formal way, or by non-professionals, or with a less systematic approach, often without the goal of alleviating the symptoms of a disorder. These outdoor programs will be placed on the continuum of mental health care." pg 54 4. "Adventure Therapy (AT) is a mode of group counseling that intentionally utilizes adventure activities to facilitate social-emotional and personal growth as well as behavior change in participants. Adventure therapy is a strength-based mode of counseling that allows natural consequences to facilitate personal insights. Counselors facilitating AT use real and perceived risks inherent in kinesthetic and metaphoric activities, to create disequilibrium in participants that requires new forms of coping. Adventure therapy can be tailored to fit outdoor settings such as wilderness, parks, and school play areas as well as indoor settings such as school buildings, gyms, and counseling offices." pg 25 5. ''…prescriptive use of adventure experiences provided by mental health professionals, often conducted in natural settings that kinesthetically engage clients on cognitive, affective and behavioral levels.'' p.33 6. "as therapy taking place outside as opposed to inside or, more specifically, in a wilderness environment as opposed to an institutional one. There's also four possible adventure-based therapeutic approaches with CAP patients: behavioural therapy; psychodynamic interventions; systemic interventions; and occupational therapy." p53-54 7. "Adventure therapy is action-oriented, conducted in an unfamiliar environment, designed to capitalize on the positive use of stress, rich in assessment possibilities, conducted in small-group and a supportive atmosphere, is solution-oriented, and changes the role of the therapist." pg. 35 8. "Adventure-based therapy (ABT) has been described by Gillis, Gass, and Russell (2008) as focusing on group development activities through problem-solving initiatives alone, or in combination with low- and high-challenge (ropes) course experiences." pg.22 9. “treatment that takes place outside as opposed to inside or more accurately in a wilderness as opposed to an institutional setting” pg. 228 | 1. Austraila, 18-50 program leaders/managers 2. USA, 13-18 years 3. USA & UK, NA 4. USA, 9^th^ grade 5. Australia, NA 6. Germany, 11-13 years 7. USA, NA 8. USA, 12-16 years 9. USA, 14-17 years | 1. Bowen 2016 2. Autry 2001 3. Berman 2013 4. Christian 2019 5. Dobud 2016 6. Eckstein 2015 7. Gass 1998 8. Gillis 2010 9. Gillis 2018 |
|  | Bush adventure therapy | | 1. Similar term as OAI | 1. Austraila, 18-50 program leaders/managers | 1. Bowen 2016 |
|  | Outdoor adventure interventions (OAI) | | 1. "OAIs for youth in Australia are diverse, but typically incorporate several days in the outdoors, during which participants engage in adventure activities and group activities requiring teamwork, with intentional involvement of program leaders. The main outcomes, as perceived by staff, were recreation and personal and social development." pg 26   "The term outdoor adventure intervention is used in this article to refer to outdoor adventure programs that combine small groups, contact with nature, participation in adventure activities, and therapeutic processes to create opportunities for change in participants and to support an individual (or family group) to move toward greater health and well-being." pg 27  "OAIs often target young people at risk, adjudicated youth, mental illness, substance use problems, and people with disabilities. The aim of these programs is generally to provide experiences and opportunities to bring about lasting educational, therapeutic, and rehabilitative change." pg 27 | 1. Austraila, 18-50 program leaders/managers | 1. Bowen 2016 |
|  | Outdoor RX | | 1. "… examples of program activities include nature walks, soccer games, and unstructured playtime at the beach." pp.520 | 1. USA, 2-13 years | 1. James 2017 |
|  | Residential treatment | | 1. "Residential treatment is a generic term for different types of residential therapeutic treatment, which includes wilderness therapy." pg 247 | 1. USA, 14-17 years | 1. Bettmann 2015 |
|  | Social and therapeutic horticulture | | 1. "Sempik uses the term “Social and Therapeutic Horticulture” (STH) to describe greenspace-based interventions with vulnerable groups such as those with mental health needs [20–24]. A core feature of Sempik and colleagues is a focus on improving the wellbeing of participants engaged in STH, rather than productive gardening, in line with Stress Reduction and Attention Restoration theories. Others, also identify the positive impacts of greenspace and horticulture-based interventions on the mental wellbeing of children.1" pg 2 | 1. UK, 10-14 years | 1. Chiumento 2018 |
|  | Therapeutic camping | | 1. "Traditional therapy in an office attempts to solve narrowly defined problems from a remote setting. Therapy at camp did the opposite, approaching a broad range of total living experiences. Problems of every shape, form, and description were met as they occurred, and through the process, the boy gradually worked all the knocks out of his engine until his whole engine ran smoothly. In other words, therapy was holistic." p.10 | 1. USA, NA | 1. Loughmiller 2007 |
|  | Wilderness therapy/treatment | | 1. Similar term as OAI 2. (Wilderness treatment) "Russell (2007) defines wilderness therapy as “[A]n integrative treatment model that incorporates well-established therapeutic modalities (e.g., cognitive behavioral and humanistic) within the context of wilderness group living and backcountry travel” (p. 53). Wilderness therapy programs typically include four primary factors to facilitate therapeutic change in clients: (a) utilizing the wilderness as an avenue for healing, (b) promotion of self-efficacy through task accomplishment, (c) restructuring of the therapeutic relationship, and (d) the development of a therapeutic social group. These factors are present in other models of treatment but are considered basic tenets in wilderness therapy settings. The length of treatment is shorter than other residential programs, typically lasting from 3 to 8 weeks." pg 248 3. "Unlike traditional residential treatment programs, wilderness therapy programs provide therapeutic care entirely within a wilderness setting. However, regardless of the setting, the nature of residential treatment involves removing adolescents from their caregivers and other social networks; thus, these primary attachment relationships and associated behavioral patterns factor importantly into the treatment process. Using small treatment groups, high staff-client ratios, and focused therapeutic approaches, wilderness therapy programs can challenge adolescents’ relational patterns and behaviors by allowing them to connect with peers in healthier ways. In wilderness therapy programs, clients must face regular losses, separations, and reunions with program staff and peers, as students graduate from programs and staff finish their weeklong shifts. These losses, separations, and reunions activate the attachment system. Thus, wilderness therapy, by placing adolescents in a new environment with few peers and staff, provides an ideal setting in which to investigate attachment processes and relationships. Relational dynamics can be more closely observed, and clients’ attachment patterns may be more visible and consequently better able to be addressed." pg 185-86 4. "...the young people were engaged in a range of activities, such as the Wilderness Challenge that involved four activities: rock-climbing, archery, problem-solving and flag painting. They also undertook night walks through the forest and played various outdoor team games." pg 131   "...rock climbing, a trapeze jump, team building activities, and forest walks." pg 131   1. (Also outdoor behavioural health care) "What was once referred to as a "boot camp" intervention has taken on an increasingly sophiscated approach as it has developed as a treatment modality for adolescents and young adults. 2. "Wilderness therapy (WT) emerged in the 1920s as a rehabilitation program for adolescents at risk. WT programs involve expedition-based interventions in outdoor wilderness settings that aim to change maladaptive behaviors via experiential learning. A focus is placed on group aspects including promoting communication with peers, interpersonal abilities, reliance on peers and staff (Clark et al. 2004; Russell and Farnum 2004), supporting group initiatives and decision-making processes, and learning how one is perceived by others (Corey and Corey 2000; Newes and Bandoroff 2004)." p.182 3. "The naturalness and solitude that wilderness offers restore and renew our spirit, offering evidence that wilderness is in itself therapeutic (Russell & Farnum, 2004). Kaplan and Kaplan (1989) propose that three factors are operating in wilderness therapy: The Wilderness: elements of the natural world that create change. Physical Self: activities or processes that facilitate learning. Social Self: social interaction." p.242 4. "Several terms have been coined to describe therapeutic wilderness programs, including adventure therapy (Williams, 2000), Outward Bound (Bacon, 1983), wilderness challenge programs (Wilson & Lipsey, 2000), outdoor behavioral healthcare (Russell, 2005), and wilderness therapy (Russell, 2001). Authors have attempted to delineate key elements within each of these and there are syntheses of the literature that offer useful comparisons, highlighting areas of congruence and variation (cf. Russell, 2001). Among most, there is an emphasis on the therapeutic aspects of clinical assessment, intervention, and monitoring. The presence of the wilderness is implicitly embedded in many of these conceptions (Beringer, 2004). Williams (2000) uses the term adventure therapy, which he claims refers to “the use of outdoor education and recreation activities as a form of therapy . . . [that] takes place in a wilderness as opposed to an institutional setting” (p. 47). In their definition, Berman and Davis-Berman (1991) state that the essential components [of wilderness therapy] involve taking troubled youth into uninhabited areas for more than a few days. The group sleeps out overnight, and is self-sufficient. The primary goal of the program is to create change in the previously identified problem areas for each participant (p. 375). According to Russell (2001), there are several guiding elements that must be present in a description of wilderness therapy that adequately captures the nuances of practice. Specifically, wilderness therapy should have a distinct and well-defined therapeutic focus aimed at non-intrusively inducing personal growth through encouraging self-reflection and the development of personal awareness. Second, therapeutic goals should be monitored and facilitated by trained professionals such as therapists, clinical social workers, or psychologists with the purpose of creating change in targeted areas. These sentiments are also expressed by Berman and Davis-Berman (1991) who draw attention to the importance of a well-articulated treatment plan with clear and attainable goals for each student. Russell also comments on the common outcomes that should be expected from participation in a wilderness program such as improvements in self-esteem, interpersonal skills, and self-management." p.219 5. "Structured nature–based activities and programmes in ‘wilder’ environments for ‘at risk’ groups or those recuperating or in recovery" p.7 6. "Wilderness Adventure Therapy (WAT) relies on teaching through experience in natural contexts and, consequently, can also be considered a contextualized intervention. It stems from the more widely known practice of Outdoor Experiential Education (OEE) which is in fact an amalgamation of environmental education and adventure education." p.1058 7. "WT is a form of experiential group therapy, defined by Weston and Tinsley (1999) as: A systematic experiential group intervention that occurs in a natural setting and employs therapeutic techniques and processes within the context of activities and experiences that contain elements of real or perceived risk (i.e. physical, social and emotional) to facilitate the improvements in the psychological and behavioural functioning of the participant. (p. 31) Defining WT is complicated by the many different terms used to describe various forms of outdoor experiential treatments. Common examples include adventure therapy, adventure counselling, adventure-based therapy and WT (Berman & Davis-Berman, 1991; Gillis & Gass, 2004; Rayment, 1998). Although there is inconsistency and considerable overlap in the definitions of these terms, one key difference is that adventure therapy does not necessarily involve a wilderness setting (Gillis & Gass, 2004). However, even this distinction is not always maintained (e.g. Williams, 2000). WT should also be differentiated from wilderness experience (Davis-Berman & Berman, 1993). Gass (1993) suggests that along with the systematic focus on specific treatment goals, reflective techniques that promote the generalization of experiences from the wilderness setting to everyday life are critical in this distinction." p.162 | 1. Austraila, 18-50 program leaders/managers 2. USA, 14-17 years 3. USA, 14-17 years 4. UK, 9-15 years 5. USA, Mean 15.6 years 6. Israel, 14-16 years 7. UK, 4-5 years 8. Multiple (review), NA 9. Multiple (Delphi), NA 10. Multiple (review), childhood 11. New Zealand, 13-18 years | 1. Bowen 2016 2. Bettmann 2015 3. Bettmann 2011 4. Brewer 2011 5. Hoag 2014 6. Margalit 2014 7. McArdle 2013 8. Rutko 2013 9. Shanahan 2019 10. Shanahan 2009 11. Somervell 2009 |
|  | | | | | |
| **Activity/Adventure/Experience/Recreation** | | | | | |
| *Activities* | | | | | |
|  | Active travel | | 1. "Active travel captured human-powered transportation such as walking or bicycling to all destinations." pg 178 2. "Active travel refers to human-powered transportation such as walking or bicycling." pg 2 | 1. Canada, 10-13 years 2. Canada, 10-13 years | 1. Borghese 2019 2. Borghese 2018 |
|  | Adventurous activities | | 1. "Adventurous activities include: Climbing: for example rock climbing, abseiling, ice climbing, gorge walking, ghyll scrambling, sea level traversing, high- and low-level ropes courses; Watersports: for example canoeing, kayaking, dragon boating, wave skiing, white-water rafting, improvised rafting, sailing, sailboarding, windsurfing; Trekking: for example hill-walking, mountaineering, fell-running, orienteering, pony trekking, off-road cycling, off-piste skiing; Caving: for example caving, pot-holing, mine exploration; Challenges and Skills: for example archery, quad bikes, assault courses, mountain boarding, initiative exercises." | 1. UK, NA | 1. Council for learning outside the classroom 2019 |
|  | Child-initiated activities | | 1. “free, spontaneous play within a structured learning environment with the adult acting as facilitator (DCELLS 2008a; DCELLS 2008b).In summary, this activity is both child-initiated and child-led.” P.214 | 1. UK, 4-7 years | 1. Maynard 2013 |
|  | Dangerous outdoor leisure activities | | 1. "potentially dangerous outdoor leisure activities relate to fireworks, roller blading, skateboarding, caving, climbing and water sports, and locations include playgrounds and farms.5-7" p113 | 1. UK, children under 15 years | 1. Pearson 2012 |
|  | Dog walking | | 1. "Dog walking status was determined by children reporting whether they had taken their dog for a walk in the last week (yes/no)" pg 260 | 1. Australia, 10-12 years | 1. Christian 2014 |
|  | Fitness break | | 1. "Fitness breaks have also been implemented within the school setting to encoruage children's active play" pp.58 | 1. NA, NA | 1. Hyndman 2015 |
|  | Foraging | | 1. "Foraging is a quest—a hunt for wild plants, fish, and other edible resources— characterized by searching, gathering, occasionally cooking, and usually consuming that which is found." p.78 | 1. Scotland, Kindergarten | 1. Nugent 2015 |
|  | Friluftsliv | | 1. "In Denmark, Norway and Sweden outdoor recreation and outdoor education is often referred to as friluftsliv (literally meaning free/open-air life)." pg 30 2. (Friluftsliv education) " unifying itself with nature and natural things, as a possible outcome of their teaching and as a premise for sustainable development." pg.243   "a continuation of earlier (hunter–gatherer) ways of living and simultaneously... a concept describing more-or-less non-competitive contemporary lifestyles in nature." pg. 233   1. (traditional outdoor recreational activities) "Friluftsliv has been defined in different ways (Odden, 2008; Tordsson, 2002). Norwegian authorities have defined friluftsliv as “staying outdoors and being physically active during leisure hours to have a change of environment and experience nature” (Klima og miljødepartementet, 2016, p. 5), which is a definition that has remained virtually unchanged in Norway from 1987 to the present. Outdoor recreation, outdoor life, or adventure are other concepts that are closely related to friluftsliv (Odden, 2008; Pedersen, 2010; Tordsson, 2002). However, the idea of friluftsliv comprises more than physical movement; it also indicates being inspired by and experiencing nature." p.22 2. "outdoor activity and leisure time in Norway" p.3 3. " a particular type of immersive nature-experience, namely the Scandinavian tradition and practice of so-called friluftsliv, which includes concepts of, e.g. ‘outdoor life’, ‘outdoor recreation and education’ or ‘adventure recreation and education’, but with an emphasis on the experience of closeness to nature during the activity (Bentsen et al., 2009a; Gelter, 2000).". p. 2 4. "‘Friluftsliv’ is seen not just as outdoor recreation, but also concerns people’s close relationship with nature, ‘feeling at home in nature’ and taking care of nature." p. 2011 5. "In the Norwegian society the concept friluftsliv (similar to the concept “outdoor life”, but holds a stronger notion of values and lifestyle) is an important part of the national cultural heritage." p.3 | 1. Denmark, Norway, & Sweden, 7-16 years 2. Norway, NA 3. Norway, 16-19 years 4. Norway, 1-5 years 5. Multiple (review), 0-18 years 6. South Korea & UK (England), 3-4 years 7. Norway & Australia, children | 1. Bentsen 2009 2. Gurholt 2014 3. Lagestad 2019 4. Lysklett 2017 5. Mygind 2019 6. Nah 2015 7. Sandseter 2012 |
|  | Games | | 1. "Pellegrini (1995) says that play is an activity done for its own sake which is flexible and fun. Play can be contrasted with exploration which may lead to play, work which has a goal, or games which are organised with the aim of winning." pg 37 | 1. New Zealand, NA | 1. Couper 2011 |
|  | Traditional games | | 1. "Oranges and Lemons, huts, marbles, swings and sandpits? Traditional games have all but disappeared in one generation. We are losing games that, according to Blatchford (1998), belong to children and have passed from child to child." pg 38 "Repetitive chants and songs make them easy to learn including circle games like The Farmer in the Dell, The Hokey Tokey, The Big Ship Sails on the Alley Alley Oh." pg 38   "Games like knucklebones, marbles, pick-up sticks, rakau (Maori stick games), string games, whip ‘n’ tops, and yo yos increase" pg 38 | 1. New Zealand, NA | 1. Couper 2011 |
|  | Green exercise/PA | | 1. "...performing PA whilst exposed to nature…" 2. "...activity in the presence of nature…" pg 2055 3. ''…is characterised by physical activity with direct exposure to nature. Such activities may include: hiking, walking, rambling, swimming, pony trekking, rock climbing, cycling, sailing and fishing.'' p.358 4. (Green PA) "We were able to define two different measures of use of green space for physical activity, or ‘green physical activity’ as we then labeled it. The first measure defined green physical activity as green spaces “used once a week or more”, the second defined green physical activity as green spaces “used three times a week or more”." p.3 5. ''…is characterised by physical activity with direct exposure to nature. Such activities may include: hiking, walking, rambling, swimming, pony trekking, rock climbing, cycling, sailing and fishing.'' p.358 | 1. UK, 8-9 years 2. USA, 10-13 years 3. UK, mean 10.1 years 4. UK, 16+ years 5. UK, 8-9 years | 1. Barton 2015 2. Beyer 2015 3. Duncan 2014 4. Ord 2013 5. Wood 2014 |
|  | Hikes | | 1. Example: "...hikes away from the kindergarten, outside the fences..." pg 309 | 1. Norway, 3.5 years | 1. Bjorgen 2015 |
|  | a) Drama hike | | 1. "Drama is a real walk into the woods: smelling, feeling and seeing Nature. It is also an imaginative search for ancient civilizations, in this case the “Hully Gullies.” Since we know little about the Hully Gullies, the purpose of the trip is to discover what these people might have looked like, their social habits, religion, sports and so on. We will use Nature and all of her formations as clues in our search." | 1. NA, NA | 1. Jordison 2001 |
|  | Integrated camping | | 1. "Integrated camping (i.e., camping involving participants with and without disabilities)" p.1 | 1. USA, 9-13 years | 1. Rynders 1990 |
|  | Mobility play activities | | 1. "Mobility activities (i.e., play activities that involve movement from one location to another) and interactions with toys/objects" pg. 41 | 1. USA, 8 months-6 years | 1. Guerette 2013 |
|  | Nature activities | | 1. “…nature activities, such as caring for goldfish and doves, planting seeds and bulbs, and tending flowers…” pg 1034 2. (nature-based activites) "a specific nature-based activity (for example, listen to frogs, hike, play in the leaves, catch frogs and turtles, fish, hop on rocks, look at nature, walk through the grass, collect things, pick flowers, skip rocks, take pictures, walk on the logs)" pg 36 | 1. Multiple, NA 2. USA, 3-6 years | 1. Brown 2017 2. Desrochers 2001 |
|  | Nature exploration | | 1. "...often highly physical, combining bodily interaction and sensory attentiveness..." pg 16   Examples: "... scour the ground for bones, pine cones, sparkly sandstone, feathers, or wildflowers’" pg 16 | 1. Sweden (adults) & Norway (1-6 years) | 1. Beery 2018 |
|  | Nature journaling and Phenology | | 1. "...students make observations, and infer changes over time in the park; they write and draw what they learn..." pg 2059 | 1. USA, 10-13 years | 1. Beyer 2015 |
|  | Non-competitive outdoor physical activity | | 1. "Non-competitive activities, including unstructured play and other activities that do not require participants to compete against one another." pg 510; Examples: "backpacking or canoeing" pg 513 | 1. USA, 10-14 years | 1. Christiana 2014 |
|  | Knowledge integration activities | | 1. "In order to synthesize the information obtained through the cognitive preparation activities and field trips, the students engaged in several types of knowledge-integration activities such as drawing and summarizing the outdoor experiences." pg. 543 | 1. Israel, 4^th^ grade | 1. Assaraf 2010 |
|  | Open air education activities | | 1. “Gair (1997) states that any open-air educational activity in rural and urban areas can be regarded as outdoor education.” Pg 100 | 1. Sweden, Finland, Latvia, Germany , Greece, 0-25 years | 1. Brodin 2009 |
|  | Outdoor and adventurous activities | | 1. ''…a means of approaching objectives through guided, direct experience in the environment, using its resources as learning materials.'' p.9 | 1. UK, 9-10 years | 1. Dismore 2005 |
|  | Outdoor expeditions | | 1. Example: "all-day weekend outdoor expeditions to nature sites including Epping Forest and Oxshott Woods" pg 1034 | 1. Multiple, NA | 1. Brown 2017 |
|  | Outdoor (physical) activities | | 1. (Outdoor activities) "...any kinds of activities conducted outside of the class.." 2. "...always involve some risk, but the risks are greatly outweighed by the health and development benefits." 3. (Outdoor physical activities) Examples: "Walking, running and boating…" pg 140 4. "Physical activities such as running, jumping, kicking or swinging" pg 3 5. "...defined as activities usually done outdoors... and usually in one’s yard or street, and included bicycling, hiking, roller skating, running, skateboarding, street hockey, swimming, walking, and yard work." pg.877 6. "nonsporting activities they usually do outdoors." pg.169 7. "…time spent outdoors." pg. 1143 8. "... with many performing daily exercises in settings such as parks, public squares, and streets, in the forms of walking, jogging, social dancing, Tai chi, and shuttlecock kicking." pp. 521 (15,16) 9. "Activities that were performed outside a building, for example park visits, walking around the neighbourhood and outdoor sports were classified as outdoor activities." p.364 10. "Child-reported child’s outdoor PA was measured by the following question: “Last week, on what days did you play outdoors for at least 30 minutes or more? Do not count outdoor play during school hours.” […] Parent-reported child’s outdoor PA was measured by the question: “Most of the time does your child play outdoors for at least 30 minutes per day? Do not count outdoor play during school hours.”" p.318 11. "How many hours a week after school their child was active outdoors" p.2   "Cycling on the pavements, Cycling in their street, Cycling two on a bike, Cycling without a helmet, Cycling on busy roads, Crossing busy roads with friends, Crossing between parked cars on the road, Crossing busy roads alone, Crossing busy roads without using the traffic lights, Running on roads, Playing in their street, Playing near old derelict buildings, Playing on building sites, Playing near the railway, Playing on busy roads, Roller skating in the park or playground, Roller skating in their street, Roller skating on a busy road, Climbing trees, Climbing walls and Climbing buildings" p. 456 | 1. Indonesia, NA 2. UK, 9-15 years 3. USA, NA 4. USA, 7^th^ Grade 5. Australia, 12 years 6. China, 6-7 years 7. China, 15-60 years 8. Canada, 10-13 years 9. USA, Grade 5 10. Denmark, 9-11 years 11. Iran, 7-9 years | 1. Asmara 2016 2. Brewer 2011 3. Burriss 2011 4. Gomez 2004 5. Gopinath 2012 6. He 2015 7. Hu 2017 8. Nguyen 2018 9. Nicksic 2018 10. Nordvall-Lassen 2018 11. Soori 2000 |
|  | Outdoor life | | 1. Similar to "Friluftsliv" but "not exact translation". pg. 270 | 1. Sweden, NA | 1. Backman 2011 |
|  | Outward bound | | 1. “The history of outdoor adventure programs dates back to 1941, when Kurt Hahn founded Outward Bound (which can be called the first version of adventure programming) in Wales (Priest & Gass, 2005). Hahn proposed Outward Bound as a way to combat perceived social ills resulting from industrialization, such as the decline of fitness, initiative, spirit of enterprise, and self-discipline (Freeman, 2011; Hopkins & Putnam, 1997; Priest & Gass, 2005). The first Outward Bound courses focused on seamanship and intended to impart the ability in young male seamen to survive harsh conditions at sea in the merchant marine during war time by teaching tenacity, perseverance, leadership and confidence (Kelly & Baer, 1969; Miner & Boldt, 2002). In subsequent years, however, the range of outdoor activities also included, for instance, backpacking, mountaineering and canoeing, and the program's goals were refined, with greater emphasis put on personal growth and self-discovery (Freeman, 2011)" p.105 2. "wilderness expedition experience" p124 | 1. Germany, 14 years & University students 2. USA, children | 1. Mutz 2016 2. O'Shaughnessy 2013 |
|  | Physical activity | | 1. "Physical activity is an important health-related behavior, and it is a good candidate for behavioral assessment because it is easily observable. " pp. 401 2. “Any body movement produced by skeletal muscles resulting in a substantial increase over resting energy expenditure”. pg6479 | 1. USA, 4 years 2. International, children | 1. Hustyi 2012 2. Tremblay 2015 |
|  | Physical adventuring | | 1. "...is a process through which participants engage in adventure tasks such as initiatives, trust activities, and climbing challenges for the purpose of acquiring physical, cognitive, and affective skills and knowledge " p.146 | 1. UK, NA | 1. Wilenski 2014 |
|  | Sedentary behaviour | | 1. Any waking activity characterized by an energy expenditure ≤1.5 metabolic equivalents and a sitting or reclining posture” pg6479. | 1. International, children | 1. Tremblay 2015 |
|  | Sport | | 1. "… being 'owned' and controlled by adults" pp. 4 | 1. Canada, 6-12 years | 1. Holt 2008 |
|  | Structured activities | | 1. "Structured as well as unstructured activities like walking, cycling, and in particular outdoor active free play have been shown to be associated with children’s PA." pg 1 2. "A structured physical activity is an organized activity characterized by specific locations, time schedules, and adult supervision... it can also include team sports, raquet sorts and fitness classes" pp.57-58 | 1. Australia, parents of 2-12 years 2. NA, NA | 1. Bohn-Goldbaum 2013 2. Hyndman 2015 |
|  | Unstructured physical activities | | 1. Examples: "...included playground climbing frames, jungle gyms..." pg 2445 | 1. Multiple, NA | 1. Brussoni 2015 |
|  | | | | | |
| *Adventure* | | | | | |
|  | Adventure | | 1. "Merriam Webster defines adventure as “an undertaking usually involving danger and unknown risks,” “the encountering of risks,” and “an exciting or remarkable experience”. pg 32 | 1. USA, NR | 1. Bradshaw 2018 |
|  | a) Art Adventure | | 1. "... adventure and risk can be offered through a change in location, presentation of new media, or simply units or lessons that encourage students to play with ideas or media in new ways." pg 33 E | 1. USA, NR | 1. Bradshaw 2018 |
|  | | | | | |
| *Expedition* | | | | | |
|  | Expeditions | | 1. "...exploration and adventure, unfamiliar cultures or wild places, challenge and achievement." pg 219   "...gap years and expeditions are slightly different (as the former often incorporates the latter, but not vice versa." pg 220  "...‘expeditions’ refers to experiences of a month and longer, and primarily to expeditions in the wilderness (the main mediums being science work and adventure), mountaineering expeditions in the greater ranges (to climb high peaks or to undertake journeys at altitude) and expeditions to developing countries (typically involving community work and adventure)." pg 221   1. "An expedition is usually defined as an educational visit taking place overseas in Europe or further afield. It involves a deliberate element of challenge or adventure and requires specialist skills for its safe management. The environment that these expeditions take place in may include mountains, deserts, jungle, forests, rivers and sea." | 1. UK, NA 2. UK, NA | 1. Allison 2010 2. Council for learning outside the classroom 2019 |
|  | Wilderness expeditions | | 1. "Wilderness expeditions are a form of wilderness experience that offer opportunities for educational experiences and promote leadership and character development. They can vary in their format (e.g., educational field trip, stewardship course, outdoor education, or wilderness management program), duration (days to weeks or months), and personal growth focus (e.g., leadership, personal or organizational development), but the outcome is often enhanced self-esteem. Thus, wilderness expeditions might provide an important opportunity to promote self-esteem and nature connectedness in adolescents, both of which are important for well-being." pg 61 | 1. 1. UK, 11-18 years | 1. 1. Barton 2016 |
|  | | | | | |
| *Experience* | | | | | |
|  | Aesthetic experiences | | 1. “‘Aesthetic experience’ is defined by John Dewey (1934) as acrucial concept in experiential learning to emphasize emotions as a part of experiencing through the ‘normal processes of living’ (p. 10).” P. 108-109 | 1. Sweden, Grade 6 | 1. Manni 2017 |
|  | Creative play experiences | | 1. "Playgrounds should include a combination of equipment that supports development of gross motor skills and other elements that inspire interactive or exploratory play." pp.62 | 1. USA, NA | 1. Christoph 1999 |
|  | Holistic experience | | 1. "Rachel’s outdoor play involved her whole body and many of her senses. For Rachel, outdoor play was a verbal, cognitive, emotional, and kinesthetic experience. At the playground, she challenged herself physically by climbing to the higher level of the merry-go-round; she challenged herself emotionally by being “brave” enough to slide down the pole that she initially described as “scary.” When Rachel showed me how she climbed up to the slide, she counted out loud, indicating that playing involved both verbal and cognitive skills: “it just takes one, two, three, four, five.”" pg 46 2. "...‘holistic’ and progressive education by accentuating communication, social interaction, spontaneity, play, curiosity and fantasy." pg 205 | 1. Canada, 4 years 2. Denmark, 27-62 years | 1. Beattie 2015 2. Bentsen 2012 |
|  | Natural/nature experiences | | 1. Despite a plethora of articles in the popular and scholarly press on the benefits of nature, a careful reading of these works demonstrates a gaping hole in them – the lack of a consistent definition of ‘nature.’ While authors describe sites of nature experiences – gardens (Fröbel), ponds (Thoreau) or mountains (Wordsworth), or the activities undertaken there, there is seldom a systematic attempt to define nature. In addition, many researchers leave open the question of whether how their unspoken definition of nature interacts with childhood care settings." pg 1036 2. "Natural experiences such as collecting leaves, throwing stones in a pond, jumping over small brush or logs, building sandcastles, collecting sticks or nuts from the ground, or creating hiding spaces challenge the child’s imagination and reasoning abilities." pg 77 3. "nature experiences provide rich and varied sensory stimuli" pg 30 4. (indirect) "Teachers and parents use books, videos, photographs, artwork, models, and other such tools to expose their children to nature. As Kellert (2002) said, such vicarious or indirect experiences can be meaningful, however, not as a substitute for direct experience." p.74 5. (direct) "Direct, hands-on, and unstructured experiences outdoors [...] Direct experiences are also supported by emphasizing the local and ordinary experiences of nature, rather than seeking the exotic (Carson, 1956; Kellert, 2002). Looking for pebbles in a neighborhood lot, observing ants or worms in the backyard, or taking walks regularly in a community park are examples of local and everyday experiences". p.74 6. " Inspired by (Bentsen et al., 2009a), immersive nature-experience was operationalised as non-competitive activities, both sedentary and active, occurring in natural environments removed from everyday environments. This, for example, did not include competitive sports in natural environments or transport to and from work or school through natural environments. All motorised activities in natural environments were excluded. " p.2 | 1. Multiple, NA 2. USA, 3-12 years 3. USA, 3-6 years 4. USA, 1-3 years 5. USA, 1-3 years 6. Multiple (review), 0-18 years | 1. Brown 2017 2. Clements 2004 3. Desrochers 2001 4. Kharod 2015 5. Kharod 2015 6. Mygind 2019 |
|  | Learned experience | | 1. " ...five categories emerged within the theme of lived experience: full body engagement; interactions with nature; active learning; curricular connections; and acts of caring, respect and appreciation (Table 1)." pg 377 | 1. Canada, 4-5 years | 1. Coe 2017 |
|  | Outdoor experience | | 1. " Outdoor experience in schooling in Scandinavian countries has long been recognised as important and even central to the physical, emotional and intellectual development of children." pp.530 2. " Outdoor experiences allow students to see organisms in situ and gain a better understanding of ‘the associated landscape, the plants and animals that inhabit it, and the different processes that influence and link them’ and local geography." pp. 271-72 (Ernst 2014; Dolan 2016) | 1. UK, 7-11 years 2. NA, 12-18 years | 1. Humberstone 2011 2. Jose 2017 |
|  | Outdoor learning/play through experience | | 1. …"an [outdoor] extension of the indoor classroom, with children's social, emotional, cognitive, and physical skills developing outdoors just as they do indoors." pg. 31 | 1. USA, Kindergarten | 1. Hanvey 010 |
|  | Residential experiences | | 1. "Residential experiences are most effective where there are good links between schools and outdoor centres so that the contribution to the curriculum of residential experiences is clear and evaluated to guide future planning. Too often, however, such coordination is absent and this challenging environment is only recognised as a ‘one-off’ activity."pg 36 | 1. UK, NA | 1. Dillon 2005 |
|  | Wilderness experiences | | 1. "Most benefits of wilderness experience can be lumped into one of three categories: intrapersonal, interpersonal and environmental. Intrapersonal benefits accure from engaging in challenges. Interpersonal benefits are realized by wilderness experience participation in the company of a small group. The group struggles together through the adventure with a shared group goal as the potential outcome. The environmental benefits too and people also benefit from it Appreciation of nature and the outdoors involves people wanting to get closer to the wilderness environment and planet Earth" pp.10 | 1. NA, NA | 1. Spence 1992 |
|  | | | | | |
| *Recreation* | | | | | |
|  | Nature-based recreation | | 1. "Nature-based recreation (NBR), broadly defined as leisure activities occurring in natural settings such as hiking, birdwatching, surfing, or canoeing (Jackson, 1986; Larson, Usher, & Chapmon, 2018; Marques, Reis, Menezes, & Salgueiro, 2017)" p.683   (during childhood) ‘How frequently did you participate in leisure activities in contact with nature during childhood? (for example, visiting natural places, playing soccer or volleyball at beach, swimming, surfing, camping, hiking, etc.).’ p.685; "Following previous studies, we used one item to measure NBR participation during adulthood (Farmer, Brenner, Drescher, Dickinson, & Knackmuhs, 2016; Larson et al., 2011): ‘How frequently do you participate in leisure activities in contact with nature. Leisure activities in nature include visiting natural places, playing soccer or volleyball at beach, swimming, surfing, camping, hiking etc.’." p.685 | 1. NA, Mean 23.6 years | 1. Rosa 2019 |
|  | Outdoor recreation | | 1. Similar to Friluftsliv but not the exact translation; "Friluftsliv entails living and engaging in physical activity in the open air during leisure time in order to achieve a change of setting and to gain experience of our natural surroundings. Friluftsliv is ‘outdoor recreation with its heart within the land and linked to a tradition of being and learning with the land’." 2. "Outdoor recreation and environmental education claim a number of adventurous founding fathers, men such as John Muir, Teddy Roosevelt, Gifford Pinchot, and David Brower. Twenty years ago outdoor recreation researchers began searching for causes of the apparent under-representation of people of color among outdoor recreationists." pp. 127 3. "Outdoor recreation is also complex. Individuals may learn scientific facts while developing recreational skills. Many outdoor recreations activities such as bird walks, interpretative trails and nature camps appear to be purely educational, and in fact there are times when outdoor education and outdoor recreation overlap so much that they may be considered as synonimous". p.44 4. "The outdoor recreation scale measured the average frequency at which respondents were active in 6 different outdoor locations (yard, driveway, street, park, schoolyard, neighbor’s yard/driveway) never, once a month or less, twice a month, or once a week or more. " p.154 | 1. Sweden, NA 2. USA, NA 3. Canada, Grade 5 4. USA, Grade 6-12 | 1. Backman 2011 2. James 1996 3. Nicholas 1989 4. Roman 2013 |
| [Back to Table of Contents](#CONTENTTABLE) | | | | | |
|  | | | | | |
| **Environment/Location/Space** | | | | | |
| *General environment/space*Choose a building block. | | | | | |
|  | Backyard | | 1. Example: "The backyard includes a child-tended garden, water and sand experiences, wild areas and a multitude of flora and fauna." pg 1037-1038 | 1. Multiple, NA | 1. Brown 2017 |
|  | Biophilic cities | | 1. "...children’s environments that foster connections to nature, may be able to guide urban green space planning for proximate access to sensory rich nature experience." pg 22-23 | 1. Sweden (Adults) & Norway (1-6 years) | 1. Beery 2018 |
|  | Built environment | | 1. "...including the nature of the urban transportation system and resulting hazards and potential exposures..." pg 2056 2. "places and spaces created or modified by people" pg 26   " built environment features such as walk/bicycle paths, presence of cul-de-sac roads, access to parks, recreational facilities, other local destinations and public transport" pg 26   1. "neighborhood built environment (e.g., residential density, safety from traffic and access to goods and services)" pg 26 | 1. USA, 10-13 years 2. Multiple, <= 7 years | 1. Beyer 2015 2. Christian 2015 |
|  | Children’s places  (places for children) | | 1. "The first is ‘places for children’, which are adult defined rather than child defined as spaces that were planned for children." pg 1029   The second is ‘children’s places’. Rasmussen states the distinction between these two descriptions is that ‘a place, including “places for children”, becomes a “children’s place” after a child connects with it physically’." pg 1029  "According to the children, what made a place a ‘children’s place’ was its lack of adult surveillance" pg 1031 | 1. Multiple, NA | 1. Brown 2017 |
|  | Free space | | 1. Time in which 'free play' can take place. | 1. Norway, NA | 1. Aasen 2009 |
|  | Green gyms | | 1. "Active work in an outdoor environment, often with a focused conservation outcome." p.8 | 1. Multiple (Delphi), NA | 1. Shanahan 2019 |
|  | Green space | | 1. "...green areas..." pg 198 2. Used synonomously with 12, "natural area". 3. "Green spaces are considered to have restorative and relaxing properties enabling an ‘escape’ from urban living, in addition to providing spaces for exercise" pg 2 4. (Haven of green space) “… represents place as “a meaningful location”. This definition of place entails three elements of a location, a locale or material setting for interactions, and evoking subjective and emotional attachments." pg 2 5. "neighborhood green spaces (e.g., nature access, parks)" pg 26   Examples: "neighborhood greenness, access to nature, green public open space, parks and playgrounds". pg 28   1. "Green space is defined as “an area of grass, trees, or other vegetation set apart for recreational or aesthetic purposes in an otherwise urban environment” (Oxford University Press, 2017)" p.3 | 1. UK, 8-9 years 2. USA, 9-13 years 3. UK, 10-14 years 4. UK, 10-14 years 5. NA, <= 7 years 6. Multiple (review) 0-18 years | 1. Barton 2015 2. Beyer 2015 (Soc Sci Med) 3. Chiumento 2018 4. Chiumento 2018 5. Christian 2015 6. McCormick 2017 |
|  | Green environments | | 1. "...natural settings..." pg 197 | 1. 1. UK, 8-9 years | 1. 1. Barton 2015 |
|  | Green school yards | | 1. "Natural environments and green schoolyards that provide a diversity of landscaping and design features encourage outdoor free play and create opportunities for children to take risks and to be physically active." pg 375 | 1. Canada, 4-5 years | 1. Coe 2017 |
|  | Ecological garden | | 1. "This study was employed in an ecological garden (EG), which is semi-natural Mediterranean chaparral habitat, located within our university campus. The EG served as an outdoor teaching lab allowing us to find suitable ways to provide teachers with outdoor teaching experiences and encourage them to become reflective practitioners." pg. 246 | 1. NA, NA | 1. Tal 2009 |
|  | Farms | | 1. "Farms are as varied as the food we eat, providing opportunities for learning both in a formal context and as an opportunity for enhancing the social and emotional aspects of development. They may focus on livestock or arable production, or a combination, and be self-contained units or part of huge estates; There are also a number that have developed as community and city farms; These may be working farms, or smaller organisations that form a part of a community which can be visited by schools." | 1. UK, NA | 1. Council for EOtC 2019 |
|  | a) Youth care farms | | 1. Youth care farms are locations where youngsters and farmers’ families live and work together. In the Netherlands, some 100 youth care farms provide services to more than 200 youngsters annually (Hassink, Meyer de, Sman van der, & Veerman, 2011)." p.140 | 1. Netherland, 17-22 years | 1. Schreuder 2014 |
|  | Flexible environment | | 1. The adaptability in the play may be a consequence of the flexibility in the outdoor environment. Brown (2003) argues that a flexible environment creates a flexible child who can adapt and be resourceful in other situations. He argues that when an environment has flexible potential, it facilitates a child’s potential for curiosity, problem-solving and creativity." pg 561 | 1. UK, 3-5 years | 1. Canning 2010 |
|  | Garden | | 1. "Gardens are certainly filled with species we associate with nature – plants, birds and insects. However, nature is circumscribed by its relationship to the garden. In fact, the very idea of the garden implies an interaction between humans and the natural world. Gardens are nature tamed and rule-bound, filled with weedless, planned symmetry. Gardeners plant, tend and harvest, interacting with nature – but the plants are there on humans’ terms." pg 1037 2. (sensory garden) "refers to a small garden that has been specially designed to fulfil the needs of a group of people want to be involved in active garden and who also enjoy the passive pleasures of being outdoors amongst plants." pp.73 | 1. Multiple, NA 2. UK, NA | 1. Brown 2017 2. Hussein 2013 |
|  | a) Children’s kitchen gardens | | 1. "Gardens in schools and kindergartens to encourage engagement in growing one’s own food and to increase access to fruit and vegetables" p.8 | 1. Multiple (Delphi), NA | 1. Shanahan 2019 |
|  | Heritage sites | | 1. "The term ‘heritage site’ is a broad one, including museums, libraries, archives, science and exploratory centres, monuments, religious or public buildings, gardens or parks, archaeological sites and historic houses... Students may look at what heritage means to them, through investigating their school grounds as well as visits off-site, then gathering these ideas to make changes to their site. These investigations could be used to create features that reflect an aspect of local heritage, such as the species planted in a hedge or an artwork reflecting local industry. Heritage sites can support not only the history curriculum, but also fieldwork activities in science and geography, as well as provide inspiration for literacy and practical context for maths problems, and support for a host of other curriculum subjects." | 1. UK, NA | 1. Council for LOtC 2019 |
|  | Open spaces | | 1. "Open spaces were defined as green spaces and sidewalks." pg 60 | 1. USA, 0-3 years | 1. Dinkel 2019 |
|  | Open streets | | 1. "Open Streets are events usually lasting 1 day and are advertised to the community as a time to visit merchants, food trucks, group fitness classes, and other activities. Open Streets can include a Play Streets–style component as an activity hub or area to allow children and their families to participate in active play." pg 2 | 1. UK & USA, NA | 1. Bridges 2019 |
|  | Outdoors | | 1. "...outdoors as a constantly changing space, where variations in temperature, light, movement, colour, smell, texture etc. stimulate play." pg 290   In the outdoors "the sense of unpredictability and freedom create an environment where children can have an active role in learning, without feeling pressured to achieve a specific product or outcome. As Waite (2010) states, outside, children can acquire rich and complex information about phenomena through firsthand experience. Instead of learning through verbal, written or audio descriptions, transmitted by adults, the child has the possibility to directly experience situations, building knowledge based on real events." pg 290  "Outdoors, it is possible to deal with challenges and learn about risks, gaining a deeper knowledge of personal limits and abilities." pg 290   1. The outdoors, whether it be the natural environment or playgrounds specifically designed for children, is the ideal context to encourage children to be themselves, to explore, to experiment, to move and make the most of the opportunities offered in a less-restricted manner (Henniger, 1994; Rivkin, 1995; Zeece & Graul, 1993). 2. " Children learn best by doing. The outdoors, weather permitting, offers children as much opportunity for active learning as they have indoors. The outdoor environment can offer rich learning experiences not found indoors. The outdoors is also the ideal place to provide experiences that are sometimes considered too messy to do indoors. Sensory experiences such as measuring flour or mixing sand and water can be more fully explored without the limits of the indoor classroom." pp. 12 | 1. Portugal, 4 month-10 years 2. NA, children 3. NA, NA | 1. Bento 2018 2. Little 2008 3. Studer 1998 |
|  | Outdoor areas | | 1. "... equipped with structures for physical activity (e.g. slides, swings and climbing frames), loose materials (e.g. kitchen supplies, wooden blocks) and places for hiding. Different natural elements were present and available for children to explore..." pg 291 2. "Outdoor areas naturally lend themselves to literacy development in young children. Oral language and imagination are the only true pieces of equipment a teacher and student need outside to enhance literacy. The world of children is play and waiting outside our classroom doors are numerous opportunities for young children to learn and grow." pp. 23 | 1. Portugal, 4 month-10 years 2. NA, NA | 1. Bento 2018 2. Monsalvatage 2013 |
|  | Outdoor environment | | 1. "Outdoor play areas are available for children to play in, and they often have access to the wild landscape in the neighboring areas." "The outdoor environment in Norway provides multiple affordances for play"   "More vigorous than indoors and play forms take different group and gender constellations"  "Children are capable of making their own decisions in this environment where they can exert influence over their own and others’ actions, where their opinions are listened to and regarded as important"   1. "...an educational area..." pg 291 2. Example: "...varied terrain, access to playing equipment and natural material..." pg 9 3. Example: "courtyard space", "woodland setting" and "public access woodland setting". pg "finding a balance between providing for child-centred values whilst also offering guidance and direction for their learning and development. Lester and Russell argues that outdoor environments can provide that balance because children will look for some reassurance from practitioners but that outdoor spaces, especially new environments will provide personal challenge, stimulate curiosity and promote exploration. " pg 561 4. Example: "Local and community parks, playgrounds, and vacant ball fields..." pg 76 5. "in outdoor environments children can experience a greater sense of freedom than is generally possible indoors in early education settings. Additionally, children can experience nature and interact with elements of the natural environment outdoors." pg. 37   "a unique learning setting, supporting a range of activities different from those provided in indoor settings." pg 37  "young children can experience a great sense of freedom in this setting: freedom to exercise choice of activity, to express themselves noisily, to engage in messy activities; to experience disagreements and resolve conflicts with peers; and even to participate in forms of rough-and-tumble and dramatic play which, while often censured by adults, have considerable positive educational and developmental value. Furthermore, outdoors is an environment where children can experience the sights, sounds, smells, and textures of the natural environment, and develop awareness of, and delight in, the intricacies of nature." pg 37  "One distinctive feature of outdoors, particularly in comparison with indoors, is the potentially greater space and freedom of movement available for children. Another feature is the different type of equipment and materials that can be provided in outdoor settings. These features enable children to engage in a variety of large muscle activities, such as running, jumping, climbing, digging and the use of wheeled toys, such as bicycles, scooters, wheel-barrows, trolleys." pg 38 | 1. Norway, NA 2. Portugal, 4 month-10 years 3. Norway, 3-5 years 4. UK, 3-5 years 5. USA, 3-12 years 6. Australia, NA | 1. Aasen 2009 2. Bento 2018 3. Bjorgen 2016 4. Canning 2010 5. Clements 2004 6. Davies 1996 |
|  | Outdoor space | | 1. Examples of outdoor space in the article include: yards, playgrounds, open space and courtyards. The importance of "safe" and "secure" outdoor space close to the home is also discussed. | 1. USA, < 6years | 1. Barlette 1997 |
|  | Parks | | 1. "...natural environments such as urban parks..." pg 2056 2. "...included a playground, a large open area, and a sports field..." pg 2 | 1. USA, 10-13 years 2. Australia, parents of 2-12 years old | 1. Beyer 2015 2. Bohn-Goldbaum 2013 |
|  | a) Pop-up parks | | 1. "Pop-up Parks can occur on street segments, or parking lots, that have been closed to vehicle traffic, creating temporary public spaces that include temporary play structures, often resembling a park more than an open play space." pg 2 | 1. UK & USA | 1. Bridges 2019 |
|  | Safe enough environment | | 1. "We like to remove the hazard and not the risk’" pg 384 2. "Just as there is a symbiotic relationship among the children, environment and teacher at the Forest Kindergarten, creating a hazard-free risk environment appeared to require a similar connection among these elements. Teacher attentiveness, self-awareness, peer attentiveness and environmental safety checks helped to reduce potential hazards and increase safety by addressing both environmental and personal characteristics of risk." pg 384 | 1. Canada, 4-5 years | 1. Coe 2017 |
|  | Spaces of liberation | | 1. "...I am struck by this idea of liberation as a powerful theme in early childhood. This is relevant to urban settings in particular, but relates to the lives of most young children. They have very little freedom. Adults determine where they will go; what they will play with; when they get up, go to sleep, eat, and walk; and generally how they live their lives (Balke, 1997). Children are typically confined to hand holding or strollers—for their own safety, of course. I am not advocating that we let children race around unsupervised, but it is important that we think carefully about when and where it is safe to liberate them. When can we say “Run!” and let them take off on their own pursuits? These times are precious to the children and teachers. In these moments of liberation, we give children the freedom and space that they need to expend their energies and to act as autonomous individuals, worthy of freedom and its responsibilities (Rinaldi, 2001)." pg 152 | 1. USA, 3 years | 1. Bentley 2012 |
|  | Wilderness | | 1. "...wilderness, which has a legal definition, at least in the United States. The Wilderness Act of 1964 defines wilderness as ‘untrammeled by man’. It also adds that wilderness should provide ‘outstanding opportunities for solitude’." pg 1037 | 1. Multiple, NA | 1. Brown 2017 |
|  | | | | | |
| *Play Environment/Spaces* | | | | | |
|  | Environmental play yards | | 1. "encourage an active interaction with plants and animals, water and dirt, weather, and the life cycle, offer children education at its most compelling." pg.76 | 1. USA, NA | 1. Greenman 2003 |
|  | Experimental play areas | | 1. "These experimental play areas are different than the traditional playground in that they include novel forms, textures and climbing structures of different heights arranged aesthetically in an outdoor setting. Maniuplulative materials such as cardboard boxes, toys, sand, and water are provided for the child's innate, maniupulative curiousity. The area compoinents function to answer the child's need to explore and fantasize." pp. 22-23 | 1. USA, NA | 1. Yerkes 1988 |
|  | Fine motor play area | | 1. "Fine motor play areas were categorized as areas with small equipment (e.g. pails, shovels) that allowed for fine motor skills like grasping or pulling." pg 60 | 1. USA, 0-3 years | 1. Dinkel 2019 |
|  | Gross motor play area | | 1. "Gross motor play areas were defined as areas with large play structures that allowed for gross motor movements such as running or walking." pg 60 | 1. USA, 0-3 years | 1. Dinkel 2019 |
|  | Natural (nature) play spaces | | 1. "Natural play spaces have been shown to afford more opportunity for risky play than traditional playgrounds." 2. "e.g., woods, gardens" pg 2 3. Example: "planting native flora within the school grounds to more resemble a natural setting" pg 306 4. “For example, Fjortoft and Sageie found that, when children were provided with a garden containing ‘mixed vegetation’ and ‘varied topography’, they were far more likely to engage in physically active play such as jumping, running, climbing and crawling (Fjortoft and Sageie 2000). In another study, Fjortoft compared the play of two groups of preschool children: one group spent time every day playing on flat playground land, and the other spent the same period of time every day on uneven natural ground, surrounded by rocks and trees.” | 1. Canada, NA 2. Australia, 5-7 years 3. Australia, 1-12 years 4. Australia, 3-4 years | 1. Alden 2019 2. Bundy 2011 3. Cumming 2015 4. Nedovic 2013 |
|  | Outdoor play areas | | 1. "Outdoor play areas are the one special place for children to engage in motor, cognitive, and social skill development because of the spontaneous free play that occurs on the playground (Morgan, 2003)." p.12   "They need to provide a space for safe exploration, play with peers and comfortable seating for adults. However, many outdoor play areas for children are dull, show a striking dearth of natural elements, and often restrict children's play to a flat, rectangular, rubberized surface." pp.20  "Play areas should include: (1) motor experiences such as running, climbing, balancing, riding, jumping, crawling or scooting; (2) activities such as dramatic block play, block building, manipulative play or art activities; (3) exploration of the natural environments, including a variety of natural and manufactured surfaces such as non-poisonous plants, shrubs and trees" pp.21 | 1. USA, early childhood | 1. Olsen 2013 |
|  | Outdoor play environment | | 1. "The outdoor play environment should enhance every aspect of child development-motor, cognitive, social,emotional-and their correlates-creativity, problem-solving, and just plain fun". Pg.87 2. "Frost and Norquist (2007) perceive the outdoor play environment as an integrated learning environment of playgrounds, gardens, and natural habitats." p.30 3. "Outdoor play environments should have equipment that is safe and developmentally appropriate for the children served. The appropriate adult-child ratio should be maintained to prevent unnecessary accidents as well as provide the interaction needed to assist children in developing outdoor physical, social, and language skills. Each facility should have teachers with up-to-date CPR and first-aid training to take care of emergency situations." pg. 153 | 1. USA, NA 2. USA, NA 3. NA, NA | 1. Henniger 1994 2. McClintic 2015 3. Taylor 1996 |
|  | Outdoor play spaces | | 1. "Herrington et al. (2007) describe seven criteria for what characterises outdoor play spaces that support the development of young children and integrate the unique qualities of playing outdoors, called the Seven Cs (7Cs). The criteria describe outdoor play spaces that describe the play affordances. The 7Cs relate character to the characteristics of a physical environment, properties and general feelings and impressions (light, sound, colours or soft/hard materials), which are found in the environment and which influence people. The context involves how the play space has physical and visual interaction with its surroundings, in the play space and in relation to other surrounding areas. Connectivity refers to connections (physical and visual) in the play space that may create understanding of time, space and movement opportunities. This may be the link between outdoor and indoor space. Change relates to how the play space is perceived and used through changes in seasons, over the course of the year and in the age groups. Chance refers to the opportunities children have to create, manipulate, shape and leave imprints using different materials (sand, water, clay, snow, ice). Chance in the play space creates spontaneous challenge, manipulation and discovery, allowing the play space to be used in different ways according to the child’s size, age and resources. The clarity of the environment relates to readability and spatial legibility, and to children’s perceptual ideas of how the play space can be used. The environment and materials (hard/soft surfaces, noise in the neighbourhood, placement of toys and play apparatuses) influence how children play. The term challenge refers to how the play space creates physical and cognitive challenges and risk-taking. While the 7Cs were developed in Canada, the criteria are general so in this study they can be applied to understand how children use their outdoor play spaces in a Norwegian context to create physical activity." pg 2-3 2. Example: "The outdoor play space was a modern and well-equipped playground with fixed children’s equipment (e.g., slides, sandbox, monkey bars, swings), portable equipment and materials (e.g., tricycles, balls, small trucks), and significant open space (i.e., total area of 193 feet by 149 feet)." pg 130 3. "outdoor spaces are often more varied and less structured than indoor spaces." pg 48 4. Example: "play structures" pg 6 5. "Exploration of outdoor play environments within childcare settings has determined the vast majority of outdoor play spaces offer a multitude of play opportunities via playground structures, open surfaces, and loose parts (e.g. cardboard boxes, fabric)." pg 258   (creative outdoor play space) "First, children must have opportunities for healthy risk taking. Secondly, playgrounds must provide graduated challenges for young children. A third characteristic of good outdoor play spaces is the ability to promote a variety of play types. Finally, creative playgrounds should allow children to manipulate the materials and equipment found there." pg.10 | 1. Norway, 3-5 years 2. USA, 4 years 3. USA, young children 4. Australia, 2-4 years 5. USA, 0-3 years 6. USA, NA 7. NA, 0-3 years | 1. Bjorgen 2016 2. Brown 2009 3. Burdette 2005 4. Christian 2019 5. Dinkel 2019 6. Henniger 1994 (Young children) 7. Thigpen 2007 |
|  | Outdoor recreational spaces | | 1. "...recreational areas specifically designed to support children’s PA, such as playgrounds, swimming pools, and playfields, playgrounds..." pg 2 | 1. Australia, 2-12 years | 1. Bohn-Goldbaum 2013 |
|  | Play-rich environment | | 1. Quality of the "environments and materials for outdoor play". | 1. Canada, NA | 1. Alden 2019 |
|  | Play space(s) | | 1. "For a play space to be effective it needs to provide interest and give children choice so that they keep revisiting and exploring different ways to use the materials (Kadis 2007). " pg 561 2. Play spaces include: Basketball court, netball court, soccer pitch, athletic track, footbal oval, cricket pitch, pull-up, handball, bocce. pg 46 3. "play spaces such as the front yard and local park" pg 26 | 1. UK, 3-5 years 2. Australia/Turkey, primary school students 3. Multiple, <= 7 years | 1. Canning 2010 2. Chancellor 2014 3. Christian 2015 |
|  | Play street | | 1. "Play Streets are an intervention to help increase outdoor play by temporarily closing public streets (closures are recurring or episodic) to traffic, creating a safe place for active play." pg 1   "Play Streets, which are defined as recurring or episodic temporary street closures to traffic that provide the public with a no-cost, safe space to actively play and be physically active. These approaches are designed primarily for youth and may include various marked play areas, loose equipment, and/or group activities." pg 1  "Play Streets refer to the temporary closure (recurring or episodic) of public streets that for a specified time create a safe space for active play. Play Streets have been implemented as a stand-alone approach, or Play Streets–style components have been incorporated in other community activities or events such as Open Streets, Ciclovías, or Pop-up Parks as part of an activity hub or area." pg 2  "A Play Streets–style intervention was defined as recurring or episodic temporary closures of a street or public area to traffic to provide the public with a safe space to actively play  and be physically active that was publicly accessible at no cost, lasted for a specific time, included some form of adult presence and/or supervision, designed primarily for youth, and could have included marked play areas, loose equipment, and/or group activities." pg 3  "The most commonly implemented activities at Play Streets were various activities directed toward children, such as loose equipment for general play and sport equipment (eg, hula hoops, jump ropes, balls, hopscotch) (n = 17), cycling/biking (n = 5), fitness classes (n = 4), music (n = 1), dancing (n = 1), and running (n = 1)." pg 7 | 1. UK & USA, NA | 1. Bridges 2019 |
|  | Risky play environment | | 1. "Environment that affords or accommodates risky play behaviours" [69] pg 6429 | 1. Multiple, NA | 1. Brussoni 2015 |
|  | Unconventional play space | | 1. "The nature of an unconventional play space is that it is not a space designed for play at all. This is a college campus with no toys, bikes, or play structures designed for young children, and yet it is one of my students’ favorite places for outdoor play." pg 153   "When we take children into undefined spaces—open places where they are safe, but where they determine the stories, the games, and the many ways of playing—we offer them new opportunities for creation, storytelling, invention, and innovation. We offer environments that offer “the plurality of conceptions of what it means to be human and to be fully alive” pg 153 | 1. USA, 3 years | 1. Bentley 2012 |
|  | Water play centre | | 1. " Indoors or outdoors, a water-play center can be as simple or elaborate as budgets permit. Commercially made tables are available for water only or for water and sand combined... improvise with a baby bathtub or other low, stable container. Children should be able to reach into the water easily when the container is placed at child's table height. Ideally, the area should be large enough for three or four children to play at the same time... Carefully select materials to enhance water play with thought given to their potential for learning and, of course, for safety... Equipment that is frequently changed or modified renews interest and provokes divergent thinking." pg 30 | 1. NA (maybe USA), NA | 1. Crosser 1994 |
|  | | | | | |
| *Home Environment* | | | | | |
|  | Home yard | | 1. "...home-yard features (yard size, lawn quality, natural features, fxed and portable play equipment, and fowers and vegetables/herbs)..."   "On a typical day, how much time does your child spend playing in the yard or street around your house?" | 1. Australia, 2-5 years | 1. Armstrong 2019 |
|  | Home range | | 1. " Home range (or territorial range) indicates "the spatial extent and experiential variety of outdoor places inhabited. It embraces the totality of a child's space-time domain of familiar places close to home as well as a constantly expanding boundary condition, leading to unfamiliar challenging encounters in new places" (Moore and Young 1978). Home range is a dynamic phenomenon which develops and extends as the child interacts with its environment. The child seeks to extend its range in order to encompass new destinations with their associated novelty. Range extension is a discontinuous process, with large increases in range associated with events such as starting school or riding a bike. The development of the range is a more continuous process, as the child explores and transforms its territory over time. Natural places within the home range are a prime source of change and variety, providing children with settings that allow continual monitoring and interest." Moore and Young (1978) suggest three levels at which children may be involved with their territorial range. "Habitual range" represents close to home activities. It develops during the pre-school years starting with the private spaces immediately around the home, and then extending into the nearby public domain of local streets, playgrounds and wasteground. "Frequented range" incorporates those places visited perhaps at weekends or during holidays in a regular way. "Occasional range" relates to exotic places visited only rarely and under unusual circumstances. It is the habitual range of children that is the most important and interesting, especially in the study of younger children, whose play is almost totally restricted to places near to home and within the habitual range." p.114 | 1. Multiple (review), children | 1. Naylor 1985 |
|  | | | | | |
| *Learning Environment* | | | | | |
|  | Informal learning environment | | 1. "A school garden falls under the broad definition of an informal learning environment (Bell et al. 2009)...Learning experiences in these informal contexts are characterised as learner-motivated, interest-based, voluntary, open-ended, non-evaluative and collaborative." pg.299 | 1. USA, NA | 1. Fisher-Maltese 2018 |
|  | Natural learning environment | | 1. “...are the everyday experiences, events, and places that are sources of children’s learning opportunities, promoting and enhancing behavioral and developmental competencies.'' p.18 2. “...are the everyday experiences, events, and places that are sources of children’s learning opportunities, promoting and enhancing behavioral and developmental competencies.'' p.18 | 1. USA, Mean 2 years 2. USA, NA | 1. Dunst 2006 2. Dunst 2001 |
|  | Outdoor learning environment | | 1. "...main role of using the outdoor learning environment within the learning process is to directly experience concrete phenomena and materials as they appear in the real world. This point becomes even more crucial when dealing with younger pupils whose abstract thinking abilities are still relatively undeveloped." 2. From Table 3: "Best practice indicators for a model outdoor learning environment: PA is supported by OLE (running, jumping on/off, crawling through, rolling, swinging, throwing, balancing, climbing); Open, grassy area for games & events; Wheeled toys, portable play equipment, & play materials available; Natural, loose materials (leaves, sticks, gravel, seeds) available for play; Sufficient man-made shade structures; Trees provide cover for about 1/3rd of outdoor area; Looping, curvy primary pathways for circulation and wheeled toys; ≥ 10 outdoor play & learning settings for activities; Outdoor classroom/program base/storage available for tools, equipment & materials for outdoor learning; Designated vegetable garden; Shrubs (3 for every 100 sq. ft), including ≥1/4 fruiting shrubs & vines; ≥ 1/4 of trees are edible fruit or nut species" pg 8 3. "Outdoor learning environments are where children connect to the real world through exploring their surroundings, communicating with others, learning and trying new things." pg 44 4. (outdoor learning play environment) Enhanced features include:   "Standards for features of outdoor learning environments including a) number of outdoor learning features, b) number of gross motor activity features, including looping pathways, c) natural features in the outdoor environment that enrich children’s play and learning such as: Non-toxic trees, shrubs, or vines; topographic variations (such as mounds, terraces, lopes); a variety of ground surfaces (mulch, grass, pebbles); smooth rocks, wood or logs; non-poisonous flowering plants or garden plants and vegetables; birdfeeders, bird baths and birdhouses." pg 90  Texas example:  "The outdoor environment provides children with the opportunity to care for living things and appreciate nature/beauty such as: Non-toxic trees, shrubs, or vines; topographic variations (such as mounds, terraces, slopes); a variety of ground surfaces (mulch, grass, pebbles); smooth rocks, wood or logs; non-poisonous flowering plants or garden plants and vegetables; birdfeeders, bird baths and birdhouses.  Outdoor environment and natural and manufactured equipment/materials, provides partial shade, motivates children to be physically active and engage in active play such as balancing, climbing, crawling, moving, pushing/pulling, riding, walking, and running. Key elements may include: balls, swings, balance beams, climbing structures, tumbling pads, tricycles or riding toys, marching music, jump ropes, space to skip, hop, and roll.  Natural outdoor environment supports social emotional development including but not limited to areas that invite social gatherings, tummy time, dramatic play, group games, music and movement, and spaces for quiet and calm activities. Key elements may include: Natural additions such as boulders, tree stumps, sand area and benches, design elements such as stages, platforms, wind chimes, canopies, teepees, gazebos." pg 91   1. A developmentally appropriate outdoor learning environment designed for and by children combines child development concepts, space ad movement organization, creative design and the children's wishes, desires and interests to make ideal play and learning environment for children." pp.15 2. "The EYLF (Australian Government Department of Education, Employment and Workplace Relations (DEEWR), 2009) provides an overview of how outdoor learning environments and play are viewed within this framework as follows: Outdoor learning spaces are a feature of Australian learning environments. They offer a vast array of possibilities not available indoors. Play spaces in natural environments include plants, trees, edible gardens, sand, rocks, mud, water and other elements from nature. These spaces invite open-ended interactions, spontaneity, risk-taking, exploration, discovery and connection with nature. (pp. 15–16)" p. 86 3. "…, an OLE is more than an outdoor playground. Instead, it is an outdoor learning space that includes intentional interactions with elements of nature (e.g. plants, animals, rocks, dirt, water, etc.). OLEs provide children with the opportunity to explore, be active, and engage in sensory experiences that simply cannot be replicated inside. Outdoors, children are free to run, be loud, engage in rough and tumble play, and take calculated risks. As a result, outdoor learning can lead to increased confidence, enhanced gross motor skills, creativity, and improved collaborative skills" pp. 86 | 1. Israel, 4^th^ grade 2. USA, 0-5 years 3. Australia/Turkey, primary school students 4. USA, NA 5. NA, NA 6. Australia, early childhood 7. Singapore, Kinder teachers | 1. Assaraf 2010 2. Byrd-Williams 2019 3. Chancellor 2014 4. Cooper 2015 5. Guddemi 1992 6. Little 2017 7. Strachan 2017 |
|  | Playful learning environment | | 1. "A PLE is an outdoor playground environment designed for educational purposes that is situated in the schoolyard and supports ColPlay, games and other physical activity." pp.392 2. “The playful learning environment (PLE) is a novel, pedagogically validated learning environment which combines information and communication technologies (ICTs) both in the classroom and outdoors in the playground. The PLE is also referred to as SmartUs, a technology-enriched playground system based in a combination of scientific research from the fields of education, physical exercise, technology and industrial design (see www.smartus.fi)." p.2 | 1. Finland, adults (25-53 years) & children (Kidner to 6^th^ Grade) 2. Finland, 7-12 years | 1. Hyvonen 2008 2. Kangas 2010 |
|  | Traditional learning space | | 1. "...traditional learning space, such as the classroom..." pg 101 | 1. Sweden, Finland, Latvia, Germany , Greece, 0-25 years | 1. Brodin 2009 |
|  | | | | | |
| *School Environment* *(N = 17)* | | | | | |
|  | Built environment for learning outside the classroom | | 1. . "The built environment is made up of historic and contemporary buildings and the spaces between them, including parks, streets and housing. Learning about the built environment is about learning to see the value of well designed spaces and the relationship between the natural environment and local community. We can learn about the built environment in the school grounds as well as in our cities and streetscapes." | 1. UK, NA | 1. Council for learning outside the classroom 2019 |
|  | Nature school yards | | 1. "Natural schoolyards, native ecological communities on school grounds, provide a space for students to interact with their natural environment" p.1 | 1. USA, Grade 7 | 1. Nelson 2013 |
|  | Recess | | 1. "normal morning or afternoon recess" pg 130 2. "During recess, children engage in real-life situations and confront relevant problems. Situations may involve playing an organized game with peers, gaining access to playground equipment, or initiating a relationship for companionship." pg 2   "Not confined to traditional classroom tasks, children freely demonstrate noncurricular strengths. As children explore the outdoor environment, they test their abilities. During recess and in the outdoors, children acclaim one another for their running, jumping, swinging, and climbing abilities." pg 3  "Recess is defined as blocks of unstructured time, typically outdoors, when children freely choose activities and playmates. Recess allows for creativity, freedom, and independence. Kieff (2001) describes high-quality recess experiences as those where children of all ages engage a variety of choices in their activities." pg 4  "The unstructured nature of recess and outdoor play" pg 2   1. "breaks during the school day" pg 42 " recess is the time when children have freedom to choose what they do and with whom they can play" pg 50   "recess breaks have an important role in facilitating children playing independently" pg 50   1. "Ramstetter, Murray, and Gardner (2013) issued an American Academy of Pediatrics policy statement positioning recess as a vital component within school curriculum and healthy child development" pg 10   "recess offers its own unique learning opportunities." pg 10   1. "Recess provides a break from classroom time and promotes learning behaviors, problem-solving skills, and learning readiness.5,11,12 Recess also offers children the opportunity to engage in physical activity on a daily basis.13" p.406 2. "Recess affords an opportunity for physical activity, generally occurs multiple times over the course of a day, and accounts for nearly a quarter of the average school day in primary grades (1)." 3. The term recess has often been thought of as time spent without any real purpose (Clements, 2000) 4. "Free time on the playgroung during during the school day is perhaps the most universal opportunity for children in Canadian elementary schools to be vigourously active in a social setting. The typical 15-minute recess breaks from classroom activity in the morning and afternoon, coupled with other opportunities at lunch, and before and after school, provide children the time to take part in active play with friends and classmates." p.42 | 1. USA, 4 years 2. USA, NA 3. Australia/Turkey, Primary school students 4. USA, Kinder to 4^th^ Grade 5. UK, 7-11 years 6. USA, 4-5 years 7. USA, early childhood 8. Canada, Grade 1-4 | 1. Brown 2009 2. Burriss 2011 3. Chancellor 2014 4. Clarke 2018 5. Knowles 2013 6. Nicaise 2012 7. Olsen 2011 8. Spencer-Cavaliere 2009 |
|  | Outdoor school recess | | 1. Takes place in a playground setting… recess is one of the few times during the school day when children have the opportunity to socialize with their peers in the absence of adult supervision" | 1. UK, Kindergarten to 8^th^ Grade | 1. Holms 2012 |
|  | School garden | | 1. The school garden contains elements from the adults’ real world. The way it is created, organised and functions expresses structures and ethical approaches of the society to which it belongs . In this sense, a school garden can also be an educational portal to ‘real’ life, reinforcing understanding of our surroundings, but also contributing to a commitment to manage them more wisely for the next generation." pg422. | 1. UK, NA | 1. Stavrianos 2016 |
|  | School ground | | 1. "...potential resources for various activities in understanding about environment...consists of various elements, both natural and man-made, that may become a micro representation of our larger scale environment and therefore become a potential setting for learning." pg. 101   "...offer potentials as ‘outdoor classrooms’ (Malone & Tranter, 2003a) that provide learning resources for various aspects of environment. In particular, the school ground could play important role in providing sensory stimulation, opportunities for action and response feedback (Wohlwill & Heft, 1987), which are all necessary to develop children’s engagement with nature and acquisition of environmental knowledge. School could develop learning programs that utilize school ground as a setting for science and environmental learning (McKendrick, 2005). School ground becomes a place for learning about living habitat and various environmental process as well as for developing environmental awareness and stewardship (Education Development Center, 2000)." pg 102   1. (and immediate surroundings for LOtC) Examples of school ground/immediate surroundings LOtC activities: "The range of possible activities may be limited by the size and nature of the site; even so most school grounds can offer some or all of the following possibilities: play areas — for problem-solving/team-building games and activities; habitats such as playing fields, hedges, meadows and ponds — for field study and science; school garden or growing areas — for science, sustainability and food education; whole site — for orienteering, outdoor literacy (e.g. storytelling) and practical numeracy; activities, visual and performing arts (e.g. murals, sculptures, mosaics, music and drama); paved areas — for D&T outdoor experiments; wooded areas — for Forest School activities; playing fields — overnight camping experiences; playground equipment and climbing/traversing walls — for adventurous activities. " | 1. Indonesia, NR 2. UK, NA | 1. Atmodiwirjo 2013 2. Council for learning outside the environment 2019 |
|  | School yard | | 1. "The schoolyard as an option appeared among the answers of only a few teachers in both groups. An appropriately designed school yard can offer a number of educational opportunities, such as finding out plants, raising awareness of wildlife conservation, aesthetic training and more. Children can plant their own garden and by maintaining it develop their sense of duty and responsibility is developing." pg 436 | 1. Hungary/Serbia | 1. Borsos 2018 |
|  | School play yard | | 1. "a place for doing (opportunities for physical activities); a place for thinking (opportunities for intellectual stimulation), a place for feeling (to provoke a sense of belonging) and a place for being (to allow them to be themselves). Her research focused on the value of improved school playgrounds as an educational resource to demonstrate how students’ attitudes, behaviours and learning skills could be enriched. From the above view, if designed well, the school garden provides a valuable resource for a wide range of users ." pg. 422 | 1. UK, NA | 1. Stavrianos 2016 |
|  | School yard restoration | | 1. Schoolyard restorations, such as rain gardens or butterfly gardens, increase the ecological services (e.g., storm water management, wildlife habitat) of school grounds (WDNR, 2013) while providing teachers with a natural habitat to teach on the school site." p.1 | 1. USA, Grade 7 | 1. Nelson 2013 |
|  | | | | | |
| *Playground* | | | 1. "...playground with complexity and variety of elements, furnished with..." (6/7.) pg 4 2. Example: "...a large area surrounding the kindergarten that consists of ordinary swings, sand pits, a climbing tower and slides, trees, a mix of grassy ground and sand/asphalt, and varied terrain for sledding and rolling on the ground" pg 308 3. Example: "...a fenced area with soft-fall flooring and containing multifunction apparatuses, swings, slides, and other equipment..." pg 2 4. Example: "modern and well-equipped playground with fixed children’s equipment (e.g., slides, sandbox, monkey bars, swings)" pg 130 5. Example: "Slides and climbers..." pg 2 6. "describe the playground as a learning place with physical and social skills very highly rated... a place for formal lessons" pg 52 7. "Playground on a raised woodchip-filled area, with mowed grass and trees in background" pg 36 8. "become territories of child activity and creativity because of their freedom, openness, absence of control, and presence of entertaining and developing equipment." pg.3407 9. "A playground should be like a small scale replica of the world, with as many as possible of the sensory experiences to be found in the world included in it." pp. 36 10. "Playground are essentially social environments, and as such provide both the physical and interpersonal resources that allow for children to meet, interact, and form relationship with peers." pp. 27 11. "Playgrounds are designed to stimulate play." pp. 42 12. (outdoor playground) "… serves as children's favourite social area where they can interact, develop friendships and release stress." pp. 55 Wells and Evans 2003 13. "Playgrounds are places for children to safely explore experiences that are out of the ordinary…. The playground is there to allow for further exploration, in the context of play, that will help children stretch their physical and intellectual abilities, social and emotional skills, and learn some basic principles that can be applied to life and in the world." pp. 20 (Owens 1997) 14. "Researchers have also found that aggressive playground behaviour of elementary school students is reduced when unstructured play or competitive games (where there is a winner and loser) are replaced by organized non-competitive games." pp. 15 15. "Playgrounds still more often than not have 'adventure' structures with ropes, bridges, swings and climbing apparatus with artificial surfaces and minimal green space, void of diversity of vegetation and wildlife." pp. 125 16. "The playground is made up of traditional items including three basketball courts, sand areas, soccer fields and tether ball. A wall for hand ball, an activity very popular among the students, is being constructed since previously students used windows-less back wall of the school. Kindergarteners have a separate playground area with an age-appropriate climbing apparatus. Two other climbing apparatus, one for the first and second grades, the other for grades three through six, are located on the main playground. There are no swings because they were deemed to be a major liability risk. When students hit the playground, play time takes a twist. Playing games like hopscotch and four square becomes a learning experience." pp. 62 17. (outdoor playground) "Outdoor playgrounds are designed to facilitate children's play and aimed at enhancing children's physical, social, emotional and cognitive development." pg. 447 18. (typical playground) "The typical playground, which has changed little in basic design since the 1930s consists of swings, slides, monkey barrs or other climbing equipment, seesaws, and a merry-go-round cemeneted into asphalt pavement." pp. 162 19. "American playgrounds have gone through an evolution from metal, gross motor, and functional equipment on concrete or asphalt pads to contemporary playgrounds of linked structures, slides, monkey bars, swings, platform bridges, and step" pp. 29 | 1. Norway, 3-5 years 2. Norway, 3-5 years 3. Australia, parents of 2-12 years 4. USA, 4 years 5. Australia, 5-7 years 6. Australia/Turkey, primary school students 7. USA, 3-6 years 8. Russia, NA 9. NA, NA 10. NA, NA 11. NA, NA 12. China, Kindergarten 13. NA, NA 14. NA, NA 15. NA, NA 16. NA, NA 17. Norway, 3-5 years 18. NA, NA 19. USA, NA | 1. Bjorgen 2016 2. Bjorgen 2015 3. Bohn-Goldbaum 2013 4. Brown 2009 5. Bundy 2011 6. Chancellor 2014 7. Desrochers 2001 8. Fillipova 2016 9. Greenman 1993 10. Hartle 1993 11. Herlain 1995 12. Hu 2015 13. Hudson 2001 14. Kilty 2001 15. O'Shaughnessy 2000 16. Rittner-Heir 2001 17. Storli 2010 18. Ward 1987 19. Wardle 1998 |
|  | Adventure playground | | 1. "Introduced in Denmark during World War II, these fenced in sites contain a wide variety of informal play settings, such as tree houses, gardens, forts and sandboxes, as well as a variety of scrap materials and tools for children to use in imaginative and constructive play activities. Building clubhouses, cooking, gardening, and taking care of animals are some of the activities found in adventure playgrounds." pp. 24 2. “Adventure playgrounds” may be a potential solution to providing safe play environments that afford opportunities for risk taking... Adventure playgrounds provide child-centered and child-directed play spaces where children create and modify their own environments. Children have access to raw materials such as building supplies and tools, as well as sand, dirt and water. In some cases, adventure playgrounds include trained play workers and volunteers for supervision and “professional scaffolding” that facilitates children’s play and removes play barriers. Different opportunities exist for children of varying developmental levels and interests to try new things, such as climbing that is graded for developmental requirements, allowing children to select risk they are comfortable with. Some adventure playgrounds in proximity to farms or community gardens provide children with the opportunity to interact with and care for animals, and grow and cook their own food." pg 3141 3. "There is no equipment, as such, in the park. Instead, kids are confronted with boards, spare tires, telephone poles, and lots and lots of mud." pg 34; "When kids enter the park, each child must pick up "dangerous" objects, like pointy boards with nails in them, before, they can have access to the park and its tools. " pg 34 4. "This is an exercise structure constructed by adults for children. One school, after considering data that showed this to be true, enriched their playground by creating zones led by classroom teachers. All over the playground, equipment was placed ready for use. This included cushions with books on the verandah, pickup sticks, marbles, stilts, skateboards, chess sets, cards, dress up clothes, skipping ropes with senior students to teach new skipping rhymes, frisbees, hula hoops, sand toys and hoses in the sandpit, water play troughs and easels for painting. All this, as well as two adventure playgrounds and the usual team games on the big fields, resulted in students who couldn’t wait to get outside into a place that allowed them choices for play." pg 39 5. "A highly informal playground within a fenced area, stocked with scrap building material, tools, provisions for animals and cooking." pp. 42 6. "re constructed from the natural environment and contain areas for playing games, ﬁreplaces for cooking and garden sand construction areas. " pp. 82 7. "Originally called junk playgrounds, adventure playgrounds adhered to a clear element of recycling in that children were making use of whatever junk was lying around in their environment for play. Most of the contemporary adventure playgrounds rely on community and business donations for play-related resources, such as tools, wood, and other construction scrap material.[...] The places where such adventurous play experiences may occur are unstructured, outdoor play spaces that allow for innovative, creative, and child-directed play to transpire. These are places where children can build dens, huts, and artistic structures with tools and scrap materials in a clearly designated place that is fenced off and attended to by a playworker. On some adventure playgrounds children also have the opportunity to care for animals and work in community gardens. They can cook over open fires and play in sand, water, and dirt. In contrast, in a North American context the word adventure generally refers to an adventurous play setting either indoors or outdoors, with ready-made play structures in controlled play environments. In North America, modified types of adventure playgrounds in the form of city community gardens, nature interpretive centers, and interactive youth centers have become multipurpose gathering places for children, youth, and, to some degree, also adults. These adapted forms of adventure playgrounds are adult structured and primarily used for outdoor educational purposes." p.269 | 1. USA, NA 2. Multiple, NA 3. USA, NA 4. New Zealand, NA 5. NA, NA 6. Oman, Kinder 7. Multiple (review), children | 1. Brett 1993 2. Brussoni 2012 3. Clendaniel 2009 4. Couper 2011 5. Herlain 1995 6. Ihmeideh 2016 7. Staempfli 2009 |
|  | Contemporary playground | | 1. "Typically designed by a professional architect using manufactured equipment, usually wood, expensive stone, and timber terracing; intended to have high asthetic appeal for adults." pp. 42 2. "covered with polyvinyl chloride plastic or ﬂoor coverings such as wood chips, grass or alternative artiﬁcial surfaces and." pp. 82 3. "Contemporary playground (joined pieces of pressure-treated lumber, slides, tire ladders, tire swings." pp. 40 4. "The natural playground (1.7 ha) is bigger than the actual contemporary playground (0.15 ha). Nevertheless, it is possible to compare the two as the adjacent park of the contemporary play area is open for playing and there are no fences or borders to conquer. Since the characteristics of the different playgrounds can be determinants for activity, a detailed playground description with typical activities is essential. The dimension of physical elements of natural playgrounds and contemporary play environments and all its affordances is adequately covered by Heft (1988). As specific physical elements of the playgrounds afford certain activities, based on Heft (1988), physical features and actualized affordances will be presented for the natural and the contemporary playground of the study (Table 1)." p.284 | 1. NA, NA 2. Oman, Kinder 3. NA, NA 4. Germany, 5-6 years | 1. Herlain 1995 2. Ihmeideh 2016 3. Jordan 1994 4. Luchs 2018 |
|  | Creative playground | | 1. "Communities in the United States have a long history of so-called creative playgrounds. Such playgrounds typically use 'scrounged materials' such as telephone poles, railroad ties, and rubber tires." pp. 24 2. "are coordinated and designed or adapted for a site. These include both modular coordinated play installations and one-off architectural designs which have a sculptural or natural quality in the forms and materials... Creative playgrounds can support a variety of motor and social play in a compact area, if they include moving parts. They are usually attractive to both adults and children. They rarely, however, have any provisions for loose parts." pg.75 3. (also Adaptive playground) "A semiformal environment combining features of the other types of playgrounds to meet the needs of a specific program." pp.42 | 1. USA, NA 2. USA, NA 3. NA, NA | 1. Brett 1993 2. Greenman 2003 3. Herlain 1995 |
|  | Designer playground | | 1. "Professional architects or designers are responsible for these playgrounds, which typically include equipment with a wider range of functions involving climbing, exploring, and participating in imaginative and creative play. Asthetic objectives are often emphasized in this type of play setting." pp. 24 | 1. USA, NA | 1. Brett 1993 |
|  | Early playground | | 1. "These early playgrounds were simple—a sandbox, a basic climbing structure—but they reflected a desire to give children a place to teach themselves about the world." pg 32 | 1. USA, NA | 1. Clendaniel 2009 |
|  | Kit, Fence, Carpet (KFC) playgrounds | | 1. "...limited appeal and affordances for play. KFC playgrounds have been rated as having inferior opportunities for promoting children’s emotional, social, physical and cognitive development." pg 46265 | 1. Multiple, NA | 1. Brussoni 2015 |
|  | Loose parts playgrounds | | 1. " There is compelling evidence that playing in natural settings has specific benefits beyond those associated with free play." pg 35 2. "Apart from the slide built into a rock formation, there is barely any physical equipment. Children can play virtually anywhere, from the pebbles that line the paths, to sand- and water-filled areas. They can make their own fun. On a recent weekend at Teardrop, kids were running up and down the rolling hills and rock formations, delighted." pg 35 | 1. USA, NA | 1. Clendaniel, 2009 |
|  | Nature playground | | 1. "...a playground that emphasized the imaginative power of young children, instead of just doing a slick architect's take on the formulaic playgrounds" pg 36   "...a winding wooden ramp, a sandbox, and a water area, there are hundreds of blue foam blocks of various shapes and sizes. Stored in a container managed by a full-time staffer, the blocks are available to children, who can use them to build whatever they want." pg 36   1. (Naturalized playground) "where children have access to trees, plants, rocks and other natural objects such as tree stumps, which are quite popular, are becoming more and more common." p.15 | 1. USA, NA 2. Canada, NA | 1. Clendaniel, 2009 2. Trudeau 2001 |
|  | Purpose-built playground | | 1. "...‘managed’ spaces..." pg 5 "...adult-designed..." pg 5 | 1. UK, 10-11 years | 1. Brockman 2011 |
|  | Traditional playground | | 1. "Consists of formal playground with steel equipment - jungle gyms, steel swings, slides, and the - set in concrete. Traditional playground emphasizes gross motor activities, but offer children little chance to play imaginatively or creatively." pp. 24 2. Example: "As Griffin (1992) describes, typical built playground can also be the site of natural inquiry and first-hand experience to the extent that teachers take advantage of ‘teachable moments’ to connect with trees in playgrounds, roots in the sandbox, the cycle of leaf growth on a particular tree or planting seeds.: pg 1038 3. ( OR standard American playground) "But what the new, safe equipment is missing, of course, is the stuff that, according to Moore, makes play fun and crucial to early-childhood development: variety, complexity, challenge, risk, flexibility, and adaptability." pg 33 4. "are open areas dotted with various pieces of unrelated commercial, usually metal, large muscle equipment such as slides, swings, climbing domes or bars, spring animals, seesaws, and so on." pg.75 5. "Traditional playgounds are typically the "obstacle course" design. Each piece is stationed and used seperately with nothing to invite continuance." pp.16 6. “Typically flat, barren area equipped with steel structures such as swings, slides, seesaws, climbers and merry-go rounds, fixed in concrete and arranged in a row." pp. 42 7. "traditional playgrounds which consist of an open area and are covered with asphalt or simply have dirt or grass surfaces with some play equipment. " pp. 82 8. "Traditional playground (swing set, slide, merry-go-round and four seater seasaw.") pp. 40 9. "flat barren, often covered with asphalt and equipped with climbing bars, a swing, a seesaw, and a slide" pg447 10. "With slide, swings, and sandbox - however traditional playgrounds affords children less cognitive and social play opportunities than 'creative' or 'adventure' playgrounds" pp.22 (Hayward 1974) | 1. USA, NA 2. Multiple, NA 3. USA, NA 4. USA, NA 5. NA, NA 6. NA, NA 7. Oman, Kinder 8. NA, NA 9. Norway, 3-5 years 10. USA, NA | 1. Brett 1993 2. Brown 2017 3. Clendaniel 2009 4. Greenman 2003 5. Guddemi 1992 6. Herlain 1995 7. Ihmeideh 2016 8. Jordan 1994 9. Storli 2010 10. Yerkes 1988 |
|  | | | | | |
| *Landscapes* | | | | | |
|  | Forest landscape | | 1. "diverse backdrop of trees, rocks, hills and other natural structures present an ideal environment in which children’s risk-taking and risky play experiences can flourish and thrive." pg 374 | 1. Canada, 4-5 years | 1. Coe 2017 |
|  | Natural landscapes | | 1. "Fjortoft and Sageie (2000) point out that the natural landscape has qualities that meet children’s needs for diverse and stimulating play environments." pg 52 | 1. Turkey & Australia, Primary school children | 1. Chancellor 2014 |
|  | Playscape | | 1. "different play environments." pg.112 | 1. Norway, 5-7 years | 1. Fjortoft 2001 |
|  | Therapeutic landscapes | | 1. "Gesler's concept of therapeutic landscapes encompasses notions of places associated with treatment, healing, and health promotion (Gesler, 1992). The natural world has long been associated with health and described as a therapeutic landscape." pg 254 | 1. USA, 9-13 years | 1. Beyer 2015 (Soc Sci Med) |
|  | | | | | |
| *Nature Environment* | | | | | |
|  | | Nature | 1. "...“trees,” “dirt,” “animals,” “anything green,” “bugs,” “leaves,” “sticks,” “wood,” “flowers,” “grass,” and “flower fragrance.” pg 256 2. "For the Romantic poets, nature had a very practical effect: it brought one closer to God." pg 1037   "Human beings exist in nature anywhere they experience meaningful kinship with other species. By this description, a natural environment may be found in wilderness or in a city; while not required to be pristine, this nature is influenced at least as much by a modicum of wildness and weather as by developers, scientists, beer drinkers, or debutantes. We know this nature when we see it." pg 1036  "The discussion of nature here is not limited to those faraway, vast and pristine places designated as ‘natural areas’ by some government authority. Nature includes parks and open spaces, meadows and abandoned fields, street trees and backyard gardens. We are referring to places near and far, common and unusual, managed and unkempt, big, small and in-between, where plants grow by human design or even despite it." pg 1037   1. "Nature seems to be inherently important - even 'natural' to children. They know what to do in and with nature. They need guidance, but not rules and guidelines." pp. 12 2. “The phenomena of the physical world collectively, including plants, animals, the landscape, and other features and products of the earth, as opposed to humans or human creations” pg6479 | 1. USA, 9-13 years 2. Multiple, NA 3. USA, NA 4. International, children | 1. Beyer 2015 (Soc Sci Med) 2. Brown 2017 3. Keeler 2008 4. Tremblay 2015 |
|  | Natural areas | | 1. (urban setting; used interchangeably with natural environments) "...urban settings where natural areas are more challenging to access." 2057 2. "Gesler's concept of therapeutic landscapes encompasses notions of places associated with treatment, healing, and health promotion (Gesler, 1992). The natural world has long been associated with health and described as a therapeutic landscape." pg 254 | 1. USA, 10-13 years 2. USA, 10-13 years | 1. Beyer 2015 2. Beyer 2015 (Soc Sci Med) |
|  | Natural environment(s) | | 1. "...wild spaces (e.g. forests) afford natural opportunities for risk such as trees to climb and slopes for sliding down, as well as naturally available loose parts such as sticks, brush, and snow." 2. "Natural environments provide large open green spaces for activity and can create more imaginative and inventive play than urban environments lacking natural features." pg 197 3. "...valuable source for diverse learning and diverse play habitats for children. Natural environments represent spaces where children see opportunities and challenges according to the qualities of the natural setting, and then use them functionally." 4. "Natural environments facilitate greater risk-taking in play allowing children to test their limits, try new skills, build confidence and develop their fundamental movement skills which supports physical activity participation. Compared with traditional ECEC playgrounds, natural environments encourage young children’s physical and mental development and physical activity." pg 2 5. "...neighborhood walkability or liveability, street connectivity, sidewalks, lighting, transit, and esthetics and domains of early child development such as language, cognitive and communication skills." pg 30 6. "Natural environments and green schoolyards that provide a diversity of landscaping and design features encourage outdoor free play and create opportunities for children to take risks and to be physically active." pg 375 7. (Also field studies) "Learning outside the classroom in the natural environment can encompass a range of places or habitats. These can include: school grounds, local parks, allotments, wasteground, hedges, walls, gardens, nature reserves, woodland, country parks, farmland, zoos, botanic gardens, quarries, cliffs, coastal areas, ponds, rivers, moorlands and mountains. 8. " Environments that include natural elements such as plants, soil, and water. These may be human made (e.g., gardens, nature playgrounds and urban parks) or wild and naturally occurring (e.g., wooded areas, meadows and beaches)." pg6479 9. " Natural environments in the health care setting are designed to provide children and adults the opportunity to make choices as well as a sense of control and freedom that does not occur inside the hospital. Outdoor spaces offer areas where families can interact away from the health care setting while coping with stresses that may present themselves as a result of hospitalization. " p.28 | 1. Canada, NA 2. UK, 8-9 years 3. Norway, 3-5 years 4. Australia, 2-4 years 5. Multiple, <= 7 years 6. Canada, 4-5 years 7. UK, NA 8. International, children 9. Canada, NA | 1. Alden 2019 2. Barton 2015 3. Bjorgen 2016 4. Christian 2019 5. Christian 2015 6. Coe 2017 7. Council for EOtC 2019 8. Tremblay 2015 9. Turner 2009 |
|  | Natural settings | | 1. "Natural settings offer the diversity, variety, and open-endedness needed to engage and challenge young children, offering the quality play and sensory experiences that support learning and development" pg 31 | 1. USA, 3-6 years | 1. Desrochers 2001 |
| [Back to Table of Contents](#CONTENTTABLE) | | | | | |
|  | | | | | |
| **Approach/Model/Theory** | | | | | |
|  | Affordances | | 1. "The degree to which any environment, built or natural, physically enables play depends on its affordances for play, that is, children’s potential use of the features in the environment they perceive for play." 2. "Reeve (2006) emphasises the element of affordances as one that promotes autonomy. Αffordance, as a concept within education, refers to the properties of an environment, which in relation to the child’s abilities can enhance learning potential. Essentially, affordance refers to the functional utility of an object/environment to a specific person/animal. It is the way that the environment complements the competences and abilities of an organism, in this instance, the child." (potential and realized) Example: "...affordances for play through a well thought out design of play spaces..." pg 2 3. "Kyttä (2006) connect a close relationships between children’s degree of independent mobility licence and actualization of affordances in the environments. Kyttä (2004) refers to potential affordances, which are opportunities inherent in the qualities of the environment (possible affordances of an environment or object), and realised affordances, which are opportunities the children use in the environment. The extent of potential affordances is defined by the individual’s qualities, such as physical skills and bodily proportions, as well as social needs and personals intention. Therefore, the potential affordances can be different for each individual, group, people and situations. Environments involving a positive interrelationships between mobility licence and actualizing affordances also functions as a zone of proximal development, which means that children are presented with a series of graduate zones of challenges slightly above their current levels of functioning. Few mobility licences make it impossible to actualize affordances." pg 2 4. "Features of the environment can enable and invite children to engage in certain types of play behaviours [70]. Affordances are unique for each individual and can be influenced by personal characteristics (e.g., strength, fear) and other features that may inspire or constrain actions (e.g., trees with low branches afford climbing). " pg 6429 5. "Affordances comprise the infinite range of functions that environmental objects may offer and are action-related...As the term suggests, actualised affordances are related to adopting an activity or its absence (e.g. prompted by a perception of danger)." pg.179 6. An ‘affordance’ (Gibson, 1986) is an incentive to action. For example, a big animal in front of us might provoke fear and make us start running, while a chair affords us the possibility to sit down and an apple affords us the possibility to eat. Pg.77; "In indoor school settings, the pupil must often regulate and suppress the tendency to actions given by the affordances. Because they do not have to sit quietly at their desk in outdoor education, there is far less necessity to regulate and suppress the tendency to action that is physiologically initiated by the limbic system." pg.77 7. " [A]n awareness of the environments and their functional significance, or their functional meaning." pg.111 8. "the environment and the objects and features of that environment afford the possibilities for numerous types of actions" pg.478 9. (Affordances for risky play) "Little and Sweller (2014) maintain that “Affordances encompass characteristics of both the environment and the person, and consequently are unique for each individual and correspond with the individual’s body size, strength, skills, and motivation […]” (p. 338).Thus, considering children’s diverse interests and developmental trajectories, both of physical capabilities (Pellegrini et al., 2007; Pellegrini and Smith, 1998) and of risk tolerance (Morrongiello et al., 2010; Sandseter, 2010a), the versatility, complexity, and flexibility of materials, equipment, and environments are central for allowing individual children to test, regulate, and find their optimal level. This will also include to what extent children can shape and manipulate environments and move equipment (Engelen et al., 2013; Heft, 1988; Sandseter, 2009a). To assess how the environment affords such diverse opportunities and meet individual needs, the Zone of Proximal Development (ZPD; Vygotsky, 1978: 84) is applied as an additional lens. The ZPD refers to what a learner can achieve with some guidance or help from an adult or more experienced peer. Therefore, the features of the environment enabling children’s learning must afford a variety of levels challenging individual children" p.260 10. (affordances in the child care outdoor environment) "In the context of outdoor play areas, the notion of affordances can be used to examine features in the environment that promote specific behavioural responses. Heft (1988) identified characteristics of the environment that afford physically active play such as flat, smooth surfaces that afford running and cycling; slopes that afford rolling and sliding; and trees that afford climbing or swinging from. In a more recent study, Sandseter (2009) examined features of the outdoor environment that afforded opportunities for risky play in line with her six categories." p.338-339 11. "Affordance then is a possibility for action by an individual. The individual needs to be able to recognise the potential and have the skills and motivation to actualise the perceived use of the environment" p.515 12. "Another theoretical framework that could help to explain the reported positive effects of natural environments is Gibson’s concept of affordances (Heft 1988). This framework seeks to account for the functional significance or meaning of environmental features for individuals. Rather than describing environments solely in terms of their physical features, affordances theory aims to account for the potential experiences or opportunities for action ‘afforded’ by environmental features. These affordances will vary according to the characteristics of the individuals who interact with the environment. The concept of affordances can thus account for the different forms of physical activity and experience provided by natural features such as bushes, trees, and uneven topography. Trees, for example, can provide opportunities to climb, to look down from or up into, to hide, to look through leaves, to experience dappled light, etc. Uneven topography can provide opportunities for the mastery of specific motor skills that would not be provided by a smooth and even surface." p.283 13. "Gibson (1979) saw people’s actions mainly as their interpretation of what the physical environment has to offer, or the affordance of the environment. The affordance also depends on the ability of the person involved, so the affordance of the environment does not need to be the same for an infant, a school child or an adult." p.3 14. (affordances of the environment) "the study draws on Gibson’s (1979) theory of the affordance of the environment. Gibson saw people’s interpretation of what the physical environment has to offer for their actions as the affordance of the environment. The affordance also has to do with the capability of the people involved, and it may not be the same for all. Thus the affordance of the environment describes the interaction between people and the environment." p.3 15. "Even though children naturally seek to engage in risky play, features of the play environment influence children’s play by affording certain types of play activities. Gibson’s (1979) theory of affordances states that the physical environment that we live in affords different actions and behaviors. The affordances of the environment include what it ‘‘invites’’ us to do, and the concept of affordances includes both the environment and the person, meaning that the affordances are unique for each individual and correspond with the individual’s body size, strength, skills, courage, fear, etc. Heft (1988) elaborated Gibson’s theory and argued that children’s outdoor environment affords different types of play and that children perceive the functions of the environment as invitations for certain activities" p.439 16. "Central to Gibsonian theory is the idea of affordances, which are features of the environment that allow an individual to perform particular actions, but the individual needs to engage in exploration to learn about the many affordances in their environments (E.J. Gibson, 1988)." p.8 17. (affordances in the preschool outdoor environment) “… refer to what this environment provides and what is perceived or recognized by children as realizable in relation to their needs, interests, motivation, or capabilities (Kyttä 2002). T Thus, the same preschool outdoor environment may offer each child different possibilities, due to their subjective perception of it (Waters 2017). For example, one child may perceive an element of the outdoor environment as climbable, while another sees it as something for sliding on.” | 1. Canada, NA 2. Denmark, NA 3. Norway, 3-5 years 4. Multiple, NA 5. New Zealand, NA 6. Norway, 5^th^ grade 7. Norway, 5-7 years 8. Canada, NA 9. Norway, 1-3 years 10. Australia, early childhood education centres 11. New Zealand, 12-60 months 12. Australia, 3-4 years 13. Iceland, 4-9 years 14. Iceland, 3-13 years 15. Norway, 4-5 years 16. Norway & Australia, children 17. Spain, 3-6 years | 1. Alden 2019 2. Barrable 2019 3. Bjorgen 2016 4. Brussoni 2015 5. Ergler 2013 6. Fiskum 2013 7. Fjortoft 2011 8. Herrington 2015 9. Kleppe 2018 10. Little and Sweller 2015 11. Mawson 2014 12. Nedovic 2013 13. Nordahl 2015 14. Nordahl 2016 15. Sandseter 2009 16. Sandseter 2012 17. Larrea 2019 |
|  | Actualized affordances | | 1. "...the way the features get used in practice as the actualized affordances which often depend on adult permission." | 1. Canada, children | 1. Alden 2019 |
|  | Embodiment | | 1. "We use Gibb’s (2003) description of embodiment as people’s subjective and felt experiences of their bodies in action and the role of this interaction on meaning making to better understand sensory experience." pg 16   "They highlight that such an approach recognizes how the senses, emotion, and cognition are all intertwined and emphasize that the ‘…essence of lived experiences occurs through the body, where intrinsic and subjective qualities of experience provide us with opportunities for insight and understanding." pg 16 | 1. Sweden (adults) & Norway (1-6 years) | 1. Beery 2018 |
|  | Environmental affordances | | 1. "Affordances are functional features of the environment characterized by particular physical attributes (e.g., water puddles on a muddy trail) and the unique response of the individuals who encounter them (e.g., one preschooler may take the opportunity to splash in each puddle while another child hops over them). The novelty of environmental affordances, such as trees for climbing and creeks for splashing, is enhanced by the changing seasonal and climactic conditions encountered by children who have sustained, engaging experiences in natural outdoor spaces." p.37-38 2. "Importantly, the functional features of an environment are determined by attributes of the environment itself (e.g. large rocks afford opportunities for climbing, hiding, etc.) and by the attributes and behaviors of the individual (a child may choose to run or skip along a path, whereas an older adult may prefer to walk more slowly). Thus, the affordances of a given environment are what it ‘invites’ an individual to do, and are uniquely dependent upon a person’s size, strengths, skills, fears and so forth (Gibson, 1979; Heft, 1988; Sandseter, 2009)." p.32 | 1. USA, 33-59 months 2. USA, 33-59 months | 1. McClain 2016 2. McClain 2016 (J Adventure Educ Outdoor Learning) |
|  | Integrative model | | 1. “Morrongiello and Lasenby-Lessard’s (2007) integrative model identifies multiple factors at child, parent/family and socialsituational levels as well as macro level factors including neighbourhood, socioeconomic status and culture that potentially influence children’s risk decisions. The model opens up the potential for a range of small and larger scale studies to examine the various factors associated with children’s risk-taking behaviour. Whilst this present study had focused on individual factors, when considered with previous studies conducted with predominantly school-aged children, it furthers our understanding of the development of children’s risk perception and appraisal in younger children.” | 1. Australia, 4-5 years | 1. Little 2010 |
|  | Social affordances | | 1. "Social affordances are a subcategory of affordances, namely possibilities for social interaction offered by the environment. Clark and Uzzell (2006) utilize the term environmental affordances to have an equally strong social dimension; the affordances may be direct (from physical environment) or indirect (mediated by the social presence or behaviour of others). Charles (2012) argues that social affordance refers to opportunities in the environment that promote social relationships and interaction." pg 3   Examples: "social affordances in character of invitations, imitations, responses, scaffolding and joint attention". pg 8 | 1. Norway, 3-5 years | 1. Bjorgen 2016 |
|  | Attention restoration theory | | 1. "Attention Restoration Theory proposes that nature assists with recovery from attention fatigue, allowing distance from routine activities and thoughts to engage without conscious effort." pg 2 2. "Following Attention Restoration Theory (ART, e.g., Kaplan, 1995) in opposition to screen watching (e.g., television), unthreatening greenish outdoor environments typically accessible to both urban and country dwellers stimulate by so-called soft fascination (Kaplan and Berman, 2010). Please note that threatening greenish outdoor environments may have more intrusive, yet desirable cognitive effects (e.g., Kahn et al., 2009)." p.773-774 | 1. UK, 10-14 years 2. Multiple (review), school children | 1. Chiumento 2018 2. Schilhab 2018 |
|  | Complex systems | | 1. "...an essential focus for science education, because they contain important ideas in national standards, and provide an integrating context across a number of science domains." pg. 540 "...dynamic, self-organizing, and continually adapting" pg. 540 | 1. Israel, 4^th^ grade | 1. Assaraf 2010 |
|  | Ecology | | 1. "Ecology describes the environment as the sum of all external conditions that surround a system, an organisation, a community or an object. The environment is divided into ‘natural’ and ‘manmade’. The natural environment includes natural ecosystems and the manmade environment includes anthropogenic systems created today or in the past" pg. 417 | 1. UK, NA | 1. Stavrianos 2016 |
|  | Ecological psychology | | 1. "In the field known as Ecological Psychology, we seek to highlight the importance of the environment during the process of cognitive development in children, given that in addition to exposing the diوٴerent ecological dimensions of this context, Ecological Psychology updates the active character of the child's participation in their environment and consequently in their development. Нe peculiarity of the child and their way of learning is no longer seen as immaturity or a distortion of reality but reveals the inventive process that guides cognition [8]." p.2 | 1. NA, 8-10 years | 1. Profice 2016 |
|  | Expectancy value model | | 1. "Eccles and Wigfield’s (2002) expectancy value model of motivation is also helpful for considering parental influence on children, beyond parents’ direct “gatekeeping” influence. This model suggested social interactions within cultural contexts influence not only how children directly experience the world, but also how they integrate the values they are developing into their identity. Thus, parents become an important influencing factor on the extent to which children value nature experiences and identify with nature." pg 31 | 1. USA, 3-6 years | 1. Desrochers 2001 |
|  | Joint attention theory | | 1. "Reed’s (1996) joint attention theory suggested young children first attend to features in the world around them that others are noticing; building from these experiences, children begin to control their own attention by pointing to or asking questions about the world they encounter. Through this process, “children learn what people around them consider worth noticing and how they appraise it, and they find their own spontaneous interests either encouraged, reprimanded, or ignored”. Consequently, they come to view nature as “a place of fascination that a family explores and appreciates together, a scary place that children are forbidden to enter, or something barely noticed as children ride by in the car”..." pg 31 | 1. USA, 3-6 years | 1. Desrochers 2001 |
|  | Ludocentric approach | | 1. "...meaning [adults] support children’s play without being either directive or negligent." | 1. Canada, NA | 1. Alden 2019 |
|  | Place-based learning model | | 1. “The place-based learning model proposed by Sanger (1997) is a three-pronged model similar to the TBTE experience with the aim of: building connections; building community; and using narrative. ‘Building connections’ is an experiential, interdisciplinary approach to learning that develops students’ skills, confidence and understanding of the value of their place. ‘Building community’ entails using cooperative learning strategies to involve all members of a place in the process of education. ‘Using narratives’ means listening to and learning from stories of the community members and the land and the ways in which they are intertwined.” | 1. NA, NA | 1. Gray 2015 |
|  | Positive youth development | | 1. "There have been many different approaches to addressing these risk factors through multisystemic interventions; however, a cohesive, research-based framework is needed for program development and evaluation in service delivery to youth exposed to multiple risk factors. The positive youth development (PYD) approach attempts to create this type of framework by providing youth with positive, asset-building experiences and meaningful, supportive relationships to develop resilience and coping skills in the face of risk factors" p.336 | 1. USA, 13-18 years | 1. Norton 2014 |
|  | The S.A.F.E. Model for Playgrounds | | 1. S.A.F.E. Model - Hudson and Thompson (2004) identified four interactive risk factors regarding injuries to children on playgrounds: Supervision, Age-appropriate design of equipment, Fall surfacing, and Equipment and surfacing maintenance. These four elements interact with one another to create a safe play environment: the S.A.F.E. Model (Figure 1) | 1. USA <= 14 years | 1. Olsen 2008 |
|  | Self-determination theory | | 1. "SDT describes the motivation for human behaviour as being on a continuum between extrinsic (outer) and intrinsic (inner) motivation, with transitional phases in between. It is based on the assumption of ‘. . . psychological needs—namely, the innate needs for competence, autonomy, and relatedness’..." 2. "The main tenet of the theory is that individuals should be supported in ways that facilitate the expression of their intrinsic tendencies rather than be controlled externally through strict rules, rewards or punishments." pg 40   "SDT is a psychological theory that illuminates the conditions and processes through which growth is optimized." pg 40   1. Used to examine student's learning motivation during an " short-term curriculum-based residential outdoor learning course" pg. 2   "In the concept of SDT, one’s learning motivational behavior is defined as a self-regulated action. To differentiate between specific loci of causality as a basis of motivational behavior, Deci and Ryan (2000) defined a continuous scale from intrinsic motivation, through extrinsic motivation, to amotivation." pg 2 | 1. Denmark, NA 2. Denmark, NA 3. Germany, 12-14 years | 1. Barfod 2018 2. Barrable 2019 3. Dettweilder 2017 |
|  | Stress reduction theory | | 1. " Stress Reduction Theory suggests that particular environments produce certain effects, with perceived “safe” environments triggering positive emotional responses." pg 2 | 1. UK, 10-14 years | 1. Chiumento 2018 |
| [Back to Table of Contents](#CONTENTTABLE) | | | | | |
|  | | | |  |  |
| **Outcome/Component** | | | |  |  |
|  | Active play opportunity, teacher-led | | 1. " teacher-initiated activities, such as running laps or active games, in which all children were expected to participate" p. 12 | 1. USA, 3-5 years | 1. Tandon 2015 |
|  | Adaptation | | 1. '...may include not only physical modifications of the environment, but also techniques used by teachers to interact and communicate with children during outdoor activities.' pg. 21 | 1. USA, NA | 1. Flynn 2002 |
|  | Adult supervision/monitoring | | 1. "Morrongiello, Corbett and Kane, distinguish between “monitoring” and “supervision” to illustrate this change, defining monitoring as a general awareness of child’s activities, as compared to supervision being a more active watching and listening (note that this distinction is not made in the clinical and developmental psychology literature." pg 6427 | 1. Multiple (review), children | 1. Brussoni 2015 |
|  | Adult support | | 1. "...role of the adult as the gatekeeper with decision-making power to allow or deny play and to protect time for play" | 1. Canada, NA | 1. Alden 2019 |
|  | Agency | | 1. "...freedom and independence to play; ability to make choices about play; and, that play is self-directed or child-led".   "the freedom to choose how long they play, with whom, how play occurs, and in a manner that gives them the agency in that experience" | 1. Canada, children | 1. Alden 2019 |
|  | Autonomy | | 1. "...the less controlled and unpredictable nature of the outdoor environment instigated the development of children’s autonomy. Outside, children did not depend entirely on the adult to solve problems, leaning on peers’ help and individual skills to overcome obstacles. As Waite, Rogers, and Evans (2013) refer, it is important to allow children space to try out different ways of being, without neglecting the need to guarantee emotional balance and healthy patterns of interaction." pg 295 | 1. Portugal, 4 months-10 years | 1. Bento, 2018 |
|  | Affirmity towards nature | | 1. "Kals, Schumacher and Montada (1999) use the related term affinity toward nature to describe the emotional bonds and cognitive interest in nature. " pg 31-32 | 1. USA, 3-6 years | 1. Desrochers 2001 |
|  | Biodiversity understanding | | 1. Achieved through "childhood interaction with variation and diversity with living and nonliving items from nature". pg 13   "...the variability among living organisms from all sources including, inter alia, terrestrial, marine and other aquatic ecosystems and the ecological complexes of which they are part; this includes diversity within species, between species and of ecosystems’ (UN 1992, 3). This general definition serves as a base from which to consider necessary interaction to support the understanding of the concept, including experience of species variation, variations between species, and the variety of ecosystems supporting biodiversity." pg 15  "... incidental learning that occurs through the encounter with natural variation, i.e. large numbers of different insects, plants, and animals during foraging." pg 15 | 1. Sweden (adults) & Norway (1-6 years) | 1. Beery 2018 |
|  | Biological knowledge | | 1. "The idea is that everyday contact with plants and animals in “relatively intact ecosystems” (Coley, 2012, p. 994) provides rich information from which children acquire facts, notice patterns, and make inferences about the natural world." p. 1147 | 1. Multiple (review), NA | 1. Longbottom 2016 |
|  | Biophilia | | 1. "...the urge to affiliate with other forms of life." 2. "According to the biophilia hypothesis humans have an innate affinity for every living thing resulting from evolutionary heritage of the human species." pg 937 3. "...an innate, programmed positive response to intertwining nature as experienced by all human beings. His definition is, simply, ‘the urge to affiliate with other forms of life’." pg 1034 | 1. USA, NA 2. Singapore, 7-18 years 3. Multiple, NA | 1. Bentley 2010 2. Braun 2017 3. Brown 2017 |
|  | Ciclovias | | 1. "Ciclovías, typically occurring on Sundays or holidays, are closures of large sections of a main city street to promote active transport (eg, walking, cycling). Some Ciclovías have started to incorporate group fitness classes and active play opportunities as activity areas." pg 2 | 1. UK & USA, NA | 1. Bridges 2019 |
|  | Collaboration in play | | 1. "Refers to processes in which shared activities, creation, enjoyment and knowledge construction are present." pp. 392 (Bodrova 2003; Hyoven 2005, Kieff 2000) | 1. Finland, teachers (25-53 years) & children (Kinder to 6^th^ Grade) | 1. Hyvonen 2008 |
|  | Connectedness to nature/nature connectedness | | 1. "Connection to nature is the degree to which “an individual includes nature as part of their identity”." pg 60   "...assessed using the State Connectedness to Nature Scale... A higher score represents a greater connection to nature." pg 63   1. "...nature connectedness as a scientific construct is likewise applied as inclusion of nature in self, emotional affinity towards nature, nature relatedness or connectivity with nature. While many authors underline the emotional base when defining the concept of nature connectedness another approach focuses on the role of the natural environment in a person`s identity. This study relies on the definition of nature connectedness as the degree to which individuals rationally perceive nature as part of their own identity. Determines three components which constitute the construct of nature connectedness. The cognitive component manifests itself as the individual feeling of being integrated with nature. The affective component is expressed through the sensitivity for nature protection. " pg 938 2. "often understood as ‘one’s subjective sense of connection with the natural world’ (Capaldi, Passmore, Nisbet, Zelenski, & Dopko, 2015, p. 2)." p.683; "We used a 13-item version of the Connectedness to Nature Scale (CNS), which measures individuals’ ‘levels of feeling emotionally connected to the natural world’ (Mayer & Frantz, 2004, p. 503). Its unidimensional structure has been confirmed with other Brazilian samples (Pessoa, Gouveia, Soares, Vilar, & Freires, 2016)." p.685 | 1. UK, 11-18 years 2. Singapore, 7-18 years 3. NA, Mean 23.6 years | 1. Barton 2016 2. Braun 2017 3. Rosa 2019 |
|  | Contemporary apprenticeship | | 1. "a teaching/learning model named contemporary apprenticeship. According to Gordon and von B€ulow (2012), this model of apprenticeship is based on social constructionism (learning by making) (Papert and Harel, 1991) and social constructivism (learning as a social process) (Kim, 2001)." p.1109 | 1. UK, college students | 1. Mata 2016 |
|  | Docile bodies | | 1. "The three signs of power creating ‘docile bodies’ (p. 151) described by Foucault (1975) are (a) hierarchical observation; (b) normalizing judgments; and (c) the examination. Children in the Giddings and Yarwood study (2005) reported great effort in resisting hierarchical observation, situations in which constant adult observation leads to docility. Their rationale for this resistance was the judgment that adults had placed upon children themselves in the past, and in particular children’s play. This judgment is what Foucault refers to as normalizing judgments, the action of adults prescribing acceptable behaviour. Foucault’s third category, the examination, is defined as a definitive occasion that combines observation and judgment, often accompanied by documentation of that judgment. By resisting the chance to be observed at Frog-hole, children not only resisted the opportunity to be judged, but also to be examined." pg 1031 | 1. Multiple, NA | 1. Brown 2017 |
|  | Ecophobia | | 1. "fear of the natural world" p.124 | 1. USA, children | 1. O'Shaughnessy 2013 |
|  | Environmental attitude | | 1. " The construct of environmental attitudes is based on the psychological tendency expressed by the individual evaluation of the natural environment resulting in an inclination towards preservation (conservation and protection of the environment) or utilization (feeling of dominance over the environment). Schultz, Shriver, Tabanico and Khazian (2004, 31) call it a ‘collection of beliefs, affect, and behavioral intentions a person holds regarding environmentally related activities or issues’. Personal appreciation of the natural environment is deemed to be shaped by both the individual’s relationship to nature as well as by cultural conditioning." pg 901 | 1. Bangladesh, Singapore, Malaysia, Germany, 7-18 years | 1. Braun 2018 |
|  | Environmental behaviour | | 1. "Environmental behavior refers to modes of behavior which aim at minimizing negative human impacts on the natural and built environment." pg 901 | 1. Bangladesh, Singapore, Malaysia, Germany, 7-18 years | 1. Braun 2018 |
|  | Environmental generational amnesia | | 1. "...a generalized acceptance of degraded environmental conditions as the non degraded norm." pg 14 | 1. Sweden (adults) & Norway (1-6 years) | 1. Beery 2018 |
|  | Environmental identity | | 1. "environmental identity is used by Clayton and Opotow (2003) to describe “a sense of connection to some part of the non-human natural environment… a belief that the environment is important to us and an important part of who we are” pg 31-32 2. " as an indicator of relatedness or connectedness to nature" pg. 229 | 1. USA 3-6 years 2. UK (England), 12-15 years | 1. Desrochers 2001 2. Hinds 2011 |
|  | Environmental knowledge | | 1. "...environmental knowledge is exclusive to each educational program there is no standardized comparable measuring instrument." pg 904 | 1. Bangladesh, Singapore, Malaysia,   Germany, 7-18 years | 1. Braun 2018 |
|  | Environmental modification | | 1. "the addition of portable play equipment (for use indoors and outdoors) that has been shown to predict physical activity levels among preschoolers. The package will include items such as: balls, hula hoop activity pack, obstacle course, stepping domes, ribbons, a hopscotch play mat, and hop-along bouncers. " pg. 4 | 1. Canada, 2.5-5 years | 1. Tucker 2016 |
|  | Flourish | | 1. "...develop into thriving, vital, fully-functioning adults…" pg 39 | 1. 1. Denmark, NA | 1. 1. Barrable 2019 |
|  | Flow | | 1. "A highly involved child is in a state of flow. Flow is characterised by the matching of high environmental challenges with equally high levels of personal skills, the merging of action and awareness, the loss of reflexive self-consciousness, a sense of control and a distortion of temporal perception." pg 307 | 1. Norway, 3-5 years | 1. Bjorgen 2015 |
|  | Harm | | 1. "Physical or mental damage or injury: something that causes someone or something to be hurt, broken, made less valuable or successful" p.6479 | 1. International, children | 1. Tremblay 2015 |
|  | Hazards/danger | | 1. "A source of harm that is not obvious to the child, such that the potential for injury is hidden " pg6479   "The potential for injury can be immediate or long term. A source of danger" pg6479 | 1. International, children | 1. Tremblay 2015 |
|  | Holistic child development | | 1. "Integrating early childhood education, environmental education, and social-emotional supports within and throughout the entirety of an early childhood program helps to enhance holistic child development." pg 21 | 1. NA, early years | 1. Carter 2016 |
|  | Hyper parenting | | 1. “‘Hyper-parenting,’ ‘invasive parenting,’ or ‘intensive parenting,’ in which a climate of ‘inflated risk’ leads parents to micromanage all aspects of their children’s lives in an effort to protect the child from adverse experiences” pg6479; “Parents attempt to become experts on optimal parenting strategies, and child health and development so as to ensure that their children achieve their full potential” pg6479 ; “A variety of different types of highly involved parents (from ‘‘helicopter parents’’ to ‘‘tiger moms’’)” pg6479 | 1. International, children | 1. Tremblay 2015 |
|  | Inclusion | | 1. "Inclusion can be defined in many ways. Generally, inclusion means to be a natural part of, for instance, a group, class or community, accessibility to activities, participation and equal opportunities. Depending on the objective of the activity, it is reasonable to believe that the educator can support inclusion by collaborative work, experimental tasks and problem-based learning." pg 100 | 1. Sweden, Finland, Latvia, Germany , Greece, 0-25 years | 1. Brodin 2009 |
|  | Independent mobility | | 1. "...children playing freely in neighbourhood streets, parks, and playgrounds." 2. "Children’s independent mobility, which refers to their freedom to travel around their neighbourhood by themselves without adult supervision, is one example of outdoor risky play and may be important for facilitating other opportunities for risky play." pg 2 3. "children's independent mobility, that is walking or cycling without adult supervision" pg 260   "To determine children's independent mobility behavior, an Independent Mobility Index was computed using questions from both parent and child questionnaires. This index has been described elsewhere. Briefly, children were asked if they actively traveled to 15 local activities/destinations (excluding trips to school) in the week prior to the survey (no, sometimes, yes). The activities (n = 6) included playing a team sport; swimming; going to a club or youth group; watching sport; music lessons; and catching a bus. The destinations (n = 9) included visiting a park, playground or playing field; own friend's house; family/family friend's house; local shop; other shops; post-box; local library (not school library); movie cinema; and Sunday school/church. Parents were also asked if they allowed their child to attend these 15 activities/destinations without an adult (no, yes). An independent mobility score was computed by summing the activities/destinations children actively traveled to and if they were allowed to do so without an adult (range 0–15)." g 260   1. "Independent mobility: defined as the extent to which the child is allowed by his/her parents to walk or bike alone in the neighborhood environment [37]. Independent mobility was assessed by one dichotomous variable based on the respondents0 agreement with the following statement: “I am regularly allowed to walk alone during daytime” (0 = disagree, 1 = agree)" p.6 | 1. Canada, NA 2. Canada, 6-12 years 3. Australia, 10-12 years 4. Israel 10-12 years | 1. Alden 2019 2. Brussoni 2015 3. Christian 2014 4. Moran 2017 |
|  | Intensive parenting | | 1. "Western middle-class social pressures to maximize children’s opportunities and adhere to practices of “intensive parenting” support the notion that parents should have children attend the “best” schools, participate in a multitude of organized activities, and provide as much protection as possible—potentially more than they personally perceive as necessary. The result has been creation of a “backseat generation” with little unstructured play time and reliance on automobile-based commuting from one activity to the next.” Pg 3138 | 1. Multiple, NA | 1. Brussoni 2012 |
|  | Inter subjectivity | | 1. "...social sharing of physically enjoyable experiences..." pg 318 | 1. Norway, 3-5 years | 1. Bjorgen 2015 |
|  | Interaction with nature | | 1. "Investigating nature, sensory explorations, passive observing of nature, critical thinking and discussion, experimenting" pg 378, Table 1 | 1. Canada, 4-5 years | 1. Coe 2017 |
|  | Involvement | | 1. "A motivating climate can promote involvement in and enjoyment of physically active play. Laevers (1993, 1996, 2005a) developed the concept of “involvement” and concluded that involvement is a good indicator of the occurrence of developmental processes in the child. Involvement is a quality of human activity, characterised by concentration and persistence, a high level of motivation, intense perceptions and experiencing of meaning, a strong flow of energy and a high degree of satisfaction, and based on the exploratory drive and basic developmental schemes." pg 307 | 1. 1. Norway, 3-5 years | 1. 1. Bjorgen 2015 |
|  | Joint and several liability reform | | 1. “Joint and Several Liability is a legal principle that permits the injured party in a tort action to recover the entire amount of compensation due for injuries from any tortfeasor who is able to pay, regardless of the degree of that party’s negligence Entities that are often viewed as those with the greatest amount of liability insurance are seeking reform to this principle so that the amount they pay towards an injured party directly correlates with the degree to which they were negligent. “ pg 6479 | 1. International, children | 1. Tremblay 2015 |
|  | Modelling | | 1. Examples: " children are modelling cues from parents, where they see or hear parents appraising outdoor settings in terms of how safe they appear to be." pg 41   "Parents who preferred forests had children with significantly stronger levels of affinity toward nature than parents who preferred the other setting types of water, fields, and parks." pg 41 | 1. USA, 3-6 years | 1. Desrochers 2001 |
| 37. | Naturalist intelligence | | 1. "This intelligence has to do with observing, understanding, and organizing patterns in the natural environment. Gardner described the naturalist as the person who "is able to recognize flora and fauna, to make other consequential distinctions in the natural world, and to use this ability productively"." pg 36 | 1. 1. NA, Middle school students | 1. 1. Broda 2002 |
| 38. | Nature-deficit disorder | | 1. "...the increasing divide between youngsters and the natural world." 2. "Louv writes about the lifestyles of today’s American youth and about how little the experience of nature is part of their lives. He documents what most of us already have observed, that kids nowadays are far more focused on their screens than on the out-of-doors. The consequence of this situation, he argues, is the declining health of our population as well as other growing societal ills." pg 7-8 3. "lack of nature-connectedness and environmental identity because children are denied uninhibited, unstructured tree-time play in natural or wild environments." Pg.21 4. "Louv (2008) coined the phrase “nature deficit disorder” to describe how human beings, especially children, are spending less time outdoors. The nature deficit disorder is termed not as a technical diagnosis, but rather as an association between a lack of time spent in the outdoors and a wide range of developmental and behavioral issues, and loss of opportunity to build connections with nature. Driessnack (2009) further extended the notion of a nature deficit disorder, suggesting that children are losing the ability to relate to others’ life experiences, because they are unable to experience their own world directly" p.324 | 1. USA, K-8 children 2. USA, NA 3. Australia, Grade 6-7 4. Multiple (review), NA | 1. Ashmann 2018 2. Bentley 2010 3. Francis 2013 4. Ray 2018 |
| 39. | Nature relatedness | | 1. "Outdoor learning in natural environments, such as FS, can support the building of a relationship between the individual and nature, in ways that are not as accessible in traditional educational settings. This development of Nature Relatedness (NR) is a positive and desired outcome for all. Not only is it linked with general psychological well-being, as well as subjective well-being, but it also relates to environmental concern and pro-environmental behaviors. NR is also associated with a greater sense of belonging within a social context, creating a virtuous cycle between social connections and nature connections." pg 48 | 1. 1. Denmark, NA | 1. Barrable 2019 |
| 40. | Nature understanding | | 1. Example: "...understanding about the Earth’s cycles – how plants grow, how weather/climate affect our lives, how plants/animals/humans interact, and how the living and non-living worlds are interdependent." pg 16 | 1. Sweden (adults) & Norway (1-6 years) | 1. Beery 2018 |
| 41. | Nurturing | | 1. "Nurturing may be defined as responding to the feelings of the child—good, bad or indifferent—and a concentration in a focus of care on the individual (General Teaching Council Scotland, 2012)." p.239 | 1. UK, 4-5 years | 1. McArdle 2013 |
| 42. | Outdoor play arrangement | | 1. "There needs to be a balance between quiet and active areas, a balance between shady and sunny areas, and a balance of levels. Having visible pathways also promotes children's safety because they direct children's movements through the yard. Playgrounds and equipment can be affected by different kinds of weather (e.g., constant dampness, hot sun, dryness, and freezing), and these conditions can cause playground hazards." | 1. NA, NA | 1. Taylor 1996 |
| 43. | Park poor | | 1. "Park poor has been defined as “three acres or less of parks per thousand residents” (Garcia, Rawson, Yellott, & Zaldana, 2009)." p.138 | 1. USA, 11-20 years | 1. Mackenzie 2017 |
| 44. | Personal and social development | | 1. "PSD is often understood as developing confidence, cooperation, trust and teamwork." pg 223   "Moral development" and "self-esteme" are also noted as central to PSD. | 1. UK, NA | 1. Allison 2010 |
| 45. | Personal ecology | | 1. “The concept of personal ecology defined by Holt (1989) as "the individual child interrelating, interweaving, and interacting with the phenomena that make up his own environment" (p. 118)” | 1. NA, children | 1. Bullock 1994 |
| 46. | Place attachment | | 1. "Place attachment describes how a person associates a place with particular social bonds and activities that can also be based in natural landscapes. Attachment to place can be strengthened through social interactions, active engagement and frequency of visits." pg 297   "Place attachment in the school setting appeared to be attributed to imaginative play encouraged through new interactions with spaces and the physical world of the school grounds." pg 307 | 1. Australia, 10-12 years | 1. Cumming 2015 |
| 47. | Place meaning | | 1. "Kudryavtsev et al. (2012) define place meaning as symbolic meaning attributed towards a place that is strengthened through direct experience and learnt through interpretive materials and from others. Place meaning can help to explain why a person may be drawn to a particular place and can shape place identity, both of which serve to promote a sense of belonging." pg 297 | 1. Australia, 10-12 years | 1. Cumming 2015 |
| 48. | Play facilitation | | 1. "...hands-off, minimal adult supervision of play...intentional, supportive role for adults (albeit with diverse permutations)..."   "...provide greater social-emotional support for children on school playgrounds, and a shift from outdoor supervision to intentional practice in early years pedagogy." | 1. Canada, NA | 1. Alden 2019 |
| 49. | Play policy | | 1. "a play policy is a statement of both an organization’s current play provision and its aspirations for change and development. Play policy makes explicit the following important criteria:   -the objectives of play and play-related services and activities,  -the connection between acceptable levels of risk and healthy play,  -an assumption of inclusive play settings for all children,  - the criteria for a quality play environment,  -the essential aspect of play as part of a child’s cultural life, and  - need to create play opportunities in the general environment." pg 6 | 1. UK, NA | 1. Stegelin 2005 |
| 50. | Play value | | 1. "Play value of the enviroment refers to how well a playground provides for children's (1) physical challenges - gross and fine motor skills; (2) intellectual challenges - spatial exploration on several scales, use of imagination, opportunities to manipulate the environment to experience cause and effect; (3) emotional challenges - feelings of mastery and success; (4) social challenges - opportunities for cooperation and interactions." pp. 20 | 1. NA, NA | 1. Hudson 2001 |
| 51. | Playground design | | 1. Approaches to playground design include: Landscape architect, collaboration with community, collaboration with teachers, collaboration with children, decisions made by principals, school boards. pg 47 | 1. Australia/Turkey, primary school students | 1. Chancellor 2014 |
| 52. | playground duty | | 1. (police)"...maintaining a high level of vigilance to prevent accidents and aggressive behaviour. Interactions with children are limited to solving problems and enforcing playground rules, and are most often negative or directive." pg 39 2. (participate) "In these schools teachers work as a team to provide equipment, teach skills and play with children, while still providing adequate supervision and giving attention to problem-solving. Teachers’ interactions with the students are restorative, positive and respectful." pg 39 | 1. New Zealand, NA 2. New Zealand, NA | 1. Couper 2011 2. Couper 2011 |
| 53. | Playground guidelines | | 1. "The guidelines specifically call for nature features such as trees, stumps, boulders, long grass water and pebbles; and explicitly call for inclusion of risky play elements." pg 1038 | 1. Multiple, NA | 1. Brown 2017 |
| 54. | Playground management | | 1. "The design and management of the playgrounds generally determine what children do outside, despite the fact that children can and do respond to restrictive rules by trying forbidden behaviours. A strong focus on preventing injury is not always balanced against the benefits provided by child-directed play." pg 54 | 1. Australia/Turkey, primary school students | 1. Chancellor 2014 |
| 55. | Playground safety standards | | 1. "Playground safety standards exist in many nations and influence playground design... Standards can have an important role in ensuring the reduction of hazards on playgrounds that result in serious injuries." pg 4626 | 1. Multiple, NA | 1. Brussoni 2015 |
| 56. | Power of nature | | 1. “becoming reconnected to complex, multiple perceptions, and lived realities of the environment [and] reviving a state of being within one’s own body, being fully within relationships, ecosystems and systems of spirituality” (Neilson, 2008, p. 137). " p. 135 | 1. Portugal, NA | 1. Neilson 2009 |
| 57. | Protectionism | | 1. "...cultural feature of ‘protectionism’ which places children in a position of helplessness rather than strength, was apparent in the teachers’ stated need to see the children at all times. This was commonly quoted by the teachers as a reason for not making more use of their outdoor environments although they also informally referred to their concerns about a growing culture among the parent body of blame and litigation. In other words, they were concerned about risk not only to children, but also to themselves." pg 1036 | 1. Multiple, NA | 1. Brown 2017 |
| 58. | Public entities | | 1. "Municipal governments, regional governments, local economic development legal entities or authorities, sectorial representative organizations." pg6479 | 1. International, children | 1. Tremblay 2015 |
| 59. | Re-engagement of place | | 1. "Beames and Ross (2010) refer to a ‘re-engagement of place’ (bringing back connections and meanings to place) as an outcome of learning outside, learning in the context of the student’s surroundings and of their own perspectives." pg 296 | 1. Australia, 10-12 years | 1. Cumming 2015 |
| 60. | Risk | | 1. A key element of outdoor education. pg 185 2. "Risk has been described as a challenging and adventurous physical activity and a thrilling and exciting form of play. These voluntary experiences involve the weighing of benefits against undesirable consequences as children push their own mental boundaries and physical limitations, often to attempt something they have never done before. " pg 367 3. “Defining risk and risk-taking at the centre of the risk dialogue are issues related to the way in which risk is defined. The term risk often has negative connotations. Definitions of risk often emphasise loss or adverse outcomes (Aven & Renn, 2009). Within the developmental psychology literature, risk-taking is usually defined as engagement in behaviours that are associated with some probability of negative outcomes (Boyer, 2006). The problem with the focus on the negative outcomes of risk in these definitions is that they fail to acknowledge that risktaking actually encompasses ‘an endless spectrum of behaviours and activities’ (Madge & Barker, 2007, p. 8) that are socially constructed, varying from one context to another and both within and across cultures. So risk is perhaps better defined as situations in which the outcome is uncertain (Aven & Renn, 2009).” P.25 | 1. Singapore, NR 2. Canada, 4-5 years 3. Australia, 4-5 years | 1. Atencio 2015 2. Coe 2017 3. Little 2015 |
| 61. | Risk appraisal | | 1. “Risk appraisal involves a subjective assessment of the probability of experiencing negative consequences in situations where there is some uncertainty regarding the outcomes (Rosenbloom 2003). The higher one perceives this probability to be the more likely one will appraise the situation as involving potential risk or harm (Hyson and Bollin 1990). | 4. Australia, 4-5 years | 4. Little 2010 |
| 62. | Risk aversion | | 1. "Fear of litigation results in minimising “risk” at all cost and decreasing the value of “real play” that occurs on school playgrounds" pg 2 | 1. Australia, 5-7 years | 1. Bundy 2011 |
| 63. | Risk avoidance | | 1. "In the United States, the area in which children were allowed to roam by their parents freely from their house shrank by 90% between 1970." pg 1034   "Sandseter (2009) reports on the mediating role of adults’ beliefs about risk that lead to the provision or denial of risky opportunities for children. ‘Handling risk and children’s risk-taking in play is one of the challenges that ECED practitioners face during their daily life’ (p. 96). " pg 103 | 1. Multiple, NA | 1. Brown 2017 |
| 64. | Risk-benefit assessment | | 1. "...the Play Safety Forum developed a practical tool for risk-benefit assessment of children’s play spaces, which considers the benefits of risky play and the reasonableness of safety measures. This tool identifies hazards (potential sources of harm), the risk from that hazard (likelihood and severity of harm), and helps determine the need for modification or removal of the hazard. Some hazards are viewed as acceptable because they offer developmental benefit to children (e.g., changes in height; loose materials such as sticks)." pg 6446-6447 | 1. International, children | 1. Brussoni 2015 |
| 65. | Risk-deficit disorder | | 1. "Eager and Little have coined the term “Risk Deficit Disorder” to describe a set of problems that children can experience resulting from attempts to remove risk from their lives." pg 3141 | 1. Multiple, NA | 1. Brussoni 2012 |
| 66. | Risk-taking | | 1. "Additionally, risk-taking and risky play experiences involve a borderline feeling of being out of control and a sense that one is overcoming fear. Moreover, risk-taking and risky play involve exposing oneself to hazards and the possibility of physical harm, with a perceived uncertainty regarding the outcome of the risky behaviour." pg 376   " Sandseter (2009a) identified two types of risk characteristics in children’s play. First, environmental characteristics are features of the environment, such as the height or steepness of a hill, that increase the chance of a child’s possible injury as a result of play. The amount of supervision by teachers and staff is considered an environmental characteristic. Second, individual characteristics are features of how play is carried out by the child, such as the height a child climbs, concentration or body control. Therefore, when examining children’s risk-taking and risky play experiences, it is essential to consider the types of activities they are engaging in, alongside the environmental and individual characteristics of those endeavours. Likewise, it is important to consider the individual differences with which children approach and engage in risk-taking and risky play experiences." pg 376   2. (children’s risk-taking) "...as the child’s uncertainty about outcomes; balancing scary-funny feelings and discomfort; trying new things and challenging oneself; testing limits in developmentally and individually appropriate ways; and, learning from failure" 3. “‘risk-taking’ often has negative connotations, the reality is that the willingness to engage in some risky activities provides opportunities to learn new skills, try new behaviours and ultimately reach our potential.” 4. “The findings from this present study support Ball’s (2002) conclusion that risk-taking in play allows children to learn risk assessment and develop a sense of risk and understanding individual characteristics that may contribute to injury risk is important in the provision of safe play environments for children.” | 1. Canada, 4-5 years 2. Multiple, NA 3. NA, children 4. Australia, 4-5 years | 1. Coe 2017 2. Alden 2019 3. Little 2008 4. Little 2010 |
| 67. | Risk perception | | 1. Identification of hazards | 1. Australia, 4-5 years4 | 1. Little 2010 |
| 68. | Scaffolding | | 1. "This research showed that adults played a fundamental role in scaffolding children’s learning only at times when the child needed assistance, suggesting that whilst not all activity was child-initiated, the support they received was. " pg 36 | 1. UK, 4-9 years | 1. Coates 2019 |
| 69. | Sensation seeking | | 1. “Sensation seeking, or the desire for intense sensations and novel experiences, is associated with a readiness to take risks (Horvath and Zuckerman 1993).” | 1. Australia, 4-5 years | 1. Little 2010 |
| 70. | Sense of place | | 1. "Cross (2001) examined the multiple ideas of what sense of place means across five separate fields. Collectively they were all very similar and contained the common themes of it being an emotional bond/connection/attachment that provides "understanding of and relation to the environment" (p. 1) and stimulates learning and discovery. It is this connection to place that gives a sit spot its magic. Having a sense of and connection to place gives people a strong feeling of belonging, which fulfills the constant human desire to belong somewhere. Once you gain that sense of place, familiarity and comfort of your sit spot, it is then that you learn the most from it. That connectedness motivates you to return, and make that place your own." pg 28 2. Figure 1 Influences impacting on students’ feelings and sense of place. pg 305   Figure 2 Summary of factors contributing to sense of place pg 306  "An emerging sense of place through place attachment and place meaning became evident." pg 305 | 1. NA, Children 2. Australia, 10-12 years | 1. Carruthers 2011 2. Cumming 2015 |
| 71. | Sensory perception/engagement/experience/learning | | 1. (sensory perception)"...which is an ancestral form of cognition, is apt for environmental education, particularly out-of-doors. It is through the senses that infants first learn about their environment. The importance of sensory perception in an infant’s world is analogous to the vital role senses played in the early survival of the human species." pg. 7   "...'other’ senses apart from the traditional five...external or physical senses...Balance, equilibrium, intuition and other self-orienting tools could also play a role in making sense of and understanding the outdoor environment." pg. 8  "In addition to clarifying cause-and-effect relationships between people and the environment, sensory perception makes students aware (literally, sensitive to) their own biological connections to the environment." pg 10  (sensory learning)"... learning opportunity from engaging, diverse, and intertwined auditory, olfactory, tactile, taste, and visual experiences." pg 13   2. (sensory experiences)"...sensory experiences for children visiting a botanical garden emphasizing how touch, sight, sound, smell, and taste were important in children’s recollection of meaningful experiences at the garden, including the colors of flowers, the sounds of bees, the feeling of rain on the body, the taste of berries and the sight of butterflies. Similarly, James and Bixler (2008) identified the importance of novelty and sensory experiences, particularly touch, among children attending a coastal beach environmental education program. The ability to have direct sensory experience of the landscape, such as sand, shells, bird parts, and live reptiles enhanced children’s learning and provided an opportunity for intimate interaction with natural objects and living creatures, making the experience meaningful." pg 16 | 1. USA, NA 2. Sweden & Norway, Adults (Sweden), 1-6 years (Norway) | 1. Auer 2008 2. Beery 2018 |
| 72. | Social emotional development | | 1. Figure 1, "Emphasis on: social and emotional competence." pg 10 | 1. NA, early years | 1. Carter 2016 |
| 73. | Social forestry | | 1. "The multipurpose and multibeneficial aspects that woodland can bring to enhance the quality of life in humans (O’Brien, 2009)." p.1108 | 1. UK, college students | 1. Mata 2016 |
| 74. | Solitary-active behaviour | | 1. "solitary-active behavior (sensorimotor and/or dramatic activity acted out by oneself despite being in social company; Coplan, Rubin, Fox, Calkins, & Stewart, 1994; see Rubin et al., 2002, for a review)." p.812-813 | 1. USA, mean 59.63 years | 1. Nelson 2008 |
| 75. | Structure | | 1. "I taught PE in a structured manner to make the most of each lesson. Warm up, skill learning, game or activity to practce the skill, cooling down, etc. were the components of my PE lessons which were guided by the learning objectives for the lesson. Such structured objectives-or outcome-based and sequenced-based teaching and learning was the norm..." pp. 477 (McEvilly 13) | 1. Singapore, NA | 1. Hussain 2018 |
| 76. | Supervision | | 1. "...permissive, observant, receptive and supportive, that involves a lot more preparation and a lot less instruction or correction in the field..." 2. "Supervision in the field of education has been defined as adults who are alert, are aware, know the play rules, and intervene when inappropriate behaviors occur (Thompson, Hudson, & Olsen, 2007). " p. 33 | 1. Canada, NA 2. USA, early childhood | 1. Alden 2019 2. Olsen 2011 |
| 77. | Playground supervision | | 1. Under taken by teachers. 2. "The zone model of playground supervision (ZoMPS), developed by members of the PPG Playground Committee, defines staff child care roles and structures adult support during outdoor playtime." p.13 | 1. Australia/Turkey, primary school students 2. USA, young children | 1. Chancellor 2014 2. Kem 2007 |
| 78. | System thinking | | 1. "Eight emergent hierarchic characteristics of system thinking in the context of earth systems as: (1) The ability to identify the components of a system and processes within the system; (2) The ability to identify simple relationships between or among the system’s components; (3) The ability to identify dynamic relationships within the system; (4) The ability to organize the systems’ components, processes, and their interactions, within a framework of relationships; (5) The ability to identify cycles of matter and energy within the system—the cyclic nature of systems; (6) The ability to recognize hidden dimensions of the system—to understand natural phenomena through patterns and interrelationships not seen on the surface; (7) The ability to make generalizations—to solve problems based on understanding systems’ mechanisms; (8) The ability to think temporally: retrospection and prediction. Understanding that some of the presented interaction within the system took place in the past, while future events may be a result of present interactions." pg. 541 | 1. Israel, 4^th^ grade | 1. Assaraf 2010 |
| 79. | Wellbeing | | 1. “Health is a state of complete physical, mental and social well-being, and not merely the absence of disease or infirmity" pp. 24; Well-being now refers to what constitutes the ‘healthy’ physical, mental and social state of individuals. The concept of well-being has emerged as a significant indicator in health discourse of the now accepted broader reach of notions of health." pp. 24 | 1. UK, 8-9 years | 1. Humberstone 2009 |
| Back to Table of Contents | | | | |  |
|  | | | | |  |
| **Element/Feature** | | | | |  |
| 1. | Abiotic elements | | 1. "e.g., rocks, soil, wind, rain, etc." | 1. USA, K-8 children | 1. Ashmann 2018 |
| 2. | Balance-on-able features | | 1. "Affords balancing. Risky play category: Great heights" pg 6429 | 1. Multiple, NA | 1. Brussoni 2015 |
| 3. | Big job | | 1. "A big job is a useful, helpful task that requires several people to work together. The need for the task and the improvement that results are obvious to children. A big job reequires physical exertion, thinking and problem solving, and functional use of language." pp.87 | 1. USA, NA | 1. Jones 2005 |
| 4. | Climbable features | | 1. "Affords climbing. Risky play category: Great heights." pg 6429 | 1. Multiple, NA | 1. Brussoni 2015 |
| 5. | Collecting | | 1. "...the gathering of items from nature primarily for play, exploration, and interest but may include occasional consumption as well. Collecting refers to the self-motivated accumulation of natural objects, such as rocks, shells, feathers, and plant parts. The definition also includes temporary collections of living creatures as well as consumable foraging. These uses are differentiated from non-recreational purposes in which collecting or foraging may be used to meet basic sustenance or cultural needs."pg 13-14 | 1. Sweden (adults) & Norway (1-6 years) | 1. Beery 2018 |
| 6. | Dangerous tools | | 1. "Can lead to injuries and wounds. Example: Cutting tools (e.g., knives, saws, or axes), strangling tools (e.g., ropes)." pg 6425 | 1. Multiple, NA | 1. Brussoni 2015 |
| 7. | Dangerous elements | | 1. "Where children can fall into or from something. Example: Cliffs, water, fire pits, trees." pg 6425 | 1. Multiple, NA | 1. Brussoni 2015 |
| 8. | Den making | | 1. "Den-making is an opportunity for children to create an enclosed area where they can create space for themselves. The process of den-making and the end product provides children with a focus for communication and building social relationships. Den-making also provides opportunities to alter and change their spaces as the materials can be recycled for different play at different times." pg 556   " In outdoor environments resources refer to the natural materials available and in den-making these usually consist of sticks, branches or leaves. Resources for den-making are also provided by early years practitioners and usually consist of items such as blankets and sheets to enable an enclosed space to be achieved or a more comfortable area for children to sit." pg 556-557 | 1. UK, 3-5 years | 1. Canning 2010 |
| 9. | Disappear/get lost | | 1. . "Where children can disappear from the supervision of adults or get lost alone. Example: Exploring alone, playing alone in unfamiliar environments, general independent mobility, or unsupervised play." pg 6425 | 1. Multiple, NA | 1. Brussoni 2015 |
| 10. | Enclosure/restriction | | 1. "Enclosure/restrictions (e.g., differently sized sub-spaces or private spaces where children can explore on their own or hide away from larger groups, mobility license). Affords getting lost, disappearing. Risky play category: Disappear/get lost" pg 6429 | 1. Multiple, NA | 1. Brussoni 2015 |
| 11. | Flat, relatively smooth surfaces | | 1. "Affords running, RTP [rough and tumble play]. Risky play category: High speed, RTP" pg 6429 | 1. Multiple, NA | 1. Brussoni 2015 |
| 12. | Fixed climbing structure | | 1. "The design of these structures varied, with some formed by tyres fixed to a frame, and others consisting of large timber structures with stairs, platforms and frequently some form of enclosure, while others were made of metal." pg 45 | 1. Australia, NA | 1. Davies 1996 |
| 13. | Fixed play structures | | 1. "e.g., climbing structures, cubby houses, trampolines" | 1. Australia, 2-5 years | 1. Armstrong 2019 |
| 14. | Graspable/detachable objects | | 1. "Affords throwing, striking, and fencing. Risky play category: RTP" pg 6429 | 1. Multiple, NA | 1. Brussoni 2015 |
| 15. | Great heights | | 1. "Danger of injury from falling. Example: Climbing/jumping from surfaces, balancing/playing on high objects (e.g., playground equipment), hanging/swinging at great heights." pg 6425 | 1. Multiple, NA | 1. Brussoni 2015 |
| 16. | High speed | | 1. "Uncontrolled speed and pace that can lead to collision with something (or someone). Example: Swinging at high speed." pg 6425 | 1. Multiple, NA | 1. Brussoni 2015 |
| 17. | Jump-down-off features | | 1. "Affords jumping down. Risky play category: Great heights" pg 6429 | 1. Multiple, NA | 1. Brussoni 2015 |
| 18. | Loose parts | | 1. "Affordances can also be provided through loose parts, open-ended, moveable materials with multiple potential uses and outcomes (such as sticks, rope, cardboard boxes, etc.) to foster creativity and engagement." 2. " theory of ‘loose parts’ which linked the degree of inventiveness and creativity that children engage with in their environment to the different resources provided within it." pg 556 3. "The term ‘Loose Parts’ is based on Nicholson’s (1971) Theory of Loose Parts. Moveable components in children’s play rather offer open-ended play as well as many forms of usage (Nicholson, 1971)." 4. "The ‘Theory of Loose Parts’ was promulgated by Simon Nicholson. He stated that ‘In any environment, both the degree of inventiveness and creativity, and the possibility of discovery, are directly proportional to the number and kind of variables within it’ (1971, 30)." p.515 5. "Loose parts may be manufactured or natural materials (Frost, 1997) or recyclable objects (Clements, 2004). Manufactured items (boxes, blocks, cloth, tools, toys, dramatic play props) and natural items (bark, seeds, stones, leaves, vines, etc.) provide flexibility, novelty, and adaptability that extend play (Wellhousen, 2002; Wilson, 2008)." p.31 6. " Loose parts consist of materials that children can pick up, throw, kick, examine, arrange, and chase. It was stated by two early childhood outdoor designers that loose parts (such as balls and dramatic play materials) make the outdoor play environment complete (Dempsey & Strickland, 1999) and increase physical activity with young children (Hannon & Brown, 2008). " p.15 | 1. Canada, NA 2. UK, 3-5 years 3. Germany, 5-6 years 4. New Zealand, 12-60 months 5. USA, NA 6. USA, early childhood | 1. Alden 2019 2. Canning 2010 3. Luchs 2013 4. Mawson 2014 5. McClintic 2015 6. Olsen 2013 |
| 19. | Manufactured equipment | | 1. Examples: "...manufactured equipment; outdoor toys (buckets, shovels, trucks, balls), swings, sandboxes, climbing racks" pg 4 | 1. Norway, 3-5 years | 1. Bjorgen 2016 |
| 20. | Math trail | | 1. A typical trail consists of a sequence of designated sites along a planned route where students stop to explore math 2. in the environment (Cross 1997; Richardson 2004). Math trails make mathematics come alive for children by engaging them cognitively, physically, and emotionally."pg 403 | 1. UK (England), 7-11 years | 1. English 2010 |
| 21. | Natural features | | 1. "... flowers, herbs or vegetables..." 2. Natural features include: sandpitch, digging patch, bushy areas where children can play, trees for climing, grassed areas for play, flower garden area, food garden area, pond/water feature, recycling facility, nature trail, bird box/table, wildlife habitats, Weather station, wildflower area, composting area. pg 47 | 1. Australia, 2-5 years 2. Australia/Turkey,primary school students | 1. Armstrong 2019 2. Chancellor 2014 |
| 22. | Natural material | | 1. Examples: “…natural materials; small trees, a varied surface of grass, sand and asphalt, and varied terrain with small hills” pg 4 2. "engaging in imaginative play scenarios, it takes time to develop and engage in such play" pg 51 | 1. Norway, 3-5 years 2. Australia/Turkey, primary school students | 1. Bjorgen 2016 2. Chancellor 2014 |
| 23. | Nature-related features | | 1. "The aim of this article is to demonstrate that primary schools, if re-designed with nature-related features, can be effective environments to promote PA in children. Nature, in this context, is defined as a physical environment consisting of plant life, water bodies, animal life, and other features consistent with the natural world." p.955 | 1. Multiple (review), childhood | 1. Sharma-Brymer 2016 |
| 24. | Obstacle course | | 1. " develop a means for children to understand spatial awareness in connection with their own bodies, offer adventure, enhance gross-motor skills and cooperative skills, provide opportunities for experimentation and exploration, offer creative and dramatic-play outlets, enhance positive self-esteem, and provide challenge." pg.18 | 1. USA, NA | 1. Griffin 1998 |
| 25. | Outdoor surfaces | | 1. " Because many children get hurt when they fall while running and jumping from equipment, the surfaces that they fall on need to be carefully prepared in order to prevent serious injuries.” Pg 154 | 1. NA, NA | 1. Taylor 1996 |
| 26. | Playground buddy benches | | 1. "...a park bench sitting on the playground under a colorful wooden rainbow. A Ralph Waldo Emerson quote stating, “The only way to have a friend, is to be one,” scrolled in an arch under the rainbow’s violet band. Text under the image of the decorated bench announced, “The buddy bench is a place where friendships begin.” The card explained children who do not know who to play with at recess are invited to sit on the bench; children playing on the playground are encouraged to invite anyone sitting on the bench to play. The buddy bench was presented as “a simple solution designed to eliminate playground loneliness.” " pg 9 | 1. USA, Kinder to 4^th^ G | 1. Clarke 2018 |
| 27. | Play structure | | 1. "These new structures engage children in multisensory experiences that stimulate and challenge their developing, physical, mental, and social abilities." pp.36 | 1. USA, NA | 1. Jacobs 2008 |
| 28. | Play equipment | | 1. "Play equipment is an important element to consider with regard to the design of the playground. Usually climbing equipment tends to be in one sport, but ideally it should be distributed in several places around the playground." pp.26 | 1. Canada, children | 1. Lambert 1999 |
| 29. | Playground equipment | | 1. . "swing, slides, climbing, etc." pg 8 1. "Play equipment is designed to provide physical challenge as well as social interaction." pp.28; "Play equipment provides opportunities for integration, especially when programmed with other activities." pp. 29 | 1. USA, NA 1. NA, NA | 1. Burriss 2011 1. Goltsman 1997 |
| 30. | Sandbox | | 1. "The use of sand in children’s programs has a long history, starting with a series of sand gardens developed in Berlin in the 1880s. These play areas, consisting of piles of sand encased in wooden borders, inspired the construction of America’s first supervised playgrounds: ten sand gardens in poor areas of Boston in 1887." pp. 221-22 | 1. USA, 3-4 years | 1. Jarrett 2010 |
| 31. | Slopes and slides | | 1. "Affords sliding, running. Risky play category: High speed" pg 6429 | 1. Multiple, NA | 1. Brussoni 2015 |
| 32. | Sit spot | | 1. "...a special place within nature that you are connected to and return to often..." pg 26   "...when you go to it your attitude overflows with childlike curiosity, discovery, and uninhibited playfulness..." pg 27  "In the sit spot children are given the opportunities to have moments of silence, solitude and stillness. Children are invited to explore their curiosity and the natural wonder as "nature  itself invites quiet contemplation or intense fascination". They are able to get eye-to-eye with a diversity of lifeforms and weather-patterns; the place where they face their fears—of bugs, being alone, of the dark—and grow past them; and the place where they meet nature as their home." pg 27 | 1. NA, Children | 1. Carruthers 2011 |
| 33. | Swing-on-able features | | 1. "Affords swinging. Risky play category: High speed, great heights." pg 6429 | 1. Multiple, NA | 1. Brussoni 2015 |
| 34. | Tramping | | 1. "The tramping movement (involving camping or hiking on weekends) is a particular Czech historical and cultural phenomenon, which fulfilled the demands of young people for freedom in natural surroundings, and as a protest against imposed society rules and restrictions. Tramping, as with scouting, developed its own culture, slang, songs, clothes, flag, anthem, rituals, magazines, literature, sports (especially canoeing and kayaking) and small settlements (cottage colonies). These special settlements with wooden cabins and simple places for camping with a campfire were built in beautifulnatural environments, especially near rivers around Prague and other bigger towns." p.69 | 1. New Zealand, adults | 1. Martin 2011 |
| 35. | Turistika | | 1. "The original form of turistika involved active movement on foot (walking, hiking), but now includes cycling, canoeing, skiing, and mountaineering. However, turistika also involves other outdoor and cultural activities (learning about nature; local history and sights; life of local people; mushroom and berry picking; music, artistic creativity, and social entertainment). Some tourism definitions do involve travelling and movement away from normal environments (Mason, 2003), but turistika is specific to the Czech context (Turcová et al., 2005). Scouting and stays in nature connected with camping can also be included as turistika (Martin, Turcová, & Neuman, 2007)." p. 69 | 1. New Zealand, adults | 1. Martin 2011 |
| 36. | Water play enhancers | | 1. " Kindle interest and stimulate the imagination by changing water-play equipment frequently. Enhance play with objects from home, school, and nature." Examples: " stoppers aquarium nets soup ladle wood scraps bulb baster lids slotted spoon empty spice cans sieve milk cartons strainer..." pg 30 | 1. NA (maybe USA), NA | 1. Crosser 1994 |
| Back to Table of Contents | | | | | |

**Supplementary Table 2. List of terms with no definitions/descriptions.**

(*Note: terms don’t exist in Table 1 are highlighted in green*)

| Term | Reference |
| --- | --- |
| 1. Active play | 1. Bartlett 1997 2. Brewer 2011 3. Brussoni 2012 4. Burdette 2005 5. Clarke 2018 6. Clements 2014 7. Davies 1996 8. Spencer-Cavaliere 2009 |
| 2. Active free play | 1. Ceciliani 2013 |
| 3. Active physical play | 1. Little 2015 |
| 4. Active transportation | 1. Brussoni 2015 |
| 5. Adaptive play | 1. Couper 2013 |
| 6. Adult-led play | 1. Dinkel 2019 |
| 7. Adventure education | 1. Karppinen 2012 2. Mygind 2019 |
| 8. Adventure play | 1. Chancellor 2014 |
| 9. Adventure playground | 1. Brussoni 2015 |
| 10. Adventure program | 1. Lamorey 2013 2. Schreuder 2014 |
| 11. Adventure therapy | 1. Brewer 2011 2. Mygind 2019 |
| 12. Adventurous activities | 1. Brodin 2009 |
| 13. Affordances | 1. Desroachers 2001 2. Kiewra 2016 |
| 14. Affordances of playground equipment | 1. Miller 2017 |
| 15. Authentic play | 1. McClain 2016 |
| 16. Backseat generation | 1. Brussoni 2012 |
| 17. Backyard | 1. Bohn-Goldbaum 2013 |
| 18. Biophilia | 1. Chiumento 2018 |
| 19. Built environment | 1. Beyer 2015 2. Bringolf-Isler 2010 3. Brown 2017 |
| 20. Chants and rhythms play | 1. Chancellor 2014 |
| 21. Child centred | 1. Brussoni 2012 |
| 22. Child centred practices | 1. Canning 2010 |
| 23. Child dedicated | 1. Brussoni 2012 |
| 24. Child directed | 1. Coe 2017 |
| 25. Childhood wildland play | 2. Vadala 2007 |
| 26. Child initiated learning | 1. Coates 2019 2. Olsen 2017 |
| 27. Child initiated play | 1. Brown 2009 2. Davies 1996 3. Olsen 2017 |
| 28. Cognitive learning | 1. Nichols 1989 |
| 29. Collaborative work | 2. Brodin 2009 |
| 30. Cooperative learning | 1. Broda 2002 |
| 31. Cooperative play | 1. Cumming 2015 |
| 32. Creative play | 1. Beyer 2015 (Soc Sci Med) 2. Couper 2013 3. Olsen 2011 |
| 33. Creative social play | 1. Makenzie 2017 |
| 34. De-natured childhood | 1. Coe 2017 |
| 35. Direct instruction | 1. Bentley 2010 |
| 36. Dirt/mud play zone | 1. Davies 1996 |
| 37. Dramatic play | 1. Ceciliani 2013 2. Davies 1996 3. Olsen 2017 |
| 38. Ecology education | 1. Manni 2017 |
| 39. Education outside the classroom | 1. Cramp 2008 2. Mygind 2019 |
| 40. Education for sustainable development | 1. Beery 2018 |
| 41. Education literacy | 1. Braun 2018 |
| 42. Education through outdoors | 1. Marsh 2000 |
| 43. Educational playgrounds | 1. Montessori 2013 |
| 44. Environmental education | 1. MacQuarrie 2018 2. Marsh 2000 3. Rios 2014 4. Ray 2019 |
| 45. Environmental ethic | 1. Carruthers 2011 |
| 46. Environmental intergenerational learning | 1. Zimmerman 2013 |
| 47. Environmental learning | 1. Beattie 2015 2. Rios 2014 |
| 48. Environmental literacy | 1. Carter 2016 |
| 49. Erlebnistherapie | 1. Karppinen 2012 |
| 50. Experiential education | 1. Karppinen 2012 2. McKenzie 2013 |
| 51. Experiential learning | 1. McKenzie 2013 2. Schreuder 2014 3. Sproule 2013 4. Turtle 2015 |
| 52. Experiential task | 1. Brodin 2009 |
| 53. Fantasy play | 1. Davies 1996 |
| 54. Fitness and motor skill learning | 1. Spencer-Cavaliere 2009 |
| 55. Free-choice play | 1. Beattie 2015 |
| 56. Free play | 1. Bentley 2010 2. Beyer 2015 3. Bohn-Goldbaum 2013 4. Brussoni 2018 5. Bundy 2011 6. Burdette 2005 7. Coates 2019 (distinct, free play) 8. Couper 2013 9. Kos 2013 10. Little 2010 11. Luchs 2013 12. McArdle 2013 13. Sandseter 2012 14. Sharma-Brymer 2016 |
| 57. Outdoor free play (free play outdoors) | 1. Chancellor 2014 2. Christian 2019 |
| 58. Forest school | 1. Beattie 2015 2. Bentsen 2009 3. Bentsen 2010 4. Little 2011 5. Mata 2016 |
| 59. Forest kindergartens | 1. Bentsen 2012 2. Carter 2016 |
| 60. Formal and informal form games with rules | 1. Chancellor 2014 |
| 61. Friluftsliv | 1. Martin 2011 2. Mutz 2016 |
| 62. Game-based learning | 1. Coates 2019 |
| 63. Green play | 1. Largo-Wight 2018 |
| 64. Green space | 1. Bentsen 2012 2. Bentsen 2010 3. Beyer 2015 |
| 65. Green space planners/managers | 1. Bentsen 2010 |
| 66. Hands-on experience | 1. Coe 2017 |
| 67. Hands-on learning | 1. Auer 2008 2. Broda 2002 |
| 68. Imaginative play | 1. Chancellor 2014 2. Clements 2014 |
| 69. Independent mobility | 1. Brussoni 2015 |
| 70. Inquiry | 1. Bentley 2010 |
| 71. Inquiry based | 1. Brooke 2017 |
| 72. Learn by doing | 1. Brodin 2009 |
| 73. Learning in natural environments | 1. Morag 2013 |
| 74. Learning through play | 1. Zamani 2016 |
| 75. Loose parts | 1. Beyer 2015 (Soc Sci Med) 2. Chancellor 2014 3. Dinkel 2019 |
| 76. Modelling | 1. Couper 2013 |
| 77. Natural learning environment | 1. Coe 2017 |
| 78. Natural play | 1. Luchs 2018 |
| 79. Natural play spaces | 1. Luchs 2013 |
| 80. Nature based early years program | 1. Coe 2017 |
| 81. Nature based learning | 1. Coe 2017 2. MacQuarrie 2015 |
| 82. Nature based preschools | 1. Carter 2016 |
| 83. Nature circle | 1. Brodin 2009 |
| 84. Nature-deficit disorder | 1. Brown 2017 2. Coe 2017 |
| 85. Nature environment | 1. Chiumento 2018 |
| 86. Nature excursion | 1. Brodin 2009 |
| 87. Nature free play | 1. Coates 2019 |
| 88. Nature Kindergartens | 1. Beattie 2015 2. MacQuarrie 2015 3. Masters 2018 |
| 89. Nature oriented playscapes | 1. Luchs 2013 |
| 90. Nature play | 1. Brown 2017 2. McClain 2016 |
| 91. Nature playground | 1. Brown 2017 |
| 92. Nature schools | 1. Bentsen 2012 |
| 93. Neighbourhood | 1. Bohn-Goldbaum 2013 |
| 94. Open-air schools | 1. Bentsen 2012 |
| 95. Outdoor active play | 1. Nguyen 2018 |
| 96. Outdoor active free play | 1. Byrd-Williams 2019 |
| 97. Outdoor activities | 1. Beyer 2015 2. Beyer 2015, Soc Sci Med |
| 98. Outdoor adventure programme | 1. Mygind 2019 2. Norton 2014 3. Sproule 2013 |
| 99. Outdoor autonomy | 1. Brussoni 2018 |
| 100. Outdoor classroom | 1. Largo-Wight 2018 |
| 101. Outdoor early childhood programmes | 1. Coe 2017 |
| 102. Outdoor education | 1. Bentsen 2012 2. Bentsen 2009 3. Coates 2019 4. Cramp 2008 5. Karppinen 2012 6. Largo-Wight 2018 7. Liu 2017 8. Mygind 2019 9. Nawaz 2014 10. Rios 2014 11. Quibell 2017 12. Ray 2018 13. Rynders 1990 14. Scott 2014 15. Stan 2009 |
| 103. Outdoor education activities | 1. Liu 2017 |
| 104. Outdoor environment | 1. Davies2013 |
| 105. Outdoor environmental education | 2. Nelson 2013 |
| 106. Outdoor family learning | 1. Zimmerman 2016 |
| 107. Outdoor free play | 1. Largo-Wight 2018 |
| 108. Outdoor Kindergarten | 1. Beery 2018 2. Nah 2015 |
| 109. Outdoor learning | 1. Bentsen 2010 2. Burriss 2011 3. Cumming 2015 4. Largo-Wight 2018 5. MacQuarrie 2018 6. MacQuarrie 2015 7. Mads 2019 8. Morag 2013 9. Nawaz 2014 10. Nedovic 2013 11. Nugent 2015 12. O’Brien 2009 13. Olsen 2013 14. Olsen 2011 15. Rios 2014 16. Sproule 2013 |
| 110. Outdoor nature play | 17. Chiumento 2018 |
| 111. Outdoor pedagogy | 75. Nah 2015 |
| 112. Outdoor personal development course | 1. Mygind 2019 |
| 113. Outdoor play | 1. Aarts 2010 2. Barton 2015 3. Bjorgen 2015 4. Bridges 2019 5. Brussoni 2018 6. Brussoni 2012 7. Bundy 2011 8. Ceciliani 2013 9. Chancellor 2014 10. Christian 2019 11. Coates 2019 12. Coe 2017 13. Desrochers 2001 14. Kern 2007 15. Kos 2013 16. Larouche 2019 17. Larrea 2019 18. Levin 2012 19. Little 2015 20. Little 2017 21. Little 2012 22. Little 2015 23. Little 2011 24. Lovell 1985 25. Lu 2019 26. Luchs 2013 27. Luchs 2018 28. Lysklett 2017 29. Marchant 2019 30. Masters 2018 31. Mawson 2014 32. Maynard 2007 33. Maynard 2013 34. McClain 2016 35. Nah 2015 36. Nawaz2014 37. Naylor 1985 38. Nedovic 2013 39. Niehues 2013 40. Nordahl 2016 41. Nugent 2015 42. O’Brien 2009 43. OIsen 2013 44. Olsen 2017 45. Pate 2013 46. Pearson 2012 47. Puett 2019 48. Ray 2018 49. Raustorp 2012 50. Rios 2014 51. Sandseter 2012 52. Scott 2013 53. Sharp 2018 54. Staempfli 2009 55. Scott 2013 |
| 114. Outdoor play spaces | 1. Desrochers 2001 |
| 115. Outdoor preschool playground | 1. Nicaise 2012 |
| 116. Outdoor programs | 2. Ngo 2014 |
| 117. Outdoor recreation | 1. Bentsen 2009 |
| 118. Outdoor risky play | 1. McFarland 2018 2. Sandseter 2012 |
| 119. Outdoor science learning | 1. McClain 2016 2. Rios 2014 3. |
| 120. Outdoor science school | 1. Mygind 2019 |
| 121. Outdoor teaching | 1. Coates 2019 |
| 122. Outdoor therapy | 1. Brewer 2011 |
| 123. Outdoor time | 1. Levin 2012 2. Ngo 2014 |
| 124. Outdoor walks | 1. Beyer 2015 |
| 125. Overseer | 1. Coates 2019 |
| 126. Parallel play | 1. Davies 1996 |
| 127. Passive learning | 1. Broda 2002 |
| 128. Peer group play | 1. Dinkel 2019 |
| 129. Physical play | 1. Dinkel 2019 |
| 130. Physical activity play | 2. Luchs 2018 |
| 131. Place based | 1. Bentley 2010 |
| 132. Place based pedagogy | 1. Lloyd 2018 |
| 133. Play-based learning | 1. Coates 2019 |
| 134. Playgrounds | 1. Brodin 2009 2. Brussoni 2015 3. Brussoni 2012 4. Clarke 2018 5. Corbett 2017 6. Couper 2011 7. Couper 2013 |
| 135. Playground structures | 1. Dinkel 2019 |
| 136. Playground rule making | 1. Chancellor 2014 |
| 137. Play for learning | 1. Brodin 2009 |
| 138. Play in natural environments | 1. Miller 2017 |
| 139. Practical skills | 1. Mata 2016 |
| 140. Pretend | 1. Bentley 2012 |
| 141. Pretend play | 1. Couper 2013 |
| 142. Problem-based learning | 1. Brodin 2009 |
| 143. Problem-based play | 1. Coates 2019 |
| 144. Professional scaffolding | 1. Brussoni 2012 |
| 145. Proximate access to nature | 1. Beery 2018 |
| 146. Recess | 1. Couper 2013 |
| 147. Risky play | 1. Brown 2017 2. Chancellor 2014 3. Coates 2019 4. Kos 2013 5. Lim 2017 6. Little 2015 7. Little and Sweller 2015 8. Sharma-Brymer 2016 |
| 148. Risky outdoor play | 1. Little 2015 |
| 149. Rough-and-tumble play | 1. Bentley 2012 2. Brown 2009 3. Chancellor 2014 4. Davies 1996 |
| 150. School-based outdoor education | 1. Mads 2019 |
| 151. School-based outdoor learning | 1. Sahrakhiz 2018 |
| 152. School ground gardening | 1. Lucas 2010 |
| 153. School ground naturalisation | 1. Lucas 2010 |
| 154. School ground restoration | 1. Lucas 2010 |
| 155. Scientific and sensory play | 1. Chancellor 2014 |
| 156. Self-directed play | 1. Beery 2018 |
| 157. Social play | 1. Davies 1996 2. Lovell 1985 3. |
| 158. Sociodramatic play | 1. Davies 1996 |
| 159. Sociomotor play | 2. Ceciliani 2013 |
| 160. Spontaneous play | 1. Bundy 2011 2. Burriss 2011 3. Couper 2013 |
| 161. Student-led learning | 1. Bradshaw 2018 |
| 162. Symbolic play | 2. Couper 2013 |
| 163. Teacher-led play | 1. Brown 2009 |
| 164. Traditional playground | 1. Brussoni 2015 2. Brussoni 2012 3. Christian 2019 |
| 165. Transportation infrastructure | 1. Beyer 2015 |
| 166. Udeskole | 1. Mads 2019 |
| 167. Unstructured | 1. Beyer 2015 |
| 168. Unstructured environments | 1. Beyer 2015 (Soc Sci Med) |
| 169. Unstructured outdoor activities | 1. Nicksic 2018 |
| 170. Unstructured outdoor free play | 1. Wolfenden 2016 |
| 171. Unstructured outdoor play | 1. Brussoni 2018 2. Larouche 2016 |
| 172. Unstructured play | 1. Burriss 2011 2. Canning 2010 3. Ceciliani 2013 |
| 173. Unstructured physical activity | 1. Brussoni 2015 |
| 174. Unsupervised play | 1. Brussoni 2015 2. Burdette 2005 |
| 175. Vigorous play | 1. Ceciliani 2013 |
| 176. Wetland ecotourism programs | 1. Liu 2017 |
| 177. Wild play | 1. Beery 2018 |
| 178. Wilderness challenge programmes | 1. Schreuder 2014 |
| 179. Wilderness programs | 1. Berman 2013 |
| 180. Wilderness schooling | 1. Coates 2019 |
| 181. Wilderness therapy | 1. Mygind 2019 2. Schreuder 2014 |
